# Effectiveness of Secondary Risk–Reducing Strategies in Patients With Unilateral Breast Cancer With Pathogenic Variants of BRCA1 and BRCA2 Subjected to Breast-Conserving Surgery: Evidence-Based Simulation Study

**DOI:** 10.2196/37144

**Published:** 2022-12-29

**Authors:** Jelena Maksimenko, Pedro Pereira Rodrigues, Miki Nakazawa-Miklaševiča, David Pinto, Edvins Miklaševičs, Genadijs Trofimovičs, Jānis Gardovskis, Fatima Cardoso, Maria João Cardoso

**Affiliations:** 1 Institute of Oncology, Department of Surgery, Breast Unit Pauls Stradiņš Clinical University Hospital Riga Stradiņš University Riga Latvia; 2 Information and Health Decision Sciences of the Faculty of Medicine University of Porto Porto Portugal; 3 Institute of Oncology Riga Stradiņš University Riga Latvia; 4 Breast Cancer Unit Champalimaud Cancer Center Lisbon Portugal; 5 Faculty of Medicine Rīga Stradiņš University Riga Latvia; 6 Department of Surgery, Faculty of Medicine Pauls Stradins Clinical University Hospital Rīga Stradiņš University Riga Latvia

**Keywords:** BRCA1 and BRCA2, secondary prophylactic strategies, breast-conserving therapy, breast cancer

## Abstract

**Background:**

Approximately 62% of patients with breast cancer with a pathogenic variant (*BRCA1* or *BRCA2*) undergo primary breast-conserving therapy.

**Objective:**

The study aims to develop a personalized risk management decision support tool for carriers of a pathogenic variant (*BRCA1* or *BRCA2*) who underwent breast-conserving therapy for unilateral early-stage breast cancer.

**Methods:**

We developed a Bayesian network model of a hypothetical cohort of carriers of *BRCA1* or *BRCA2* diagnosed with stage I/II unilateral breast cancer and treated with breast-conserving treatment who underwent subsequent second primary cancer risk–reducing strategies. Using event dependencies structured according to expert knowledge and conditional probabilities obtained from published evidence, we predicted the 40-year overall survival rate of different risk-reducing strategies for 144 cohorts of women defined by the type of pathogenic variants (*BRCA1* or *BRCA2*), age at primary breast cancer diagnosis, breast cancer subtype, stage of primary breast cancer, and presence or absence of adjuvant chemotherapy.

**Results:**

Absence of adjuvant chemotherapy was the most powerful factor that was linked to a dramatic decline in survival. There was a negligible decline in the mortality in patients with triple-negative breast cancer, who received no chemotherapy and underwent any secondary risk–reducing strategy, compared with surveillance. The potential survival benefit from any risk-reducing strategy was more modest in patients with triple-negative breast cancer who received chemotherapy compared with patients with luminal breast cancer. However, most patients with triple-negative breast cancer in stage I benefited from bilateral risk-reducing mastectomy and risk-reducing salpingo-oophorectomy or just risk-reducing salpingo-oophorectomy. Most patients with luminal stage I/II unilateral breast cancer benefited from bilateral risk-reducing mastectomy and risk-reducing salpingo-oophorectomy. The impact of risk-reducing salpingo-oophorectomy in patients with luminal breast cancer in stage I/II increased with age. Most older patients with the *BRCA1* and *BRCA2* pathogenic variants in exons 12-24/25 with luminal breast cancer may gain a similar survival benefit from other risk-reducing strategies or surveillance.

**Conclusions:**

Our study showed that it is mandatory to consider the complex interplay between the types of *BRCA1* and *BRCA2* pathogenic variants, age at primary breast cancer diagnosis, breast cancer subtype and stage, and received systemic treatment. As no prospective study results are available at the moment, our simulation model, which will integrate a decision support system in the near future, could facilitate the conversation between the health care provider and patient and help to weigh all the options for risk-reducing strategies leading to a more balanced decision.

## Introduction

Breast cancer is the most common cancer and the leading cause of cancer mortality among women in economically developed and developing countries [1]. In unselected patients with breast cancer aged 35-64 years, pathogenic variants of *BRCA1* and *BRCA2* were detected in 2.4% and 2.3%, respectively [2]. In approximately 63% of patients with breast cancer related to the *pathogenic variant BRCA1* or *BRCA2*, genetic testing was performed after surgery of the primary cancer, with 62% of these patients also undergoing a primary breast-conserving treatment (BCT) [3].

Patients with breast cancer with *BRCA1* or *BRCA2* who underwent BCT have a significantly higher risk of a second primary ipsilateral breast event that is almost exclusively a new primary breast cancer rather than a true recurrence [4-6]. In addition, 27% of carriers of the *BRCA1* pathogenic variant and 19% of carriers of the *BRCA2* pathogenic variant will develop a second primary contralateral breast cancer within 10 years after the first primary breast cancer diagnosis [7].

Current guidelines describe different cancer risk management strategies: enhanced breast cancer screening, risk-reducing bilateral mastectomy (RRBM), risk-reducing salpingo-oophorectomy (RRSO), and chemoprevention [8]. Annual breast cancer screening with mammography and magnetic resonance imaging allows one to detect breast cancer at an early stage [9], although it cannot be prevented. In carriers of the *BRCA1* and *BRCA2* pathogenic variants, prophylactic mastectomy reduces the risk of subsequent breast cancer by approximately 90% [10]. However, the prophylactic mastectomy procedure could also have a potentially damaging effect on the patient’s body image and sexual well-being [11-14]. Risk-reducing bilateral salpingo-oophorectomy (RRBSO) can be offered to carriers of the *BRCA1* and *BRCA2* pathogenic variants who are more than 35 years old or who have completed childbearing [8]. RRBSO reduces the risk of ovarian cancer by approximately 80%. However, the impact of RRBSO on second primary breast cancer risk remains uncertain and research findings are inconsistent [15-19]. RRBSO may also increase patients’ risk of osteopenia, osteoporosis, cardiovascular disease, and may negatively impact cognitive function and quality of life [20]. Therefore, only 70% of carriers of the *BRCA1* and *BRCA2* pathogenic variants elect for RRBSO [21].

Previous studies have revealed that different factors, such as the patient’s age at first breast cancer diagnosis, the type of pathogenic variant (ie, *BRCA1* or *BRCA2*), first breast cancer subtype, adjuvant systemic treatment received, presence or absence of ovarian cancer, may influence the degree to which a particular patient will benefit from various prophylactic strategies [15,16,22-33]. Ultimately, the patient and her health care team, who have already faced the first breast cancer treatment, are confronted with complex decisions regarding the optimal prophylactic strategy for subsequent cancer risk management. In addition, there are no prospective trials comparing different cancer risk–reducing strategies in patients with breast cancer that tested positive for the *BRCA1* and *BRCA2* pathogenic variants who were treated with BCT at the first event.

The aim of this study is to develop a personalized risk management guideline for carriers of the pathogenic variants of *BRCA1* and *BRCA2* who underwent BCT for unilateral early-stage breast cancer taking into account the patient characteristics and tumor prognostic parameters as well as systemic treatment received.

## Methods

### Study Design: Network Model and Strategies

We have developed a temporal Bayesian network model to estimate the expected overall survival of a hypothetical cohort of *BRCA1* and *BRCA2* carriers diagnosed with stage I-II unilateral breast cancer and treated with BCT who underwent subsequent second primary cancer prevention strategies. A Bayesian network is a directed acyclic graph that represents the joint distribution of a single set of variables. Each variable is represented by a node in the graph and is dependent on the set of variables represented by its ascendant nodes. This dependence is represented by a conditional probability table that describes the probability distribution of each variable given its ascendant variables. Temporal Bayesian networks are a special type of model where each (temporal) variable is expressed in multiple linked nodes to represent events in different moments in time; for example, a 2-year model for the event “ovarian cancer” (OC) could be defined with 2 linked nodes: “OC-y1” and “OC-y2,” where OC-y2 is certain if OC-y1 is true, and P(OC-y2) is given by the yearly risk of OC if OC-y1 is false, adjusted for all their ascendant nodes.

All risk estimates were converted into yearly estimates by conditional probabilities, depending on the original metric published in the literature with needed conversions (eg, risk for years between 5 and 10 used 10-year estimates converted to actual follow-up) [34,35]. If incidence estimates were given for a certain follow-up time (eg, lifetime ovarian cancer risk), the probability for each year “i” is computed as 1 – exp(–rate[p,o] × c), where “p” is the original risk for occurrence within “o” years, and rate(p,o) = –ln(1 – p)/o.

If hazard ratios were given, survival for each group was computed as ref ^ hr, where “ref” is the expected survival for the reference group.

The simulation was run for a yearly follow-up of 40 years after diagnosis, yielding a temporal Bayesian network with 40 nodes per temporal variable (eg, ipsilateral recurrence). We predicted the overall survival following different prevention strategies for 144 cohorts (Multimedia Appendix 1) of women defined by the location of the *BRCA1* and *BRCA2* pathogenic variants (*BRCA1*: exons 1-10, exon 11, and exons 12-24; *BRCA2*: exons 1-10; exon 11; exons 12-25), age at primary breast cancer diagnosis (<40 years old, 40-50 years old, and >50 years old), breast cancer subtype (luminal-like and triple negative [TN]), stage of primary breast cancer (stage I and II), and presence or absence of adjuvant chemotherapy.

Data on 1 million simulations were generated. Each subgroup combination had around 6900 patients simulated across the 9 different intervention policies: (1) surveillance; (2) contralateral risk–reducing mastectomy; (3) RRBM; (4) contralateral risk–reducing mastectomy and RRBSO; (5) RRBM-RRBSO; (6) 5-year tamoxifen therapy; (7) contralateral risk–reducing mastectomy and 5-year tamoxifen therapy; (8) RRBSO; and (9) RRBM and 5 years’ tamoxifen therapy. As a result, around 770 patients were distributed to each subgroup × policy combination. All these intervention policies were considered with or without adjuvant chemotherapy, totaling 18 different policies.

For each patient, the first temporal node to be activated was identified, and survival computed for each patient. The overall survival of patients assigned for each subgroup × policy combination was plotted as Kaplan-Meier curves for 40-year follow-up and compared by the log-rank test. Hazard ratios for each subgroup were computed according to the proportional hazard Cox regression. However, given the simulation nature of the data, it was not possible to analyze any *P* value or CI estimates.

A temporal Bayesian network model was constructed using R (R Foundation for Statistical Computing) statistical software packages ‘bnlearn’ [36] and ‘gRain’ [37], assigning the overall survival associated for each strategy.

Key decision variables used in baseline and sensitivity analyses were obtained from peer-reviewed English language literature published in PubMed and from publicly available databases (Multimedia Appendix 2; also see [5,19,22,23,30,31,34,38-45]). We obtained the age-specific risk of death from other causes from Colzani et al [46]. TN breast cancer was defined as estrogen receptor (ER) <10%, progesterone receptor (PR) <10%, human epidermal growth factor receptor 2 (HER2–) 0 or 1+. Luminal phenotype breast cancer was defined as ER+, PR+, and HER2– 0 or 1+.

### First Primary Breast Cancer

Both *BRCA1* and *BRCA2* pathogenic variant–related tumors rarely showed evidence of HER2 amplification or expression [47-54].

Therefore, we did not include carriers of the *BRCA1* and *BRCA2* pathogenic variants with HER2-positive breast cancer subtypes in our hypothetical cohort. We derived that the cumulative incidence of first primary TN breast cancer in *BRCA1* and *BRCA2* carriers is 69% and 15%, respectively [55], and that other breast cancers are of luminal phenotype by immunohistochemistry. According to a recently published meta-analysis of 66 studies, there is no clear evidence supporting the different prognosis for patients with breast cancer with *BRCA1* and *BRCA2* pathogenic variants compared with sporadic cases [48,50].

Therefore, we used stage-specific and breast cancer subtype–specific mortality rates adjusted for age, race/ethnicity, and socioeconomic status reported in the population-based study by *Parise and* Caqqiano [38], where 143,333 female primary first invasive breast cancer cases were included. We assumed that 52.3% of breast cancer cases were diagnosed in stage I, 43.1% in stage II, and 4% in stage III [5].

### BRCA1/BRCA2 Pathogenic Variant Genotype-Phenotype Correlation and Ovarian Cancer

In the study published by Bayraktar et al [22], patients with exon 20 *BRCA1* pathogenic variant and patients with exons 12-25 *BRCA2* pathogenic variant had a higher risk of developing both breast and ovarian cancer compared with patients with other exon mutations. We assumed ovarian cancer lifetime incidence in patients with unilateral primary breast cancer as breast and ovarian cancer prevalence by the pathogenic variant of the *BRCA1* and *BRCA2* combined exon group [22,56]. The risk of ovarian cancer was modeled assuming an expected lifetime of 80 years. For each age stratum, we computed the initial risk of ovarian cancer for patients aged 35, 45, and 65 years, respectively.

In patients with *BRCA1-* and *BRCA2*-related breast cancer, adjuvant tamoxifen and chemotherapy showed no impact on the risk reduction of subsequent ovarian cancer [28]. Therefore, we did not evaluate the impact of systemic treatment on ovarian cancer rates in our simulation model.

We used the distribution of stage at diagnosis of ovarian cancer and 10-year survival rates for ovarian cancer reported by Benedet et al [39].

A recently published study [28] showed no long-term survival benefit in patients with *BRCA1-* and *BRCA2*-related ovarian cancer compared with patients with sporadic ovarian cancer.

### Second Primary Contralateral Breast Cancer

For contralateral breast cancer we assumed the same breast cancer distribution as for the first primary breast cancer [38]. We summarized the stage-specific and breast cancer subtype–specific mortality rate of the first primary breast cancer with the stage-specific and breast cancer subtype–specific mortality rate of the second primary contralateral breast cancer.

Multiple studies showed that a younger age at the onset of first breast cancer is associated with a higher contralateral breast cancer risk [23-26]. We assumed the age at the onset of first breast cancer based on the cumulative, contralateral breast cancer risk estimates proposed by Graeser et al [24]. We used the lifetime breast cancer–specific mortality for ductal carcinoma in situ of 3.3% reported by Narod et al [40]. We assumed the breast cancer–specific mortality of 100% for patients with metastatic invasive contralateral breast cancer [41]. We assumed that RRBSO does not reduce the risk of contralateral breast cancer in carriers of the *BRCA1* and *BRCA2* pathogenic variants [57].

### Ipsilateral Breast Cancer

According to the previously published studies, carriers of the *BRCA1* and *BRCA2* pathogenic variants who underwent BCT for the first primary breast cancer have an increased risk of ipsilateral breast events compared with carriers of the *BRCA1* and *BRCA2* pathogenic variants who underwent a mastectomy [4-6]. We assumed the local failure cumulative incidence reported by Pierce et al [6]. Previous studies demonstrated that RRBSO and adjuvant chemotherapy decrease the risk of an ipsilateral breast event. We used hazard ratios published by Valachis et al [31]. In this meta-analysis, RRBSO decreased the risk of an ipsilateral breast event by 58% and adjuvant chemotherapy decreased the risk of an ipsilateral breast event by 49%. There was no evidence to support the protective effect of tamoxifen against ipsilateral breast cancer [31]. Despite the high rate of ipsilateral events in carriers of the pathogenic variants of *BRCA1* and *BRCA2* who underwent BCT, there was no statistically significant impact on distant recurrence and disease-specific survival [5]. These findings could be explained by the limited sample size in the study’s cohorts and detection of ipsilateral events in the early stage due to the close surveillance of carriers of the *BRCA1* and *BRCA2* pathogenic variants [5]. The mean time to ipsilateral events was approximately 7 years. This fact conveys the impression that most of these events were new primary breast cancers [4-6]. Therefore, for patients who developed ipsilateral breast cancer we assumed the same stage-specific and breast cancer subtype–specific mortality rates as for the first primary breast cancer [38]. We summarized the stage-specific and breast cancer subtype–specific mortality rate of the first primary breast cancer with the stage-specific and breast cancer subtype–specific mortality rate of an ipsilateral breast event. We assumed that the stage distribution and breast cancer mortality in patients with a new second primary breast cancer are the same as those for the contralateral breast cancer [38,40-42].

### Prevention Strategies

#### Prophylactic Oophorectomy

We considered an 80% risk reduction of ovarian cancer in carriers of the *BRCA1* pathogenic variant and 79% risk reduction in carriers of the *BRCA2* pathogenic variant [15].

In our hypothetical cohort, patients underwent an RRBSO within 5 years after the first primary breast cancer diagnosis. At the moment, there is no clear evidence suggesting that hormone replacement therapy does not offset the second primary breast cancer risk induced by RRBSO in *BRCA1 and BRCA2* carriers [18,58-60]. Therefore, we assumed that none of the patients in our cohort received hormone replacement therapy. RRBSO is associated with a higher risk of noncancer-related death. Thus, in the simulation, we applied increased noncancer mortality for patients who underwent RRBSO before the age of 45 and had an onset of first primary breast cancer before the age of 40 [43]. The mortality risk following RRBSO was assumed to be the same as that following a mastectomy.

#### Contralateral Prophylactic Mastectomy

We assumed contralateral risk-reducing bilateral mastectomy–adjusted risk reduction of primary mastectomy [44]. Patients who underwent contralateral mastectomy have a statistically significantly better survival rate compared with patients who underwent surveillance [42]. The survival benefit was even more pronounced in patients with primary breast cancer onset under 40 years of age, with no TN breast subtype, and not treated with chemotherapy [42].

#### Ipsilateral Prophylactic Mastectomy

We assumed the local failure cumulative incidence in patients who underwent a mastectomy, as reported by Pierce et al [5]. According to Pierce et al [5], or carriers of *BRCA1* and *BRCA2*, who underwent mastectomy and received chemotherapy and RRBSO, the status of the pathogenic variants of *BRCA1* and *BRCA2* had no impact on local failure rate [5]. We assumed that an isolated locoregional recurrence after mastectomy has no impact on the survival of patients with breast cancer [61].

#### Tamoxifen

We used data from the combined International Retrospective-Prospective Carriers of the Pathogenic Variants of the *BRCA1* and *BRCA2* cohort study, in which 1583 carriers of the pathogenic variant of *BRCA1* and 881 carriers of the pathogenic variant of *BRCA2* with unilateral breast cancer were included [30]. We assumed that 5-year tamoxifen administration reduces 15-year mortality by 30% in patients with luminal-like primary breast cancer [62].

We assumed that tamoxifen reduces the age-specific ER status–adjusted risk of second primary contralateral breast cancer by 56% in carriers of the *BRCA1* pathogenic variant and by 67% in carriers of the *BRCA2* pathogenic variant [30]. We assumed that chemotherapy has no additional protective effect on the contralateral breast cancer development in carriers of *BRCA1* and *BRCA2* who received tamoxifen [30]. We assumed that the use of tamoxifen for 5 years has no impact on mortality due to cardiovascular or thromboembolic disease [63].

### Ethical Considerations

Our simulation model of a hypothetical cohort was based on previously published data, and therefore did not require a submission to a research ethics committee.

## Results

### Effectiveness of Secondary Prevention Strategies

The predicted 40-year overall survival rate for carriers of *BRCA1* and *BRCA2* variants after unilateral BCT who received secondary cancer prevention strategies or surveillance is presented in Multimedia Appendix 3.

**Figure 1:**
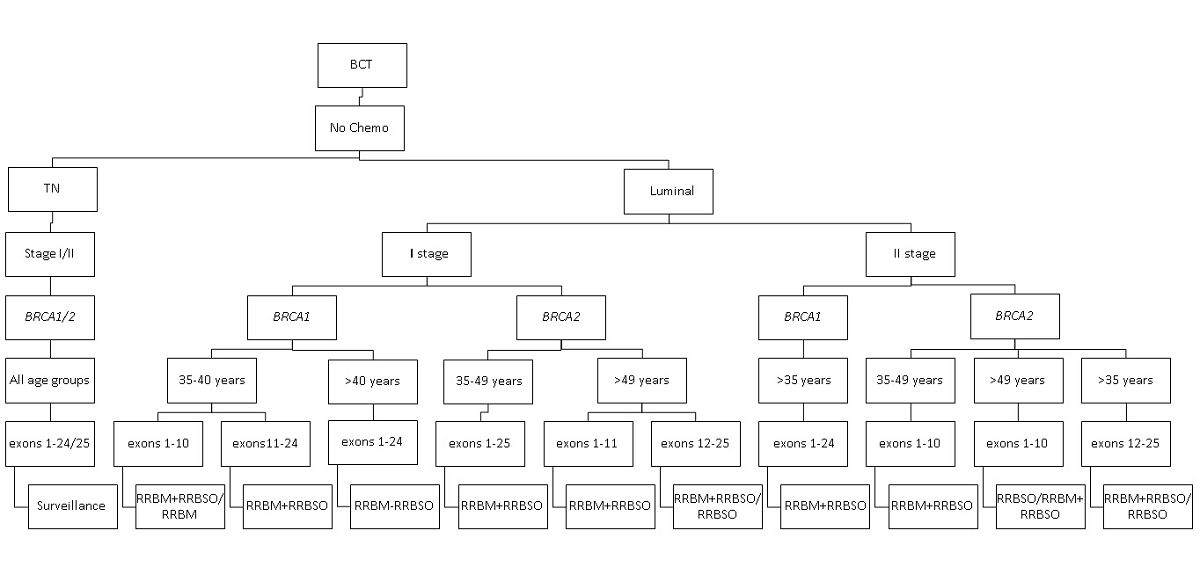
The most effective secondary prophylactic strategies in *BRCA1* and *BRCA2* carriers who received no adjuvant chemotherapy. BCT: breast-conserving treatment; RRBM: risk-reducing bilateral mastectomy; RRBSO: risk-reducing bilateral salpingo-oophorectomy; TN: triple negative.

The impact of secondary prevention strategies on the survival of carriers of the *BRCA1* and *BRCA2* pathogenic variants with luminal breast cancer who received no adjuvant chemotherapy is presented in Figure 1.

Absence of adjuvant chemotherapy was the most powerful factor that was linked to a dramatic decline in survival for patients with breast cancer with *BRCA1* and *BRCA2* mutation. There was a *negligible increase in survival* for carriers of the pathogenic variants *BRCA1* and *BRCA2* with TN breast cancer and who received any secondary prevention strategy compared with surveillance.

Most carriers of *BRCA1* and *BRCA2* with luminal breast cancer in stage I benefited from RRBM-RRBSO. However, patients with breast cancer aged less than 40 years with the *BRCA1* pathogenic variant in exons 1-10 in stage I who underwent RRBM + RRBSO had an almost similar impact on survival compared with those who underwent RRBM alone. By contrast, patients with breast cancer aged over 49 years with the *BRCA2* pathogenic variant in exons 12-25 in stage I who underwent RRBM + RRBSO had an almost similar impact on survival compared with those who underwent RRBSO alone.

In patients with breast cancer with the *BRCA1* and *BRCA2* pathogenic variants with luminal breast cancer in stage II, RRBM + RRBSO had a very modest impact on survival compared with surveillance. Interestingly, RRBM-RRBSO or only RRBSO was the most effective prevention strategy in patients with luminal breast cancer aged over 35 years with the *BRCA2* pathogenic variant in exons 12-25 in stage II and in patients aged over 49 years with the *BRCA2* pathogenic variant in exons 1-10 in stage II. By contrast, there was a negligible increase in survival among carriers of *BRCA1* and *BRCA2* aged over 35 years with luminal breast cancer in exon 11 who underwent any secondary prevention strategy compared with surveillance.

Interestingly, we noted that the impact of RRBSO in patients with the *BRCA1* and *BRCA2* pathogenic variants with luminal breast cancer in stage I/II increased with the age.

**Figure 2:**
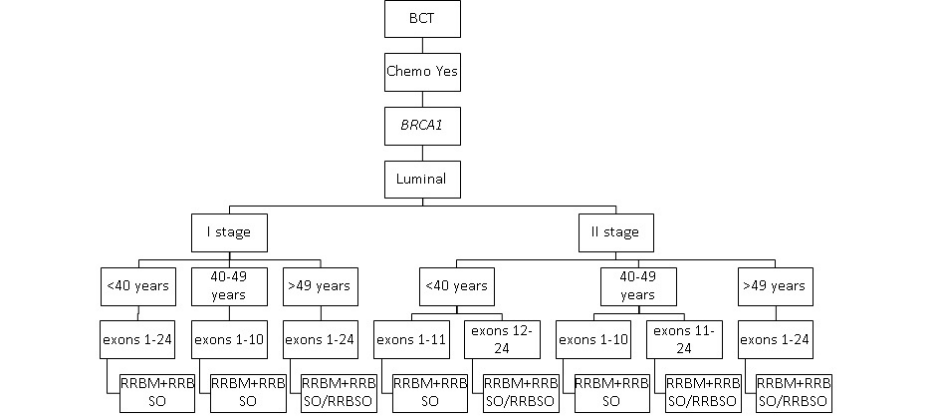
The most effective secondary prophylactic strategies in *BRCA1* carriers with luminal breast cancer who received adjuvant chemotherapy. BCT: breast-conserving treatment; RRBM: risk-reducing bilateral mastectomy; RRBSO: risk-reducing bilateral salpingo-oophorectomy.

**Figure 3:**
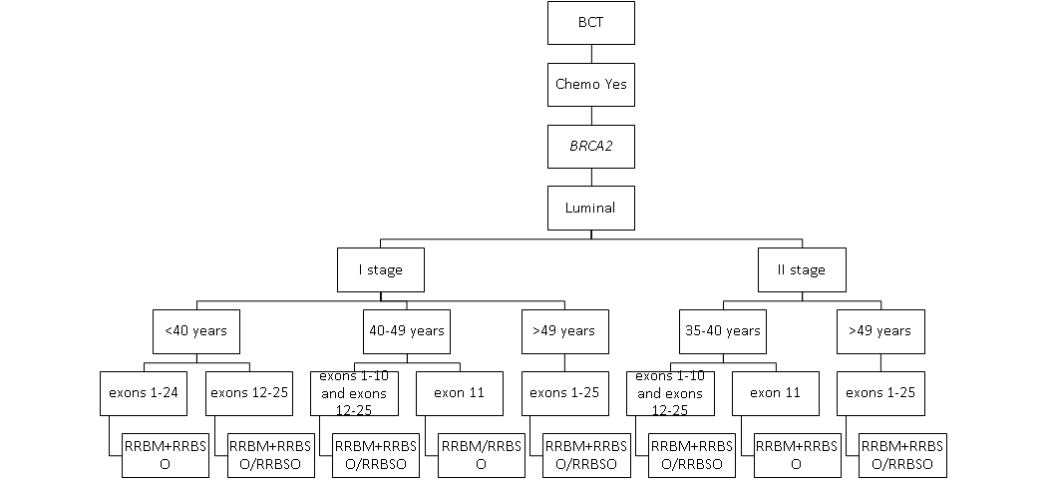
The most effective secondary prophylactic strategies in *BRCA2* carriers with luminal breast cancer who received adjuvant chemotherapy. BCT: breast-conserving treatment; RRBM: risk-reducing bilateral mastectomy; RRBSO: risk-reducing bilateral salpingo-oophorectomy.

The impact on the survival of secondary risk–reducing strategies among carriers of *BRCA1* and *BRCA2* variants with luminal breast cancer after adjuvant chemotherapy is presented in Figures 2 and 3.

RRBM-RRBSO was the most effective risk-reducing strategy in patients with luminal breast cancer who received adjuvant chemotherapy. The protective role of RRBSO in patients with luminal breast cancer increased with their age at diagnosis, stage of the disease, and was impacted by the type of pathogenic variant (*BRCA1* or *BRCA2*).

**Figure 4:**
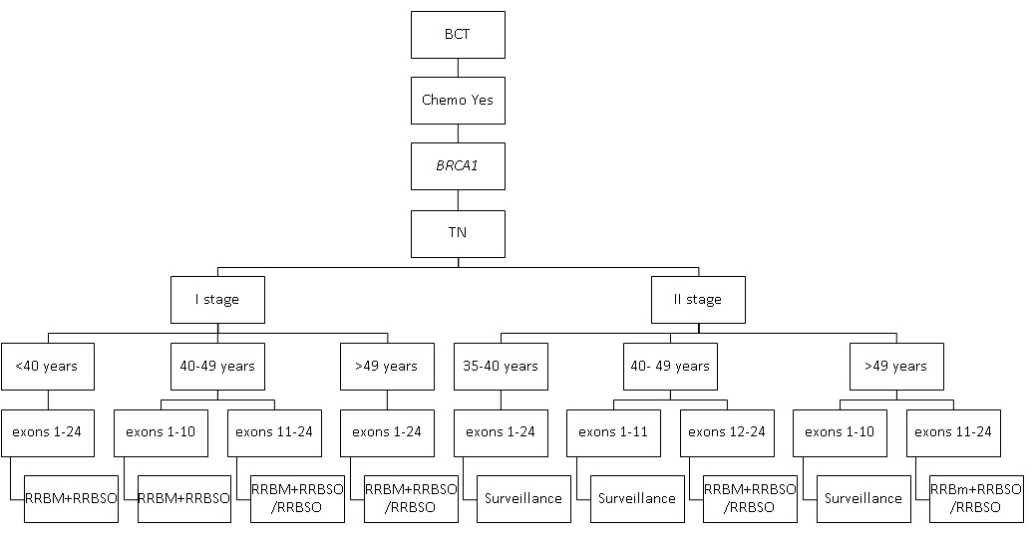
The most effective secondary prophylactic strategies in *BRCA1* carriers with TN breast cancer after adjuvant chemotherapy. BCT: breast-conserving treatment; RRBM: risk-reducing bilateral mastectomy; RRBSO: risk-reducing bilateral salpingo-oophorectomy; TN: triple negative.

**Figure 5:**
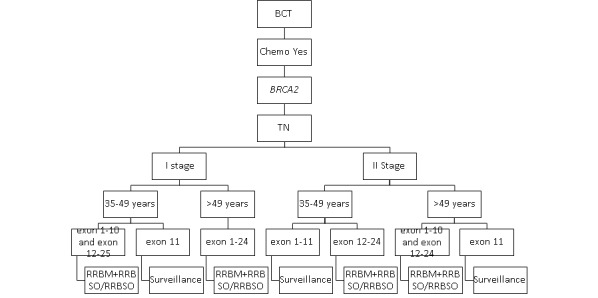
The most effective secondary prophylactic strategies in *BRCA2* carriers with TN breast cancer after adjuvant chemotherapy. BCT: breast-conserving treatment; RRBM: risk-reducing bilateral mastectomy; RRBSO: risk-reducing bilateral salpingo-oophorectomy; TN: triple negative.

The impact on survival of secondary risk–reducing strategies in carriers of the pathogenic variants of *BRCA1* and *BRCA2* with TN breast cancer after adjuvant chemotherapy is shown in Figures 4 and 5.

The potential survival benefit from any risk-reducing strategy was modest in patients with TN breast cancer when compared with patients with luminal breast cancer. However, most carriers of *BRCA1* and *BRCA2* with TN breast cancer in stage I benefited from RRBM-RRBSO or just RRBSO.

RRBM-RRBSO was only the most effective risk-reducing strategy in patients with TN breast cancer under 40 years in stage I with the *BRCA1* pathogenic variant and in patients aged 40-49 years with the *BRCA1* pathogenic variant in exons 1-10. Further, patients with TN breast cancer aged 35-49 years in stage I with the *BRCA2* pathogenic variant in exon 11 had a very modest benefit from RRBM-RRBSO.

There was a negligible increase in survival in almost all carriers of the pathogenic variants of *BRCA1* and *BRCA2* with TN breast cancer in stage II who underwent any secondary risk–reducing strategy compared with surveillance.

However, RRBM-RRBSO or just RRBSO was the most effective risk-reducing strategy in patients with TN breast cancer aged over 40 years with the *BRCA1* pathogenic variant in exons 12-24 in stage II and in patients aged over 35 years with the *BRCA2* pathogenic variant in exons 12-24 in stage II. The impact of RRBSO in patients with *BRCA1* and *BRCA2* mutation with TN breast cancer in stage I/II increased with age.

### Sensitivity Analysis

There were difficulties in validating our results because of the lack of previously published studies with similar subgroups of patients that match all the detailed clinical and treatment variables.

**Figure 6:**
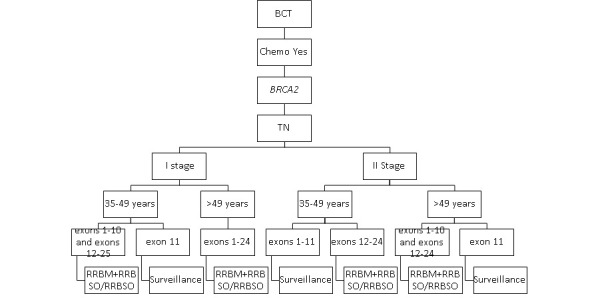
Kaplan-Meier survival plots for 15 years' simulation performed using TN subgroup cohort definitions from the largest published prospective POSH study. TN: triple negative.

Validation of our method was performed using TN subgroup cohort definitions from the largest published prospective study (POSH) [64].Settings of the simulation were as follows: (1) from the 1 million patients in our simulation, we only considered those who were younger than 40 years at TN breast cancer diagnosis; (2) the distribution of *BRCA1* and *BRCA2* carriers with TN breast cancer was performed according to Copson et al [64] (*BRCA1*: 123/136, *BRCA2*: 13/136); (3) the distribution of adjuvant chemotherapy in the TN *BRCA*-positive group was performed according to the study by Copson et al [64] (probability = 117/136); (4) the distribution of exon was uniform; (5) primary breast cancer stage distribution was used from the literature, considering the fact that all the patients were either in stage I or II (about 61.2% in stage I and nearly 38.8% in stage II). The simulation was run for 15 years after diagnosis with network steps of 2.5 years across the 9 different intervention policies. The overall survival of patients was plotted as a Kaplan-Meier curve (Figure 6) and compared with CIs from Copson et al [64] for TN *BRCA1-* and *BRCA2*-positive cases.

In our simulation, the overall survival was similar to the results in the POSH study. Similarly, in our simulation model patients with TN breast cancer aged under 40 years with the *BRCA1* and *BRCA2* pathogenic variants in stage I/II who received adjuvant chemotherapy had no survival benefit from RRBM. RRBM-RRBSO was the most effective risk-reducing strategy in these patients.

## Discussion

### Principal Findings

To our knowledge, this is the first study to simulate the expected overall survival and determine the most effective personalized management strategies for carriers *BRCA1* and *BRCA2* variants who underwent BCT for unilateral early-stage breast cancer taking into account the type of the pathogenic variant (*BRCA1* or *BRCA2*), age at primary breast cancer diagnosis, breast cancer subtype, stage, and received systemic treatment. Absence of adjuvant chemotherapy was the most powerful factor that was linked to a dramatic decline in survival for patients with breast cancer with the pathogenic variants of *BRCA1* and *BRCA2*. There was a negligible decline in mortality among carriers of *BRCA1* and *BRCA2* with TN breast cancer who received no chemotherapy and underwent any secondary risk–reducing strategy compared with surveillance. The potential survival benefit from any risk-reducing strategy was more modest in patients with TN breast cancer who received chemotherapy compared with patients with luminal breast cancer. However, most carriers of *BRCA1* and *BRCA2* with TN breast cancer in stage I benefited from RRBM-RRBSO or just RRBSO. Most carriers of the pathogenic variant of *BRCA1* or *BRCA2* with luminal breast cancer in stage I-II (unilateral breast cancer) benefited from RRBM-RRBSO. The impact of RRBSO in patients with the *BRCA1* and *BRCA2* pathogenic variants with luminal breast cancer in stage I/II increased with age. Most older patients with the *BRCA1* and *BRCA2* pathogenic variants in exons 12-24/25 with luminal breast cancer may gain a similar survival benefit from other risk-reducing strategies or surveillance.

### Comparison With Prior Work

To date, only Schrag et al [65] have addressed secondary cancer risk–reducing strategies in *BRCA1* and *BRCA2* carriers who underwent BCT for unilateral breast cancer, using decision-analytic models. They calculated life-expectancy gains for different age groups; lymph node positive or negative status; and low, moderate, or high penetrance of the *BRCA1* and *BRCA2* pathogenic variants using a Markov model that incorporated 8 prevention strategies [65]. By contrast, we developed a temporal Bayesian network model with a total of 1 million simulated patients across 9 different intervention policies. Our study is an advancement over previous ones due to the incorporation of variables from up-to-date peer-reviewed studies considering the type of pathogenic variant (*BRCA1* or *BRCA2*), age at primary breast cancer diagnosis, breast cancer subtype and stage, status of systemic treatment received.

Our study showed that most *BRCA1* and *BRCA2* carriers benefited from RRBM + RRBSO. However, in patients with TN breast cancer who received no adjuvant chemotherapy, the impact of secondary risk–reducing strategies on overall mortality was reduced as a result of the higher risk of dying from primary breast cancer rather than from subsequent primary secondary cancer. Nevertheless, younger patients with limited disease who received adjuvant chemotherapy gained more benefit from aggressive surgical management (RRBM + RRBSO). By contrast, in older patients with more advanced disease the protective role of bilateral risk-reducing salpingo-oophorectomy was comparable to RRBM + RRBSO and was more pronounced in exons 12-24 of the *BRCA1* pathogenic variant and in exons 12-25 of the *BRCA2* pathogenic variant. In our study, we assumed that RRBSO does not reduce the risk of contralateral breast cancer in carriers of the *BRCA1* and *BRCA2* pathogenic variants. Recent studies showed no impact of RRBSO on primary and second primary breast cancer risk reduction [19,66]. As the risk of contralateral and ipsilateral breast cancer decreases with age [5,24] and the risk of ovarian cancer increases with age [67], patients with a higher probability of ovarian cancer gain a stronger protective effect from RRBSO and a low additional protective effect from RRBM.

In our model, we assumed breast cancer subtype–specific, population-based mortality and the general breast cancer population–based impact of adjuvant chemotherapy on outcomes. According to the prospective study by Clifton et al [68], for patients with TN breast cancer with the *BRCA1 and BRCA2* pathogenic variants in stage I/II, there was no difference in overall survival for those who received neoadjuvant chemotherapy compared with those who received adjuvant chemotherapy [68].

However, a growing body of evidence indicates that *BRCA1* and *BRCA2* TN as well as luminal cancers are more chemosensitive and achieve higher pathologic complete response (pCR) rates compared with breast cancer without the *BRCA1* or *BRCA2* pathogenic variant [69,70]. Paradoxically, in *BRCA1* and *BRCA2* breast cancer tumors it seems that pCR does not serve as a surrogate marker of better clinical outcome and a higher pCR does not translate into improved disease-free and overall survival [71,72]. Therefore, we did not include a neoadjuvant chemotherapy treatment strategy in our model.

In the systematic review published by Davey et al [73], there was no difference in 15-year mortality between BCT and mastectomy in *BRCA1* and *BRCA2* carriers with breast cancer. Most patients in both BCT and mastectomy groups had T1/2 tumors; approximately 60% of patients had ER–, N+ disease, or underwent adjuvant chemotherapy; and approximately 50% of patients underwent RRBSO [73]. In our study, for most patients with TN breast cancer in stage II who received chemotherapy, and all patients with TN breast cancer in stage I/II who did not receive chemotherapy, there was no difference in survival between any secondary prevention strategy and surveillance.

In a study published by Wan et al [74], where 8396 consecutive patients with breast cancer after surgery were included, no survival benefit was shown in carriers with *BRCA1* and *BRCA2* who underwent BCT compared with those who underwent mastectomy with or without radiotherapy. However, only 73 carriers with the *BRCA1* pathogenic variant and 106 with the *BRCA2* pathogenic variant who underwent BCT, and 104 carriers with the *BRCA1* pathogenic variant and 198 with the *BRCA2* pathogenic variant who underwent mastectomy were included in the study, with a relatively short follow-up period of 7.5 years. As the study is retrospective with a relatively small patient number in the subgroups, caution should be exercised while estimating the study’s results and thus, further prospective research with a larger study population is needed.

### Limitations

The main limitation of our study is that it is a computer simulation and it can misrepresent reality. Nevertheless, our model could prove to be a valuable decision support tool and we plan to validate our model on the target patient population. Risk modifiers assume adjusted risk estimates, and therefore they are additive, and some evidence is thin and dated in some of the included estimates. We assumed independence of risk factors where it was not possible to model any interaction, and this is a limitation. Nonetheless, the final validation shows concurrent results, supporting our model.

### Conclusions

At present, no personalized guidelines are available for the prophylactic management of second primary breast cancer in patients with the *BRCA1* and *BRCA2* pathogenic variants with unilateral breast cancer who underwent BCT as a primary procedure. Our study showed that it is mandatory to consider the complex interplay between the type of *BRCA1* and *BRCA2* pathogenic variants, age at primary breast cancer diagnosis, breast cancer subtype and stage, and systemic treatment received. As no prospective study results are available, our simulation model could facilitate the conversation between the health care provider and patient and help to weigh all the options for risk-reducing strategies, thus leading to a more balanced decision. However, we plan to expand and update our model by including more variables from new evidence-based research and develop a computer-based clinical decision tool.

## Appendix

Multimedia Appendix 1 Overall survival following different prevention strategies for 144 cohorts.

Multimedia Appendix 2 Key decision variables used in baseline and sensitivity analyses.

Multimedia Appendix 3 Secondary cancer prevention strategies in *BRCA1/2* mutation carriers.

## Abbreviations

BCT: breast-conserving treatment
ER: estrogen receptor
FIGO: International Federation of Gynaecology and Obstetrics
HER2: human epidermal growth factor receptor 2
pCR: pathologic complete response
PR: progesterone receptor
RRBM: risk-reducing bilateral mastectomy
RRBSO: risk-reducing bilateral salpingo-oophorectomy
TN: triple negative

## Availability of Data and Materials

The authors confirm that the data supporting the findings of this study are available within the article or its Multimedia Appendices.
