# Correction: Effectiveness of Secondary Risk–Reducing Strategies in Patients With Unilateral Breast Cancer With Pathogenic Variants of BRCA1 and BRCA2 Subjected to Breast-Conserving Surgery: Evidence-Based Simulation Study

**DOI:** 10.2196/45810

**Published:** 2023-03-16

**Authors:** Jelena Maksimenko, Pedro Pereira Rodrigues, Miki Nakazawa-Miklaševiča, David Pinto, Edvins Miklaševičs, Genadijs Trofimovičs, Jānis Gardovskis, Fatima Cardoso, Maria João Cardoso

**Affiliations:** 1 Institute of Oncology, Department of Surgery, Breast Unit Pauls Stradiņš Clinical University Hospital Riga Stradiņš University Riga Latvia; 2 Information and Health Decision Sciences of the Faculty of Medicine University of Porto Porto Portugal; 3 Institute of Oncology Riga Stradiņš University Riga Latvia; 4 Breast Cancer Unit Champalimaud Cancer Center Lisbon Portugal; 5 Faculty of Medicine Rīga Stradiņš University Riga Latvia; 6 Department of Surgery, Faculty of Medicine Pauls Stradins Clinical University Hospital Rīga Stradiņš University Riga Latvia

In “Effectiveness of Secondary Risk–Reducing Strategies in Patients With Unilateral Breast Cancer With Pathogenic Variants of BRCA1 and BRCA2 Subjected to Breast-Conserving Surgery: Evidence-Based Simulation Study” (JMIR Form Res 2022;6(12):e37144) the authors noted one error.

In the originally published article Figure 6 appeared as a duplicate of Figure 5.

Figure 6 has been corrected as follows:

**Figure 6:**
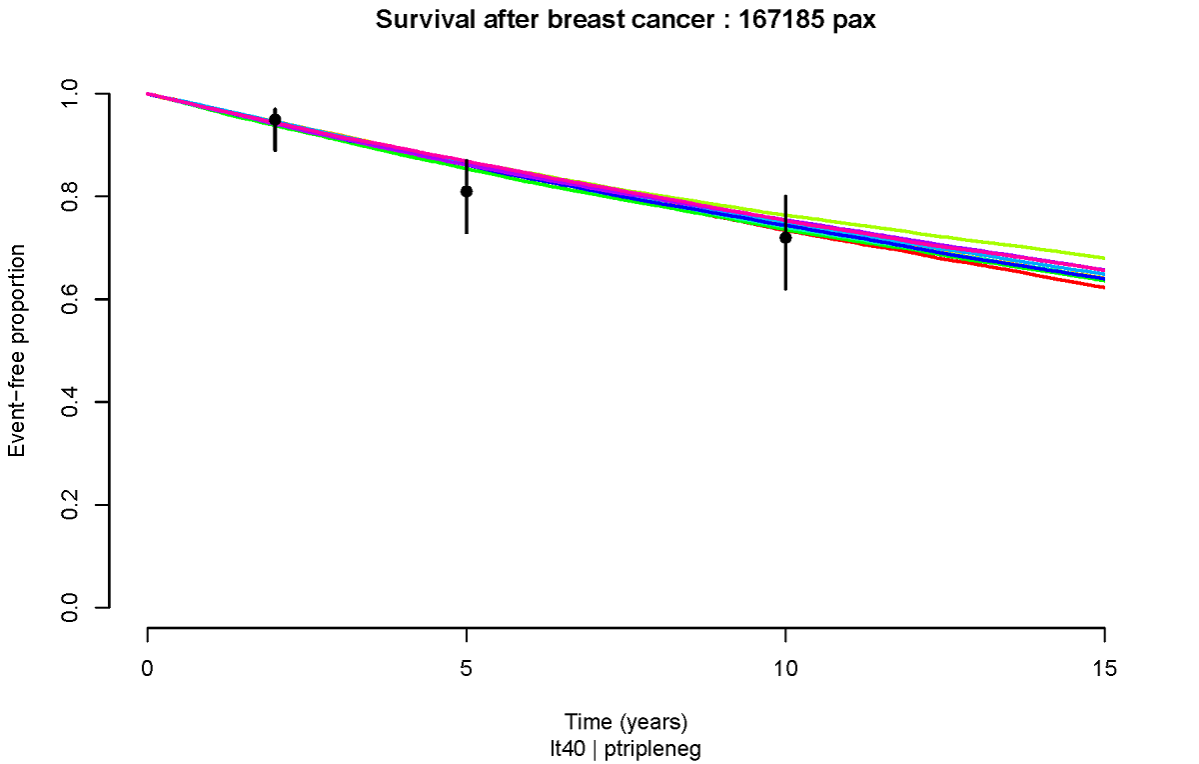
Kaplan-Meier survival plots for simulation for 15 years, performed using TN subgroup cohort definitions from the largest published prospective POSH study.

The correction version appeared in the online version of the paper on the JMIR Publications website on January 18, 2023. Because this was made after submission to full-text repositories, the corrected article has also been resubmitted to those repositories.

