# Supplementary material for: Effectiveness of Secondary Risk–Reducing Strategies in Patients With Unilateral Breast Cancer With Pathogenic Variants of BRCA1 and BRCA2 Subjected to Breast-Conserving Surgery: Evidence-Based Simulation Study
Source: JMIR Form Res. 2022 Dec 29;6(12):e37144. doi: 10.2196/37144 (PMC9837710; doi:10.2196/37144)
Supplement: Multimedia Appendix 1 [file formative_v6i12e37144_app1.pdf]

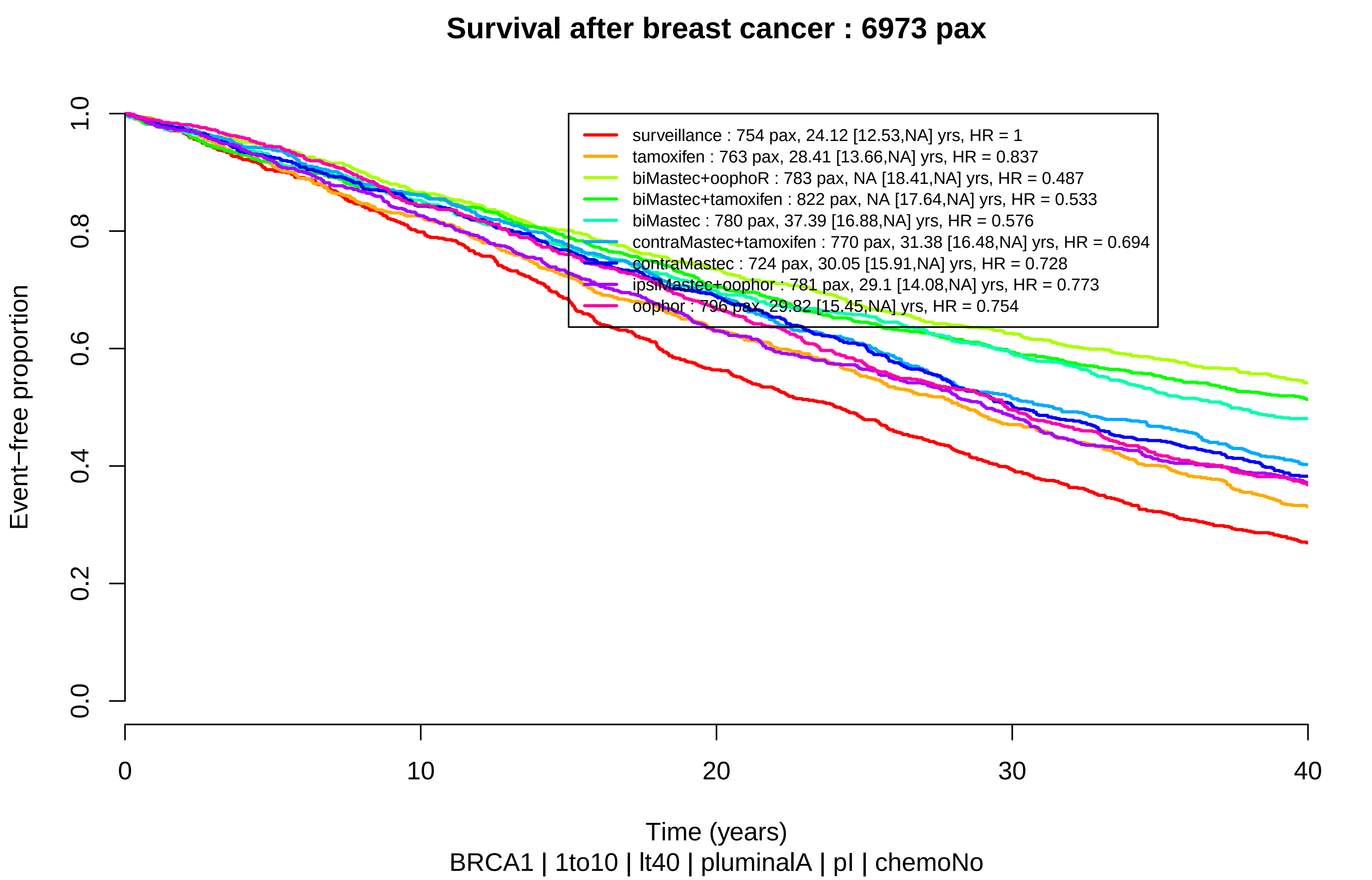

Survival after breast cancer : 6973 pax
surveillance : 754 pax, 24.12 [12.53,NA] yrs, HR = 1
tamoxifen : 763 pax, 28.41 [13.66,NA] yrs, HR = 0.837
biMastec+oophoR : 783 pax, NA [18.41,NA] yrs, HR = 0.487
biMastec+tamoxifen : 822 pax, NA [17.64,NA] yrs, HR = 0.533
biMastec : 780 pax, 37.39 [16.88,NA] yrs, HR = 0.576
contraMastec+tamoxifen : 770 pax, 31.38 [16.48,NA] yrs, HR = 0.694
contraMastec : 724 pax, 30.05 [15.91,NA] yrs, HR = 0.728
ipsiMastec+oophor : 781 pax, 29.1 [14.08,NA] yrs, HR = 0.773
oophor : 796 pax, 29.82 [15.45,NA] yrs, HR = 0.754
Event−free proportion
0.0
0.2
0.4
0.6
0.8
1.0
0
10
20
30
40
Time (years)
BRCA1 | 1to10 | lt40 | pluminalA | pI | chemoNo

Survival after breast cancer : 6897 pax

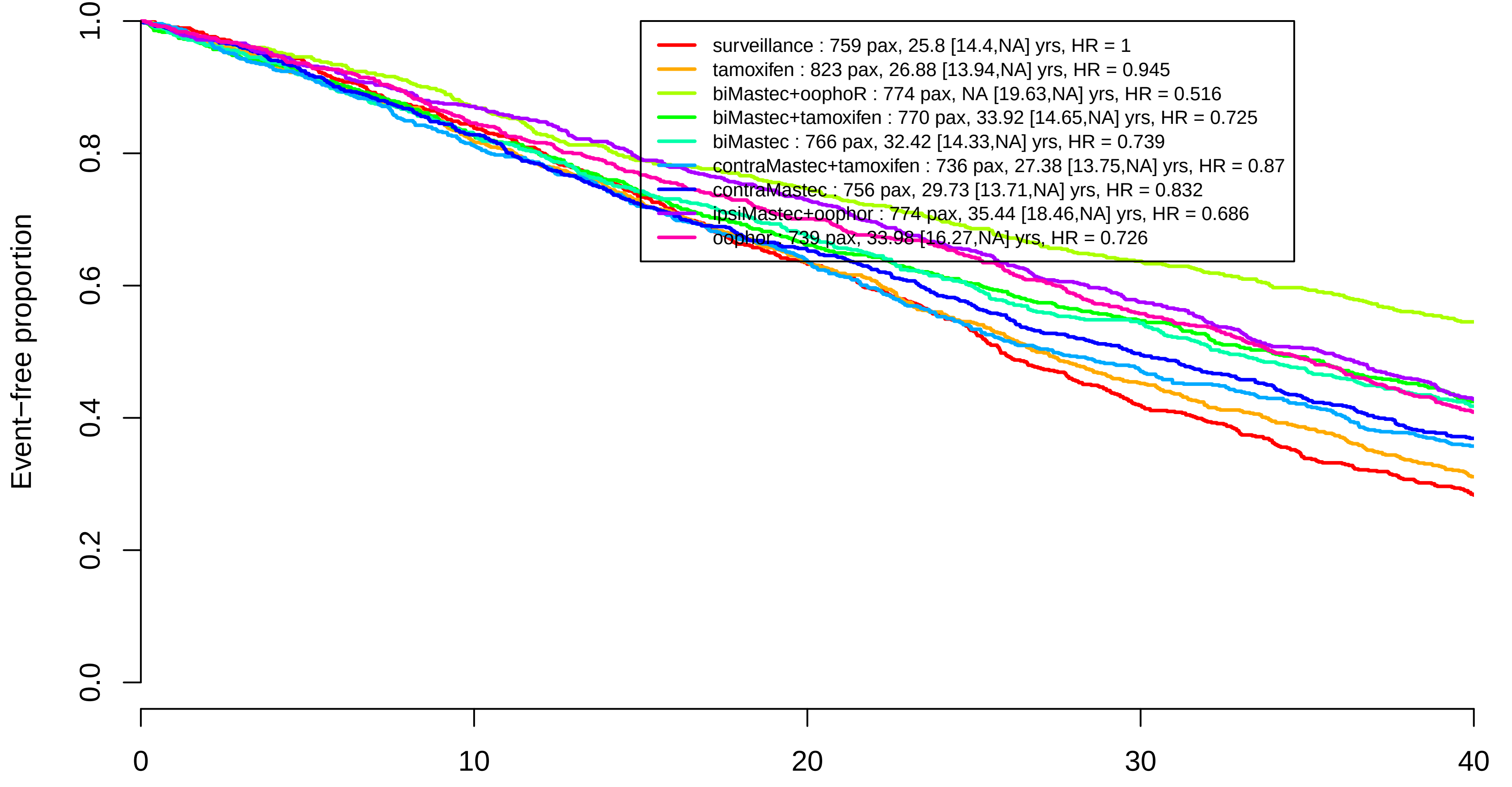

BRCA2 | 1to10 | lt40 | pluminalA | pI | chemoNo

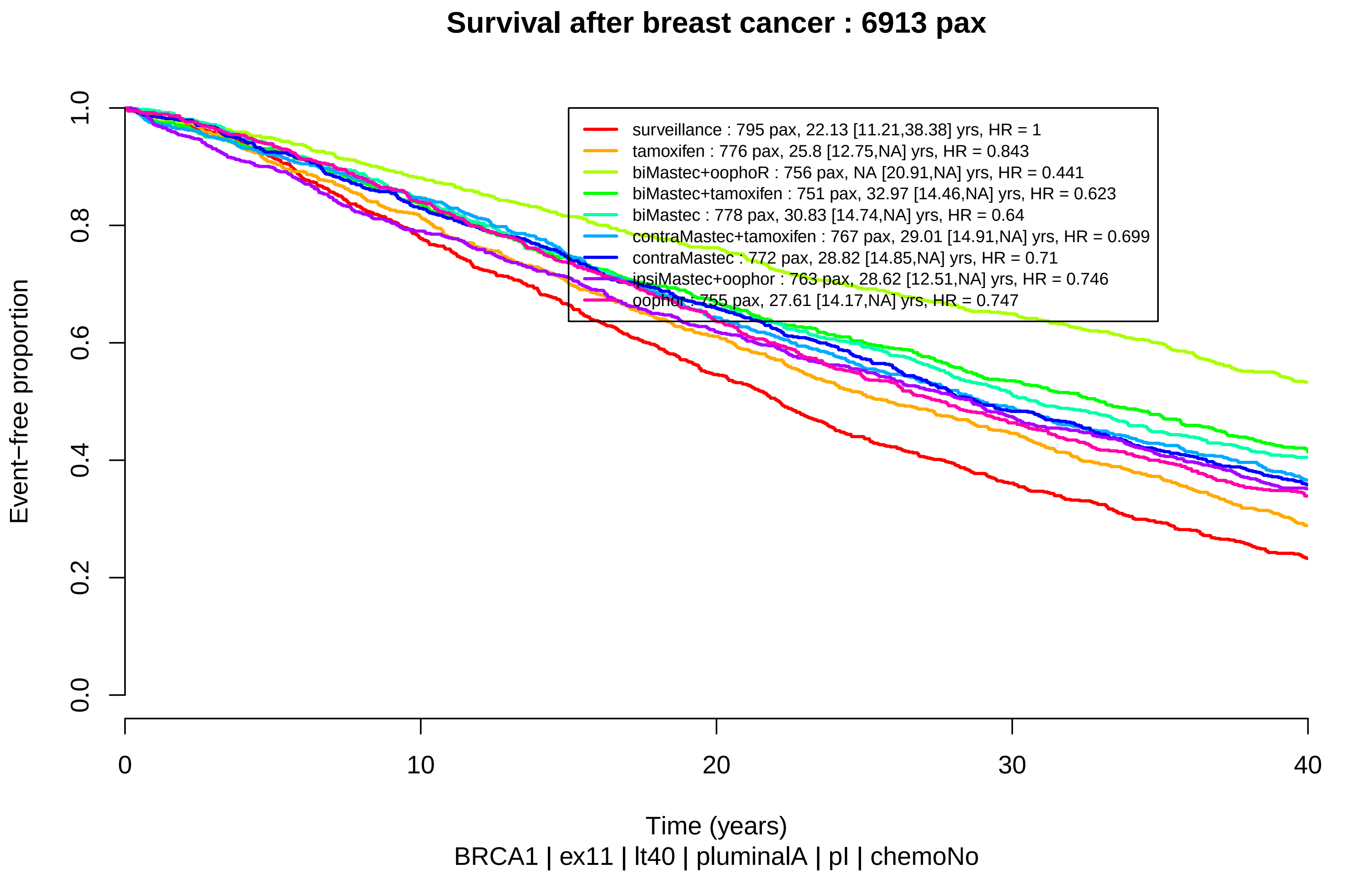
Survival after breast cancer : 6913 pax
surveillance : 795 pax, 22.13 [11.21,38.38] yrs, HR = 1
tamoxifen : 776 pax, 25.8 [12.75,NA] yrs, HR = 0.843
biMastec+oophoR : 756 pax, NA [20.91,NA] yrs, HR = 0.441
biMastec+tamoxifen : 751 pax, 32.97 [14.46,NA] yrs, HR = 0.623
biMastec : 778 pax, 30.83 [14.74,NA] yrs, HR = 0.64
contraMastec+tamoxifen : 767 pax, 29.01 [14.91,NA] yrs, HR = 0.699
contraMastec : 772 pax, 28.82 [14.85,NA] yrs, HR = 0.71
ipsiMastec+oophor : 763 pax, 28.62 [12.51,NA] yrs, HR = 0.746
oophor : 755 pax, 27.61 [14.17,NA] yrs, HR = 0.747
Event−free proportion
0.0
0.2
0.4
0.6
0.8
1.0
0
10
20
30
40
Time (years)
BRCA1 | ex11 | lt40 | pluminalA | pl | chemoNo

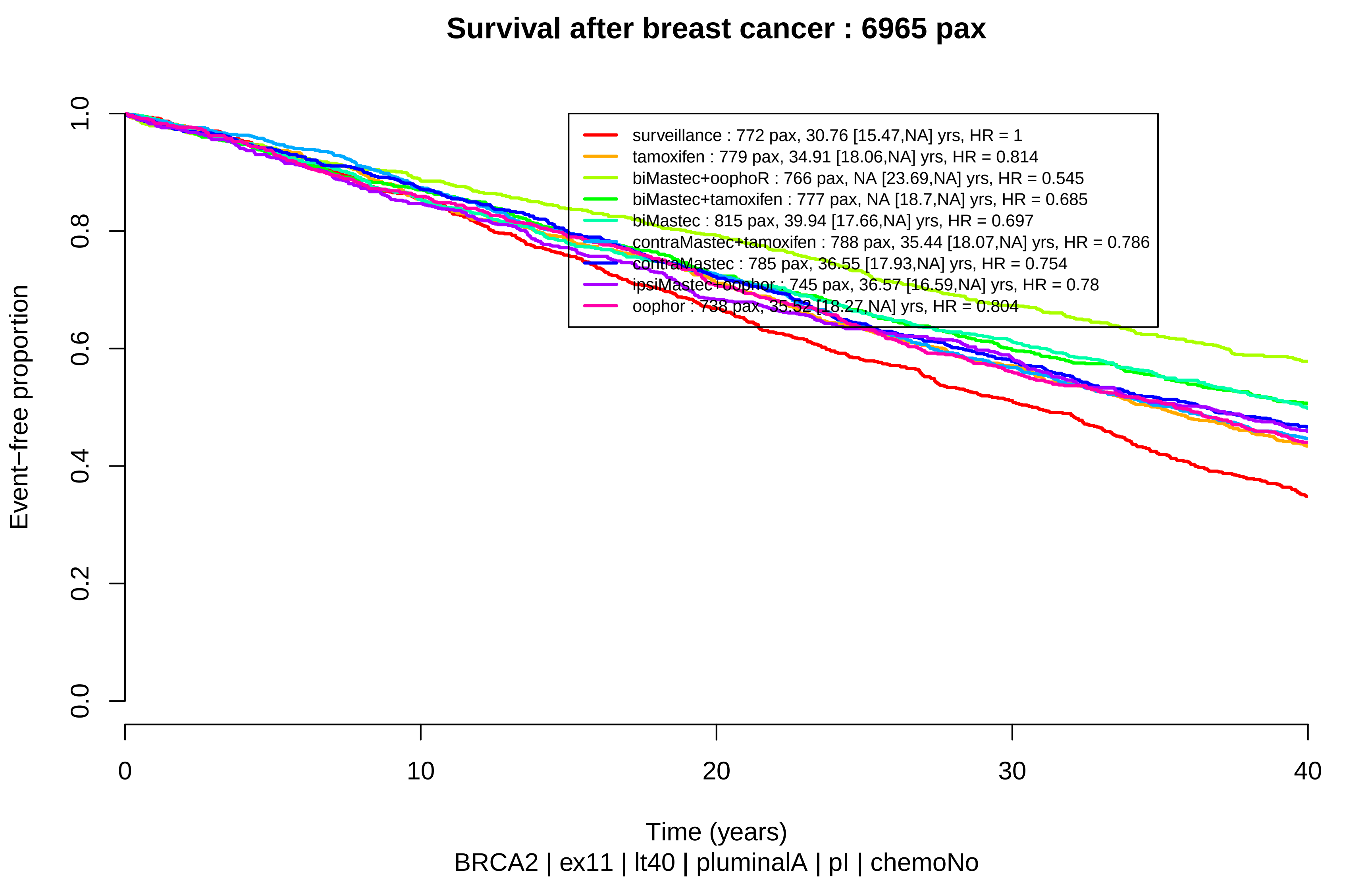
Survival after breast cancer : 6965 pax
surveillance : 772 pax, 30.76 [15.47,NA] yrs, HR = 1
tamoxifen : 779 pax, 34.91 [18.06,NA] yrs, HR = 0.814
biMastec+oophoR : 766 pax, NA [23.69,NA] yrs, HR = 0.545
biMastec+tamoxifen : 777 pax, NA [18.7,NA] yrs, HR = 0.685
biMastec : 815 pax, 39.94 [17.66,NA] yrs, HR = 0.697
contraMastec+tamoxifen : 788 pax, 35.44 [18.07,NA] yrs, HR = 0.786
contraMastec : 785 pax, 36.55 [17.93,NA] yrs, HR = 0.754
ipsiMastec+oophor : 745 pax, 36.57 [16.59,NA] yrs, HR = 0.78
oophor : 738 pax, 35.52 [18.27,NA] yrs, HR = 0.804
Event−free proportion
0.0
0.2
0.4
0.6
0.8
1.0
0
10
20
30
40
Time (years)
BRCA2 | ex11 | lt40 | pluminalA | pl | chemoNo

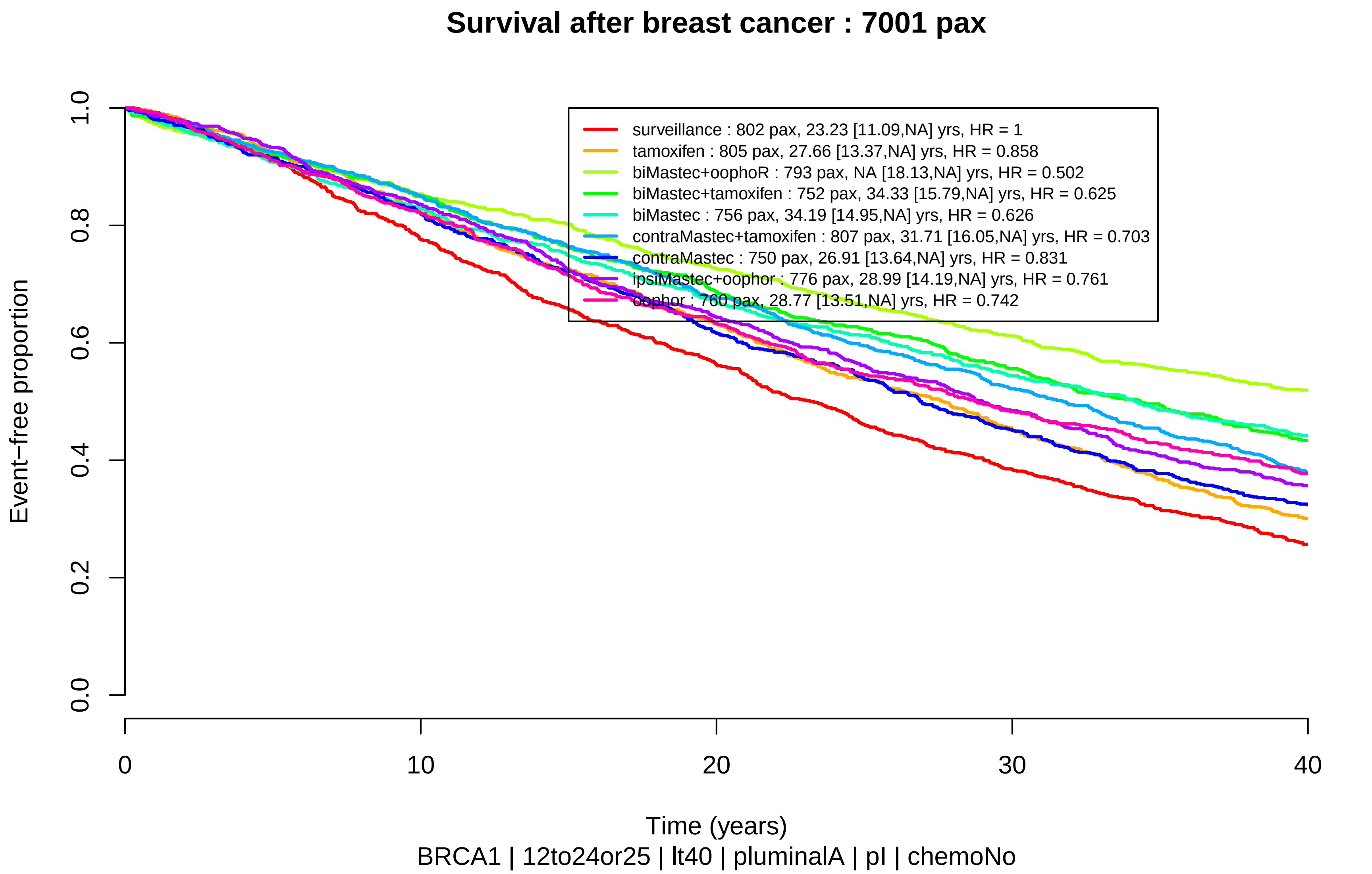

Survival after breast cancer : 7001 pax
surveillance : 802 pax, 23.23 [11.09,NA] yrs, HR = 1
tamoxifen : 805 pax, 27.66 [13.37,NA] yrs, HR = 0.858
biMastec+oophoR : 793 pax, NA [18.13,NA] yrs, HR = 0.502
biMastec+tamoxifen : 752 pax, 34.33 [15.79,NA] yrs, HR = 0.625
biMastec : 756 pax, 34.19 [14.95,NA] yrs, HR = 0.626
contraMastec+tamoxifen : 807 pax, 31.71 [16.05,NA] yrs, HR = 0.703
contraMastec : 750 pax, 26.91 [13.64,NA] yrs, HR = 0.831
ipsiMastec+oophor : 776 pax, 28.99 [14.19,NA] yrs, HR = 0.761
oophor : 760 pax, 28.77 [13.51,NA] yrs, HR = 0.742
Event−free proportion
0.0
0.2
0.4
0.6
0.8
1.0
0
10
20
30
40
Time (years)
BRCA1 | 12to24or25 | lt40 | pluminalA | pI | chemoNo

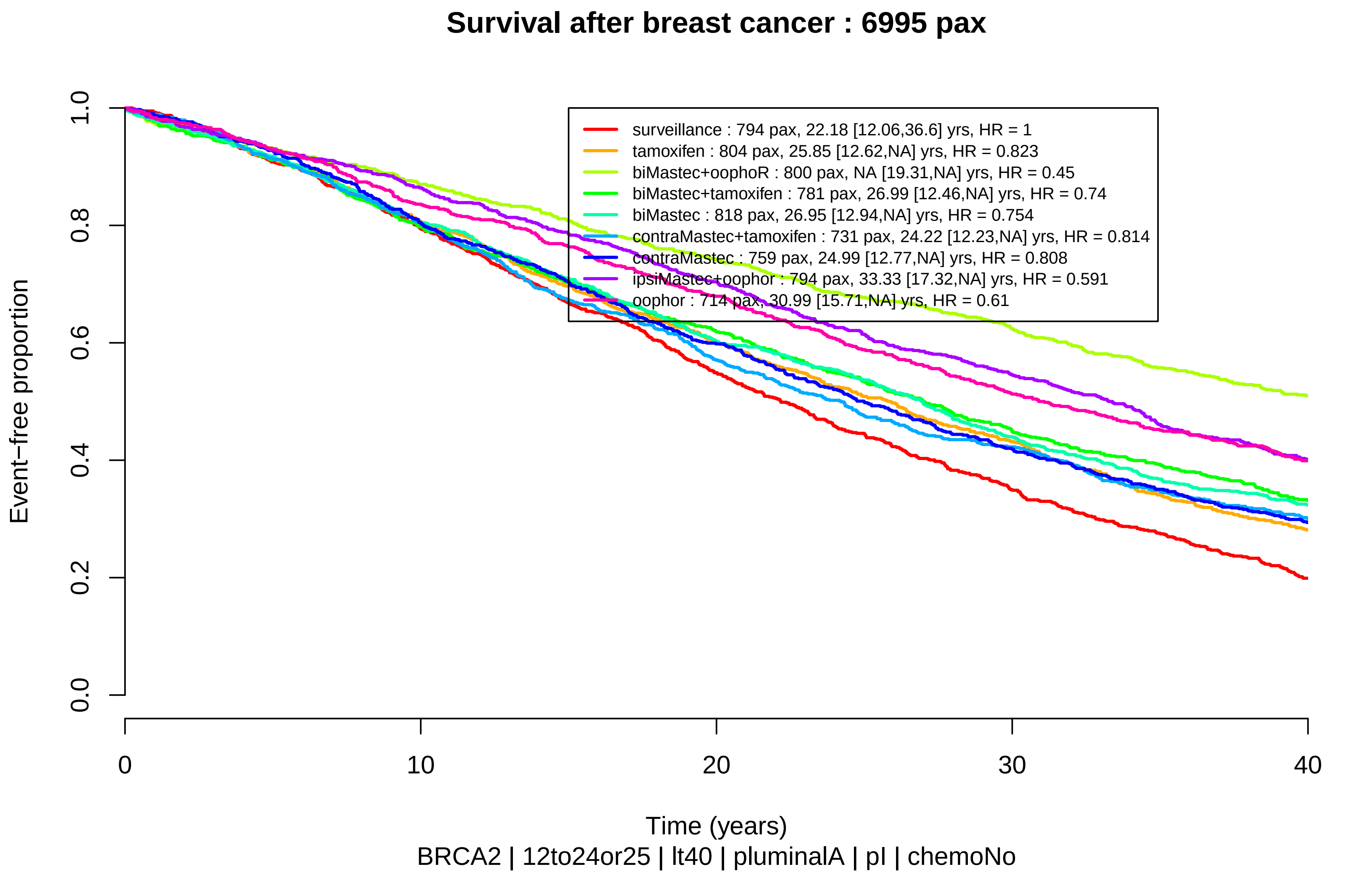
Survival after breast cancer : 6995 pax
surveillance : 794 pax, 22.18 [12.06,36.6] yrs, HR = 1
tamoxifen : 804 pax, 25.85 [12.62,NA] yrs, HR = 0.823
biMastec+oophoR : 800 pax, NA [19.31,NA] yrs, HR = 0.45
biMastec+tamoxifen : 781 pax, 26.99 [12.46,NA] yrs, HR = 0.74
biMastec : 818 pax, 26.95 [12.94,NA] yrs, HR = 0.754
contraMastec+tamoxifen : 731 pax, 24.22 [12.23,NA] yrs, HR = 0.814
contraMastec : 759 pax, 24.99 [12.77,NA] yrs, HR = 0.808
ipsiMastec+oophor : 794 pax, 33.33 [17.32,NA] yrs, HR = 0.591
oophor : 714 pax, 30.99 [15.71,NA] yrs, HR = 0.61
Event−free proportion
0.0
0.2
0.4
0.6
0.8
1.0
0
10
20
30
40
Time (years)
BRCA2 | 12to24or25 | lt40 | pluminalA | pI | chemoNo

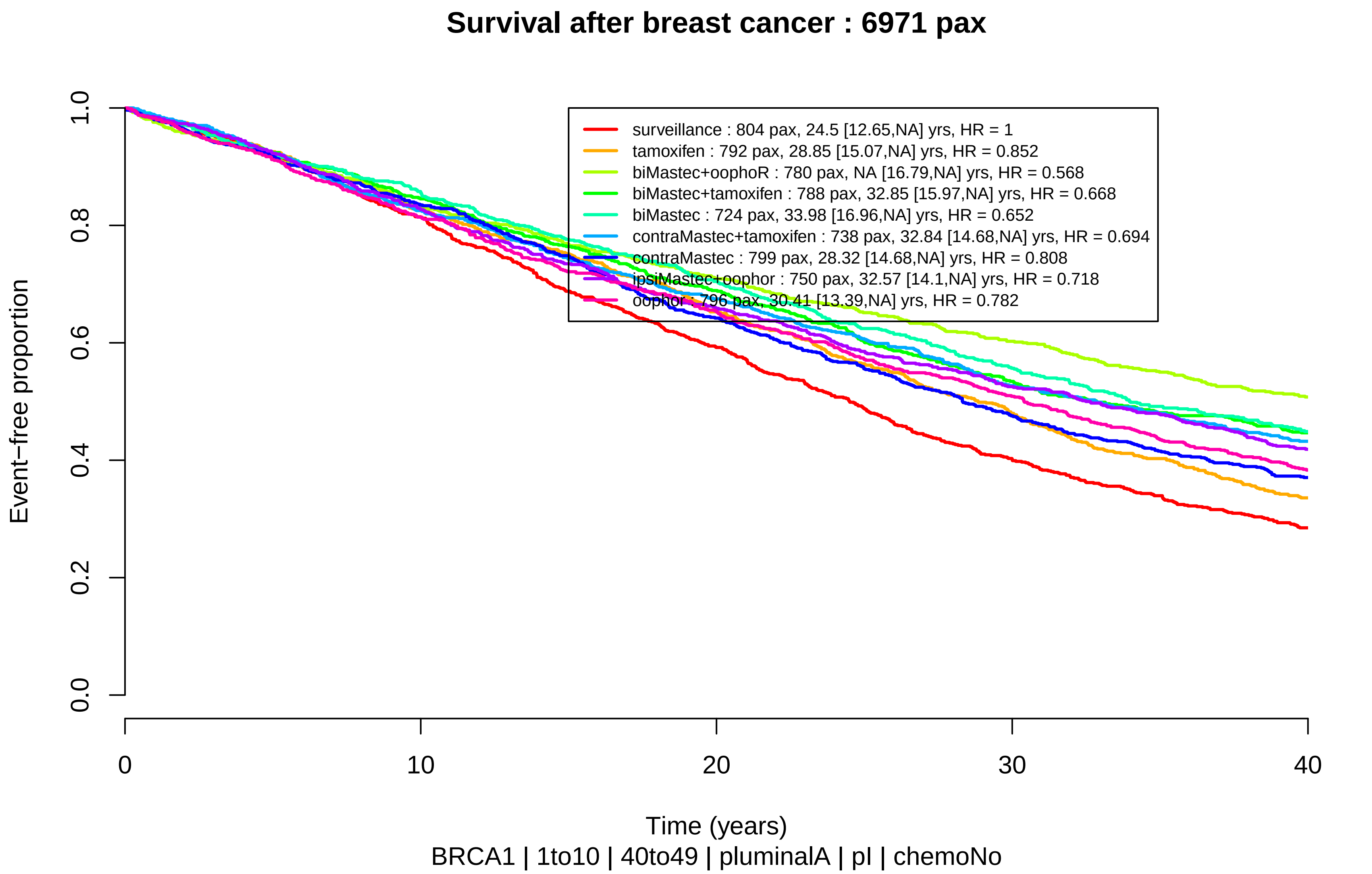

Survival after breast cancer : 6971 pax
surveillance : 804 pax, 24.5 [12.65,NA] yrs, HR = 1
tamoxifen : 792 pax, 28.85 [15.07,NA] yrs, HR = 0.852
biMastec+oophoR : 780 pax, NA [16.79,NA] yrs, HR = 0.568
biMastec+tamoxifen : 788 pax, 32.85 [15.97,NA] yrs, HR = 0.668
biMastec : 724 pax, 33.98 [16.96,NA] yrs, HR = 0.652
contraMastec+tamoxifen : 738 pax, 32.84 [14.68,NA] yrs, HR = 0.694
contraMastec : 799 pax, 28.32 [14.68,NA] yrs, HR = 0.808
ipsiMastec+oophor : 750 pax, 32.57 [14.1,NA] yrs, HR = 0.718
oophor : 796 pax, 30.41 [13.39,NA] yrs, HR = 0.782
Event−free proportion
0.0
0.2
0.4
0.6
0.8
1.0
0
10
20
30
40
Time (years)
BRCA1 | 1to10 | 40to49 | pluminalA | pI | chemoNo

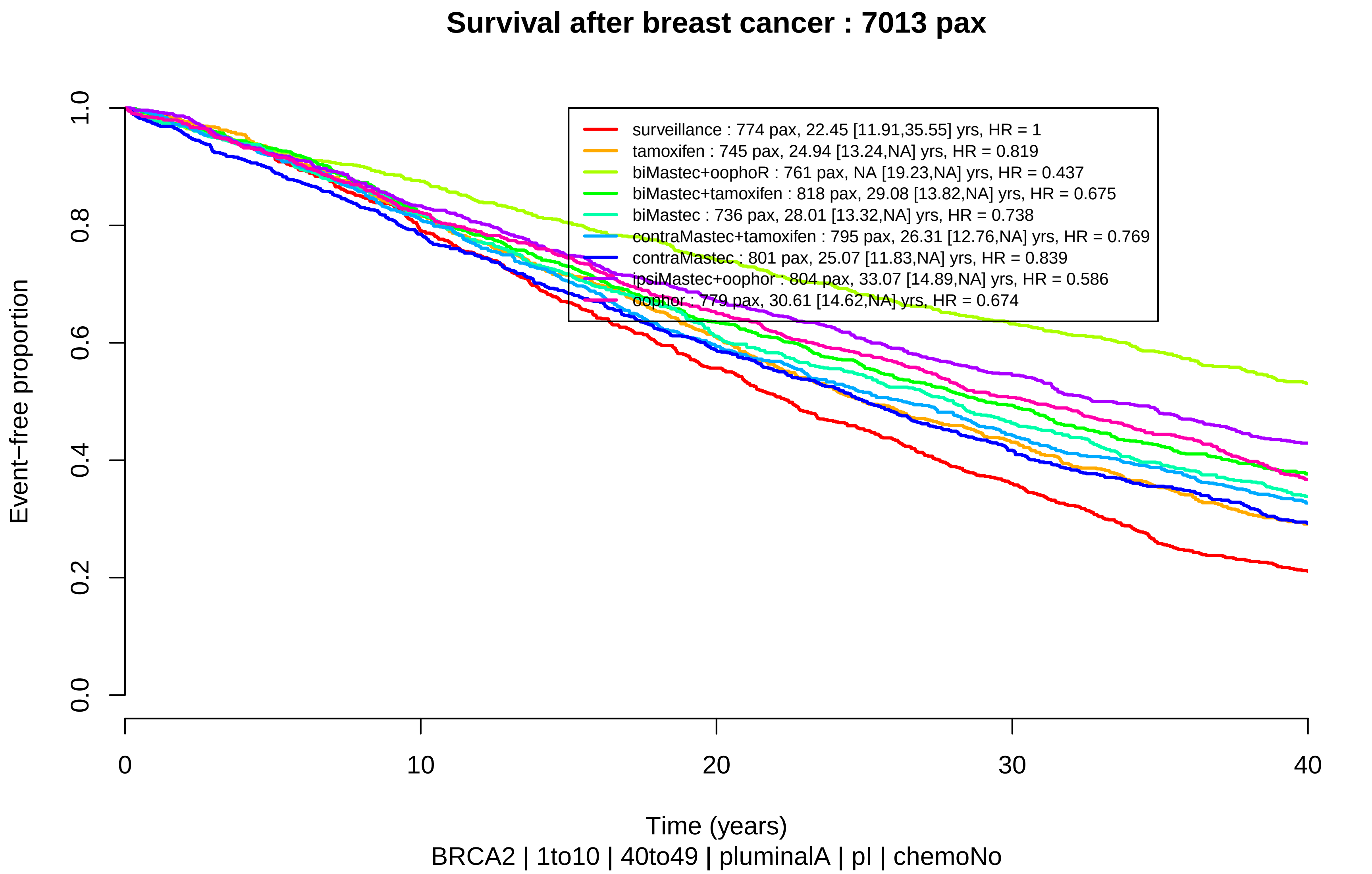

Survival after breast cancer : 7013 pax
surveillance : 774 pax, 22.45 [11.91,35.55] yrs, HR = 1
tamoxifen : 745 pax, 24.94 [13.24,NA] yrs, HR = 0.819
biMastec+oophoR : 761 pax, NA [19.23,NA] yrs, HR = 0.437
biMastec+tamoxifen : 818 pax, 29.08 [13.82,NA] yrs, HR = 0.675
biMastec : 736 pax, 28.01 [13.32,NA] yrs, HR = 0.738
contraMastec+tamoxifen : 795 pax, 26.31 [12.76,NA] yrs, HR = 0.769
contraMastec : 801 pax, 25.07 [11.83,NA] yrs, HR = 0.839
ipsiMastec+oophor : 804 pax, 33.07 [14.89,NA] yrs, HR = 0.586
oophor : 779 pax, 30.61 [14.62,NA] yrs, HR = 0.674
Event−free proportion
0.0
0.2
0.4
0.6
0.8
1.0
0
10
20
30
40
Time (years)
BRCA2 | 1to10 | 40to49 | pluminalA | pI | chemoNo

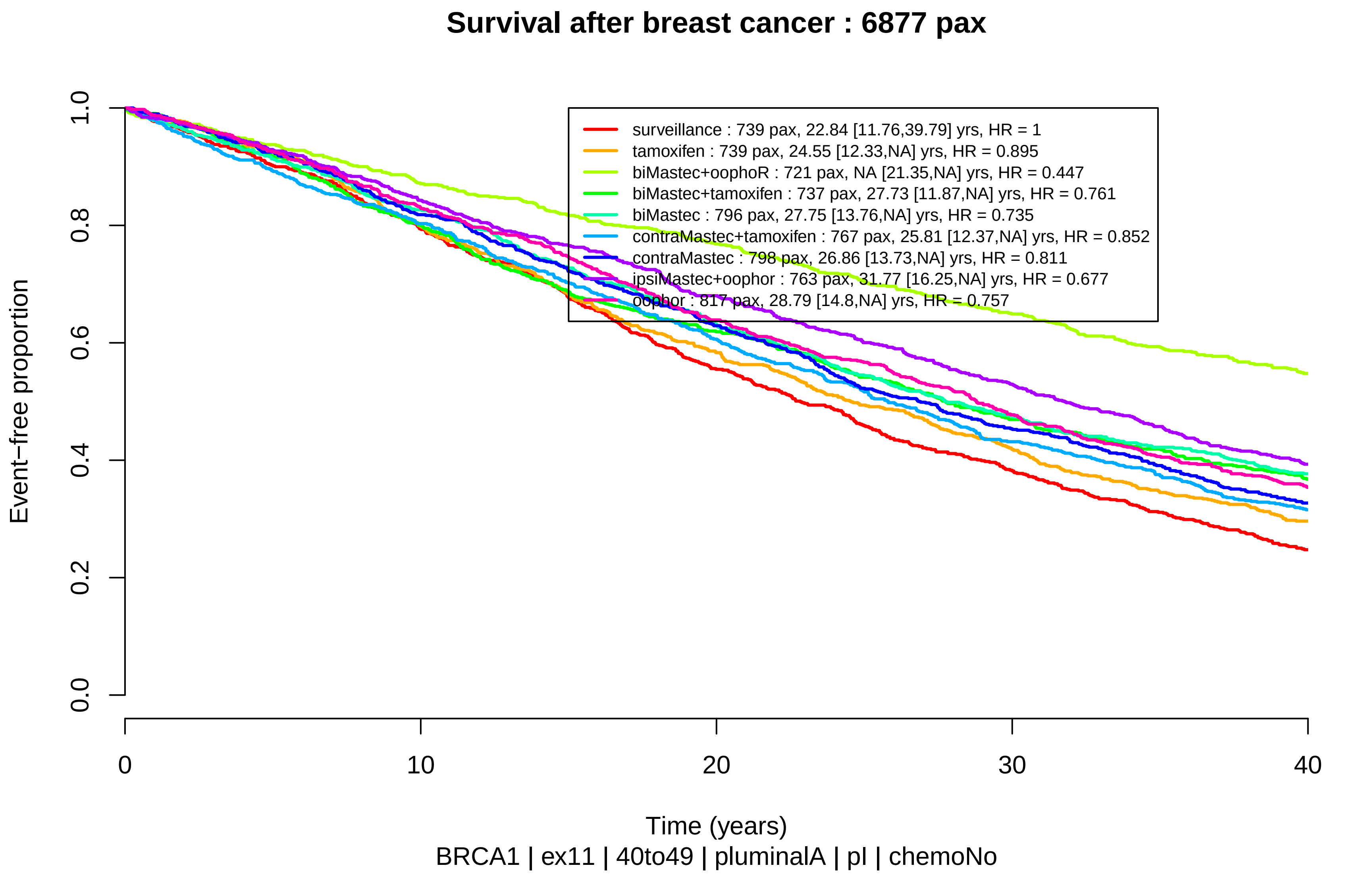

Survival after breast cancer : 6877 pax
surveillance : 739 pax, 22.84 [11.76,39.79] yrs, HR = 1
tamoxifen : 739 pax, 24.55 [12.33,NA] yrs, HR = 0.895
biMastec+oophoR : 721 pax, NA [21.35,NA] yrs, HR = 0.447
biMastec+tamoxifen : 737 pax, 27.73 [11.87,NA] yrs, HR = 0.761
biMastec : 796 pax, 27.75 [13.76,NA] yrs, HR = 0.735
contraMastec+tamoxifen : 767 pax, 25.81 [12.37,NA] yrs, HR = 0.852
contraMastec : 798 pax, 26.86 [13.73,NA] yrs, HR = 0.811
ipsiMastec+oophor : 763 pax, 31.77 [16.25,NA] yrs, HR = 0.677
oophor : 817 pax, 28.79 [14.8,NA] yrs, HR = 0.757
Event−free proportion
0.0
0.2
0.4
0.6
0.8
1.0
0
10
20
30
40
Time (years)
BRCA1 | ex11 | 40to49 | pluminalA | pI | chemoNo

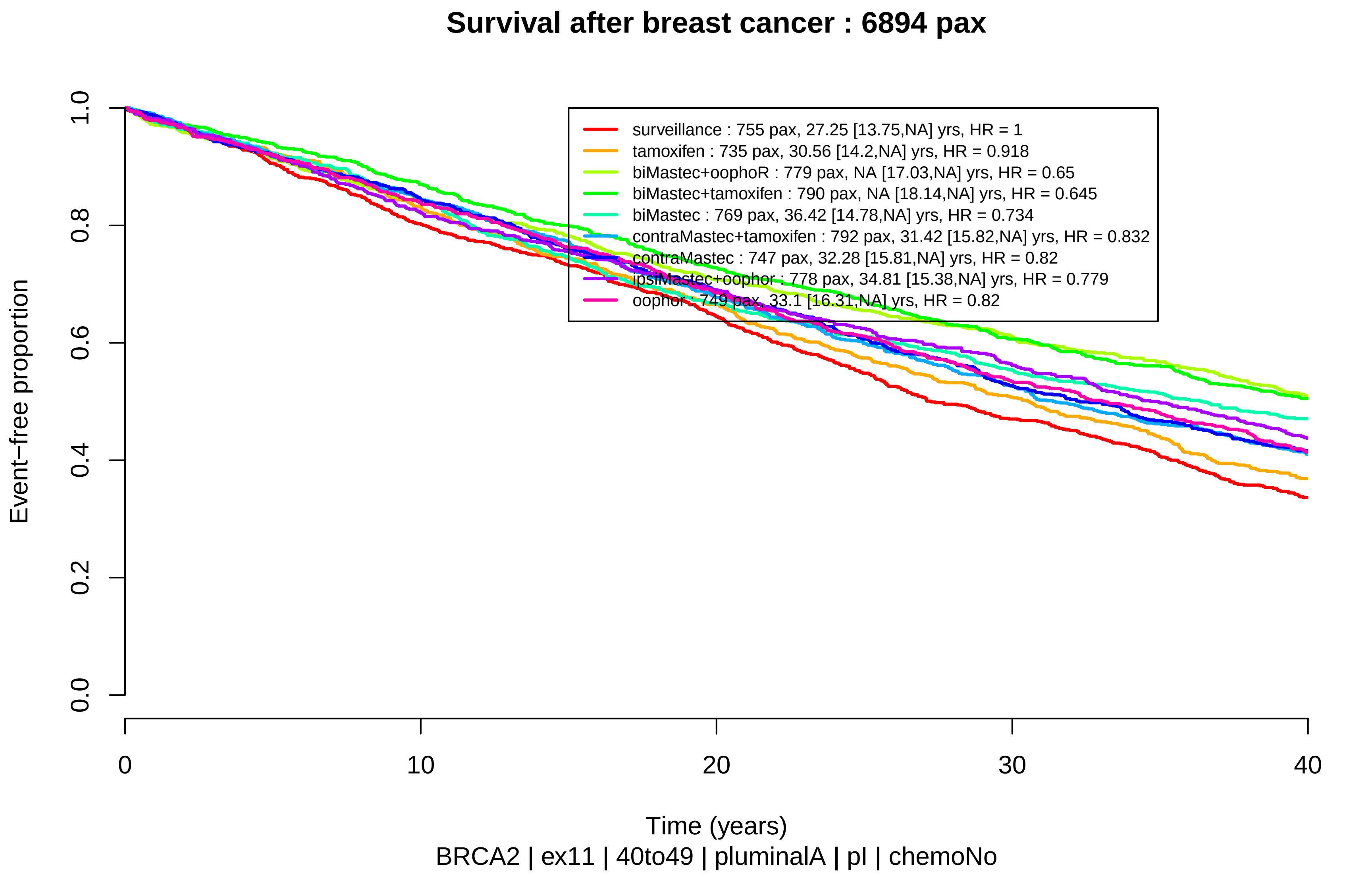

Survival after breast cancer : 6894 pax
surveillance : 755 pax, 27.25 [13.75,NA] yrs, HR = 1
tamoxifen : 735 pax, 30.56 [14.2,NA] yrs, HR = 0.918
biMastec+oophoR : 779 pax, NA [17.03,NA] yrs, HR = 0.65
biMastec+tamoxifen : 790 pax, NA [18.14,NA] yrs, HR = 0.645
biMastec : 769 pax, 36.42 [14.78,NA] yrs, HR = 0.734
contraMastec+tamoxifen : 792 pax, 31.42 [15.82,NA] yrs, HR = 0.832
contraMastec : 747 pax, 32.28 [15.81,NA] yrs, HR = 0.82
ipsiMastec+oophor : 778 pax, 34.81 [15.38,NA] yrs, HR = 0.779
oophor : 749 pax, 33.1 [16.31,NA] yrs, HR = 0.82
Event−free proportion
0.0
0.2
0.4
0.6
0.8
1.0
0
10
20
30
40
Time (years)
BRCA2 | ex11 | 40to49 | pluminalA | pI | chemoNo

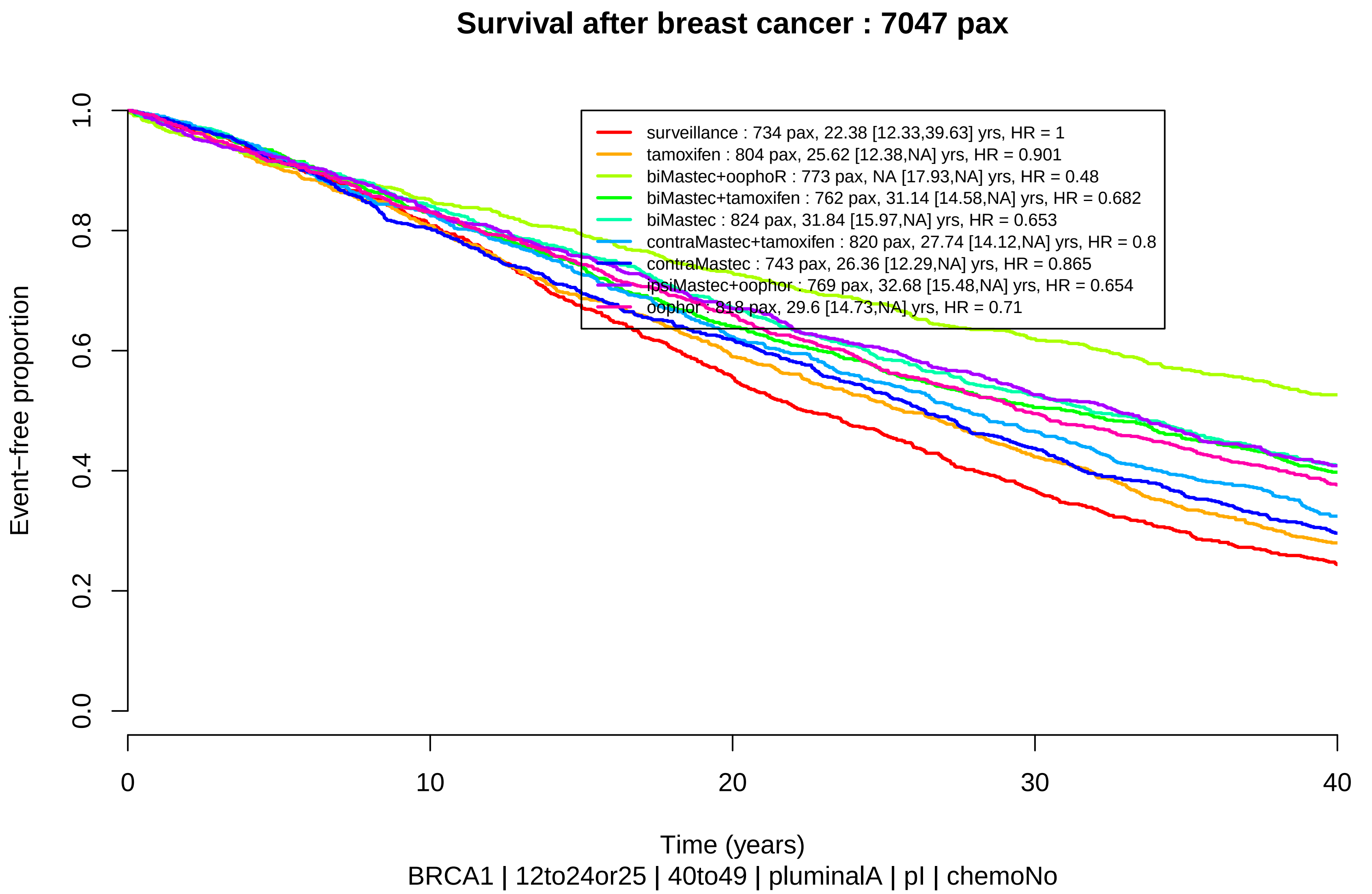
Survival after breast cancer : 7047 pax
Event−free proportion
0.0
0.2
0.4
0.6
0.8
1.0
0
10
20
30
40
Time (years)
surveillance : 734 pax, 22.38 [12.33,39.63] yrs, HR = 1
tamoxifen : 804 pax, 25.62 [12.38,NA] yrs, HR = 0.901
biMastec+oophoR : 773 pax, NA [17.93,NA] yrs, HR = 0.48
biMastec+tamoxifen : 762 pax, 31.14 [14.58,NA] yrs, HR = 0.682
biMastec : 824 pax, 31.84 [15.97,NA] yrs, HR = 0.653
contraMastec+tamoxifen : 820 pax, 27.74 [14.12,NA] yrs, HR = 0.8
contraMastec : 743 pax, 26.36 [12.29,NA] yrs, HR = 0.865
ipsiMastec+oophor : 769 pax, 32.68 [15.48,NA] yrs, HR = 0.654
oophor : 818 pax, 29.6 [14.73,NA] yrs, HR = 0.71
BRCA1 | 12to24or25 | 40to49 | pluminalA | pI | chemoNo

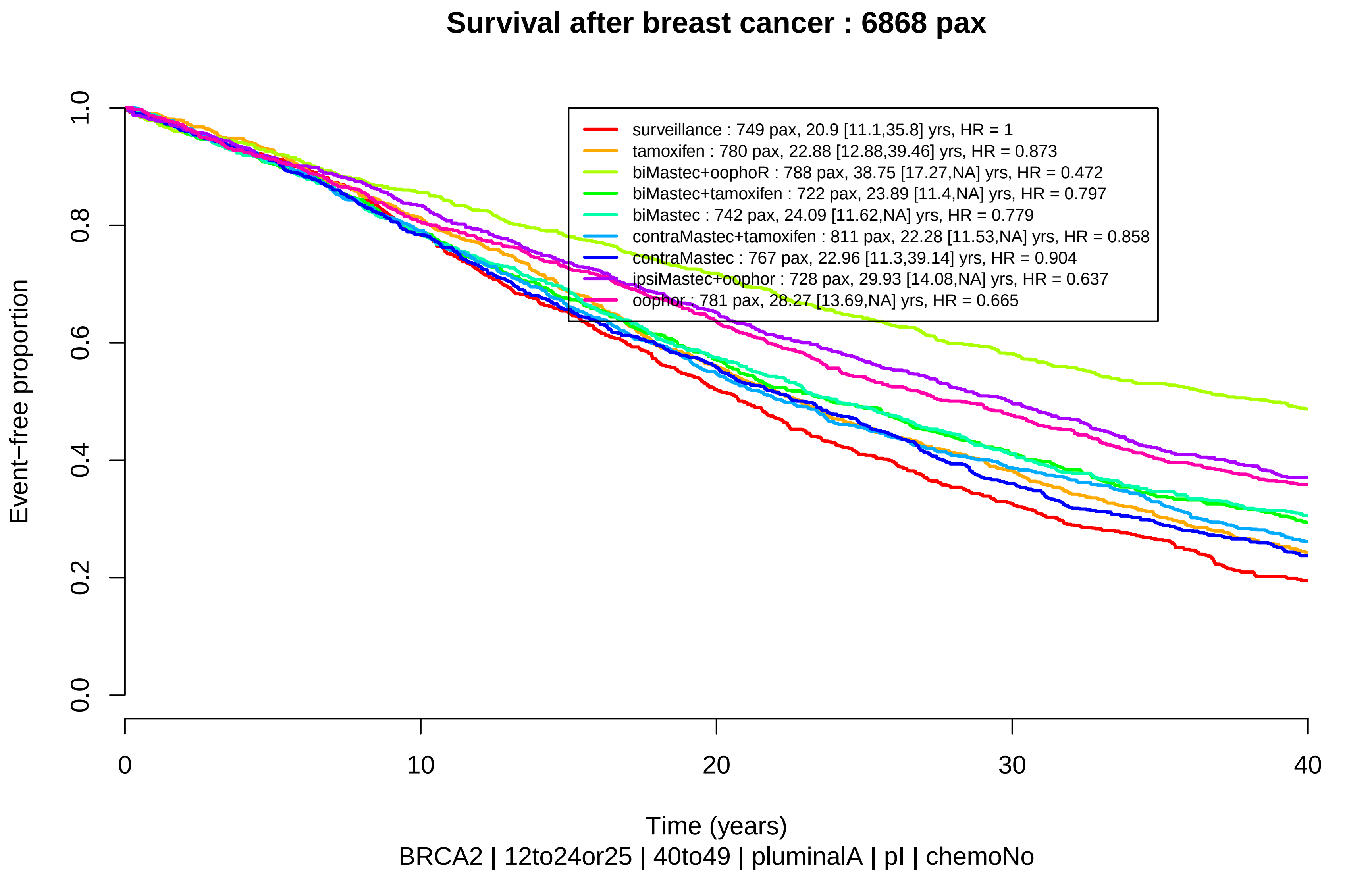
Survival after breast cancer : 6868 pax
Event−free proportion
0.0
0.2
0.4
0.6
0.8
1.0
0
10
20
30
40
Time (years)
BRCA2 | 12to24or25 | 40to49 | pluminalA | pI | chemoNo
surveillance : 749 pax, 20.9 [11.1,35.8] yrs, HR = 1
tamoxifen : 780 pax, 22.88 [12.88,39.46] yrs, HR = 0.873
biMastec+oophoR : 788 pax, 38.75 [17.27,NA] yrs, HR = 0.472
biMastec+tamoxifen : 722 pax, 23.89 [11.4,NA] yrs, HR = 0.797
biMastec : 742 pax, 24.09 [11.62,NA] yrs, HR = 0.779
contraMastec+tamoxifen : 811 pax, 22.28 [11.53,NA] yrs, HR = 0.858
contraMastec : 767 pax, 22.96 [11.3,39.14] yrs, HR = 0.904
ipsiMastec+oophor : 728 pax, 29.93 [14.08,NA] yrs, HR = 0.637
oophor : 781 pax, 28.27 [13.69,NA] yrs, HR = 0.665

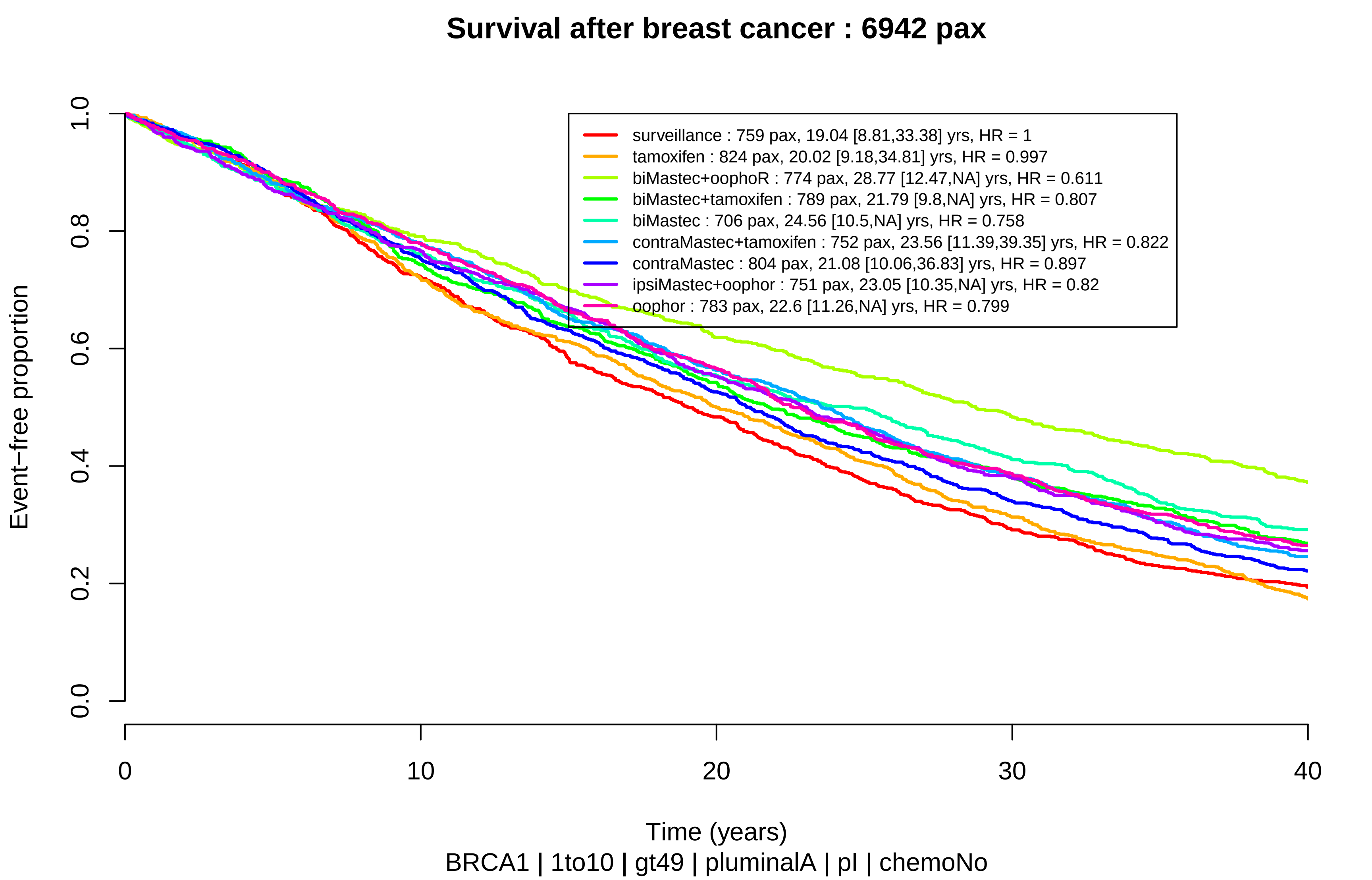

Survival after breast cancer : 6942 pax
surveillance : 759 pax, 19.04 [8.81,33.38] yrs, HR = 1
tamoxifen : 824 pax, 20.02 [9.18,34.81] yrs, HR = 0.997
biMastec+oophoR : 774 pax, 28.77 [12.47,NA] yrs, HR = 0.611
biMastec+tamoxifen : 789 pax, 21.79 [9.8,NA] yrs, HR = 0.807
biMastec : 706 pax, 24.56 [10.5,NA] yrs, HR = 0.758
contraMastec+tamoxifen : 752 pax, 23.56 [11.39,39.35] yrs, HR = 0.822
contraMastec : 804 pax, 21.08 [10.06,36.83] yrs, HR = 0.897
ipsiMastec+oophor : 751 pax, 23.05 [10.35,NA] yrs, HR = 0.82
oophor : 783 pax, 22.6 [11.26,NA] yrs, HR = 0.799
Event−free proportion
0.0
0.2
0.4
0.6
0.8
1.0
0
10
20
30
40
Time (years)
BRCA1 | 1to10 | gt49 | pluminalA | pI | chemoNo

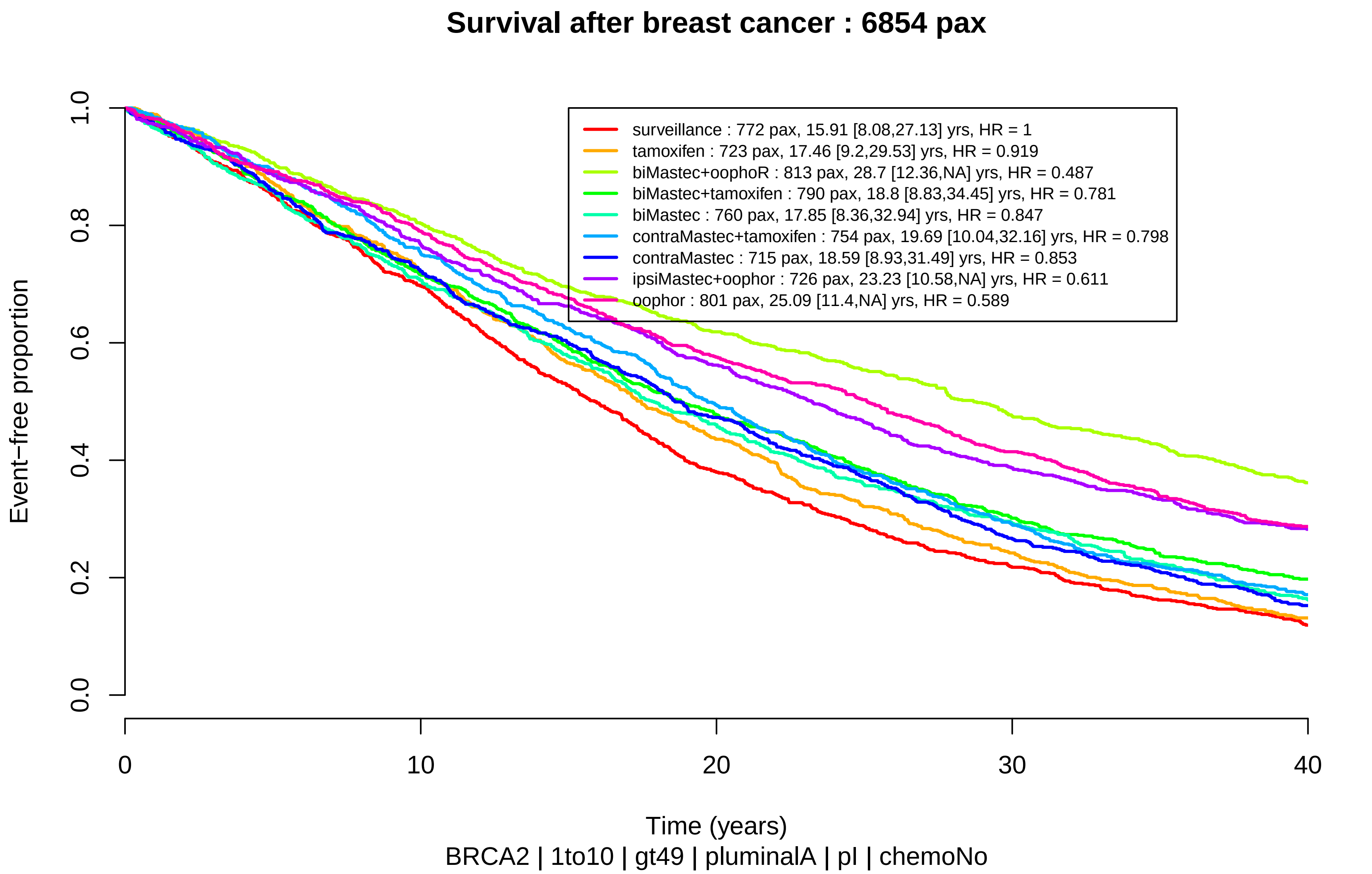

Survival after breast cancer : 6854 pax
surveillance : 772 pax, 15.91 [8.08,27.13] yrs, HR = 1
tamoxifen : 723 pax, 17.46 [9.2,29.53] yrs, HR = 0.919
biMastec+oophoR : 813 pax, 28.7 [12.36,NA] yrs, HR = 0.487
biMastec+tamoxifen : 790 pax, 18.8 [8.83,34.45] yrs, HR = 0.781
biMastec : 760 pax, 17.85 [8.36,32.94] yrs, HR = 0.847
contraMastec+tamoxifen : 754 pax, 19.69 [10.04,32.16] yrs, HR = 0.798
contraMastec : 715 pax, 18.59 [8.93,31.49] yrs, HR = 0.853
ipsiMastec+oophor : 726 pax, 23.23 [10.58,NA] yrs, HR = 0.611
oophor : 801 pax, 25.09 [11.4,NA] yrs, HR = 0.589
Event−free proportion
0.0
0.2
0.4
0.6
0.8
1.0
0
10
20
30
40
Time (years)
BRCA2 | 1to10 | gt49 | pluminalA | pI | chemoNo

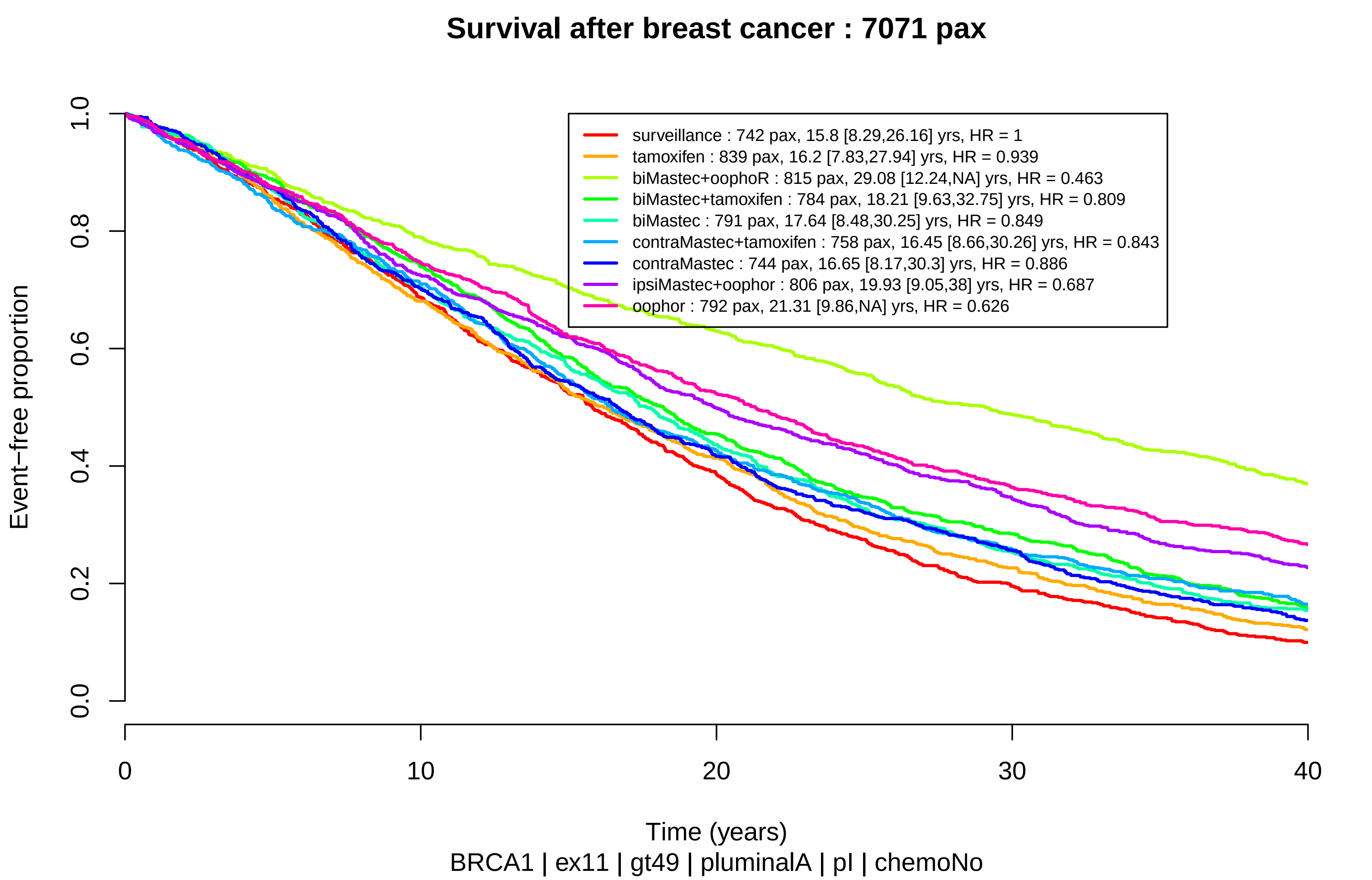

Survival after breast cancer : 7071 pax
Event−free proportion
0.0
0.2
0.4
0.6
0.8
1.0
0
10
20
30
40
Time (years)
BRCA1 | ex11 | gt49 | pluminalA | pI | chemoNo
surveillance : 742 pax, 15.8 [8.29,26.16] yrs, HR = 1
tamoxifen : 839 pax, 16.2 [7.83,27.94] yrs, HR = 0.939
biMastec+oophoR : 815 pax, 29.08 [12.24,NA] yrs, HR = 0.463
biMastec+tamoxifen : 784 pax, 18.21 [9.63,32.75] yrs, HR = 0.809
biMastec : 791 pax, 17.64 [8.48,30.25] yrs, HR = 0.849
contraMastec+tamoxifen : 758 pax, 16.45 [8.66,30.26] yrs, HR = 0.843
contraMastec : 744 pax, 16.65 [8.17,30.3] yrs, HR = 0.886
ipsiMastec+oophor : 806 pax, 19.93 [9.05,38] yrs, HR = 0.687
oophor : 792 pax, 21.31 [9.86,NA] yrs, HR = 0.626

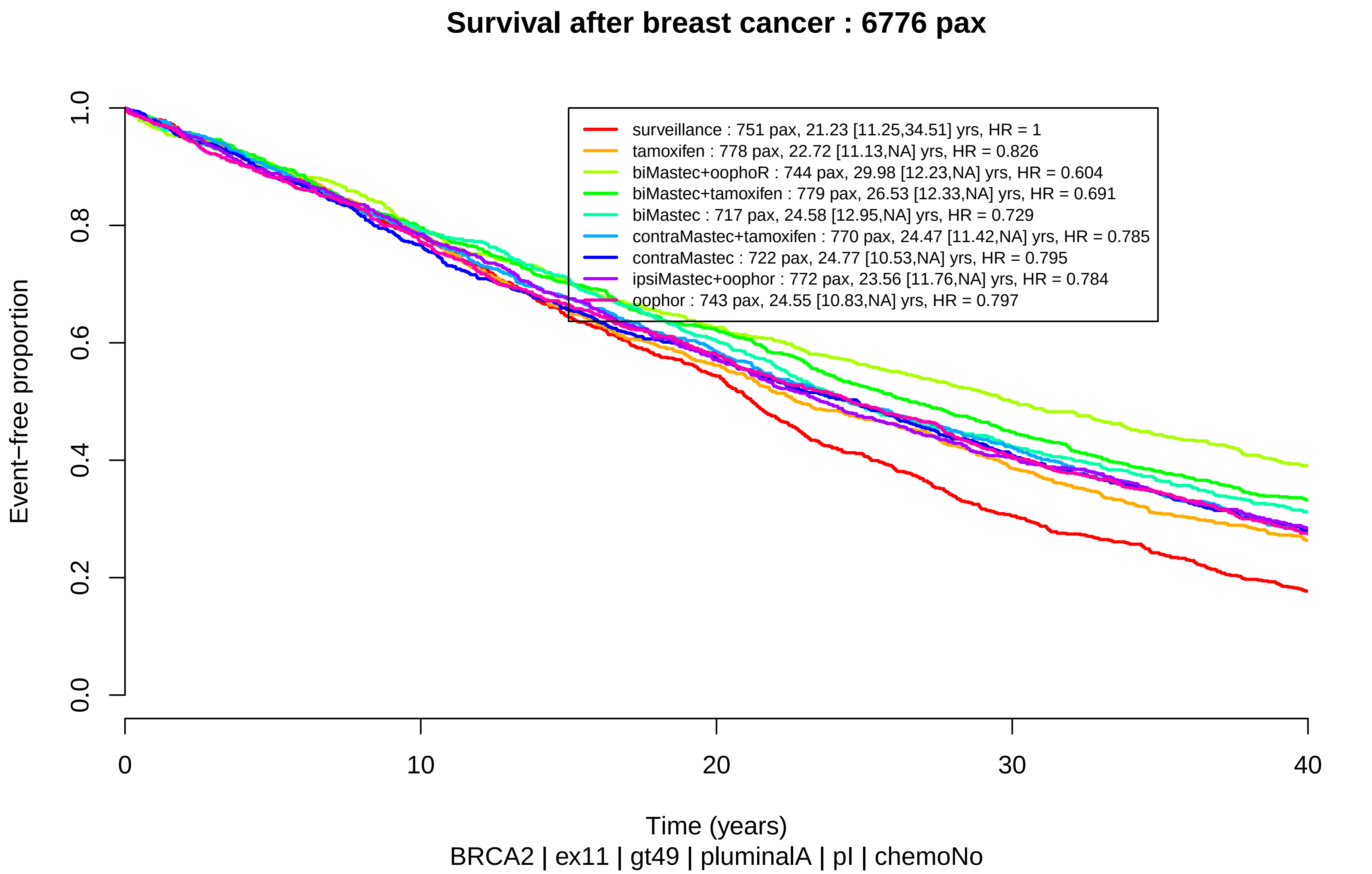

Survival after breast cancer : 6776 pax
surveillance : 751 pax, 21.23 [11.25,34.51] yrs, HR = 1
tamoxifen : 778 pax, 22.72 [11.13,NA] yrs, HR = 0.826
biMastec+oophoR : 744 pax, 29.98 [12.23,NA] yrs, HR = 0.604
biMastec+tamoxifen : 779 pax, 26.53 [12.33,NA] yrs, HR = 0.691
biMastec : 717 pax, 24.58 [12.95,NA] yrs, HR = 0.729
contraMastec+tamoxifen : 770 pax, 24.47 [11.42,NA] yrs, HR = 0.785
contraMastec : 722 pax, 24.77 [10.53,NA] yrs, HR = 0.795
ipsiMastec+oophor : 772 pax, 23.56 [11.76,NA] yrs, HR = 0.784
oophor : 743 pax, 24.55 [10.83,NA] yrs, HR = 0.797
Event−free proportion
0.0
0.2
0.4
0.6
0.8
1.0
0
10
20
30
40
Time (years)
BRCA2 | ex11 | gt49 | pluminalA | pI | chemoNo

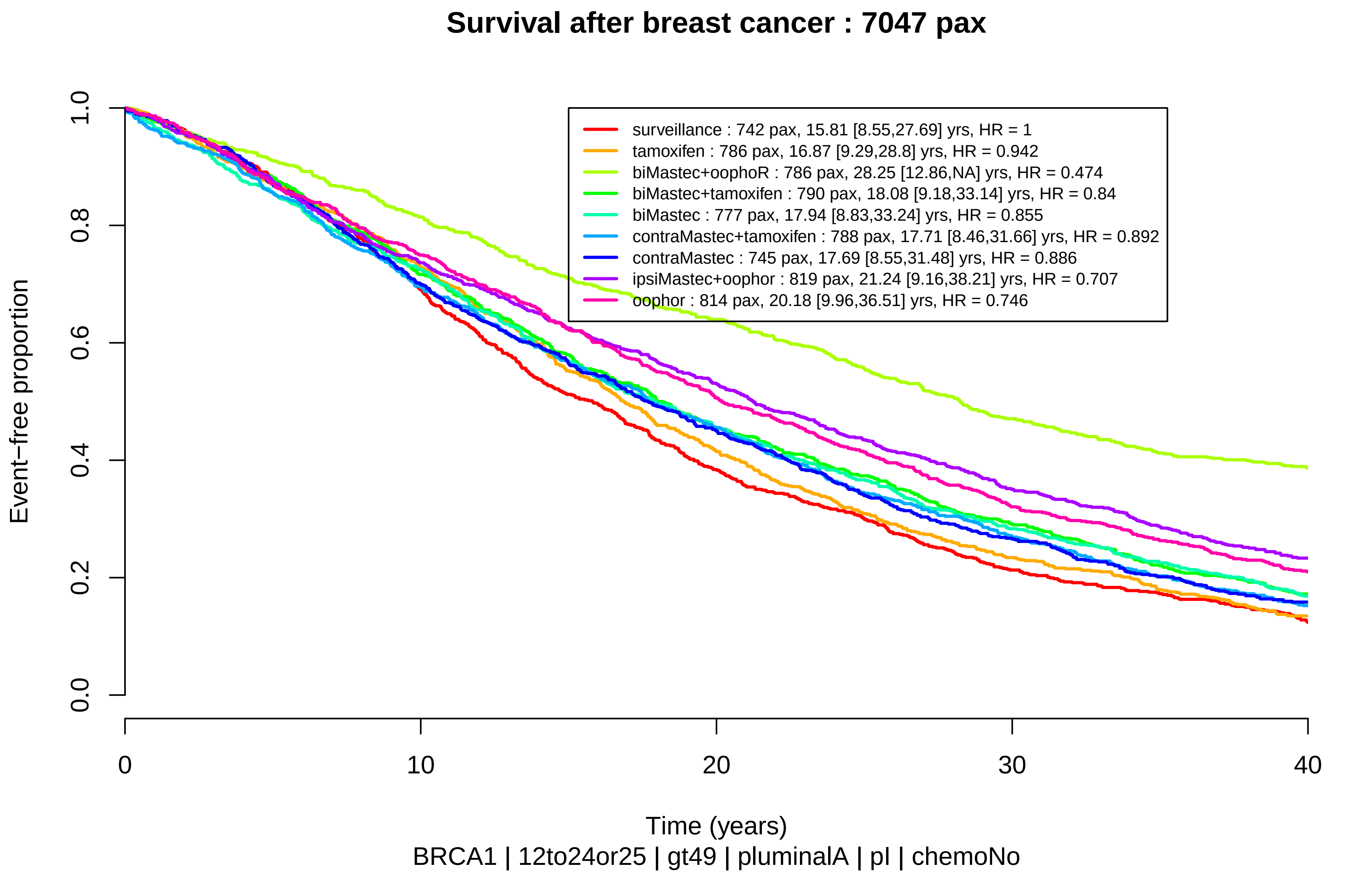

Survival after breast cancer : 7047 pax
surveillance : 742 pax, 15.81 [8.55,27.69] yrs, HR = 1
tamoxifen : 786 pax, 16.87 [9.29,28.8] yrs, HR = 0.942
biMastec+oophoR : 786 pax, 28.25 [12.86,NA] yrs, HR = 0.474
biMastec+tamoxifen : 790 pax, 18.08 [9.18,33.14] yrs, HR = 0.84
biMastec : 777 pax, 17.94 [8.83,33.24] yrs, HR = 0.855
contraMastec+tamoxifen : 788 pax, 17.71 [8.46,31.66] yrs, HR = 0.892
contraMastec : 745 pax, 17.69 [8.55,31.48] yrs, HR = 0.886
ipsiMastec+oophor : 819 pax, 21.24 [9.16,38.21] yrs, HR = 0.707
oophor : 814 pax, 20.18 [9.96,36.51] yrs, HR = 0.746
Event−free proportion
0.0
0.2
0.4
0.6
0.8
1.0
0
10
20
30
40
Time (years)
BRCA1 | 12to24or25 | gt49 | pluminalA | pl | chemoNo

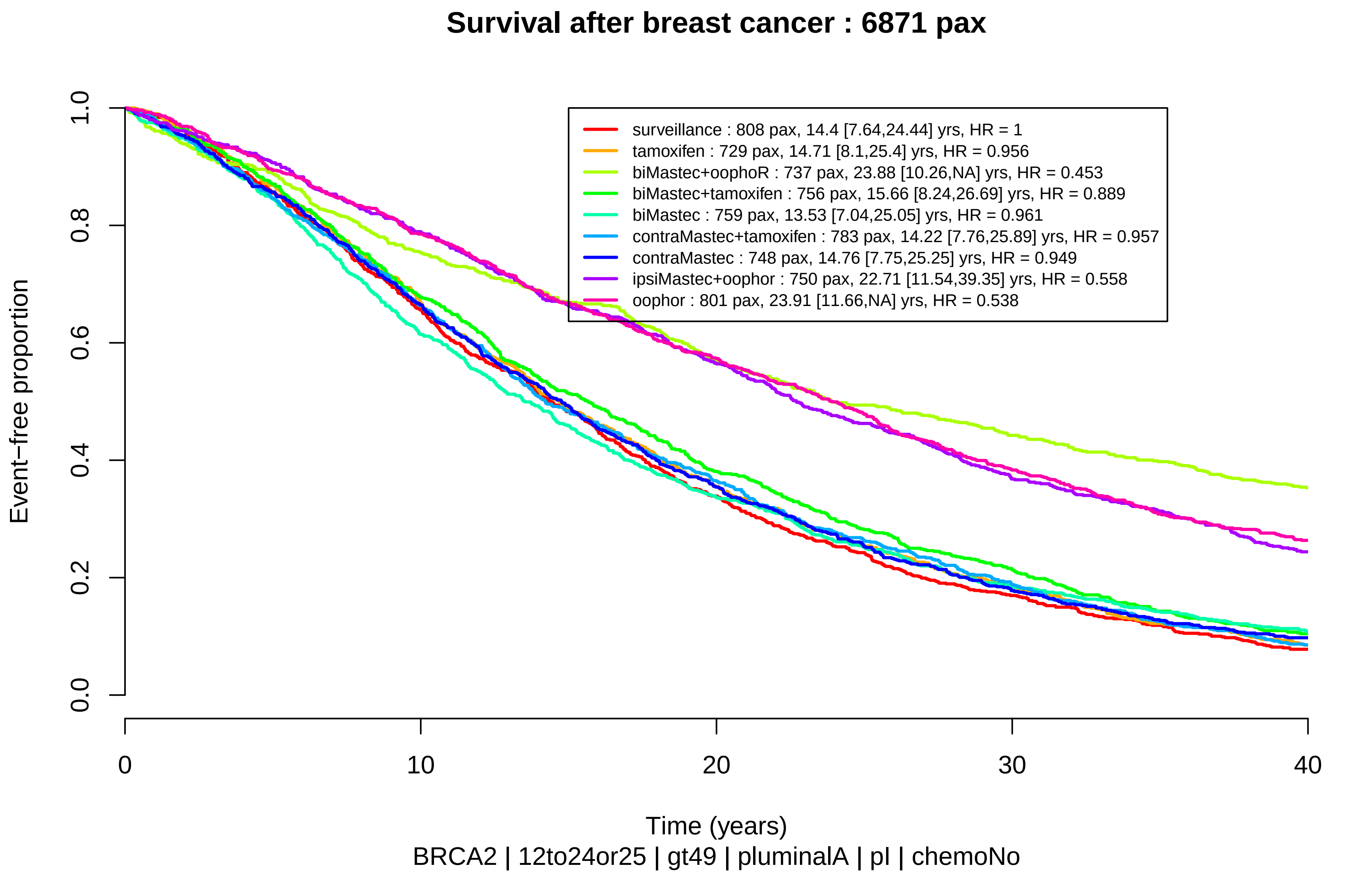
Survival after breast cancer : 6871 pax
Event−free proportion
0.0
0.2
0.4
0.6
0.8
1.0
0
10
20
30
40
Time (years)
BRCA2 | 12to24or25 | gt49 | pluminalA | pI | chemoNo
surveillance : 808 pax, 14.4 [7.64,24.44] yrs, HR = 1
tamoxifen : 729 pax, 14.71 [8.1,25.4] yrs, HR = 0.956
biMastec+oophoR : 737 pax, 23.88 [10.26,NA] yrs, HR = 0.453
biMastec+tamoxifen : 756 pax, 15.66 [8.24,26.69] yrs, HR = 0.889
biMastec : 759 pax, 13.53 [7.04,25.05] yrs, HR = 0.961
contraMastec+tamoxifen : 783 pax, 14.22 [7.76,25.89] yrs, HR = 0.957
contraMastec : 748 pax, 14.76 [7.75,25.25] yrs, HR = 0.949
ipsiMastec+oophor : 750 pax, 22.71 [11.54,39.35] yrs, HR = 0.558
oophor : 801 pax, 23.91 [11.66,NA] yrs, HR = 0.538

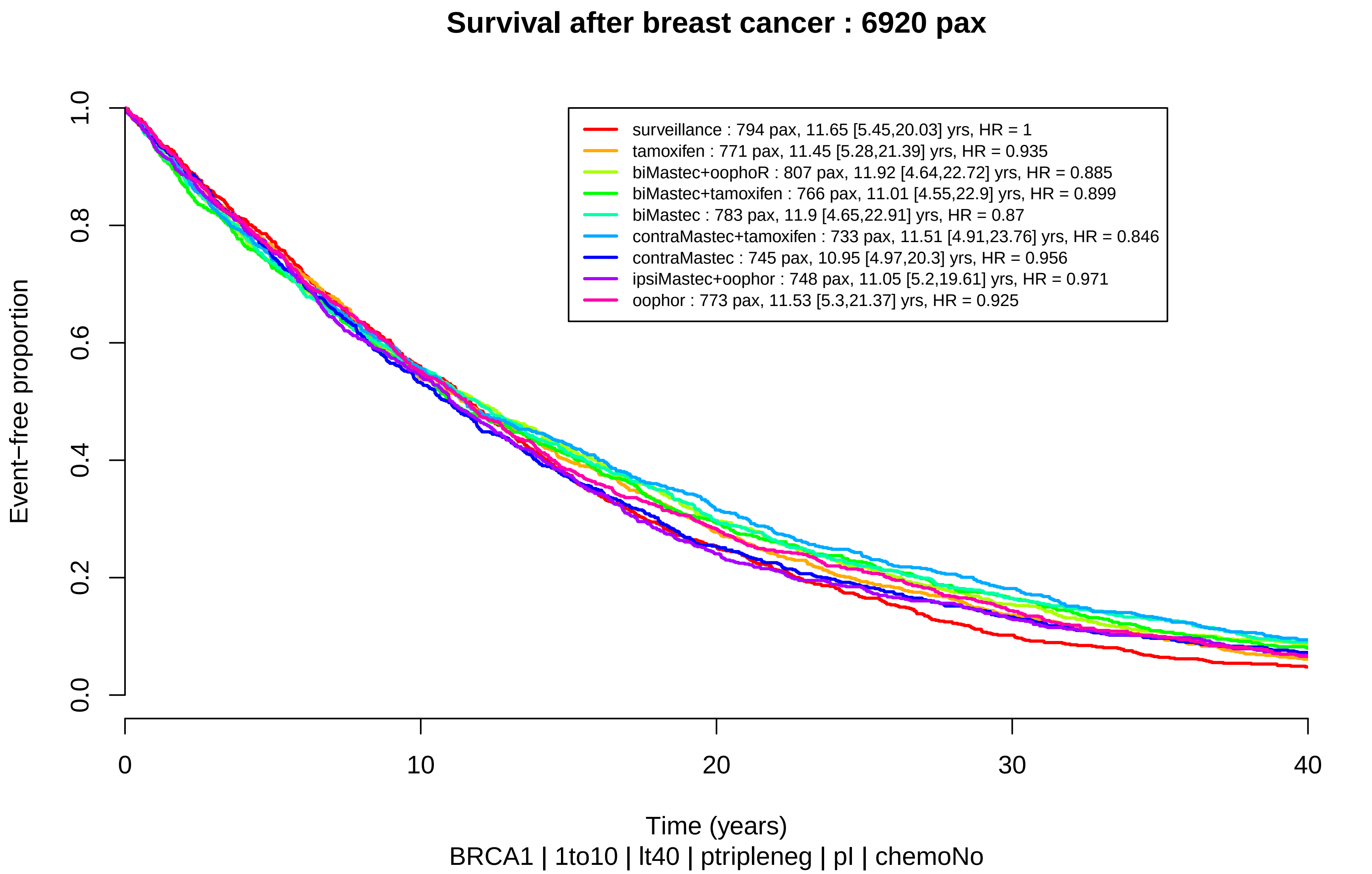
Survival after breast cancer : 6920 pax
Event−free proportion
0.0
0.2
0.4
0.6
0.8
1.0
0
10
20
30
40
Time (years)
BRCA1 | 1to10 | lt40 | ptripleneg | pl | chemoNo
surveillance : 794 pax, 11.65 [5.45,20.03] yrs, HR = 1
tamoxifen : 771 pax, 11.45 [5.28,21.39] yrs, HR = 0.935
biMastec+oophoR : 807 pax, 11.92 [4.64,22.72] yrs, HR = 0.885
biMastec+tamoxifen : 766 pax, 11.01 [4.55,22.9] yrs, HR = 0.899
biMastec : 783 pax, 11.9 [4.65,22.91] yrs, HR = 0.87
contraMastec+tamoxifen : 733 pax, 11.51 [4.91,23.76] yrs, HR = 0.846
contraMastec : 745 pax, 10.95 [4.97,20.3] yrs, HR = 0.956
ipsiMastec+oophor : 748 pax, 11.05 [5.2,19.61] yrs, HR = 0.971
oophor : 773 pax, 11.53 [5.3,21.37] yrs, HR = 0.925

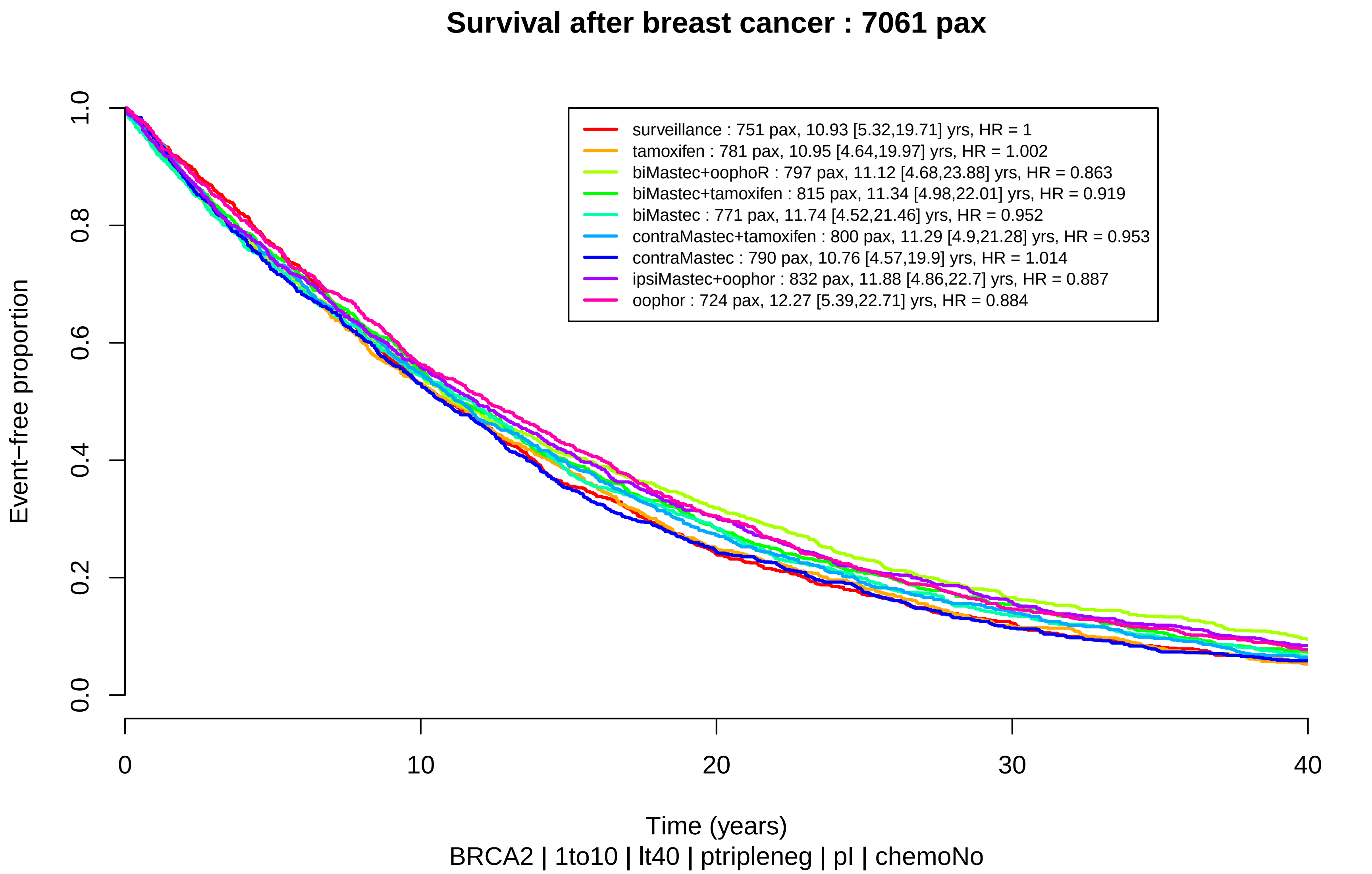
Survival after breast cancer : 7061 pax
Event−free proportion
0.0
0.2
0.4
0.6
0.8
1.0
0
10
20
30
40
Time (years)
BRCA2 | 1to10 | lt40 | ptripleneg | pl | chemoNo
surveillance : 751 pax, 10.93 [5.32,19.71] yrs, HR = 1
tamoxifen : 781 pax, 10.95 [4.64,19.97] yrs, HR = 1.002
biMastec+oophoR : 797 pax, 11.12 [4.68,23.88] yrs, HR = 0.863
biMastec+tamoxifen : 815 pax, 11.34 [4.98,22.01] yrs, HR = 0.919
biMastec : 771 pax, 11.74 [4.52,21.46] yrs, HR = 0.952
contraMastec+tamoxifen : 800 pax, 11.29 [4.9,21.28] yrs, HR = 0.953
contraMastec : 790 pax, 10.76 [4.57,19.9] yrs, HR = 1.014
ipsiMastec+oophor : 832 pax, 11.88 [4.86,22.7] yrs, HR = 0.887
oophor : 724 pax, 12.27 [5.39,22.71] yrs, HR = 0.884

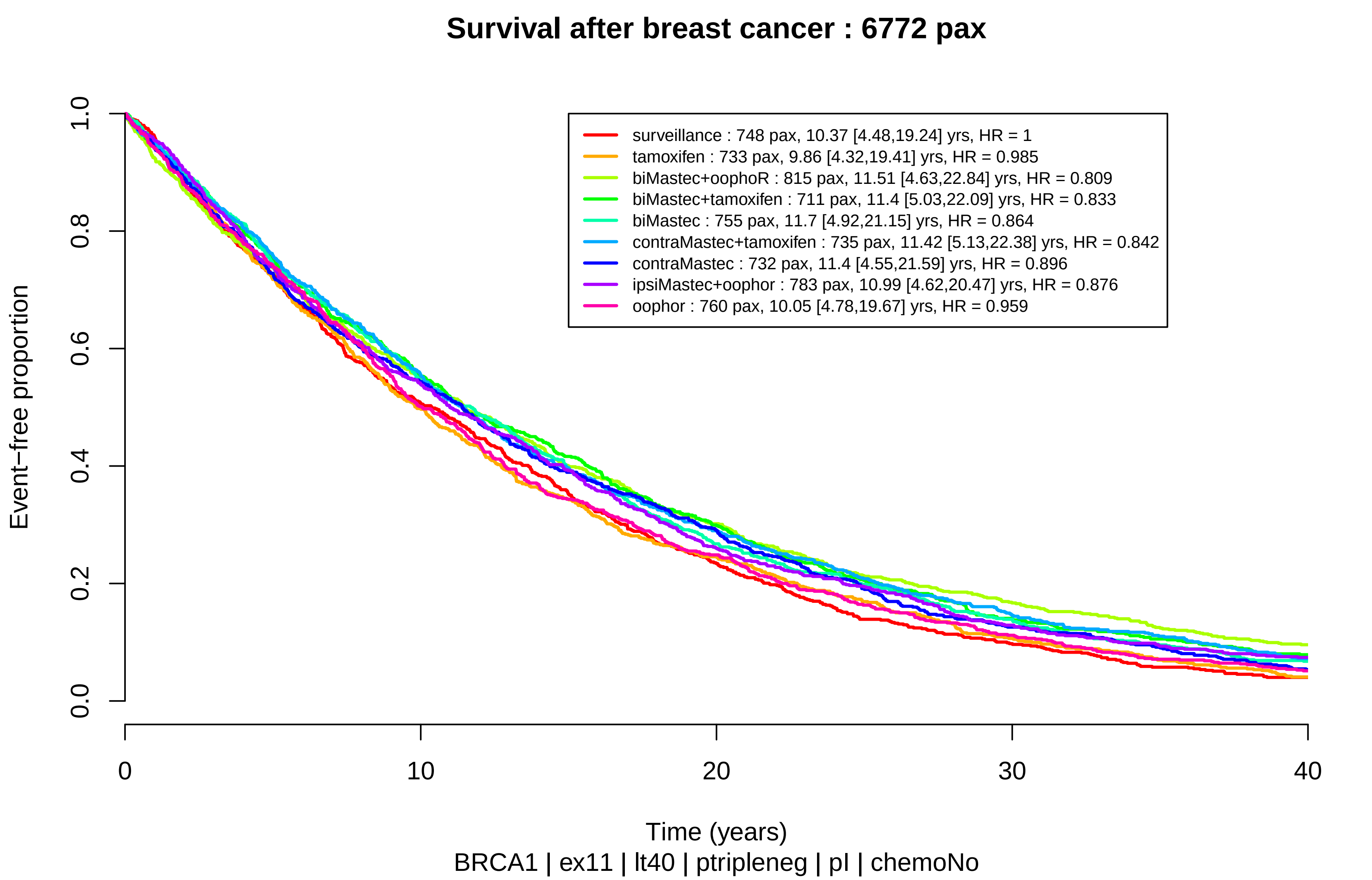

Survival after breast cancer : 6772 pax
surveillance : 748 pax, 10.37 [4.48,19.24] yrs, HR = 1
tamoxifen : 733 pax, 9.86 [4.32,19.41] yrs, HR = 0.985
biMastec+oophoR : 815 pax, 11.51 [4.63,22.84] yrs, HR = 0.809
biMastec+tamoxifen : 711 pax, 11.4 [5.03,22.09] yrs, HR = 0.833
biMastec : 755 pax, 11.7 [4.92,21.15] yrs, HR = 0.864
contraMastec+tamoxifen : 735 pax, 11.42 [5.13,22.38] yrs, HR = 0.842
contraMastec : 732 pax, 11.4 [4.55,21.59] yrs, HR = 0.896
ipsiMastec+oophor : 783 pax, 10.99 [4.62,20.47] yrs, HR = 0.876
oophor : 760 pax, 10.05 [4.78,19.67] yrs, HR = 0.959
Event−free proportion
0.0
0.2
0.4
0.6
0.8
1.0
0
10
20
30
40
Time (years)
BRCA1 | ex11 | lt40 | ptripleneg | pl | chemoNo

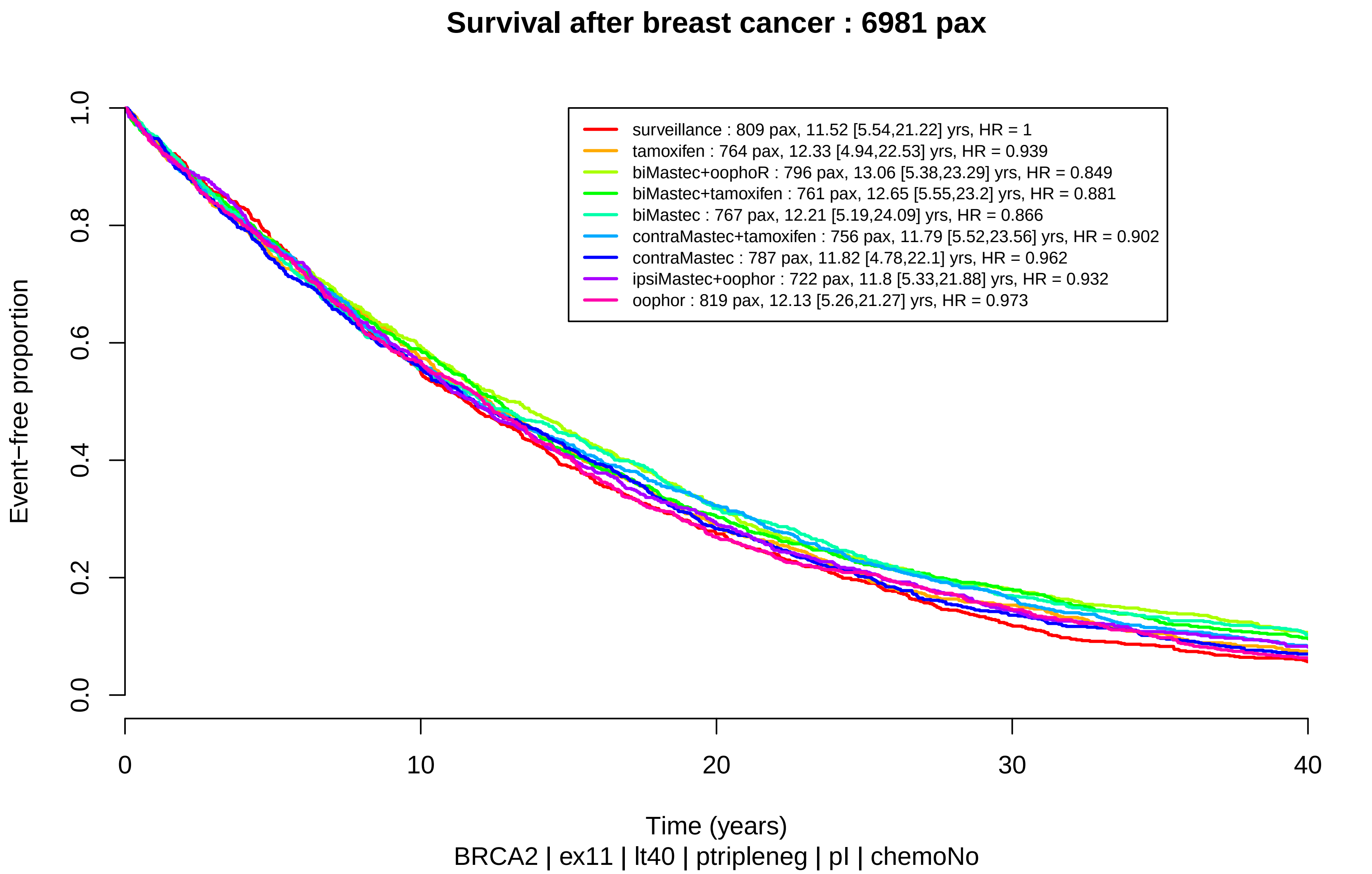

Survival after breast cancer : 6981 pax
surveillance : 809 pax, 11.52 [5.54,21.22] yrs, HR = 1
tamoxifen : 764 pax, 12.33 [4.94,22.53] yrs, HR = 0.939
biMastec+oophoR : 796 pax, 13.06 [5.38,23.29] yrs, HR = 0.849
biMastec+tamoxifen : 761 pax, 12.65 [5.55,23.2] yrs, HR = 0.881
biMastec : 767 pax, 12.21 [5.19,24.09] yrs, HR = 0.866
contraMastec+tamoxifen : 756 pax, 11.79 [5.52,23.56] yrs, HR = 0.902
contraMastec : 787 pax, 11.82 [4.78,22.1] yrs, HR = 0.962
ipsiMastec+oophor : 722 pax, 11.8 [5.33,21.88] yrs, HR = 0.932
oophor : 819 pax, 12.13 [5.26,21.27] yrs, HR = 0.973
Event−free proportion
0.0
0.2
0.4
0.6
0.8
1.0
0
10
20
30
40
Time (years)
BRCA2 | ex11 | lt40 | ptripleneg | pl | chemoNo

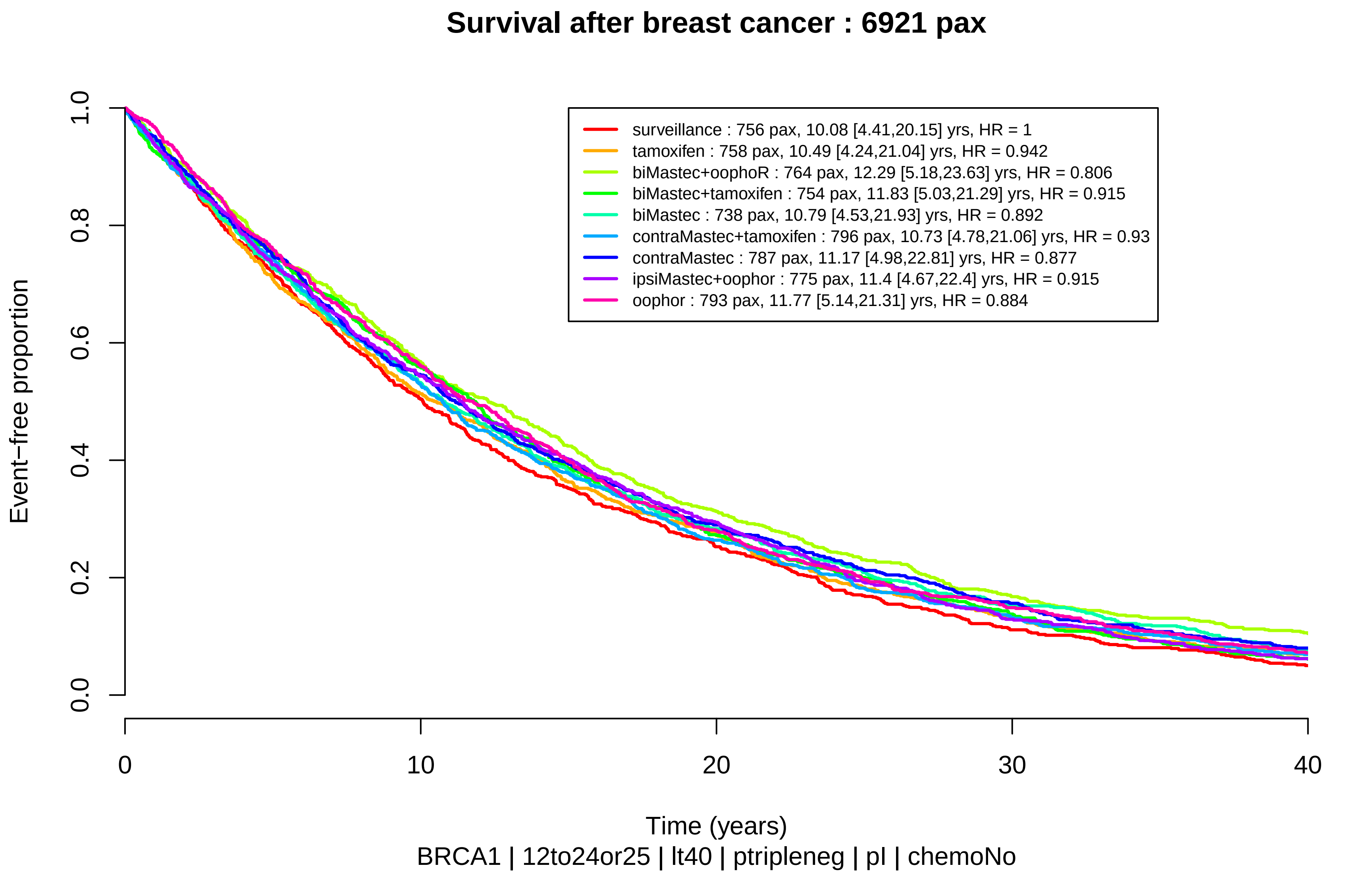

Survival after breast cancer : 6921 pax
Event−free proportion
0.0
0.2
0.4
0.6
0.8
1.0
0
10
20
30
40
Time (years)
BRCA1 | 12to24or25 | lt40 | ptripleneg | pl | chemoNo
surveillance : 756 pax, 10.08 [4.41,20.15] yrs, HR = 1
tamoxifen : 758 pax, 10.49 [4.24,21.04] yrs, HR = 0.942
biMastec+oophoR : 764 pax, 12.29 [5.18,23.63] yrs, HR = 0.806
biMastec+tamoxifen : 754 pax, 11.83 [5.03,21.29] yrs, HR = 0.915
biMastec : 738 pax, 10.79 [4.53,21.93] yrs, HR = 0.892
contraMastec+tamoxifen : 796 pax, 10.73 [4.78,21.06] yrs, HR = 0.93
contraMastec : 787 pax, 11.17 [4.98,22.81] yrs, HR = 0.877
ipsiMastec+oophor : 775 pax, 11.4 [4.67,22.4] yrs, HR = 0.915
oophor : 793 pax, 11.77 [5.14,21.31] yrs, HR = 0.884

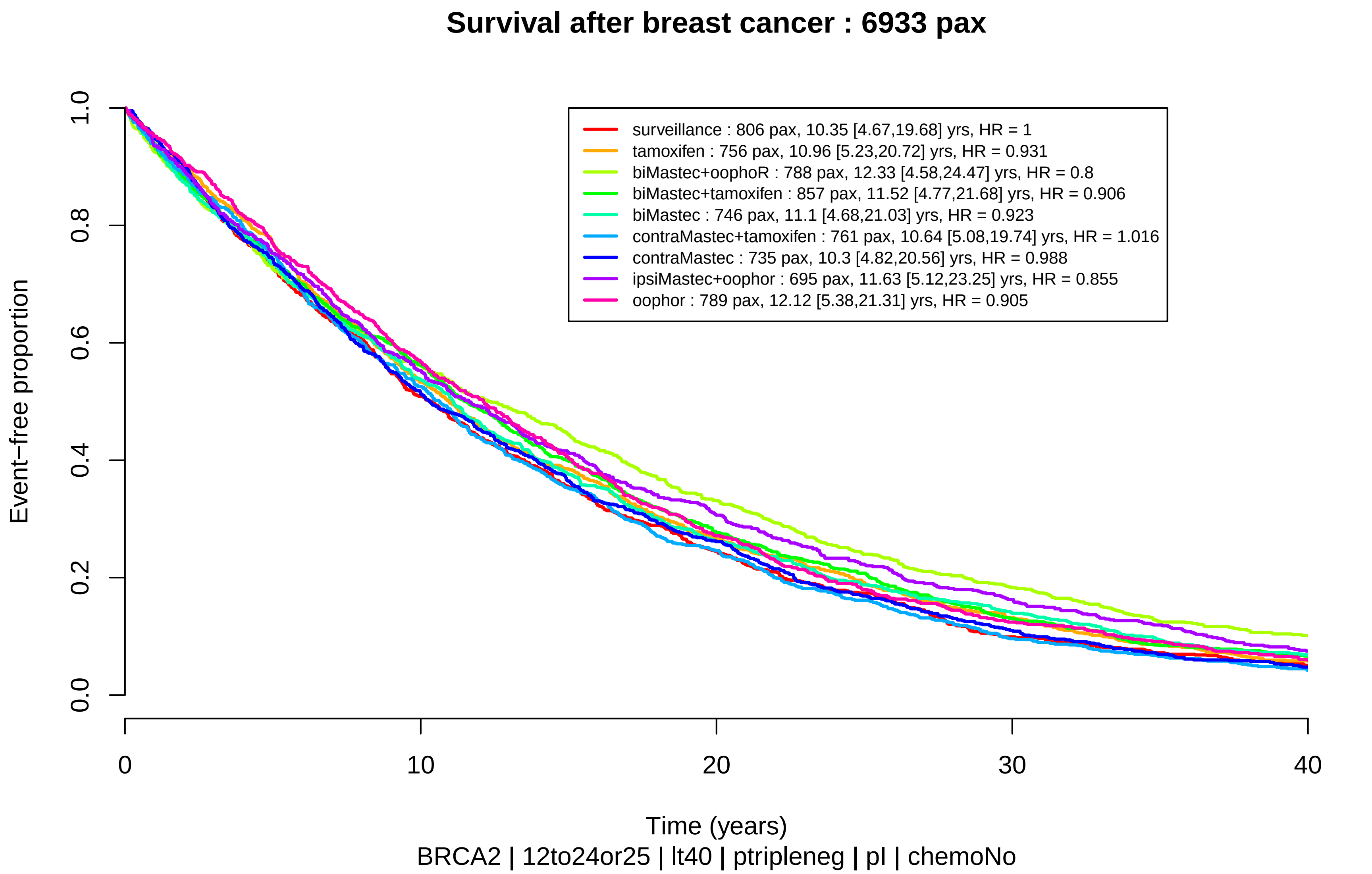
Survival after breast cancer : 6933 pax
surveillance : 806 pax, 10.35 [4.67,19.68] yrs, HR = 1
tamoxifen : 756 pax, 10.96 [5.23,20.72] yrs, HR = 0.931
biMastec+oophoR : 788 pax, 12.33 [4.58,24.47] yrs, HR = 0.8
biMastec+tamoxifen : 857 pax, 11.52 [4.77,21.68] yrs, HR = 0.906
biMastec : 746 pax, 11.1 [4.68,21.03] yrs, HR = 0.923
contraMastec+tamoxifen : 761 pax, 10.64 [5.08,19.74] yrs, HR = 1.016
contraMastec : 735 pax, 10.3 [4.82,20.56] yrs, HR = 0.988
ipsiMastec+oophor : 695 pax, 11.63 [5.12,23.25] yrs, HR = 0.855
oophor : 789 pax, 12.12 [5.38,21.31] yrs, HR = 0.905
Event−free proportion
0.0
0.2
0.4
0.6
0.8
1.0
0
10
20
30
40
Time (years)
BRCA2 | 12to24or25 | lt40 | ptripleneg | pl | chemoNo

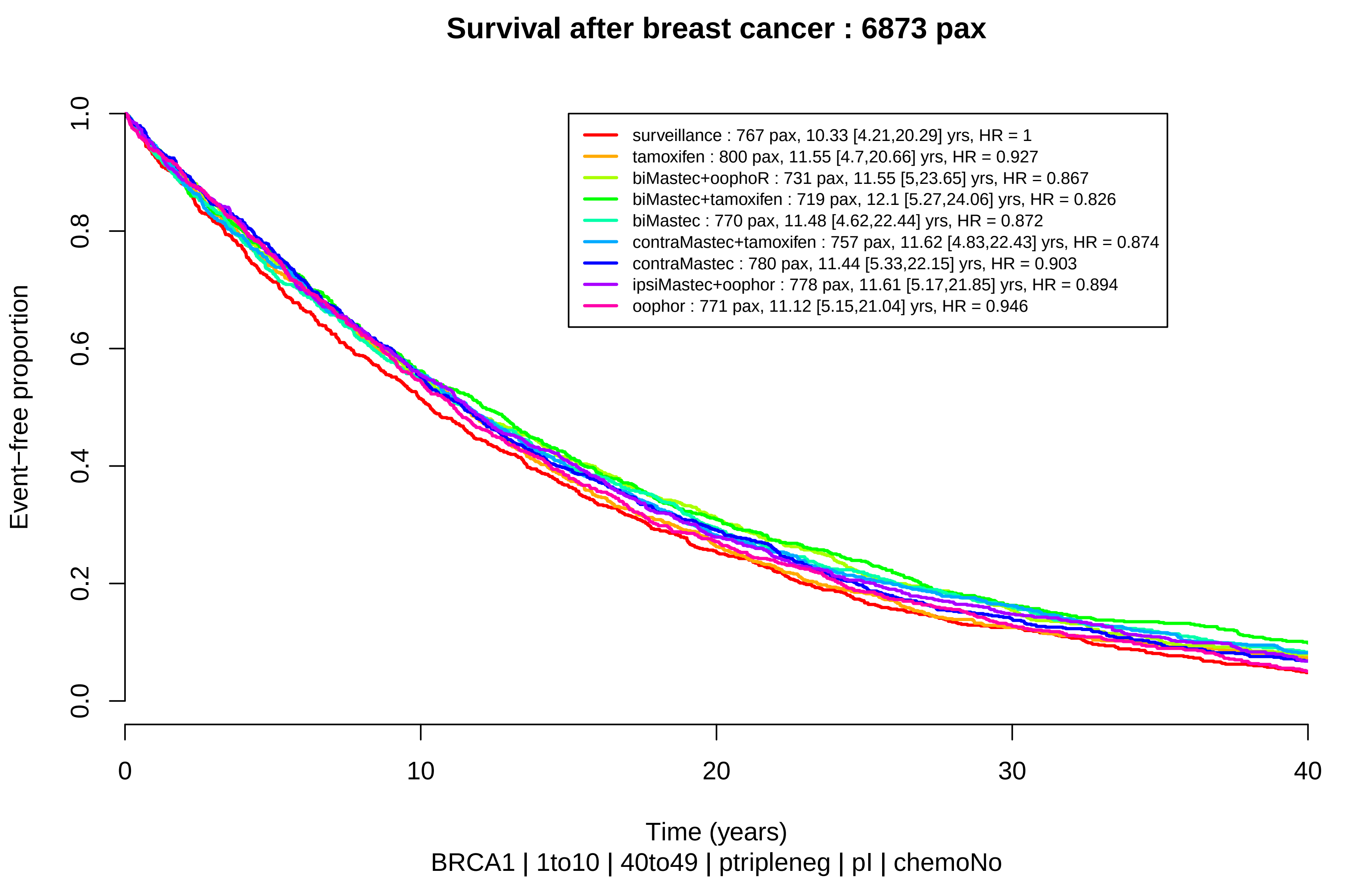

Survival after breast cancer : 6873 pax
surveillance : 767 pax, 10.33 [4.21,20.29] yrs, HR = 1
tamoxifen : 800 pax, 11.55 [4.7,20.66] yrs, HR = 0.927
biMastec+oophoR : 731 pax, 11.55 [5,23.65] yrs, HR = 0.867
biMastec+tamoxifen : 719 pax, 12.1 [5.27,24.06] yrs, HR = 0.826
biMastec : 770 pax, 11.48 [4.62,22.44] yrs, HR = 0.872
contraMastec+tamoxifen : 757 pax, 11.62 [4.83,22.43] yrs, HR = 0.874
contraMastec : 780 pax, 11.44 [5.33,22.15] yrs, HR = 0.903
ipsiMastec+oophor : 778 pax, 11.61 [5.17,21.85] yrs, HR = 0.894
oophor : 771 pax, 11.12 [5.15,21.04] yrs, HR = 0.946
Event−free proportion
0.0
0.2
0.4
0.6
0.8
1.0
0
10
20
30
40
Time (years)
BRCA1 | 1to10 | 40to49 | ptripleneg | pl | chemoNo

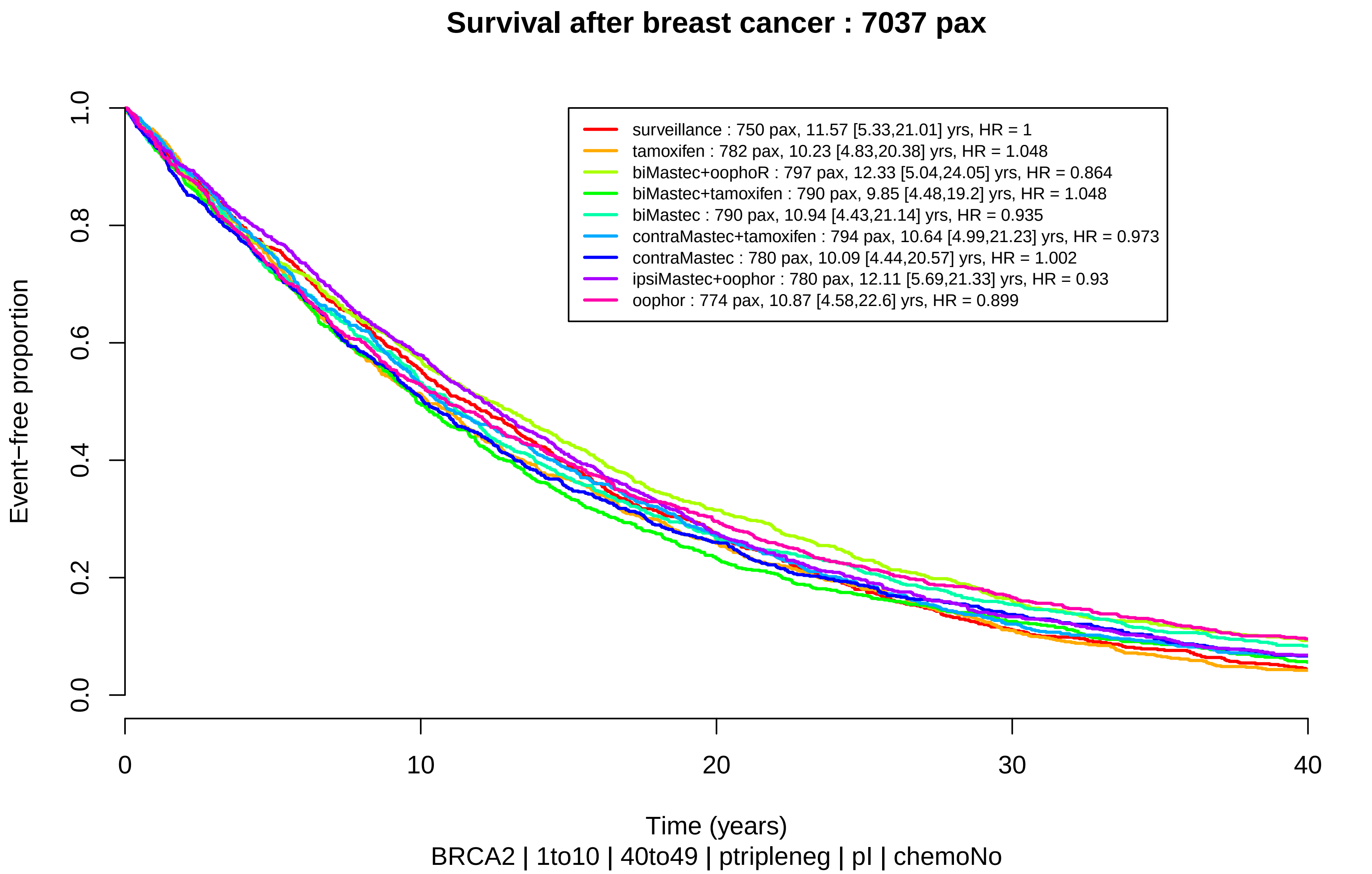

Survival after breast cancer : 7037 pax
surveillance : 750 pax, 11.57 [5.33,21.01] yrs, HR = 1
tamoxifen : 782 pax, 10.23 [4.83,20.38] yrs, HR = 1.048
biMastec+oophoR : 797 pax, 12.33 [5.04,24.05] yrs, HR = 0.864
biMastec+tamoxifen : 790 pax, 9.85 [4.48,19.2] yrs, HR = 1.048
biMastec : 790 pax, 10.94 [4.43,21.14] yrs, HR = 0.935
contraMastec+tamoxifen : 794 pax, 10.64 [4.99,21.23] yrs, HR = 0.973
contraMastec : 780 pax, 10.09 [4.44,20.57] yrs, HR = 1.002
ipsiMastec+oophor : 780 pax, 12.11 [5.69,21.33] yrs, HR = 0.93
oophor : 774 pax, 10.87 [4.58,22.6] yrs, HR = 0.899
Event−free proportion
0.0
0.2
0.4
0.6
0.8
1.0
0
10
20
30
40
Time (years)
BRCA2 | 1to10 | 40to49 | ptripleneg | pl | chemoNo

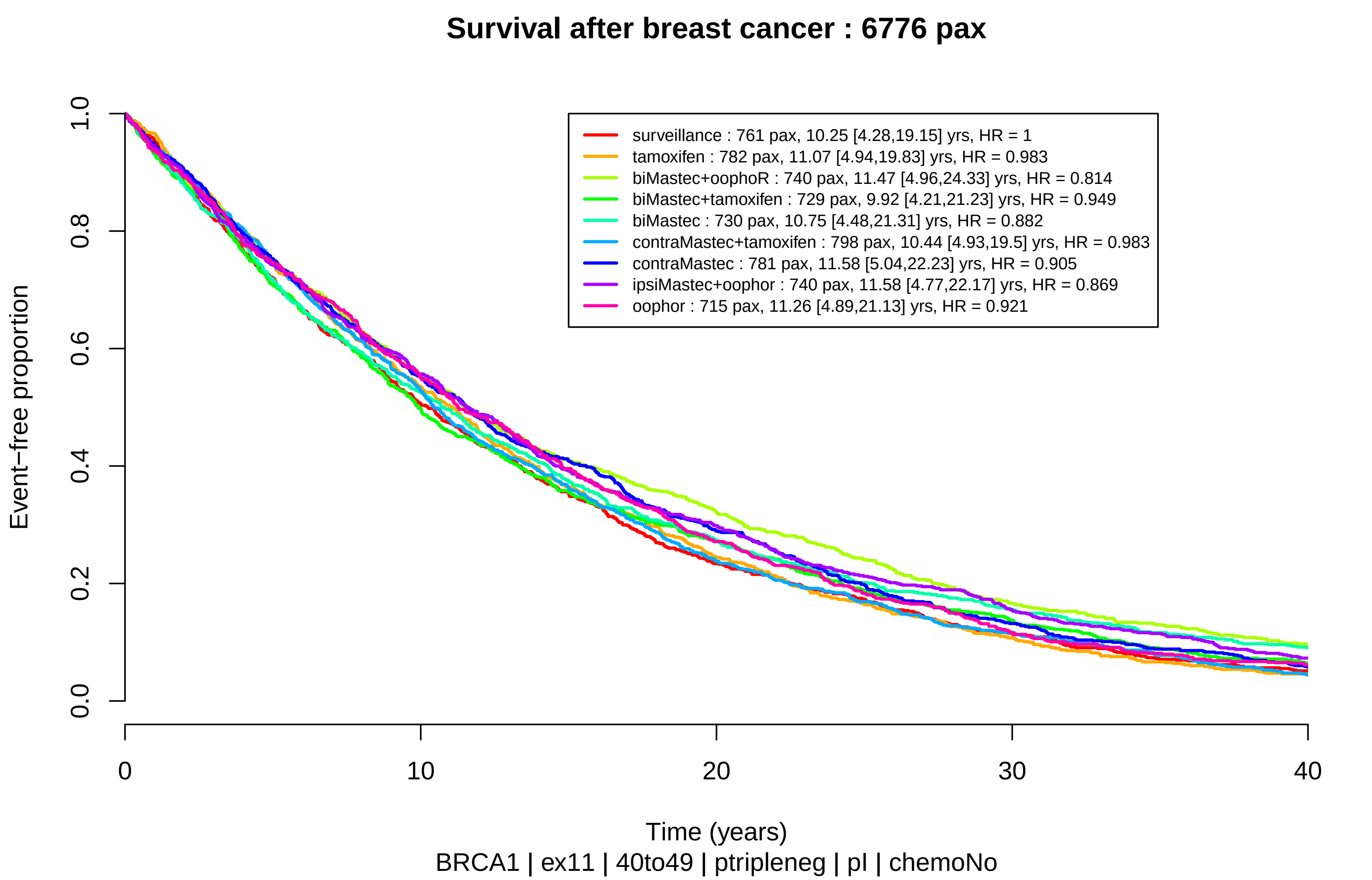

Survival after breast cancer : 6776 pax
surveillance : 761 pax, 10.25 [4.28,19.15] yrs, HR = 1
tamoxifen : 782 pax, 11.07 [4.94,19.83] yrs, HR = 0.983
biMastec+oophoR : 740 pax, 11.47 [4.96,24.33] yrs, HR = 0.814
biMastec+tamoxifen : 729 pax, 9.92 [4.21,21.23] yrs, HR = 0.949
biMastec : 730 pax, 10.75 [4.48,21.31] yrs, HR = 0.882
contraMastec+tamoxifen : 798 pax, 10.44 [4.93,19.5] yrs, HR = 0.983
contraMastec : 781 pax, 11.58 [5.04,22.23] yrs, HR = 0.905
ipsiMastec+oophor : 740 pax, 11.58 [4.77,22.17] yrs, HR = 0.869
oophor : 715 pax, 11.26 [4.89,21.13] yrs, HR = 0.921
Event−free proportion
0.0
0.2
0.4
0.6
0.8
1.0
0
10
20
30
40
Time (years)
BRCA1 | ex11 | 40to49 | ptripleneg | pl | chemoNo

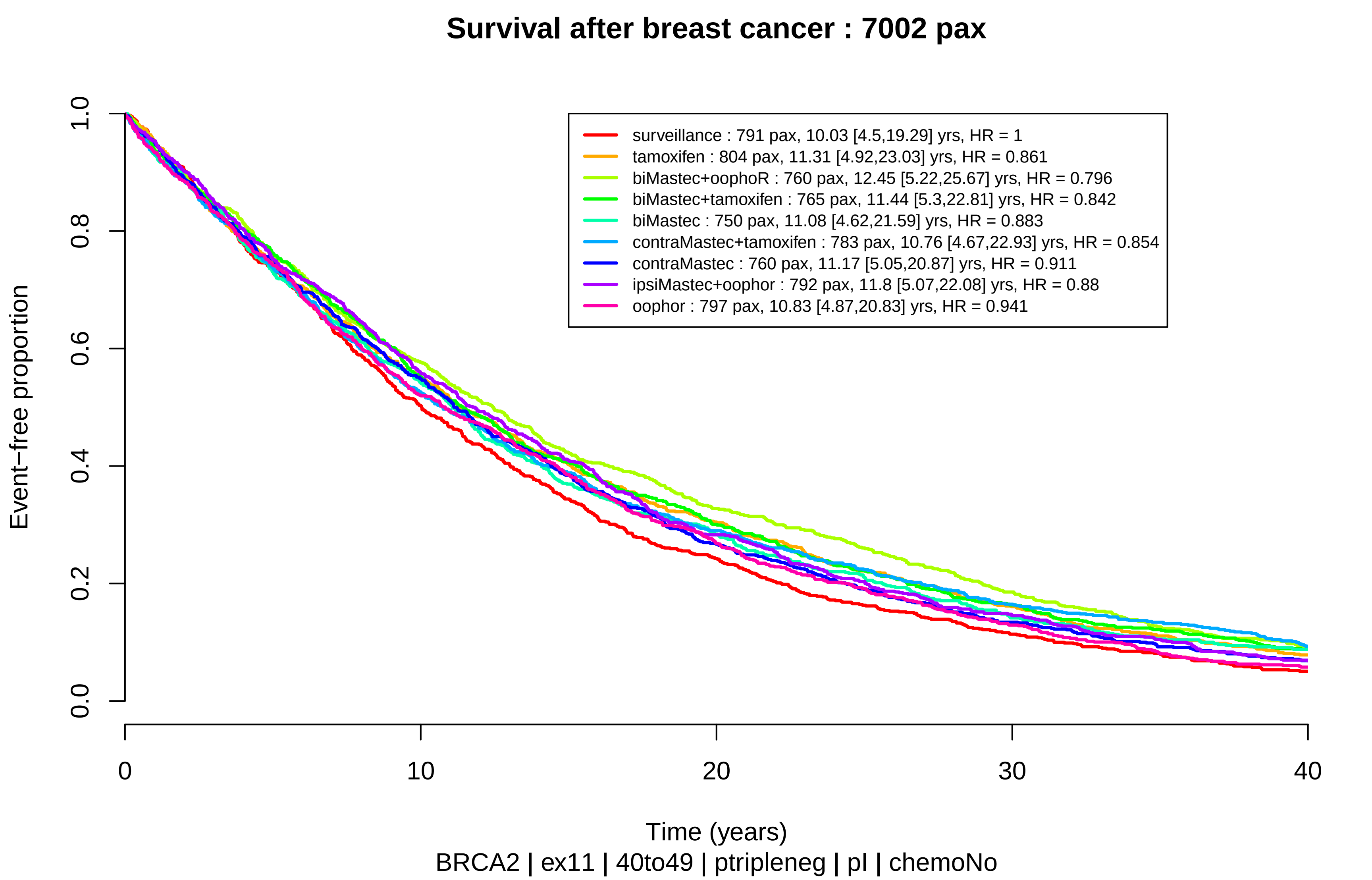

Survival after breast cancer : 7002 pax
surveillance : 791 pax, 10.03 [4.5,19.29] yrs, HR = 1
tamoxifen : 804 pax, 11.31 [4.92,23.03] yrs, HR = 0.861
biMastec+oophoR : 760 pax, 12.45 [5.22,25.67] yrs, HR = 0.796
biMastec+tamoxifen : 765 pax, 11.44 [5.3,22.81] yrs, HR = 0.842
biMastec : 750 pax, 11.08 [4.62,21.59] yrs, HR = 0.883
contraMastec+tamoxifen : 783 pax, 10.76 [4.67,22.93] yrs, HR = 0.854
contraMastec : 760 pax, 11.17 [5.05,20.87] yrs, HR = 0.911
ipsiMastec+oophor : 792 pax, 11.8 [5.07,22.08] yrs, HR = 0.88
oophor : 797 pax, 10.83 [4.87,20.83] yrs, HR = 0.941
Event−free proportion
0.0
0.2
0.4
0.6
0.8
1.0
0
10
20
30
40
Time (years)
BRCA2 | ex11 | 40to49 | ptripleneg | pI | chemoNo

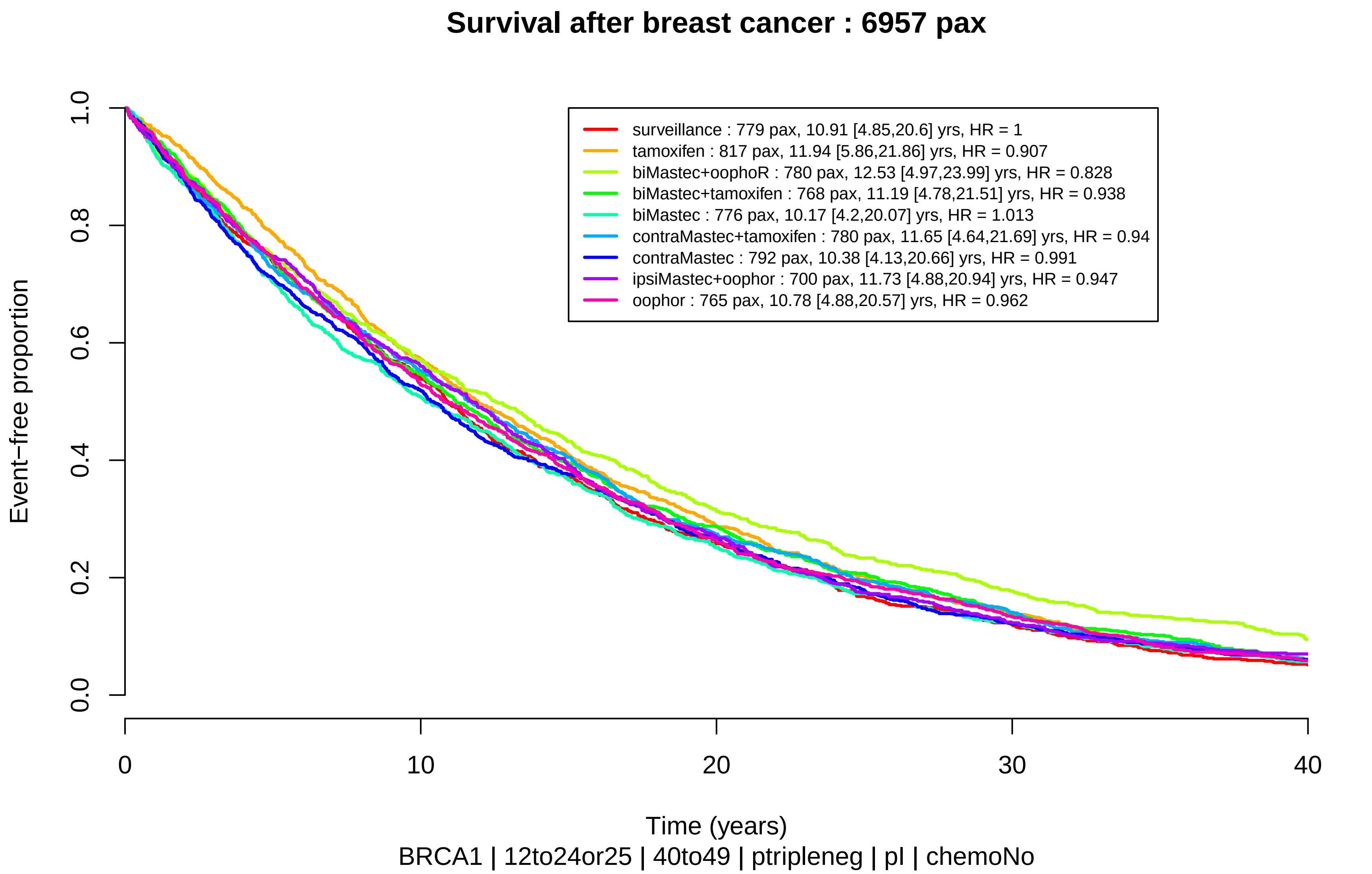

Survival after breast cancer : 6957 pax
Event−free proportion
0.0
0.2
0.4
0.6
0.8
1.0
surveillance : 779 pax, 10.91 [4.85,20.6] yrs, HR = 1
tamoxifen : 817 pax, 11.94 [5.86,21.86] yrs, HR = 0.907
biMastec+oophoR : 780 pax, 12.53 [4.97,23.99] yrs, HR = 0.828
biMastec+tamoxifen : 768 pax, 11.19 [4.78,21.51] yrs, HR = 0.938
biMastec : 776 pax, 10.17 [4.2,20.07] yrs, HR = 1.013
contraMastec+tamoxifen : 780 pax, 11.65 [4.64,21.69] yrs, HR = 0.94
contraMastec : 792 pax, 10.38 [4.13,20.66] yrs, HR = 0.991
ipsiMastec+oophor : 700 pax, 11.73 [4.88,20.94] yrs, HR = 0.947
oophor : 765 pax, 10.78 [4.88,20.57] yrs, HR = 0.962
0
10
20
30
40
Time (years)
BRCA1 | 12to24or25 | 40to49 | ptripleneg | pl | chemoNo

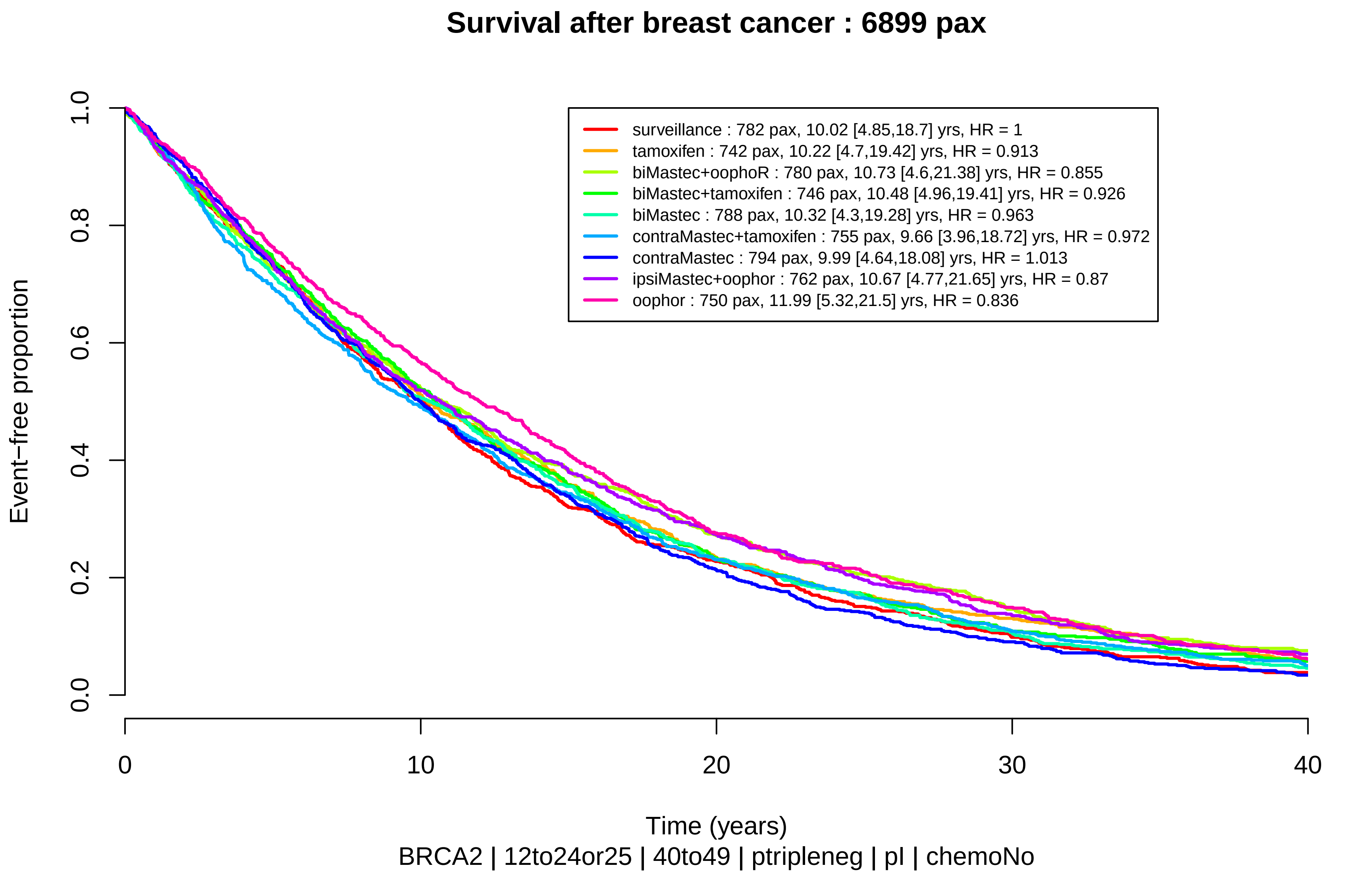

Survival after breast cancer : 6899 pax
Event−free proportion
0.0
0.2
0.4
0.6
0.8
1.0
0
10
20
30
40
Time (years)
BRCA2 | 12to24or25 | 40to49 | ptripleneg | pl | chemoNo
surveillance : 782 pax, 10.02 [4.85,18.7] yrs, HR = 1
tamoxifen : 742 pax, 10.22 [4.7,19.42] yrs, HR = 0.913
biMastec+oophoR : 780 pax, 10.73 [4.6,21.38] yrs, HR = 0.855
biMastec+tamoxifen : 746 pax, 10.48 [4.96,19.41] yrs, HR = 0.926
biMastec : 788 pax, 10.32 [4.3,19.28] yrs, HR = 0.963
contraMastec+tamoxifen : 755 pax, 9.66 [3.96,18.72] yrs, HR = 0.972
contraMastec : 794 pax, 9.99 [4.64,18.08] yrs, HR = 1.013
ipsiMastec+oophor : 762 pax, 10.67 [4.77,21.65] yrs, HR = 0.87
oophor : 750 pax, 11.99 [5.32,21.5] yrs, HR = 0.836

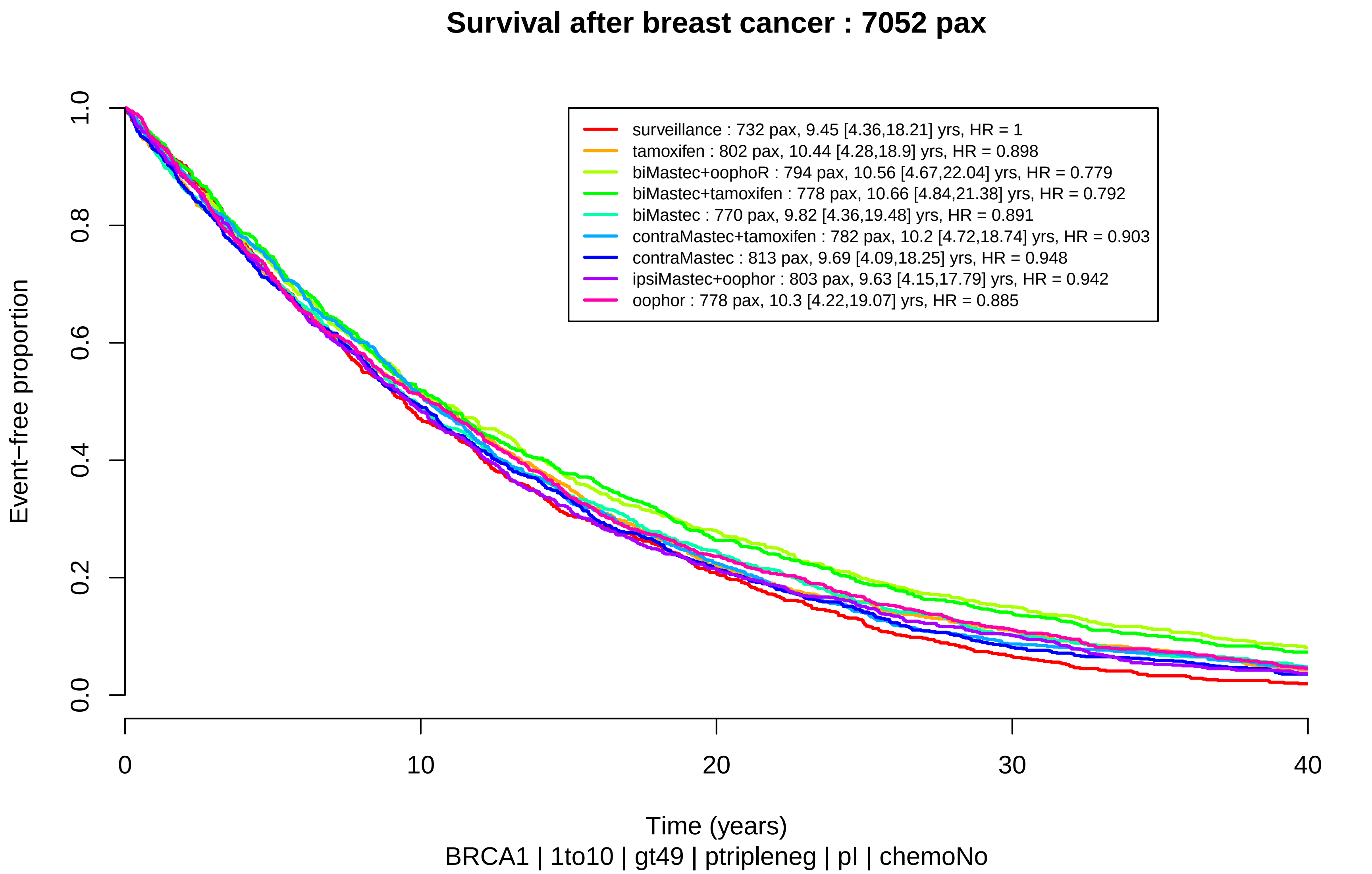

Survival after breast cancer : 7052 pax
surveillance : 732 pax, 9.45 [4.36,18.21] yrs, HR = 1
tamoxifen : 802 pax, 10.44 [4.28,18.9] yrs, HR = 0.898
biMastec+oophoR : 794 pax, 10.56 [4.67,22.04] yrs, HR = 0.779
biMastec+tamoxifen : 778 pax, 10.66 [4.84,21.38] yrs, HR = 0.792
biMastec : 770 pax, 9.82 [4.36,19.48] yrs, HR = 0.891
contraMastec+tamoxifen : 782 pax, 10.2 [4.72,18.74] yrs, HR = 0.903
contraMastec : 813 pax, 9.69 [4.09,18.25] yrs, HR = 0.948
ipsiMastec+oophor : 803 pax, 9.63 [4.15,17.79] yrs, HR = 0.942
oophor : 778 pax, 10.3 [4.22,19.07] yrs, HR = 0.885
Event−free proportion
0.0
0.2
0.4
0.6
0.8
1.0
0
10
20
30
40
Time (years)
BRCA1 | 1to10 | gt49 | ptripleneg | pl | chemoNo

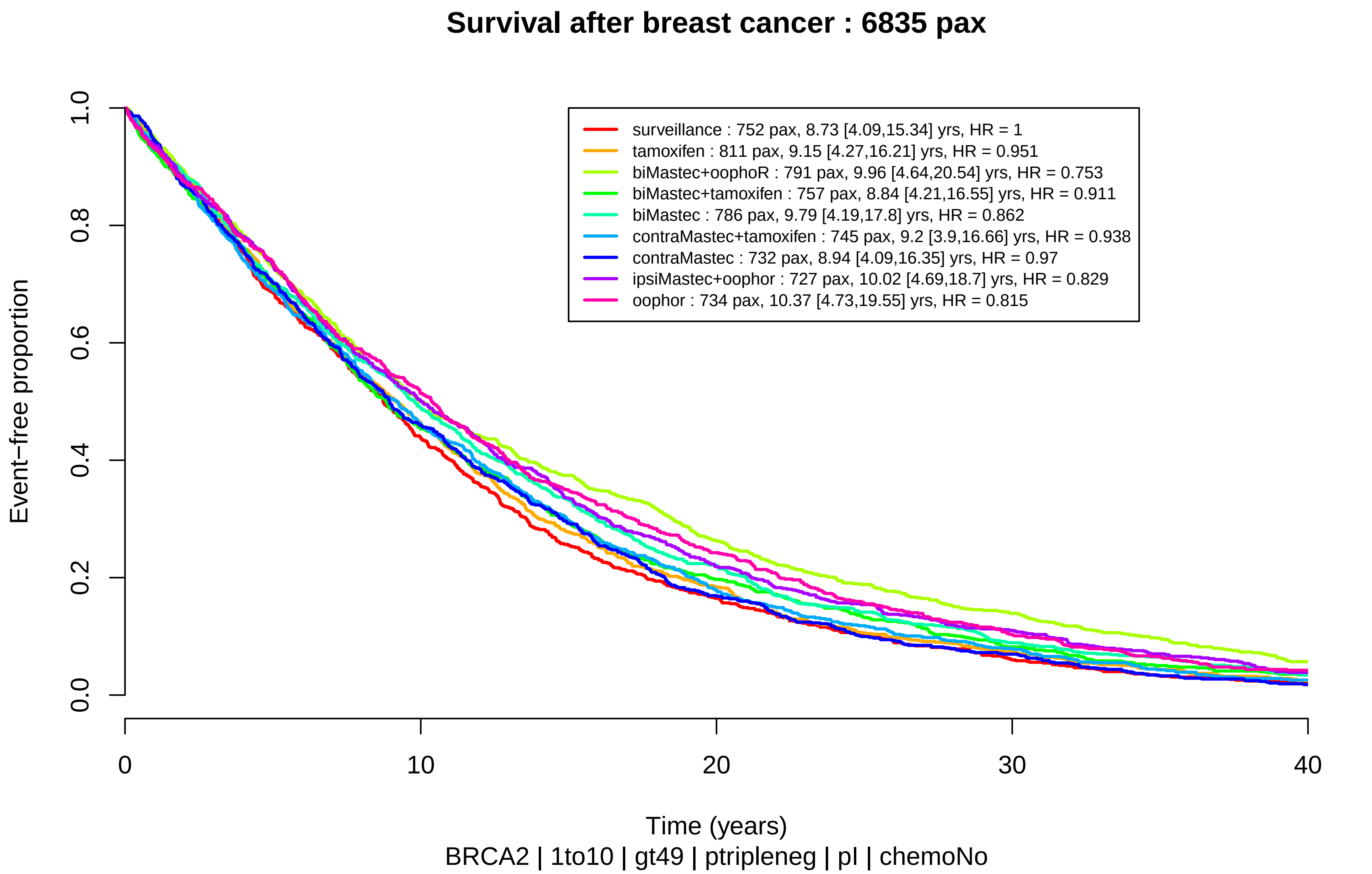
Survival after breast cancer : 6835 pax
surveillance : 752 pax, 8.73 [4.09,15.34] yrs, HR = 1
tamoxifen : 811 pax, 9.15 [4.27,16.21] yrs, HR = 0.951
biMastec+oophoR : 791 pax, 9.96 [4.64,20.54] yrs, HR = 0.753
biMastec+tamoxifen : 757 pax, 8.84 [4.21,16.55] yrs, HR = 0.911
biMastec : 786 pax, 9.79 [4.19,17.8] yrs, HR = 0.862
contraMastec+tamoxifen : 745 pax, 9.2 [3.9,16.66] yrs, HR = 0.938
contraMastec : 732 pax, 8.94 [4.09,16.35] yrs, HR = 0.97
ipsiMastec+oophor : 727 pax, 10.02 [4.69,18.7] yrs, HR = 0.829
oophor : 734 pax, 10.37 [4.73,19.55] yrs, HR = 0.815
Event−free proportion
0.0
0.2
0.4
0.6
0.8
1.0
0
10
20
30
40
Time (years)
BRCA2 | 1to10 | gt49 | ptripleneg | pl | chemoNo

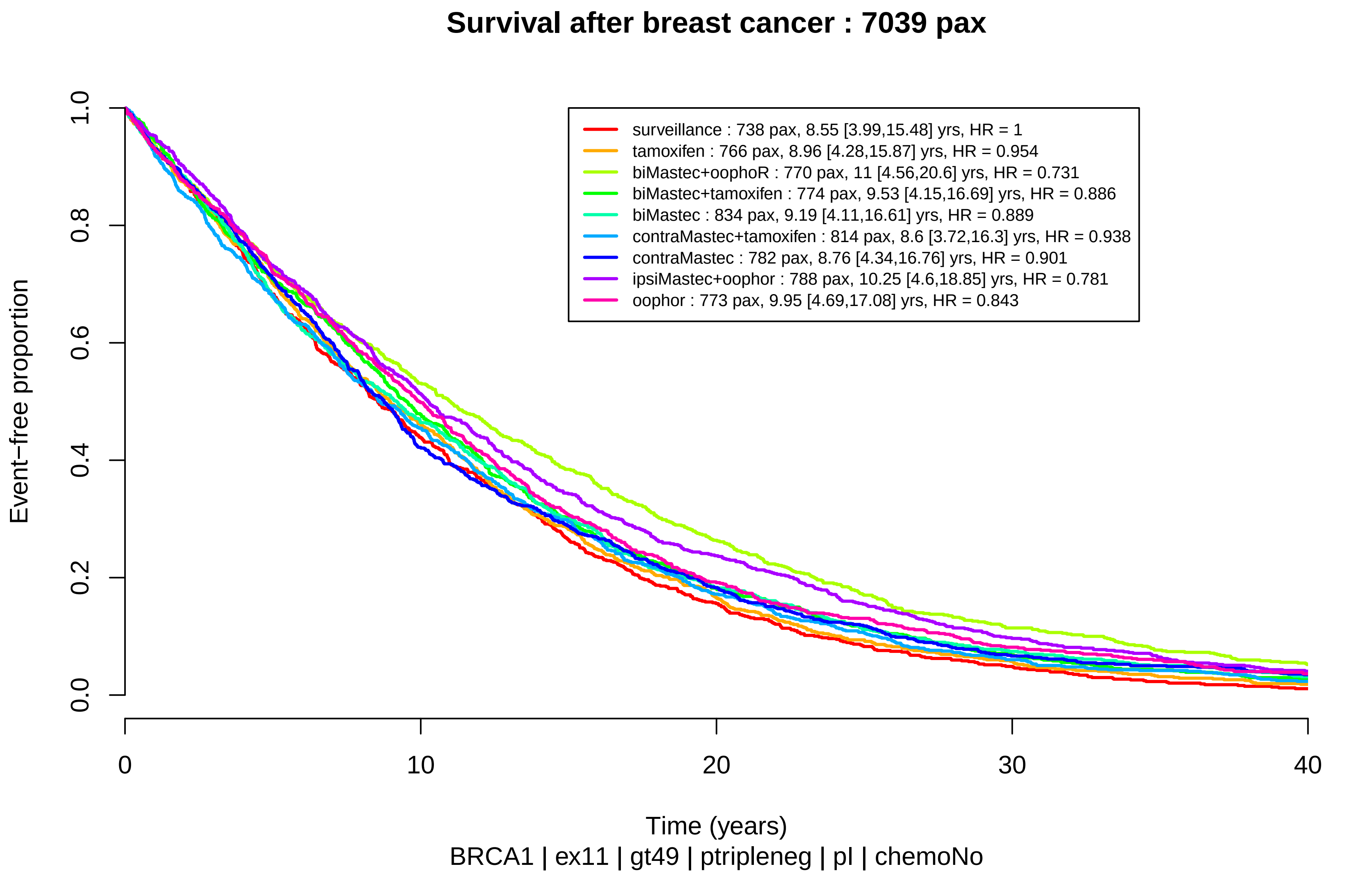

Survival after breast cancer : 7039 pax
surveillance : 738 pax, 8.55 [3.99,15.48] yrs, HR = 1
tamoxifen : 766 pax, 8.96 [4.28,15.87] yrs, HR = 0.954
biMastec+oophoR : 770 pax, 11 [4.56,20.6] yrs, HR = 0.731
biMastec+tamoxifen : 774 pax, 9.53 [4.15,16.69] yrs, HR = 0.886
biMastec : 834 pax, 9.19 [4.11,16.61] yrs, HR = 0.889
contraMastec+tamoxifen : 814 pax, 8.6 [3.72,16.3] yrs, HR = 0.938
contraMastec : 782 pax, 8.76 [4.34,16.76] yrs, HR = 0.901
ipsiMastec+oophor : 788 pax, 10.25 [4.6,18.85] yrs, HR = 0.781
oophor : 773 pax, 9.95 [4.69,17.08] yrs, HR = 0.843
Event−free proportion
0.0
0.2
0.4
0.6
0.8
1.0
0
10
20
30
40
Time (years)
BRCA1 | ex11 | gt49 | ptripleneg | pl | chemoNo

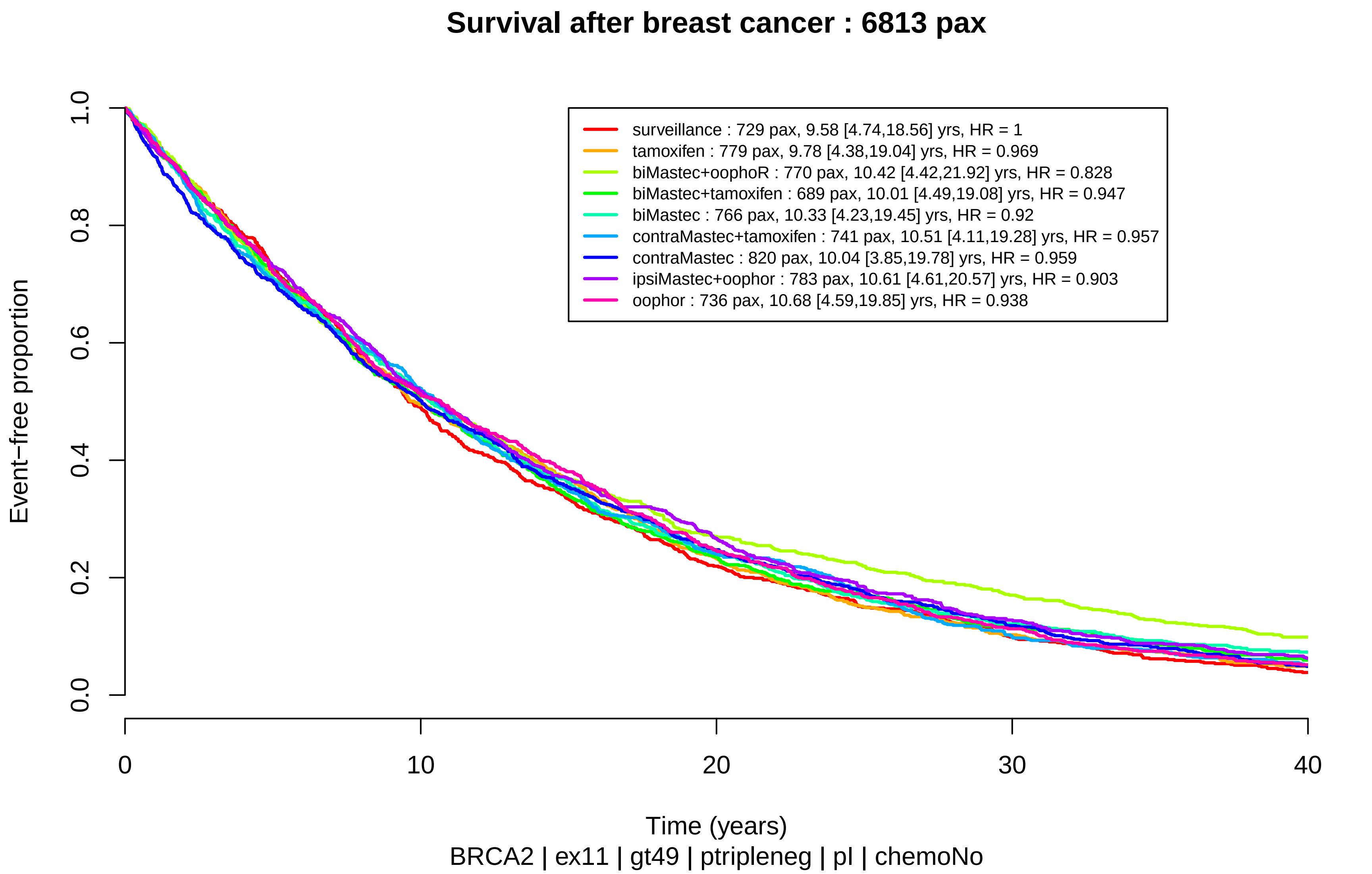

Survival after breast cancer : 6813 pax
surveillance : 729 pax, 9.58 [4.74,18.56] yrs, HR = 1
tamoxifen : 779 pax, 9.78 [4.38,19.04] yrs, HR = 0.969
biMastec+oophoR : 770 pax, 10.42 [4.42,21.92] yrs, HR = 0.828
biMastec+tamoxifen : 689 pax, 10.01 [4.49,19.08] yrs, HR = 0.947
biMastec : 766 pax, 10.33 [4.23,19.45] yrs, HR = 0.92
contraMastec+tamoxifen : 741 pax, 10.51 [4.11,19.28] yrs, HR = 0.957
contraMastec : 820 pax, 10.04 [3.85,19.78] yrs, HR = 0.959
ipsiMastec+oophor : 783 pax, 10.61 [4.61,20.57] yrs, HR = 0.903
oophor : 736 pax, 10.68 [4.59,19.85] yrs, HR = 0.938
Event−free proportion
0.0
0.2
0.4
0.6
0.8
1.0
0
10
20
30
40
Time (years)
BRCA2 | ex11 | gt49 | ptripleneg | pl | chemoNo

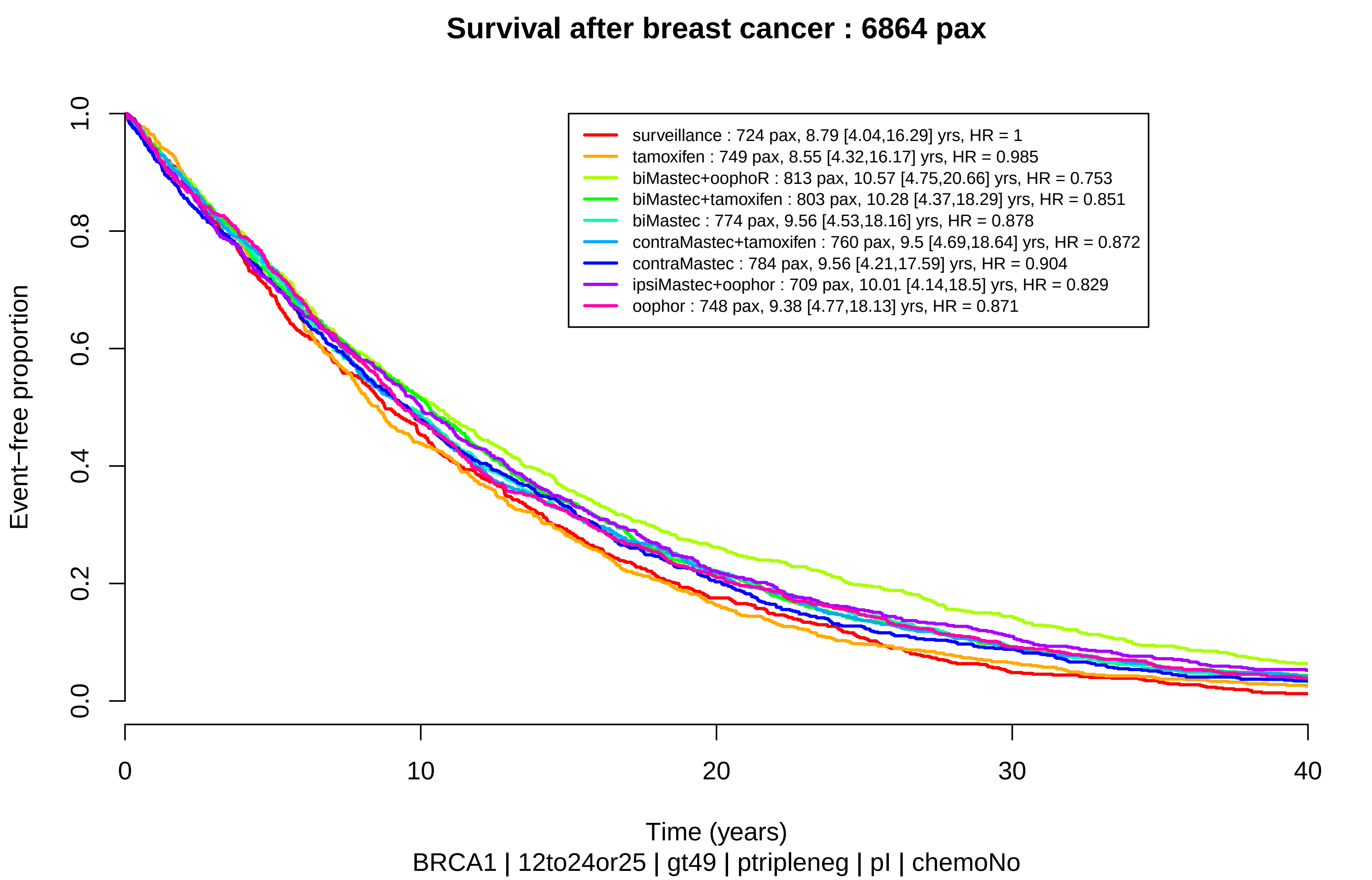
Survival after breast cancer : 6864 pax
Event−free proportion
1.0
0.8
0.6
0.4
0.2
0.0
0
10
20
30
40
Time (years)
BRCA1 | 12to24or25 | gt49 | ptripleneg | pl | chemoNo
surveillance : 724 pax, 8.79 [4.04,16.29] yrs, HR = 1
tamoxifen : 749 pax, 8.55 [4.32,16.17] yrs, HR = 0.985
biMastec+oophoR : 813 pax, 10.57 [4.75,20.66] yrs, HR = 0.753
biMastec+tamoxifen : 803 pax, 10.28 [4.37,18.29] yrs, HR = 0.851
biMastec : 774 pax, 9.56 [4.53,18.16] yrs, HR = 0.878
contraMastec+tamoxifen : 760 pax, 9.5 [4.69,18.64] yrs, HR = 0.872
contraMastec : 784 pax, 9.56 [4.21,17.59] yrs, HR = 0.904
ipsiMastec+oophor : 709 pax, 10.01 [4.14,18.5] yrs, HR = 0.829
oophor : 748 pax, 9.38 [4.77,18.13] yrs, HR = 0.871

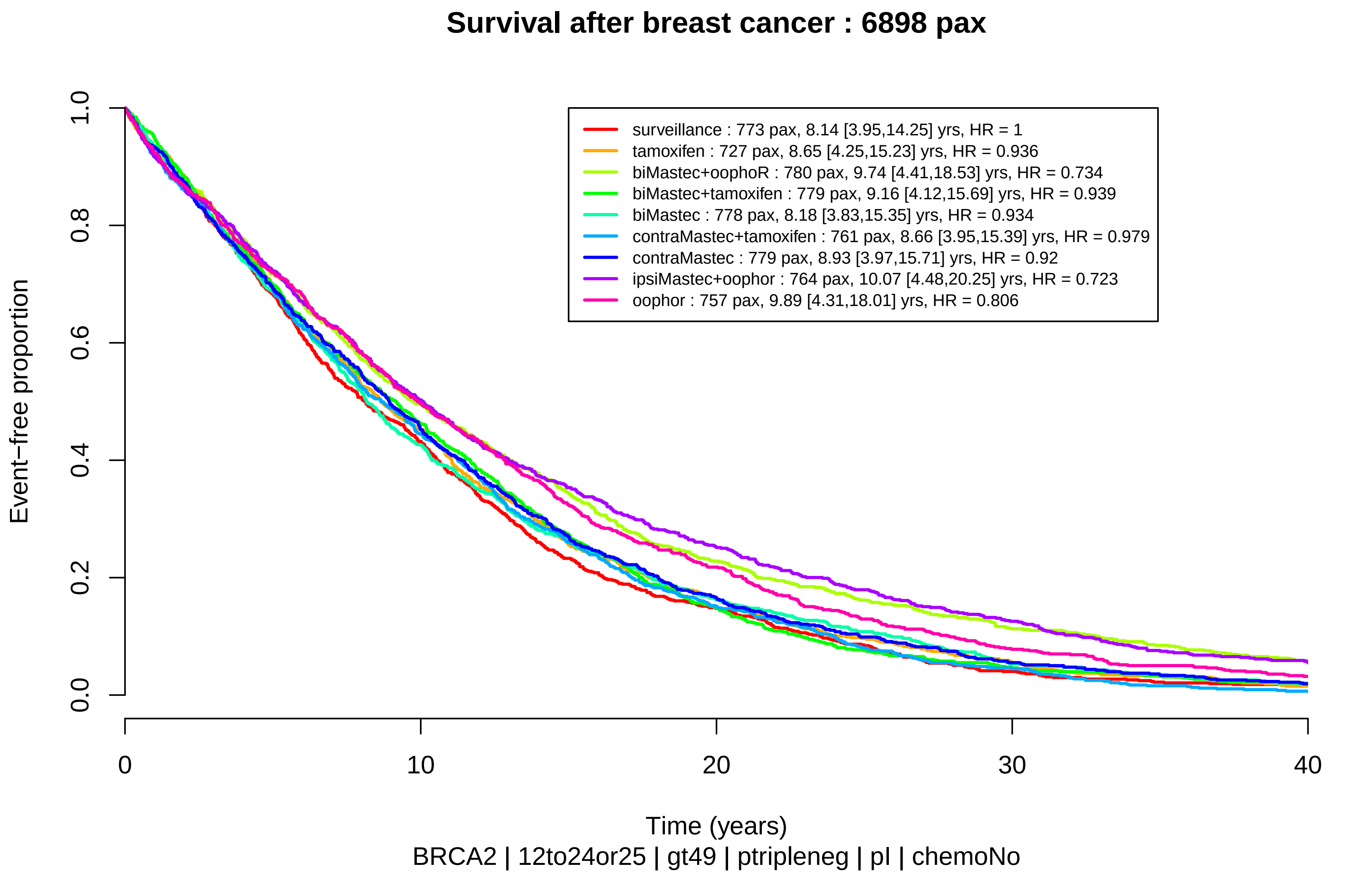

Survival after breast cancer : 6898 pax
surveillance : 773 pax, 8.14 [3.95,14.25] yrs, HR = 1
tamoxifen : 727 pax, 8.65 [4.25,15.23] yrs, HR = 0.936
biMastec+oophoR : 780 pax, 9.74 [4.41,18.53] yrs, HR = 0.734
biMastec+tamoxifen : 779 pax, 9.16 [4.12,15.69] yrs, HR = 0.939
biMastec : 778 pax, 8.18 [3.83,15.35] yrs, HR = 0.934
contraMastec+tamoxifen : 761 pax, 8.66 [3.95,15.39] yrs, HR = 0.979
contraMastec : 779 pax, 8.93 [3.97,15.71] yrs, HR = 0.92
ipsiMastec+oophor : 764 pax, 10.07 [4.48,20.25] yrs, HR = 0.723
oophor : 757 pax, 9.89 [4.31,18.01] yrs, HR = 0.806
Event−free proportion
0.0
0.2
0.4
0.6
0.8
1.0
0
10
20
30
40
Time (years)
BRCA2 | 12to24or25 | gt49 | ptripleneg | pI | chemoNo

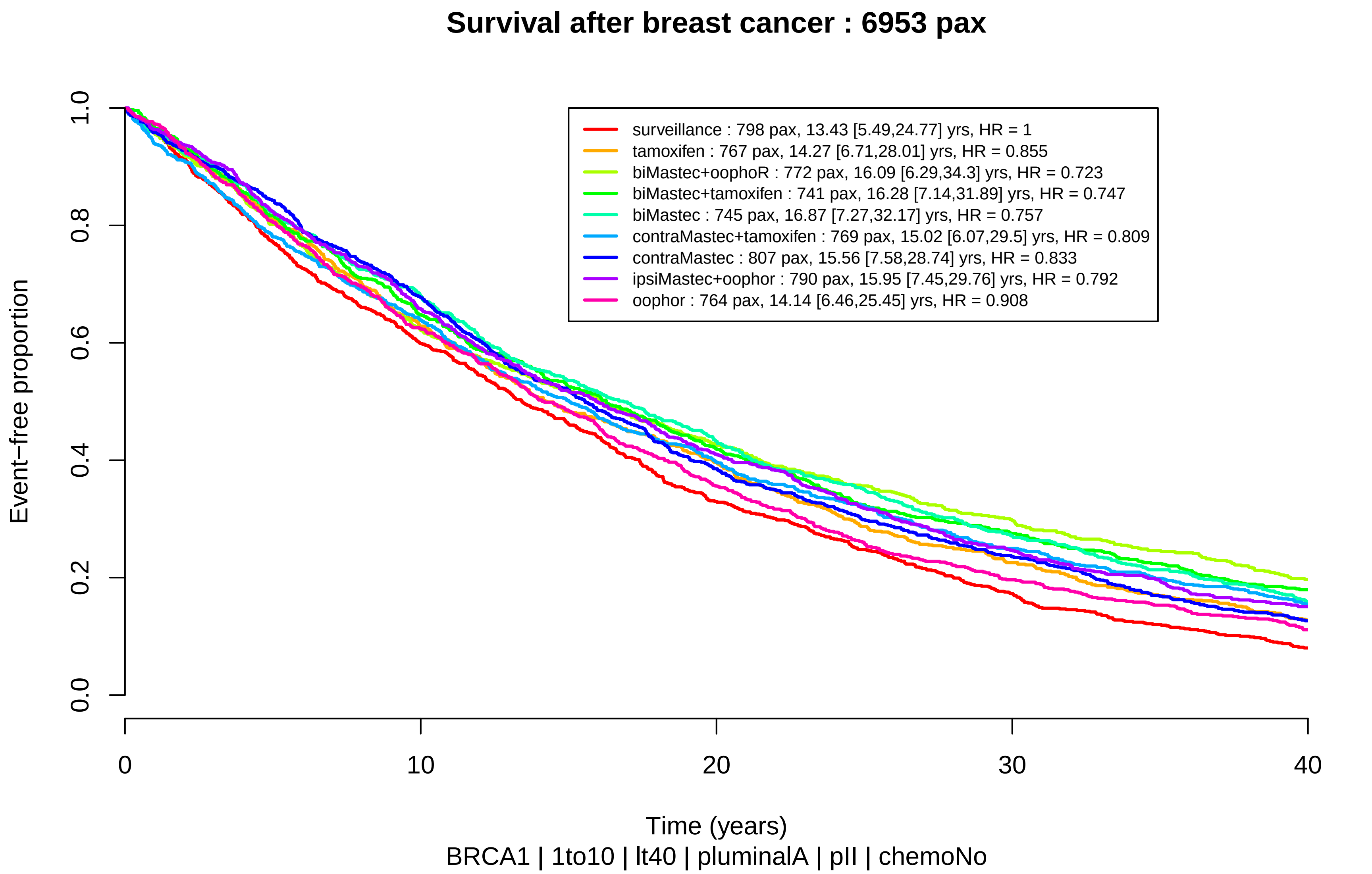

Survival after breast cancer : 6953 pax
surveillance : 798 pax, 13.43 [5.49,24.77] yrs, HR = 1
tamoxifen : 767 pax, 14.27 [6.71,28.01] yrs, HR = 0.855
biMastec+oophoR : 772 pax, 16.09 [6.29,34.3] yrs, HR = 0.723
biMastec+tamoxifen : 741 pax, 16.28 [7.14,31.89] yrs, HR = 0.747
biMastec : 745 pax, 16.87 [7.27,32.17] yrs, HR = 0.757
contraMastec+tamoxifen : 769 pax, 15.02 [6.07,29.5] yrs, HR = 0.809
contraMastec : 807 pax, 15.56 [7.58,28.74] yrs, HR = 0.833
ipsiMastec+oophor : 790 pax, 15.95 [7.45,29.76] yrs, HR = 0.792
oophor : 764 pax, 14.14 [6.46,25.45] yrs, HR = 0.908
Event−free proportion
0.0
0.2
0.4
0.6
0.8
1.0
0
10
20
30
40
Time (years)
BRCA1 | 1to10 | lt40 | pluminalA | pII | chemoNo

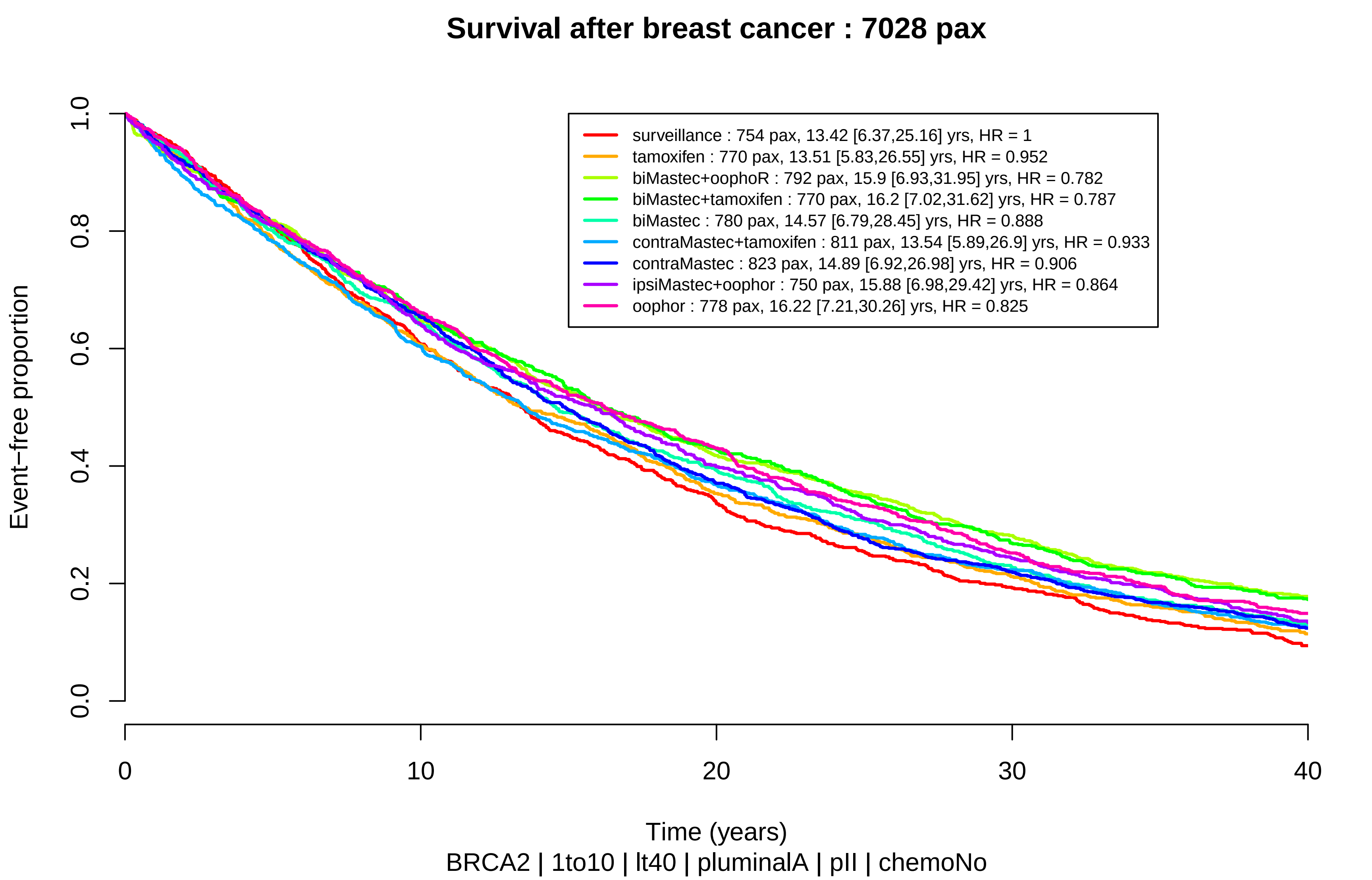

Survival after breast cancer : 7028 pax
surveillance : 754 pax, 13.42 [6.37,25.16] yrs, HR = 1
tamoxifen : 770 pax, 13.51 [5.83,26.55] yrs, HR = 0.952
biMastec+oophoR : 792 pax, 15.9 [6.93,31.95] yrs, HR = 0.782
biMastec+tamoxifen : 770 pax, 16.2 [7.02,31.62] yrs, HR = 0.787
biMastec : 780 pax, 14.57 [6.79,28.45] yrs, HR = 0.888
contraMastec+tamoxifen : 811 pax, 13.54 [5.89,26.9] yrs, HR = 0.933
contraMastec : 823 pax, 14.89 [6.92,26.98] yrs, HR = 0.906
ipsiMastec+oophor : 750 pax, 15.88 [6.98,29.42] yrs, HR = 0.864
oophor : 778 pax, 16.22 [7.21,30.26] yrs, HR = 0.825
Event−free proportion
0.0
0.2
0.4
0.6
0.8
1.0
0
10
20
30
40
Time (years)
BRCA2 | 1to10 | lt40 | pluminalA | pII | chemoNo

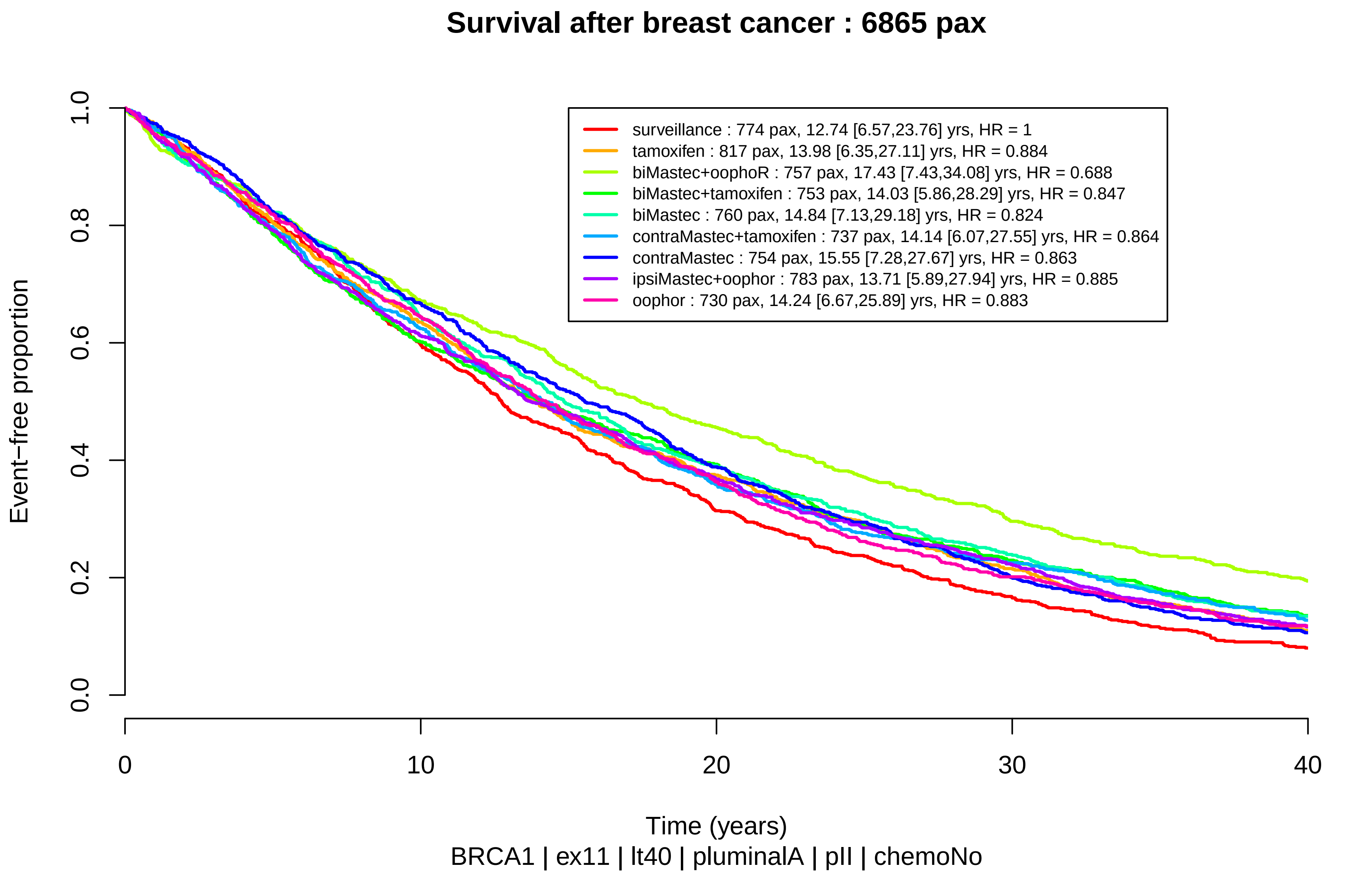

Survival after breast cancer : 6865 pax
surveillance : 774 pax, 12.74 [6.57,23.76] yrs, HR = 1
tamoxifen : 817 pax, 13.98 [6.35,27.11] yrs, HR = 0.884
biMastec+oophoR : 757 pax, 17.43 [7.43,34.08] yrs, HR = 0.688
biMastec+tamoxifen : 753 pax, 14.03 [5.86,28.29] yrs, HR = 0.847
biMastec : 760 pax, 14.84 [7.13,29.18] yrs, HR = 0.824
contraMastec+tamoxifen : 737 pax, 14.14 [6.07,27.55] yrs, HR = 0.864
contraMastec : 754 pax, 15.55 [7.28,27.67] yrs, HR = 0.863
ipsiMastec+oophor : 783 pax, 13.71 [5.89,27.94] yrs, HR = 0.885
oophor : 730 pax, 14.24 [6.67,25.89] yrs, HR = 0.883
Event−free proportion
0.0
0.2
0.4
0.6
0.8
1.0
0
10
20
30
40
Time (years)
BRCA1 | ex11 | lt40 | pluminalA | pII | chemoNo

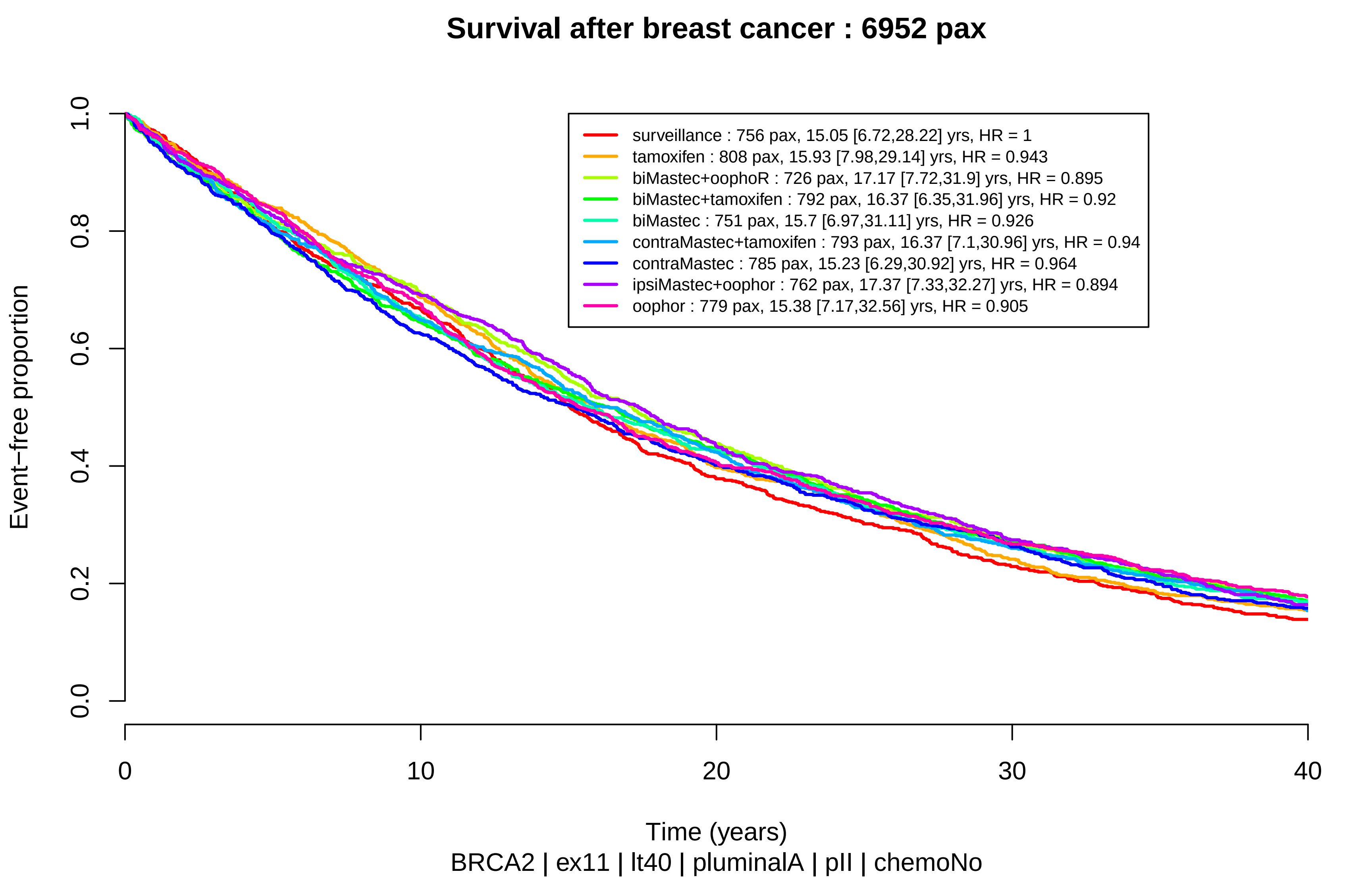

Survival after breast cancer : 6952 pax
Event−free proportion
0.0
0.2
0.4
0.6
0.8
1.0
0
10
20
30
40
Time (years)
surveillance : 756 pax, 15.05 [6.72,28.22] yrs, HR = 1
tamoxifen : 808 pax, 15.93 [7.98,29.14] yrs, HR = 0.943
biMastec+oophoR : 726 pax, 17.17 [7.72,31.9] yrs, HR = 0.895
biMastec+tamoxifen : 792 pax, 16.37 [6.35,31.96] yrs, HR = 0.92
biMastec : 751 pax, 15.7 [6.97,31.11] yrs, HR = 0.926
contraMastec+tamoxifen : 793 pax, 16.37 [7.1,30.96] yrs, HR = 0.94
contraMastec : 785 pax, 15.23 [6.29,30.92] yrs, HR = 0.964
ipsiMastec+oophor : 762 pax, 17.37 [7.33,32.27] yrs, HR = 0.894
oophor : 779 pax, 15.38 [7.17,32.56] yrs, HR = 0.905
BRCA2 | ex11 | lt40 | pluminalA | pII | chemoNo

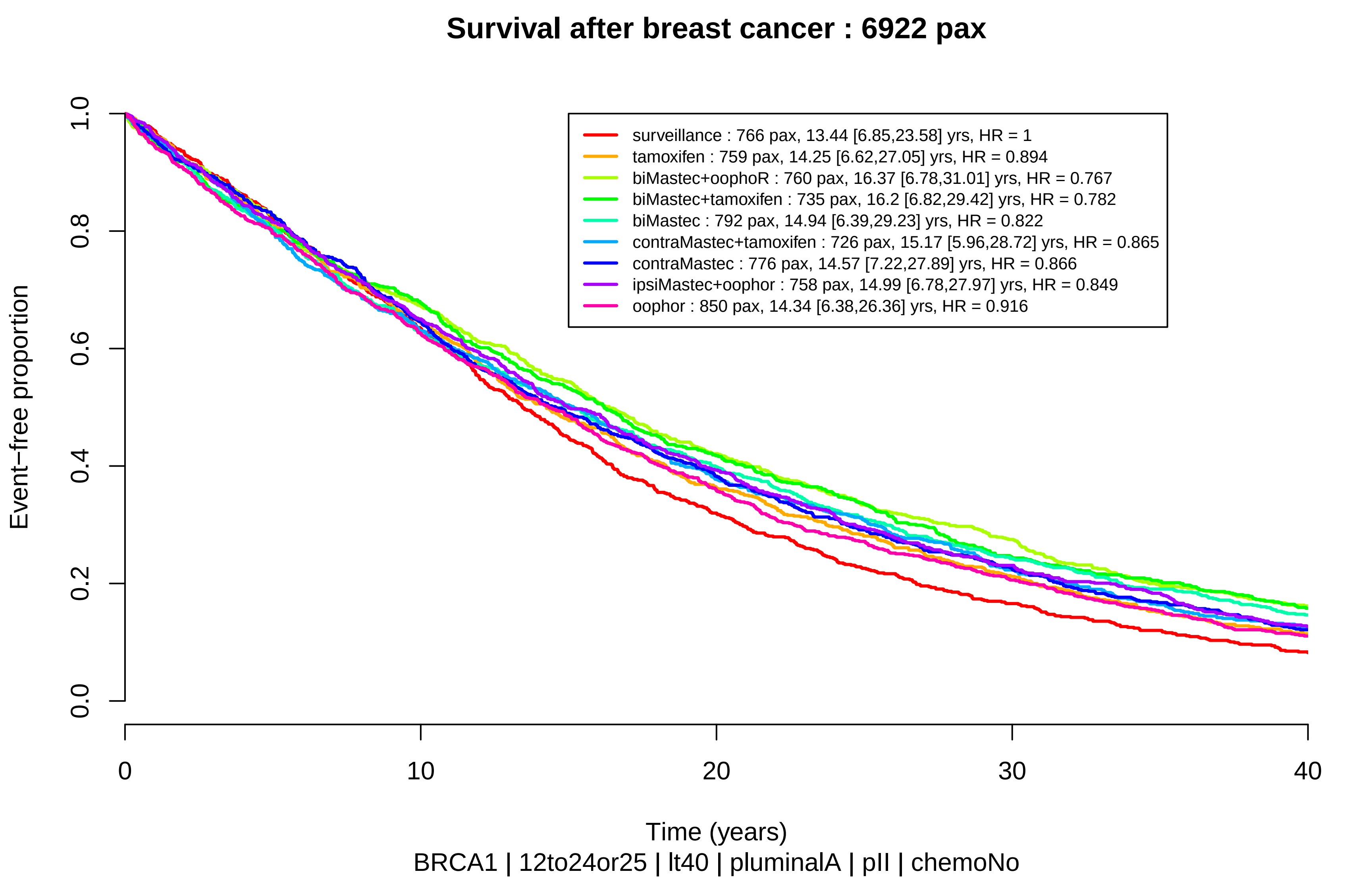

Survival after breast cancer : 6922 pax
surveillance : 766 pax, 13.44 [6.85,23.58] yrs, HR = 1
tamoxifen : 759 pax, 14.25 [6.62,27.05] yrs, HR = 0.894
biMastec+oophoR : 760 pax, 16.37 [6.78,31.01] yrs, HR = 0.767
biMastec+tamoxifen : 735 pax, 16.2 [6.82,29.42] yrs, HR = 0.782
biMastec : 792 pax, 14.94 [6.39,29.23] yrs, HR = 0.822
contraMastec+tamoxifen : 726 pax, 15.17 [5.96,28.72] yrs, HR = 0.865
contraMastec : 776 pax, 14.57 [7.22,27.89] yrs, HR = 0.866
ipsiMastec+oophor : 758 pax, 14.99 [6.78,27.97] yrs, HR = 0.849
oophor : 850 pax, 14.34 [6.38,26.36] yrs, HR = 0.916
Event−free proportion
0.0
0.2
0.4
0.6
0.8
1.0
0
10
20
30
40
Time (years)
BRCA1 | 12to24or25 | lt40 | pluminalA | pII | chemoNo

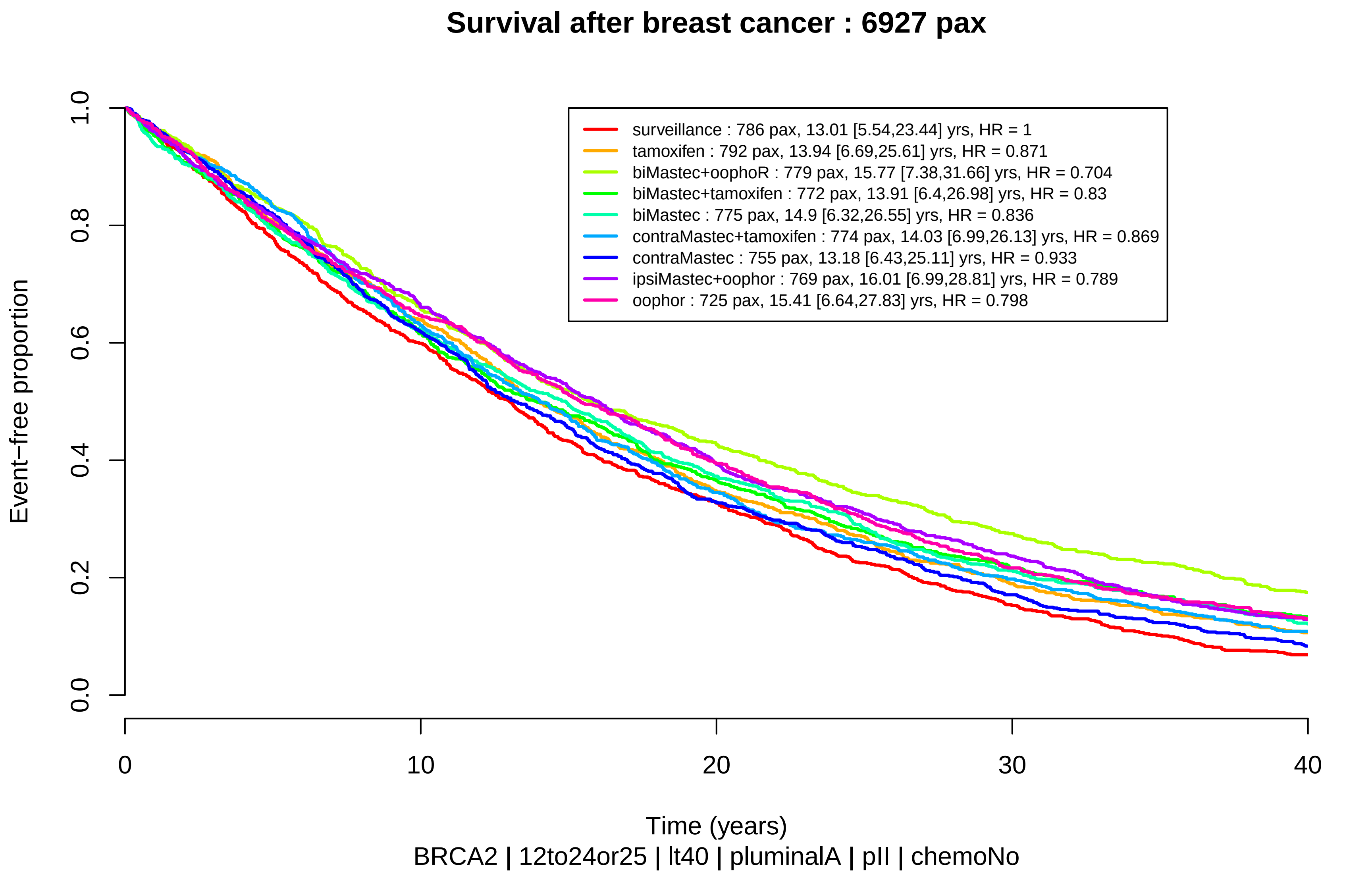
Survival after breast cancer : 6927 pax
surveillance : 786 pax, 13.01 [5.54,23.44] yrs, HR = 1
tamoxifen : 792 pax, 13.94 [6.69,25.61] yrs, HR = 0.871
biMastec+oophoR : 779 pax, 15.77 [7.38,31.66] yrs, HR = 0.704
biMastec+tamoxifen : 772 pax, 13.91 [6.4,26.98] yrs, HR = 0.83
biMastec : 775 pax, 14.9 [6.32,26.55] yrs, HR = 0.836
contraMastec+tamoxifen : 774 pax, 14.03 [6.99,26.13] yrs, HR = 0.869
contraMastec : 755 pax, 13.18 [6.43,25.11] yrs, HR = 0.933
ipsiMastec+oophor : 769 pax, 16.01 [6.99,28.81] yrs, HR = 0.789
oophor : 725 pax, 15.41 [6.64,27.83] yrs, HR = 0.798
Event−free proportion
0.0
0.2
0.4
0.6
0.8
1.0
0
10
20
30
40
Time (years)
BRCA2 | 12to24or25 | lt40 | pluminalA | pII | chemoNo

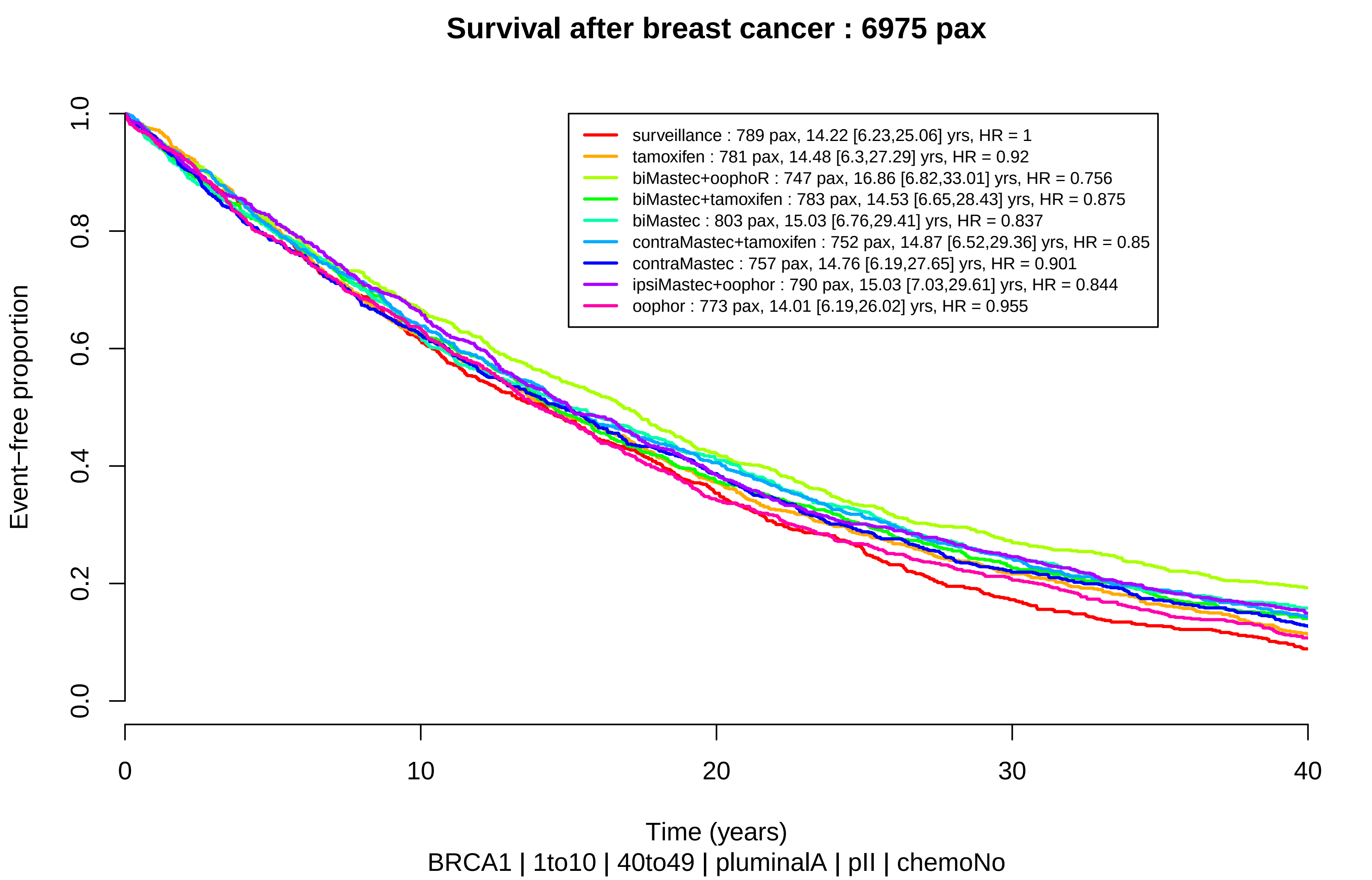

Survival after breast cancer : 6975 pax
surveillance : 789 pax, 14.22 [6.23,25.06] yrs, HR = 1
tamoxifen : 781 pax, 14.48 [6.3,27.29] yrs, HR = 0.92
biMastec+oophoR : 747 pax, 16.86 [6.82,33.01] yrs, HR = 0.756
biMastec+tamoxifen : 783 pax, 14.53 [6.65,28.43] yrs, HR = 0.875
biMastec : 803 pax, 15.03 [6.76,29.41] yrs, HR = 0.837
contraMastec+tamoxifen : 752 pax, 14.87 [6.52,29.36] yrs, HR = 0.85
contraMastec : 757 pax, 14.76 [6.19,27.65] yrs, HR = 0.901
ipsiMastec+oophor : 790 pax, 15.03 [7.03,29.61] yrs, HR = 0.844
oophor : 773 pax, 14.01 [6.19,26.02] yrs, HR = 0.955
Event−free proportion
0.0
0.2
0.4
0.6
0.8
1.0
0
10
20
30
40
Time (years)
BRCA1 | 1to10 | 40to49 | pluminalA | pII | chemoNo

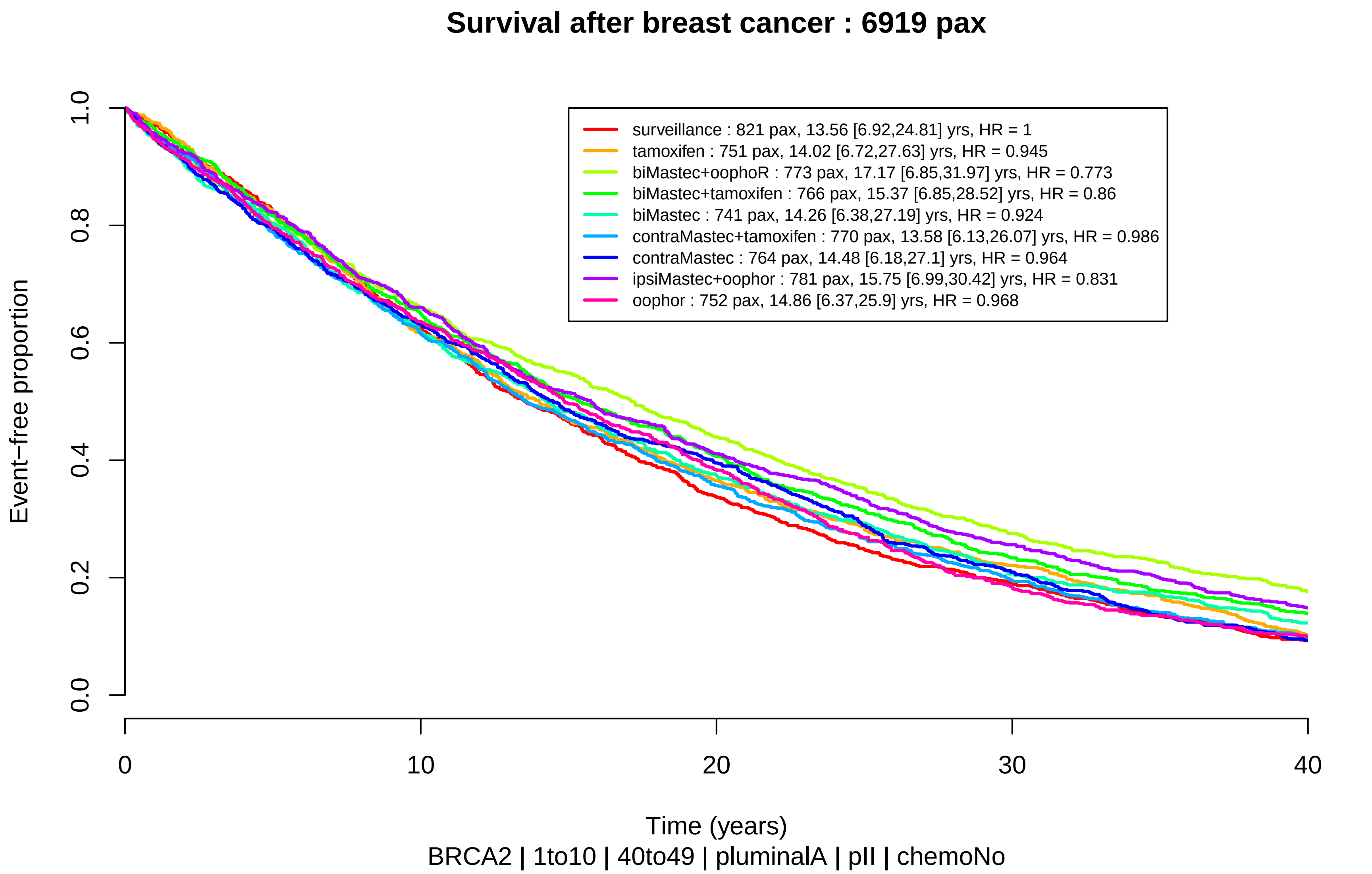

Survival after breast cancer : 6919 pax
surveillance : 821 pax, 13.56 [6.92,24.81] yrs, HR = 1
tamoxifen : 751 pax, 14.02 [6.72,27.63] yrs, HR = 0.945
biMastec+oophoR : 773 pax, 17.17 [6.85,31.97] yrs, HR = 0.773
biMastec+tamoxifen : 766 pax, 15.37 [6.85,28.52] yrs, HR = 0.86
biMastec : 741 pax, 14.26 [6.38,27.19] yrs, HR = 0.924
contraMastec+tamoxifen : 770 pax, 13.58 [6.13,26.07] yrs, HR = 0.986
contraMastec : 764 pax, 14.48 [6.18,27.1] yrs, HR = 0.964
ipsiMastec+oophor : 781 pax, 15.75 [6.99,30.42] yrs, HR = 0.831
oophor : 752 pax, 14.86 [6.37,25.9] yrs, HR = 0.968
Event−free proportion
0.0
0.2
0.4
0.6
0.8
1.0
0
10
20
30
40
Time (years)
BRCA2 | 1to10 | 40to49 | pluminalA | pII | chemoNo

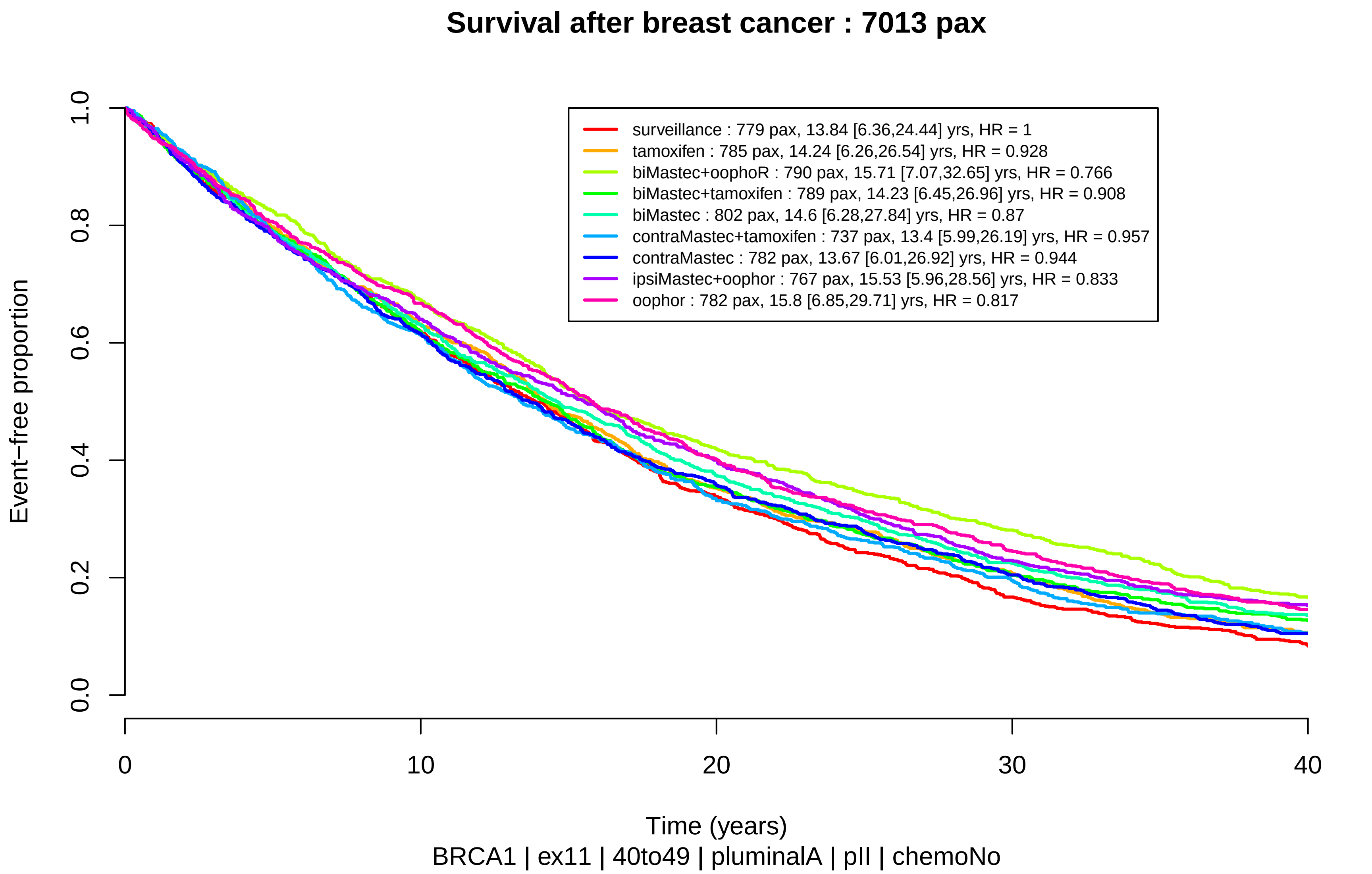
Survival after breast cancer : 7013 pax
surveillance : 779 pax, 13.84 [6.36,24.44] yrs, HR = 1
tamoxifen : 785 pax, 14.24 [6.26,26.54] yrs, HR = 0.928
biMastec+oophoR : 790 pax, 15.71 [7.07,32.65] yrs, HR = 0.766
biMastec+tamoxifen : 789 pax, 14.23 [6.45,26.96] yrs, HR = 0.908
biMastec : 802 pax, 14.6 [6.28,27.84] yrs, HR = 0.87
contraMastec+tamoxifen : 737 pax, 13.4 [5.99,26.19] yrs, HR = 0.957
contraMastec : 782 pax, 13.67 [6.01,26.92] yrs, HR = 0.944
ipsiMastec+oophor : 767 pax, 15.53 [5.96,28.56] yrs, HR = 0.833
oophor : 782 pax, 15.8 [6.85,29.71] yrs, HR = 0.817
Event−free proportion
0.0
0.2
0.4
0.6
0.8
1.0
0
10
20
30
40
Time (years)
BRCA1 | ex11 | 40to49 | pluminalA | pII | chemoNo

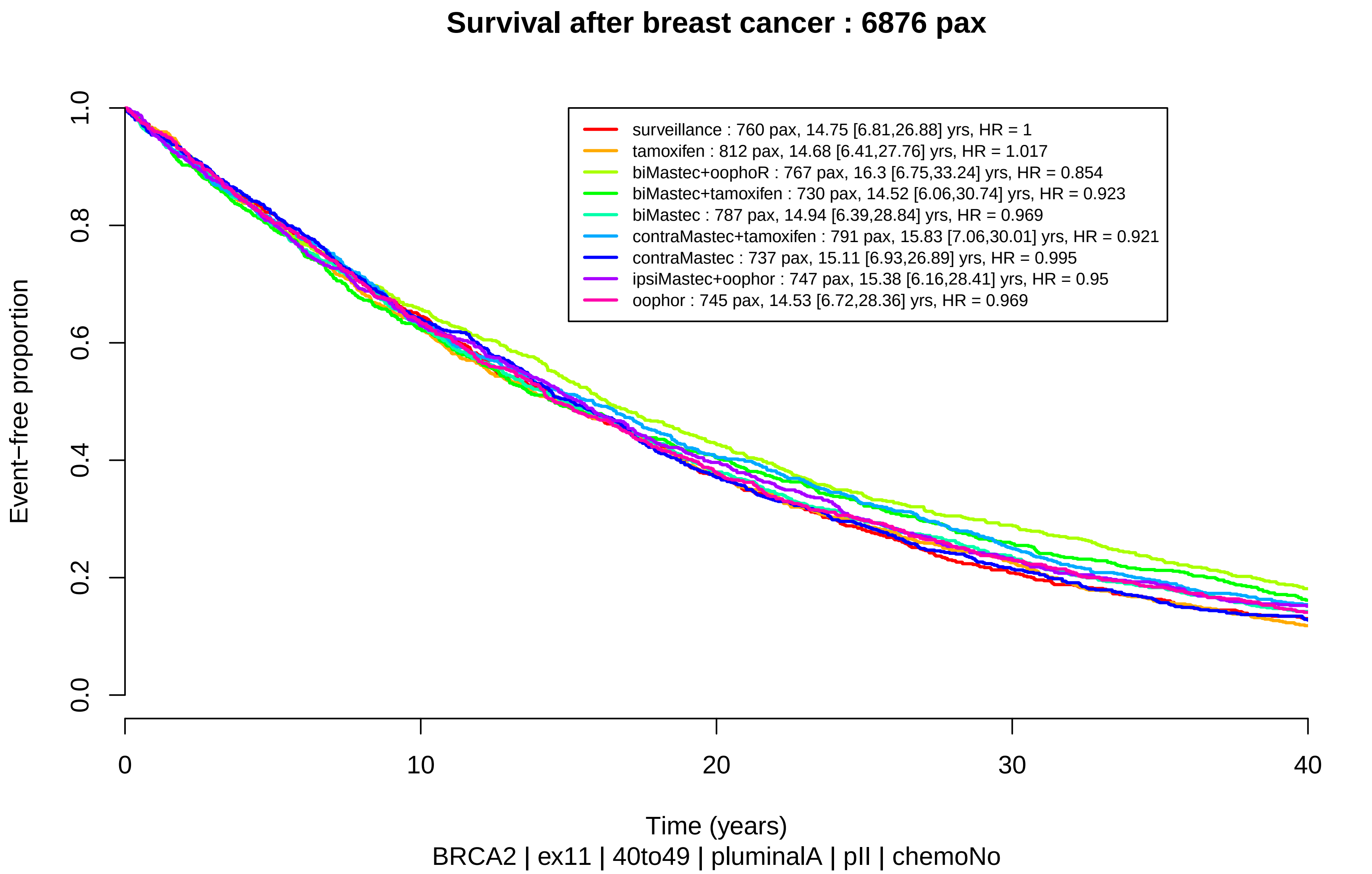

Survival after breast cancer : 6876 pax
surveillance : 760 pax, 14.75 [6.81,26.88] yrs, HR = 1
tamoxifen : 812 pax, 14.68 [6.41,27.76] yrs, HR = 1.017
biMastec+oophoR : 767 pax, 16.3 [6.75,33.24] yrs, HR = 0.854
biMastec+tamoxifen : 730 pax, 14.52 [6.06,30.74] yrs, HR = 0.923
biMastec : 787 pax, 14.94 [6.39,28.84] yrs, HR = 0.969
contraMastec+tamoxifen : 791 pax, 15.83 [7.06,30.01] yrs, HR = 0.921
contraMastec : 737 pax, 15.11 [6.93,26.89] yrs, HR = 0.995
ipsiMastec+oophor : 747 pax, 15.38 [6.16,28.41] yrs, HR = 0.95
oophor : 745 pax, 14.53 [6.72,28.36] yrs, HR = 0.969
Event−free proportion
0.0
0.2
0.4
0.6
0.8
1.0
0
10
20
30
40
Time (years)
BRCA2 | ex11 | 40to49 | pluminalA | pII | chemoNo

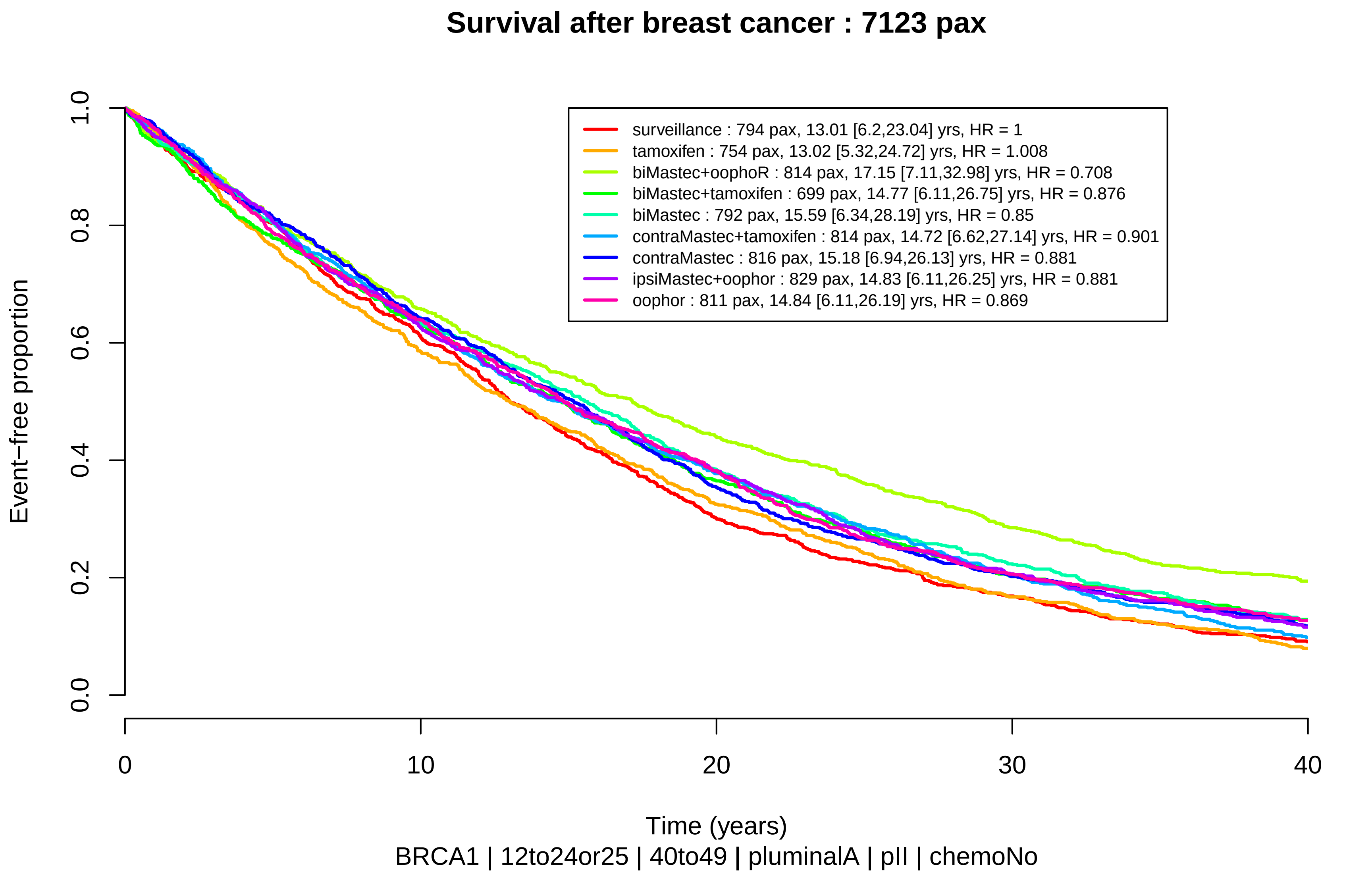
Survival after breast cancer : 7123 pax
surveillance : 794 pax, 13.01 [6.2,23.04] yrs, HR = 1
tamoxifen : 754 pax, 13.02 [5.32,24.72] yrs, HR = 1.008
biMastec+oophoR : 814 pax, 17.15 [7.11,32.98] yrs, HR = 0.708
biMastec+tamoxifen : 699 pax, 14.77 [6.11,26.75] yrs, HR = 0.876
biMastec : 792 pax, 15.59 [6.34,28.19] yrs, HR = 0.85
contraMastec+tamoxifen : 814 pax, 14.72 [6.62,27.14] yrs, HR = 0.901
contraMastec : 816 pax, 15.18 [6.94,26.13] yrs, HR = 0.881
ipsiMastec+oophor : 829 pax, 14.83 [6.11,26.25] yrs, HR = 0.881
oophor : 811 pax, 14.84 [6.11,26.19] yrs, HR = 0.869
Event−free proportion
0.0
0.2
0.4
0.6
0.8
1.0
0
10
20
30
40
Time (years)
BRCA1 | 12to24or25 | 40to49 | pluminalA | pII | chemoNo

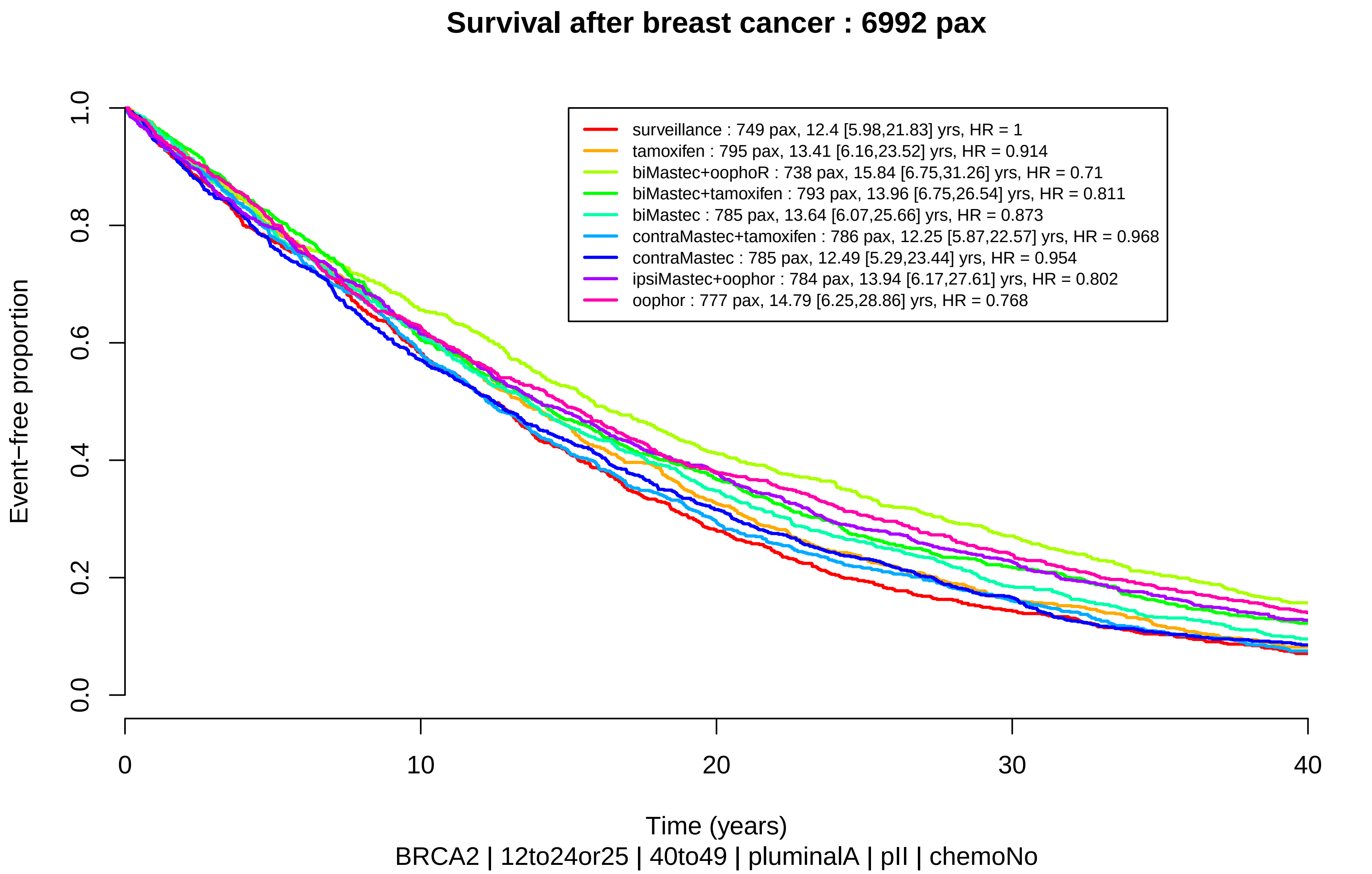

Survival after breast cancer : 6992 pax
surveillance : 749 pax, 12.4 [5.98,21.83] yrs, HR = 1
tamoxifen : 795 pax, 13.41 [6.16,23.52] yrs, HR = 0.914
biMastec+oophoR : 738 pax, 15.84 [6.75,31.26] yrs, HR = 0.71
biMastec+tamoxifen : 793 pax, 13.96 [6.75,26.54] yrs, HR = 0.811
biMastec : 785 pax, 13.64 [6.07,25.66] yrs, HR = 0.873
contraMastec+tamoxifen : 786 pax, 12.25 [5.87,22.57] yrs, HR = 0.968
contraMastec : 785 pax, 12.49 [5.29,23.44] yrs, HR = 0.954
ipsiMastec+oophor : 784 pax, 13.94 [6.17,27.61] yrs, HR = 0.802
oophor : 777 pax, 14.79 [6.25,28.86] yrs, HR = 0.768
Event−free proportion
0.0
0.2
0.4
0.6
0.8
1.0
0
10
20
30
40
Time (years)
BRCA2 | 12to24or25 | 40to49 | pluminalA | pII | chemoNo

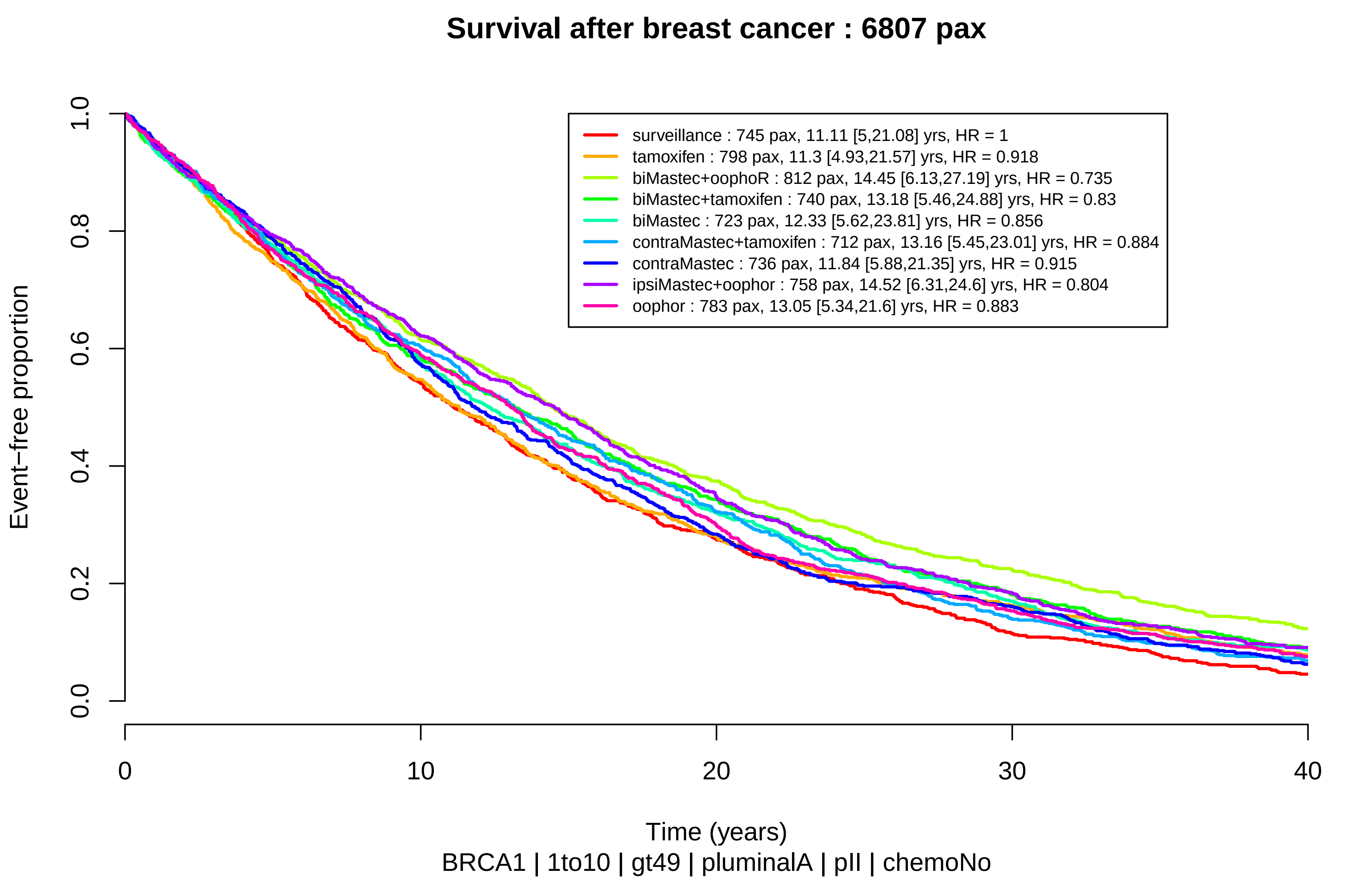

Survival after breast cancer : 6807 pax
surveillance : 745 pax, 11.11 [5,21.08] yrs, HR = 1
tamoxifen : 798 pax, 11.3 [4.93,21.57] yrs, HR = 0.918
biMastec+oophoR : 812 pax, 14.45 [6.13,27.19] yrs, HR = 0.735
biMastec+tamoxifen : 740 pax, 13.18 [5.46,24.88] yrs, HR = 0.83
biMastec : 723 pax, 12.33 [5.62,23.81] yrs, HR = 0.856
contraMastec+tamoxifen : 712 pax, 13.16 [5.45,23.01] yrs, HR = 0.884
contraMastec : 736 pax, 11.84 [5.88,21.35] yrs, HR = 0.915
ipsiMastec+oophor : 758 pax, 14.52 [6.31,24.6] yrs, HR = 0.804
oophor : 783 pax, 13.05 [5.34,21.6] yrs, HR = 0.883
Event−free proportion
0.0
0.2
0.4
0.6
0.8
1.0
0
10
20
30
40
Time (years)
BRCA1 | 1to10 | gt49 | pluminalA | pII | chemoNo

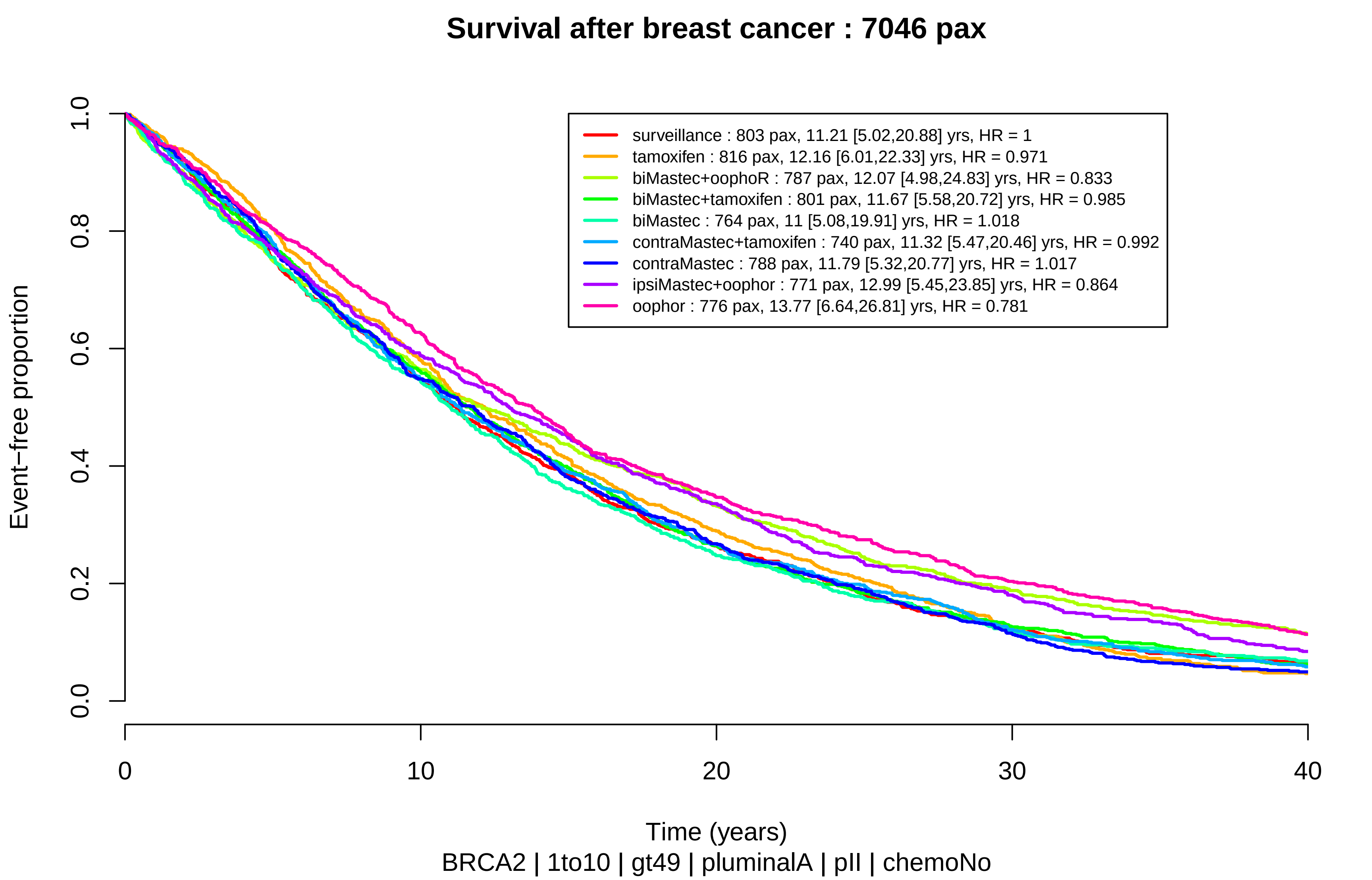
Survival after breast cancer : 7046 pax
surveillance : 803 pax, 11.21 [5.02,20.88] yrs, HR = 1
tamoxifen : 816 pax, 12.16 [6.01,22.33] yrs, HR = 0.971
biMastec+oophoR : 787 pax, 12.07 [4.98,24.83] yrs, HR = 0.833
biMastec+tamoxifen : 801 pax, 11.67 [5.58,20.72] yrs, HR = 0.985
biMastec : 764 pax, 11 [5.08,19.91] yrs, HR = 1.018
contraMastec+tamoxifen : 740 pax, 11.32 [5.47,20.46] yrs, HR = 0.992
contraMastec : 788 pax, 11.79 [5.32,20.77] yrs, HR = 1.017
ipsiMastec+oophor : 771 pax, 12.99 [5.45,23.85] yrs, HR = 0.864
oophor : 776 pax, 13.77 [6.64,26.81] yrs, HR = 0.781
Event−free proportion
0.0
0.2
0.4
0.6
0.8
1.0
0
10
20
30
40
Time (years)
BRCA2 | 1to10 | gt49 | pluminalA | pII | chemoNo

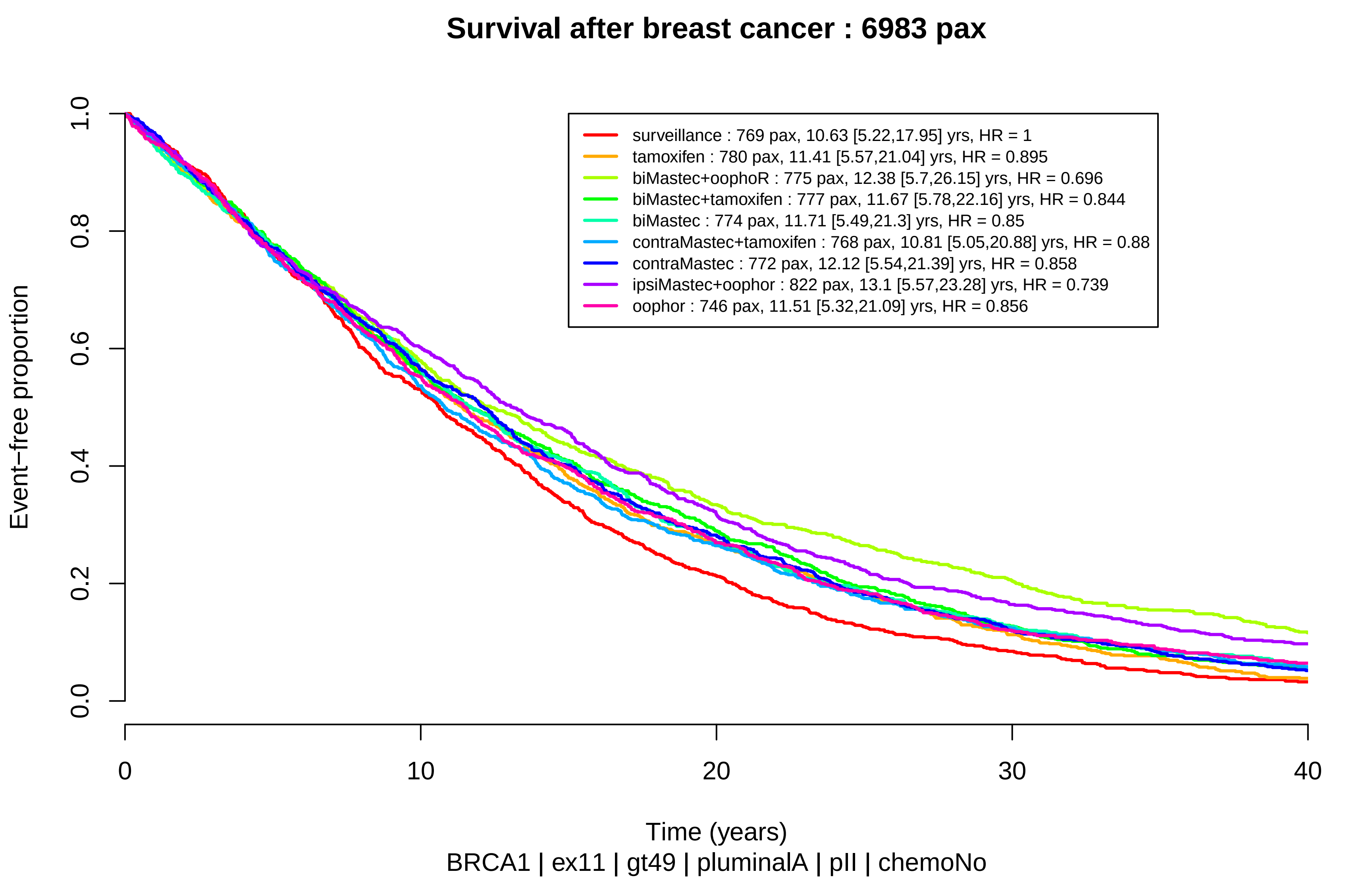

Survival after breast cancer : 6983 pax
surveillance : 769 pax, 10.63 [5.22,17.95] yrs, HR = 1
tamoxifen : 780 pax, 11.41 [5.57,21.04] yrs, HR = 0.895
biMastec+oophoR : 775 pax, 12.38 [5.7,26.15] yrs, HR = 0.696
biMastec+tamoxifen : 777 pax, 11.67 [5.78,22.16] yrs, HR = 0.844
biMastec : 774 pax, 11.71 [5.49,21.3] yrs, HR = 0.85
contraMastec+tamoxifen : 768 pax, 10.81 [5.05,20.88] yrs, HR = 0.88
contraMastec : 772 pax, 12.12 [5.54,21.39] yrs, HR = 0.858
ipsiMastec+oophor : 822 pax, 13.1 [5.57,23.28] yrs, HR = 0.739
oophor : 746 pax, 11.51 [5.32,21.09] yrs, HR = 0.856
Event−free proportion
0.0
0.2
0.4
0.6
0.8
1.0
0
10
20
30
40
Time (years)
BRCA1 | ex11 | gt49 | pluminalA | pII | chemoNo

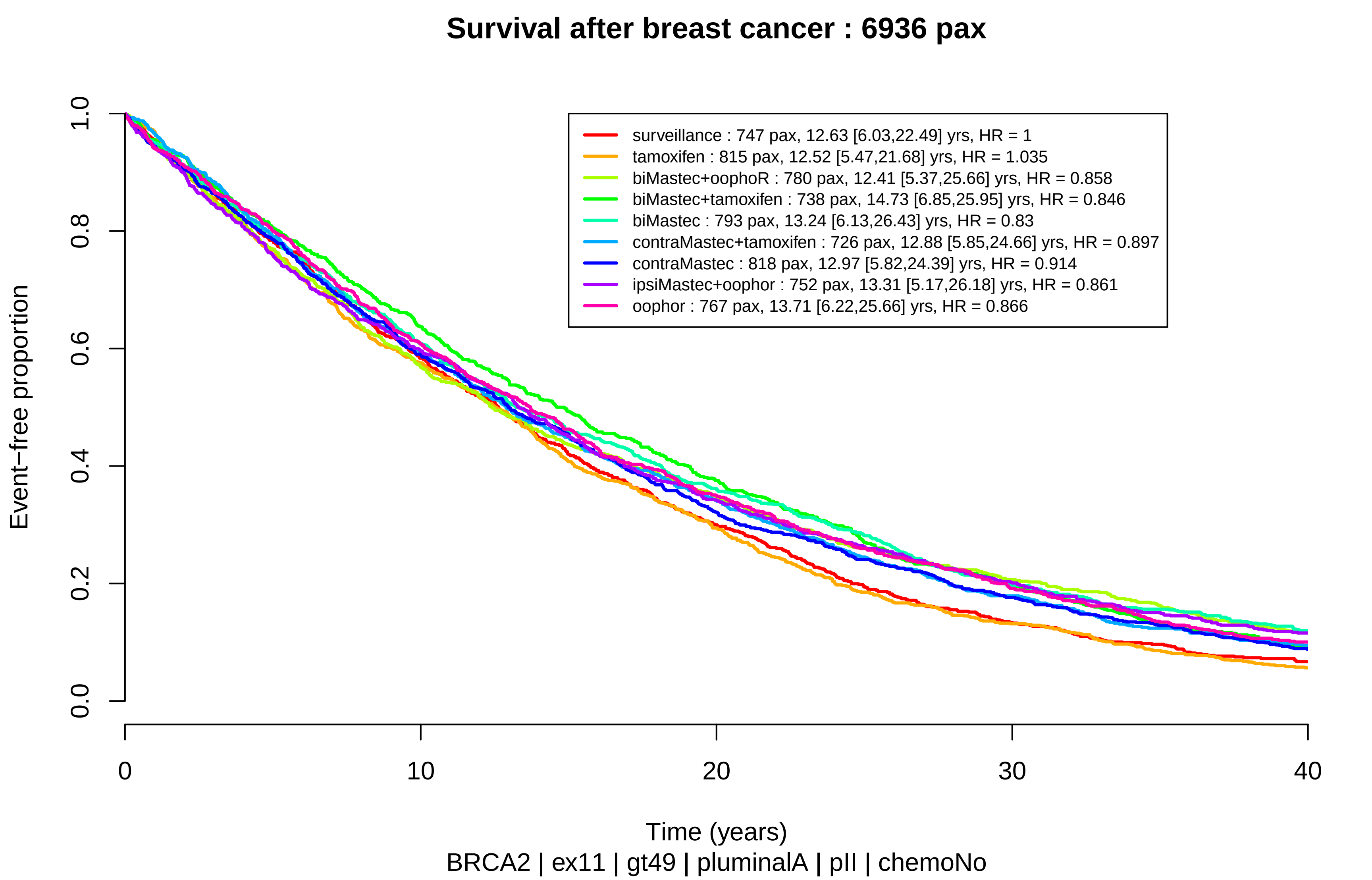

Survival after breast cancer : 6936 pax
surveillance : 747 pax, 12.63 [6.03,22.49] yrs, HR = 1
tamoxifen : 815 pax, 12.52 [5.47,21.68] yrs, HR = 1.035
biMastec+oophoR : 780 pax, 12.41 [5.37,25.66] yrs, HR = 0.858
biMastec+tamoxifen : 738 pax, 14.73 [6.85,25.95] yrs, HR = 0.846
biMastec : 793 pax, 13.24 [6.13,26.43] yrs, HR = 0.83
contraMastec+tamoxifen : 726 pax, 12.88 [5.85,24.66] yrs, HR = 0.897
contraMastec : 818 pax, 12.97 [5.82,24.39] yrs, HR = 0.914
ipsiMastec+oophor : 752 pax, 13.31 [5.17,26.18] yrs, HR = 0.861
oophor : 767 pax, 13.71 [6.22,25.66] yrs, HR = 0.866
Event−free proportion
0.0
0.2
0.4
0.6
0.8
1.0
0
10
20
30
40
Time (years)
BRCA2 | ex11 | gt49 | pluminalA | pII | chemoNo

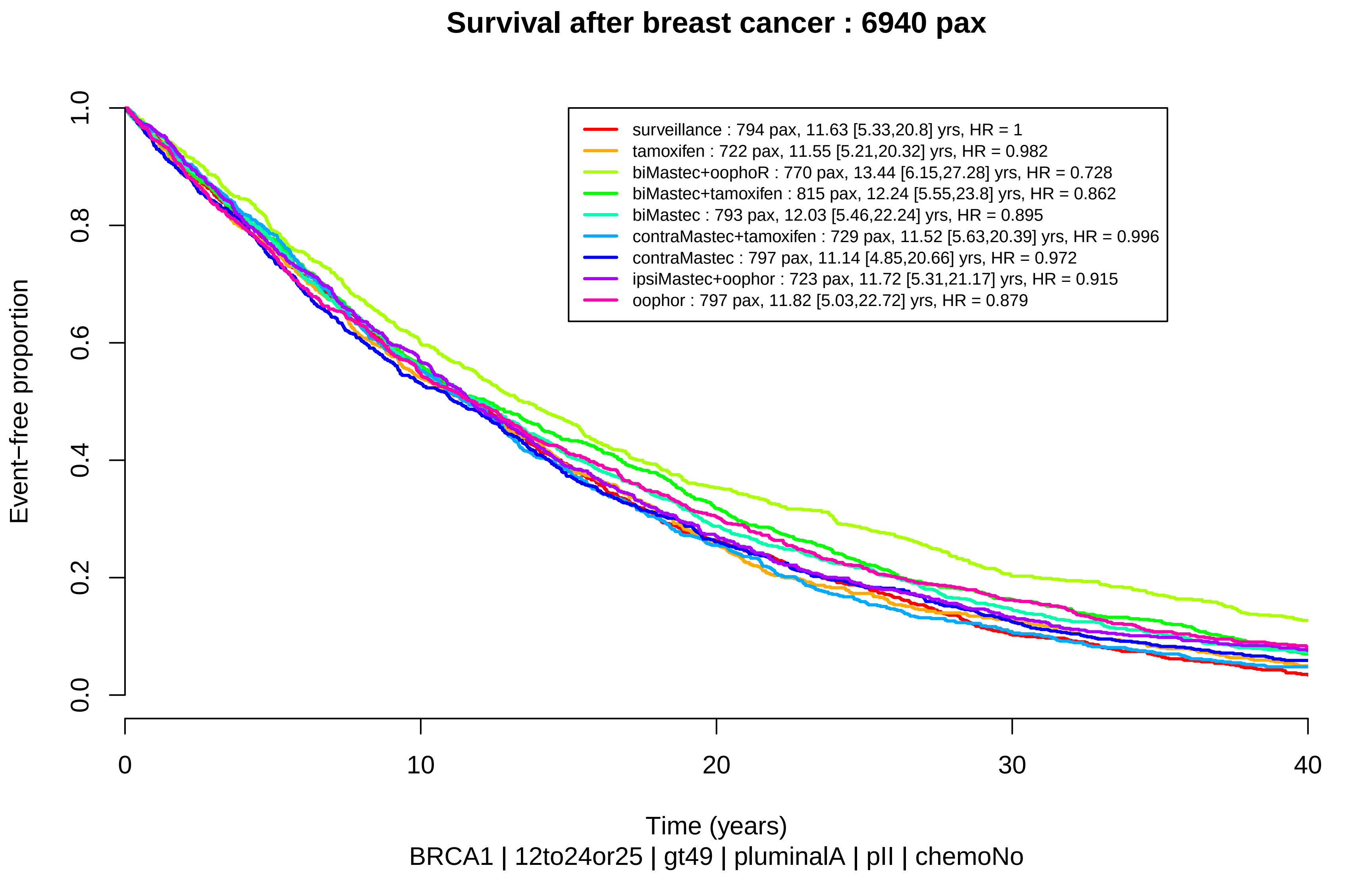
Survival after breast cancer : 6940 pax
surveillance : 794 pax, 11.63 [5.33,20.8] yrs, HR = 1
tamoxifen : 722 pax, 11.55 [5.21,20.32] yrs, HR = 0.982
biMastec+oophoR : 770 pax, 13.44 [6.15,27.28] yrs, HR = 0.728
biMastec+tamoxifen : 815 pax, 12.24 [5.55,23.8] yrs, HR = 0.862
biMastec : 793 pax, 12.03 [5.46,22.24] yrs, HR = 0.895
contraMastec+tamoxifen : 729 pax, 11.52 [5.63,20.39] yrs, HR = 0.996
contraMastec : 797 pax, 11.14 [4.85,20.66] yrs, HR = 0.972
ipsiMastec+oophor : 723 pax, 11.72 [5.31,21.17] yrs, HR = 0.915
oophor : 797 pax, 11.82 [5.03,22.72] yrs, HR = 0.879
Event−free proportion
0.0
0.2
0.4
0.6
0.8
1.0
0
10
20
30
40
Time (years)
BRCA1 | 12to24or25 | gt49 | pluminalA | pII | chemoNo

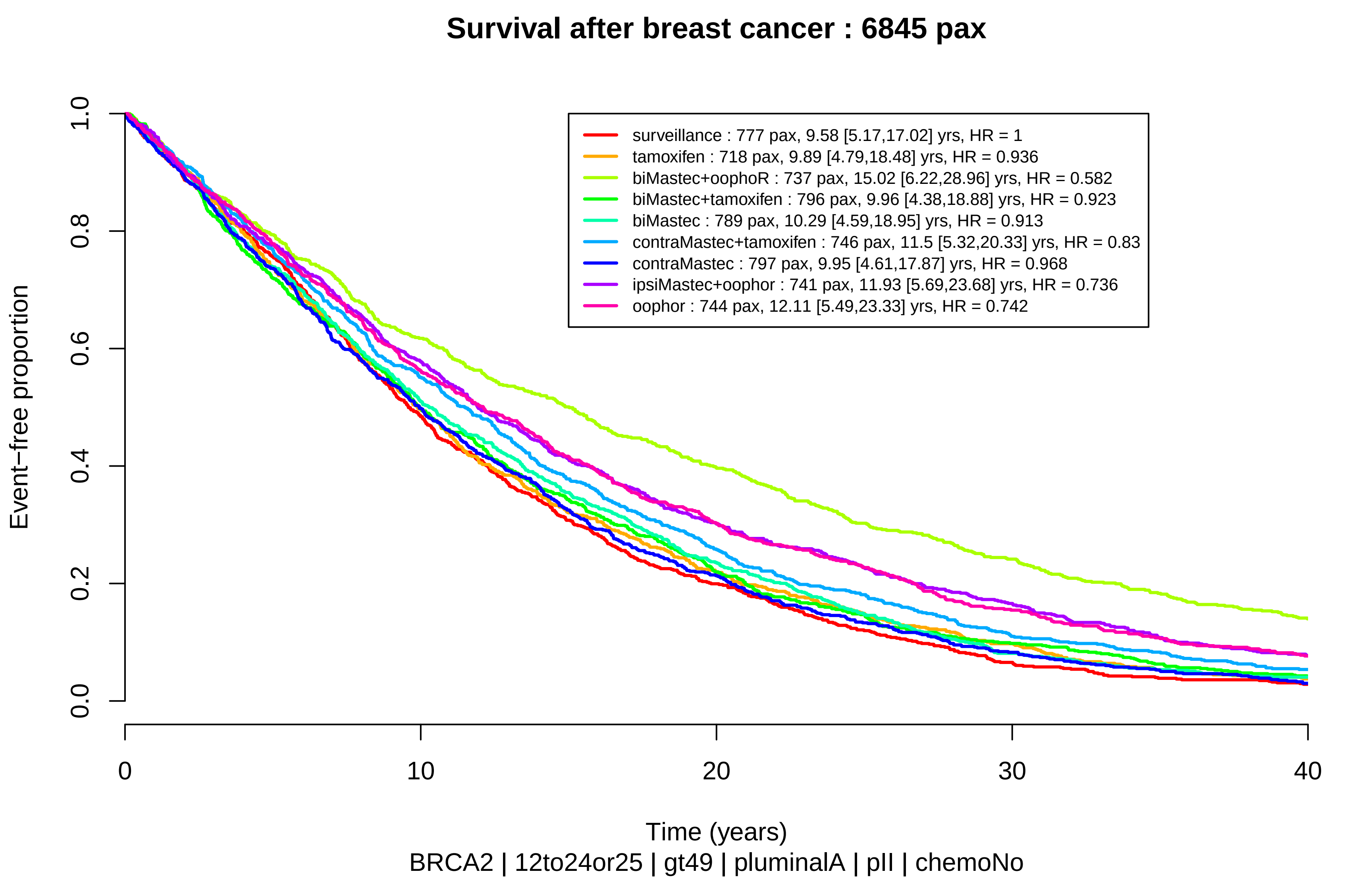

Survival after breast cancer : 6845 pax
surveillance : 777 pax, 9.58 [5.17,17.02] yrs, HR = 1
tamoxifen : 718 pax, 9.89 [4.79,18.48] yrs, HR = 0.936
biMastec+oophoR : 737 pax, 15.02 [6.22,28.96] yrs, HR = 0.582
biMastec+tamoxifen : 796 pax, 9.96 [4.38,18.88] yrs, HR = 0.923
biMastec : 789 pax, 10.29 [4.59,18.95] yrs, HR = 0.913
contraMastec+tamoxifen : 746 pax, 11.5 [5.32,20.33] yrs, HR = 0.83
contraMastec : 797 pax, 9.95 [4.61,17.87] yrs, HR = 0.968
ipsiMastec+oophor : 741 pax, 11.93 [5.69,23.68] yrs, HR = 0.736
oophor : 744 pax, 12.11 [5.49,23.33] yrs, HR = 0.742
Event−free proportion
0.0
0.2
0.4
0.6
0.8
1.0
0
10
20
30
40
Time (years)
BRCA2 | 12to24or25 | gt49 | pluminalA | pII | chemoNo

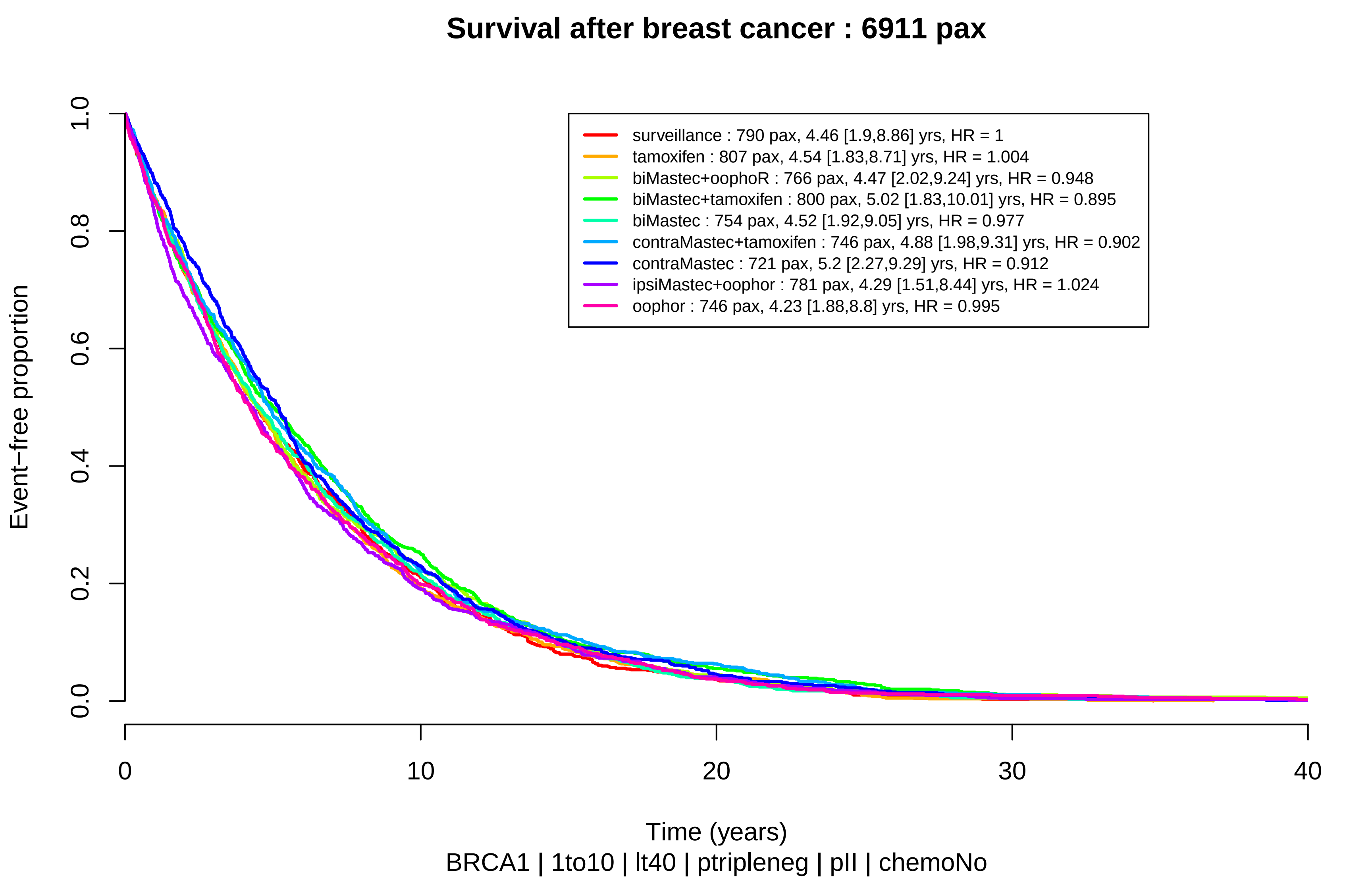
Survival after breast cancer : 6911 pax
surveillance : 790 pax, 4.46 [1.9,8.86] yrs, HR = 1
tamoxifen : 807 pax, 4.54 [1.83,8.71] yrs, HR = 1.004
biMastec+oophoR : 766 pax, 4.47 [2.02,9.24] yrs, HR = 0.948
biMastec+tamoxifen : 800 pax, 5.02 [1.83,10.01] yrs, HR = 0.895
biMastec : 754 pax, 4.52 [1.92,9.05] yrs, HR = 0.977
contraMastec+tamoxifen : 746 pax, 4.88 [1.98,9.31] yrs, HR = 0.902
contraMastec : 721 pax, 5.2 [2.27,9.29] yrs, HR = 0.912
ipsiMastec+oophor : 781 pax, 4.29 [1.51,8.44] yrs, HR = 1.024
oophor : 746 pax, 4.23 [1.88,8.8] yrs, HR = 0.995
Event−free proportion
0.0
0.2
0.4
0.6
0.8
1.0
0
10
20
30
40
Time (years)
BRCA1 | 1to10 | lt40 | ptripleneg | pII | chemoNo

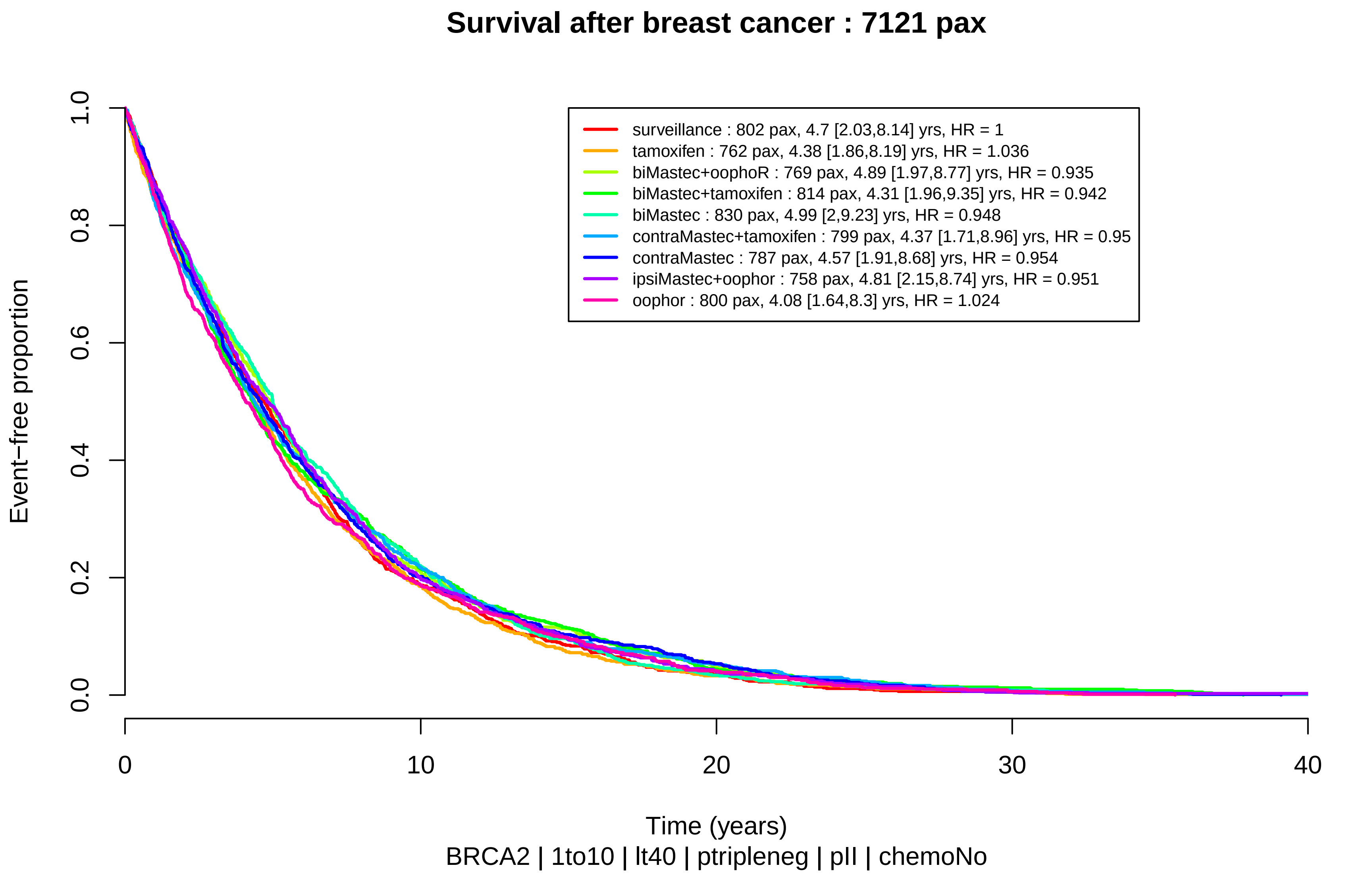
Survival after breast cancer : 7121 pax
Event−free proportion
0.0
0.2
0.4
0.6
0.8
1.0
0
10
20
30
40
Time (years)
BRCA2 | 1to10 | lt40 | ptripleneg | pII | chemoNo
surveillance : 802 pax, 4.7 [2.03,8.14] yrs, HR = 1
tamoxifen : 762 pax, 4.38 [1.86,8.19] yrs, HR = 1.036
biMastec+oophoR : 769 pax, 4.89 [1.97,8.77] yrs, HR = 0.935
biMastec+tamoxifen : 814 pax, 4.31 [1.96,9.35] yrs, HR = 0.942
biMastec : 830 pax, 4.99 [2,9.23] yrs, HR = 0.948
contraMastec+tamoxifen : 799 pax, 4.37 [1.71,8.96] yrs, HR = 0.95
contraMastec : 787 pax, 4.57 [1.91,8.68] yrs, HR = 0.954
ipsiMastec+oophor : 758 pax, 4.81 [2.15,8.74] yrs, HR = 0.951
oophor : 800 pax, 4.08 [1.64,8.3] yrs, HR = 1.024

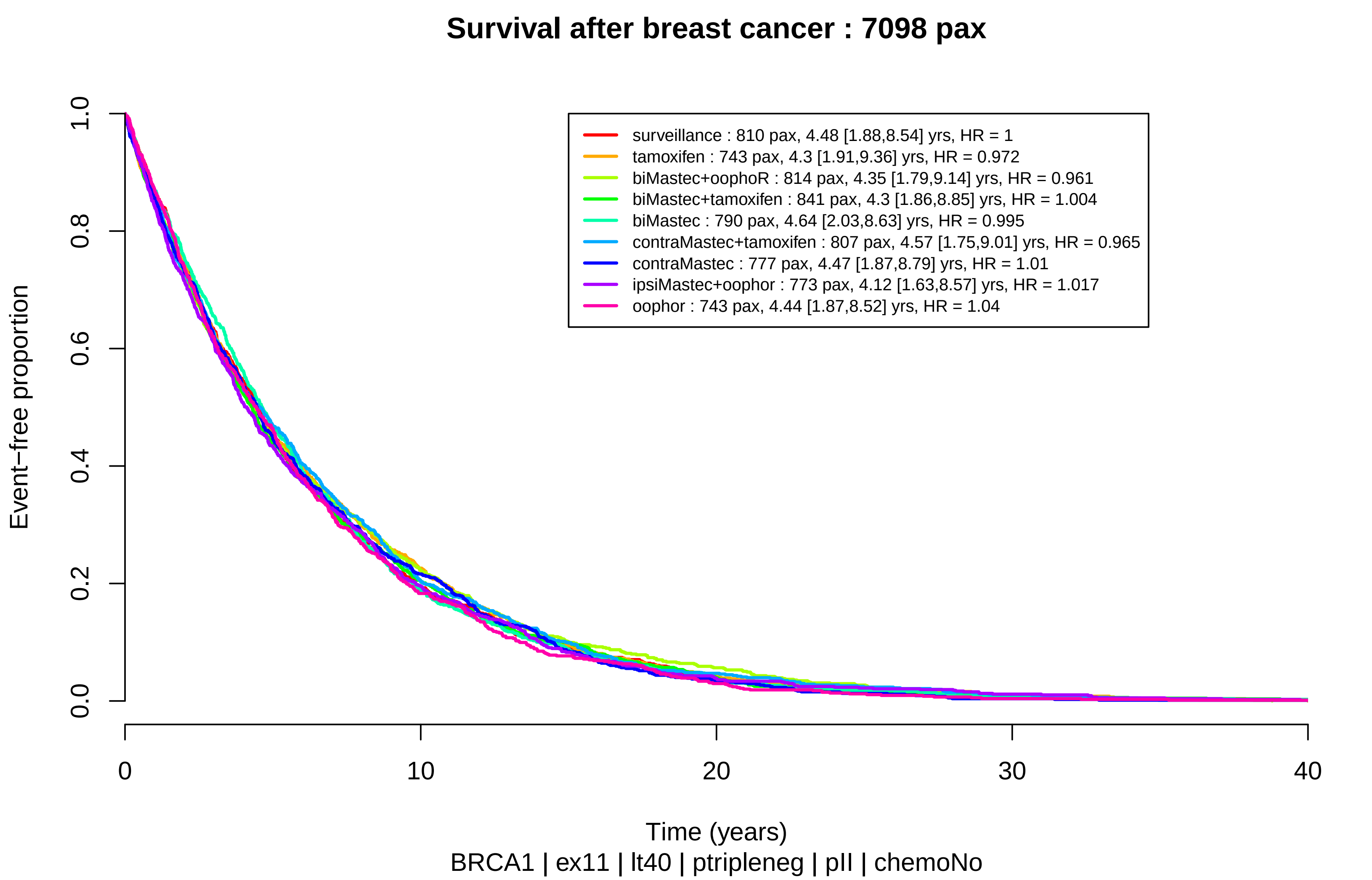
Survival after breast cancer : 7098 pax
surveillance : 810 pax, 4.48 [1.88,8.54] yrs, HR = 1
tamoxifen : 743 pax, 4.3 [1.91,9.36] yrs, HR = 0.972
biMastec+oophoR : 814 pax, 4.35 [1.79,9.14] yrs, HR = 0.961
biMastec+tamoxifen : 841 pax, 4.3 [1.86,8.85] yrs, HR = 1.004
biMastec : 790 pax, 4.64 [2.03,8.63] yrs, HR = 0.995
contraMastec+tamoxifen : 807 pax, 4.57 [1.75,9.01] yrs, HR = 0.965
contraMastec : 777 pax, 4.47 [1.87,8.79] yrs, HR = 1.01
ipsiMastec+oophor : 773 pax, 4.12 [1.63,8.57] yrs, HR = 1.017
oophor : 743 pax, 4.44 [1.87,8.52] yrs, HR = 1.04
Event−free proportion
0.0
0.2
0.4
0.6
0.8
1.0
0
10
20
30
40
Time (years)
BRCA1 | ex11 | lt40 | ptripleneg | pII | chemoNo

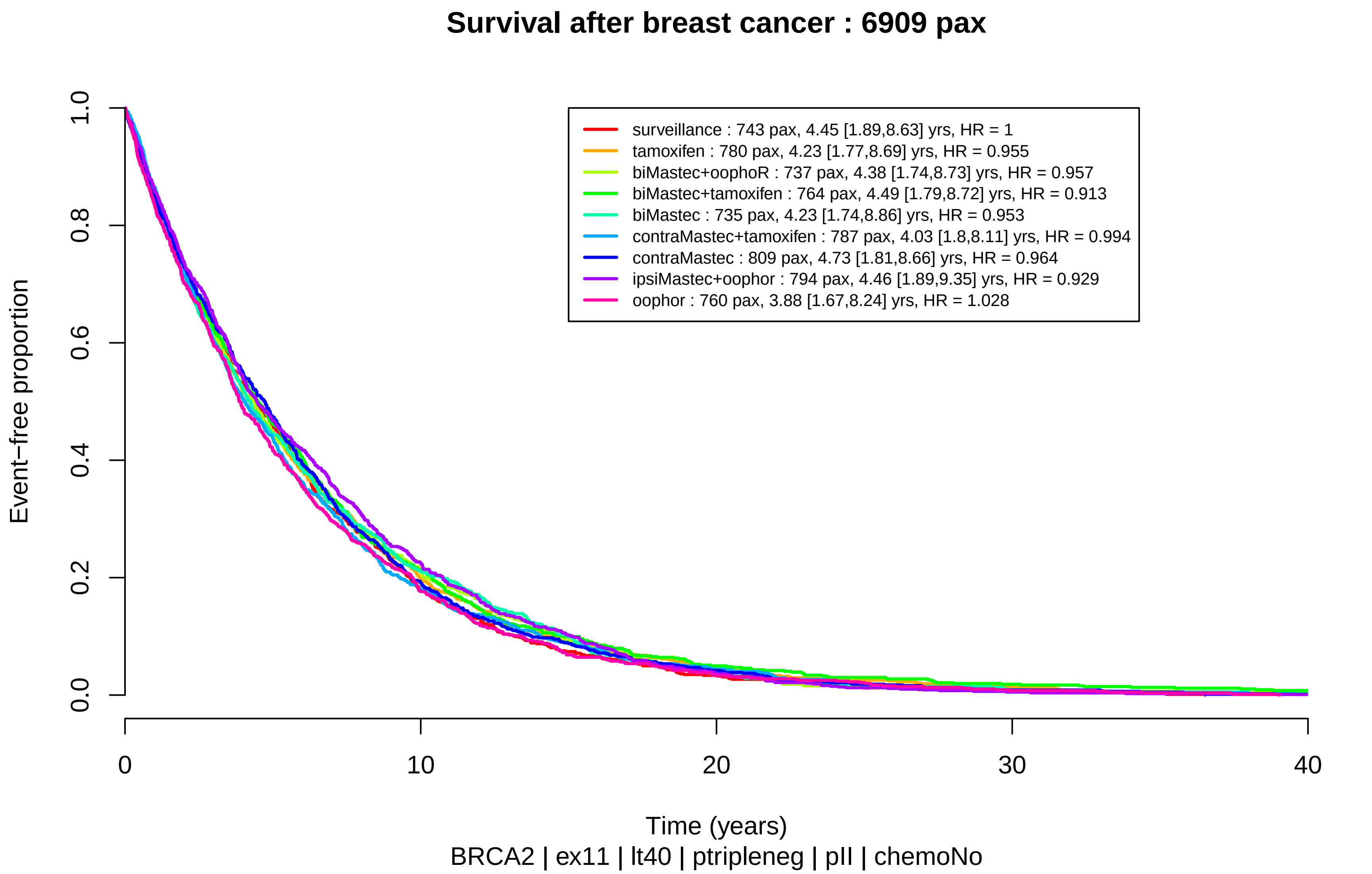

Survival after breast cancer : 6909 pax
surveillance : 743 pax, 4.45 [1.89,8.63] yrs, HR = 1
tamoxifen : 780 pax, 4.23 [1.77,8.69] yrs, HR = 0.955
biMastec+oophoR : 737 pax, 4.38 [1.74,8.73] yrs, HR = 0.957
biMastec+tamoxifen : 764 pax, 4.49 [1.79,8.72] yrs, HR = 0.913
biMastec : 735 pax, 4.23 [1.74,8.86] yrs, HR = 0.953
contraMastec+tamoxifen : 787 pax, 4.03 [1.8,8.11] yrs, HR = 0.994
contraMastec : 809 pax, 4.73 [1.81,8.66] yrs, HR = 0.964
ipsiMastec+oophor : 794 pax, 4.46 [1.89,9.35] yrs, HR = 0.929
oophor : 760 pax, 3.88 [1.67,8.24] yrs, HR = 1.028
Event−free proportion
0.0
0.2
0.4
0.6
0.8
1.0
0
10
20
30
40
Time (years)
BRCA2 | ex11 | lt40 | ptripleneg | pII | chemoNo

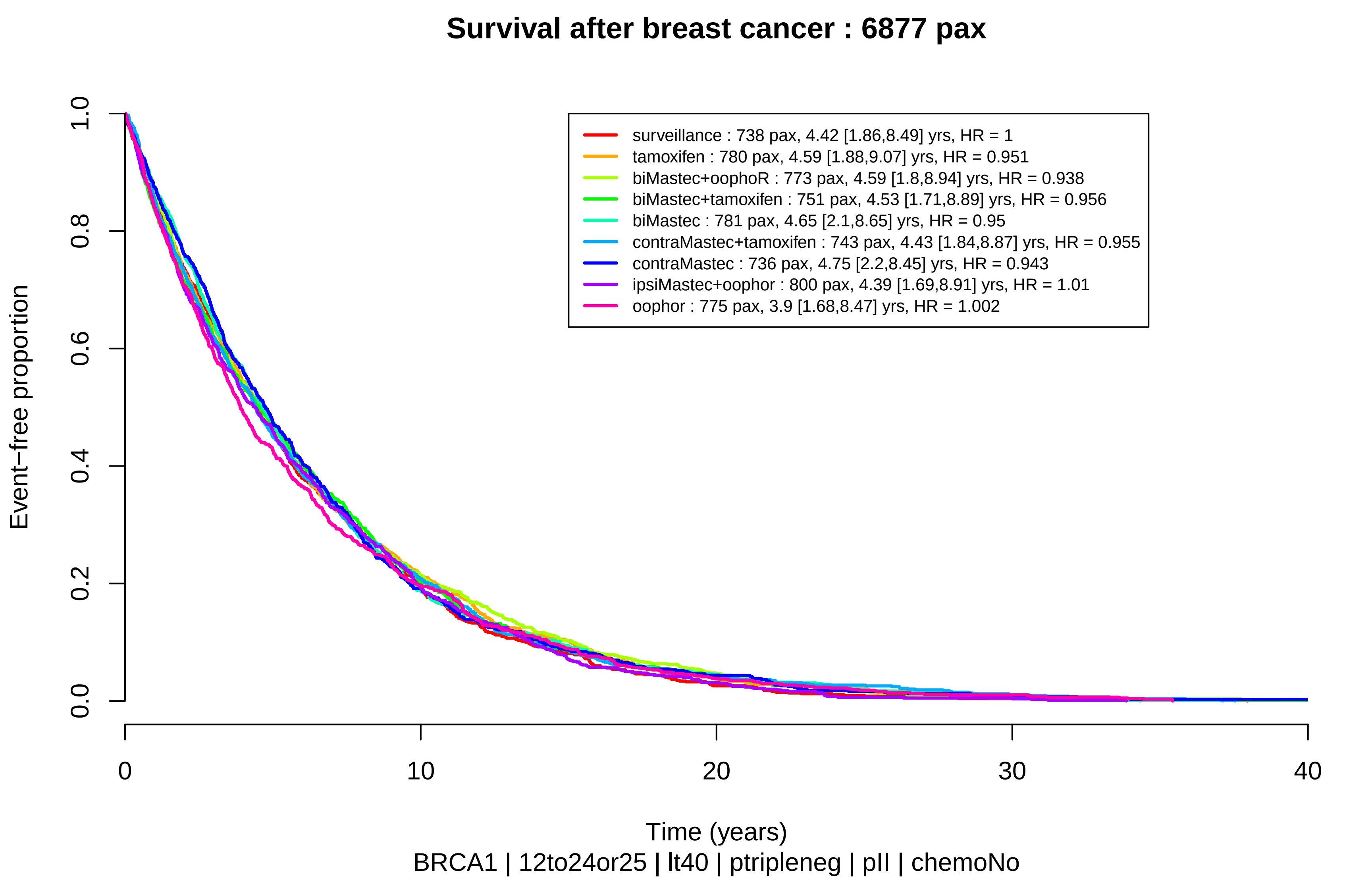

Survival after breast cancer : 6877 pax
Event−free proportion
0.0
0.2
0.4
0.6
0.8
1.0
0
10
20
30
40
Time (years)
BRCA1 | 12to24or25 | lt40 | ptripleneg | pII | chemoNo
surveillance : 738 pax, 4.42 [1.86,8.49] yrs, HR = 1
tamoxifen : 780 pax, 4.59 [1.88,9.07] yrs, HR = 0.951
biMastec+oophoR : 773 pax, 4.59 [1.8,8.94] yrs, HR = 0.938
biMastec+tamoxifen : 751 pax, 4.53 [1.71,8.89] yrs, HR = 0.956
biMastec : 781 pax, 4.65 [2.1,8.65] yrs, HR = 0.95
contraMastec+tamoxifen : 743 pax, 4.43 [1.84,8.87] yrs, HR = 0.955
contraMastec : 736 pax, 4.75 [2.2,8.45] yrs, HR = 0.943
ipsiMastec+oophor : 800 pax, 4.39 [1.69,8.91] yrs, HR = 1.01
oophor : 775 pax, 3.9 [1.68,8.47] yrs, HR = 1.002

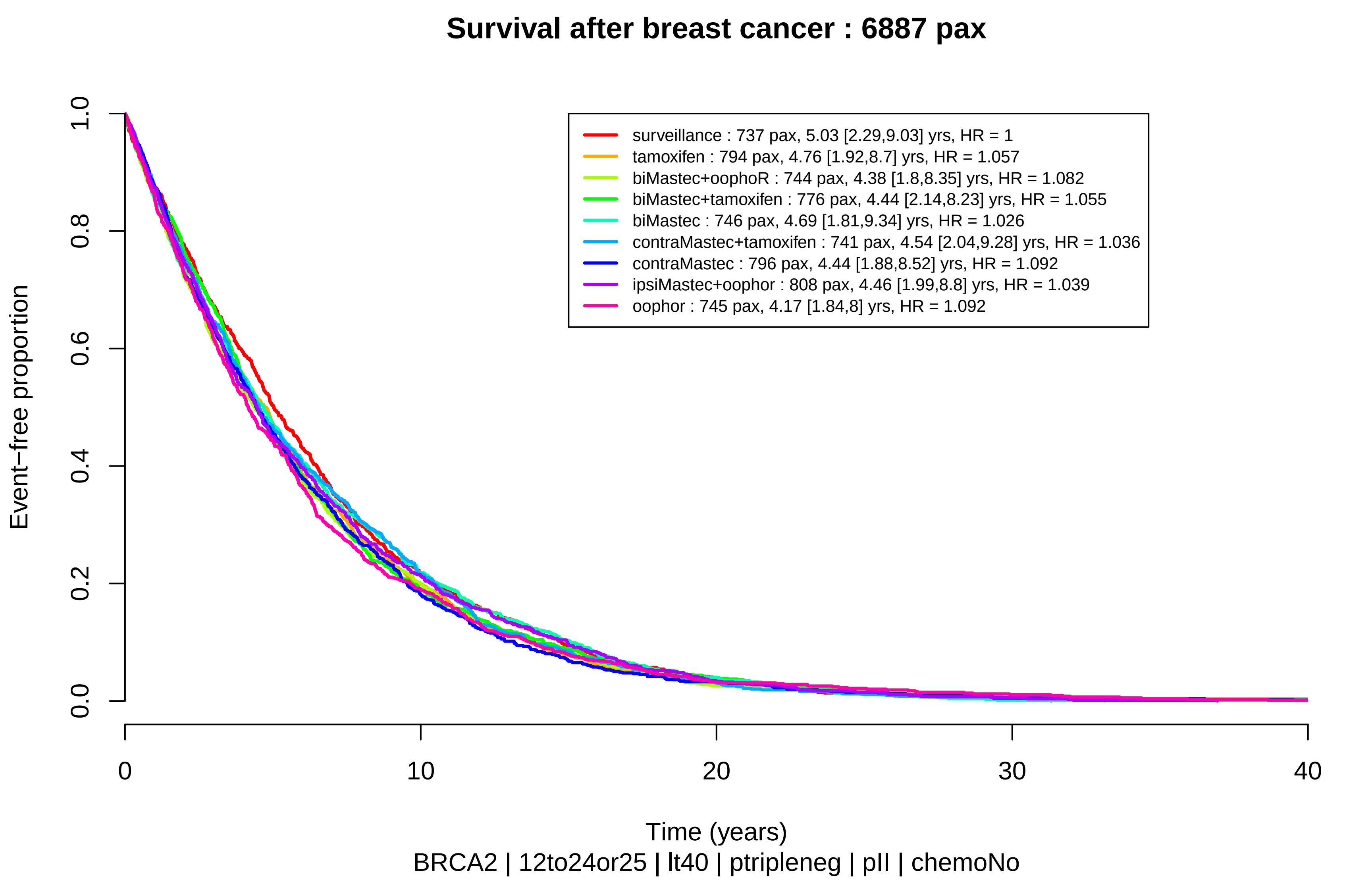
Survival after breast cancer : 6887 pax
surveillance : 737 pax, 5.03 [2.29,9.03] yrs, HR = 1
tamoxifen : 794 pax, 4.76 [1.92,8.7] yrs, HR = 1.057
biMastec+oophoR : 744 pax, 4.38 [1.8,8.35] yrs, HR = 1.082
biMastec+tamoxifen : 776 pax, 4.44 [2.14,8.23] yrs, HR = 1.055
biMastec : 746 pax, 4.69 [1.81,9.34] yrs, HR = 1.026
contraMastec+tamoxifen : 741 pax, 4.54 [2.04,9.28] yrs, HR = 1.036
contraMastec : 796 pax, 4.44 [1.88,8.52] yrs, HR = 1.092
ipsiMastec+oophor : 808 pax, 4.46 [1.99,8.8] yrs, HR = 1.039
oophor : 745 pax, 4.17 [1.84,8] yrs, HR = 1.092
Event−free proportion
0.0
0.2
0.4
0.6
0.8
1.0
0
10
20
30
40
Time (years)
BRCA2 | 12to24or25 | lt40 | ptripleneg | pII | chemoNo

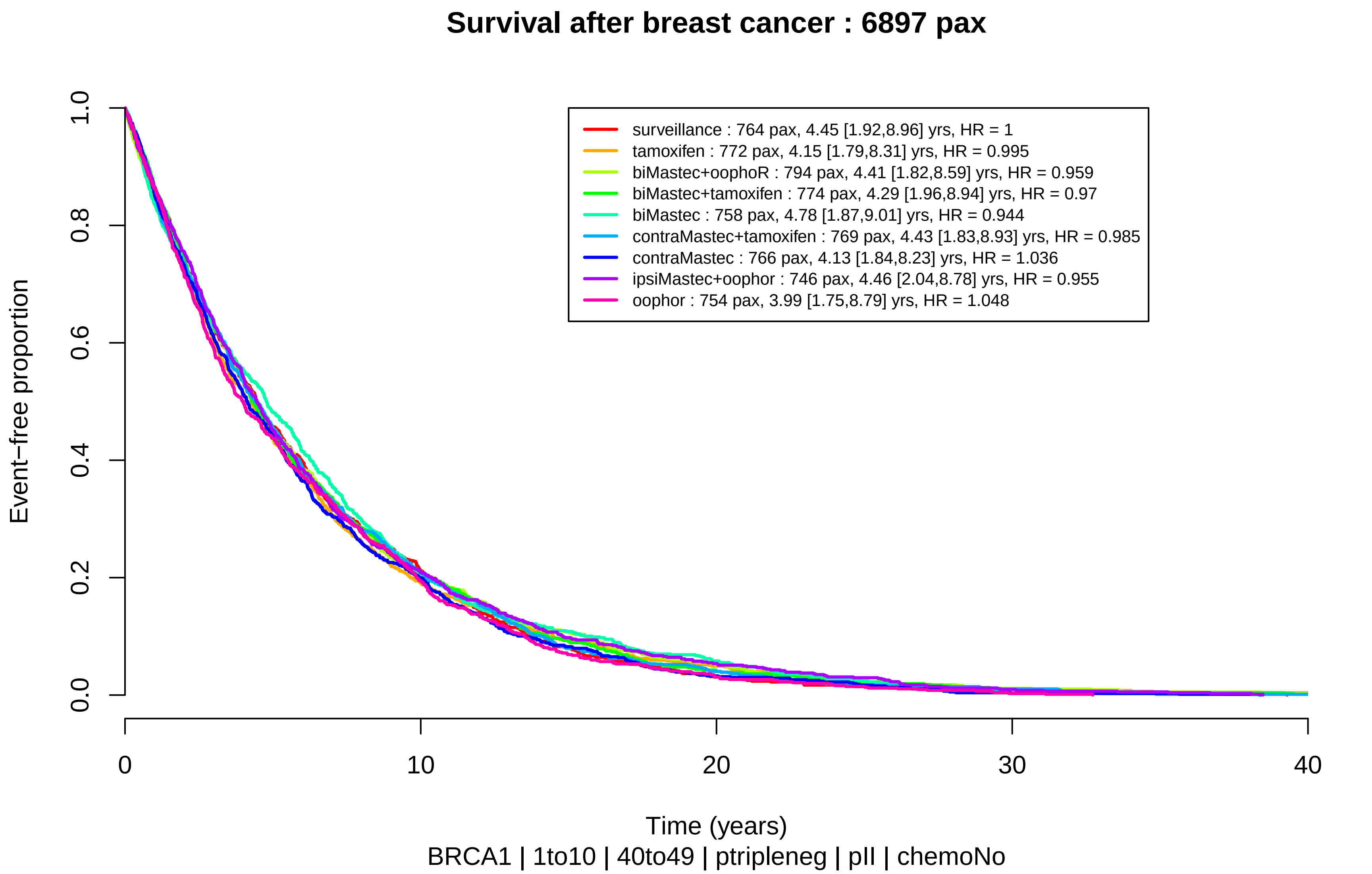

Survival after breast cancer : 6897 pax
Event−free proportion
0.0
0.2
0.4
0.6
0.8
1.0
0
10
20
30
40
Time (years)
BRCA1 | 1to10 | 40to49 | ptripleneg | pII | chemoNo
surveillance : 764 pax, 4.45 [1.92,8.96] yrs, HR = 1
tamoxifen : 772 pax, 4.15 [1.79,8.31] yrs, HR = 0.995
biMastec+oophoR : 794 pax, 4.41 [1.82,8.59] yrs, HR = 0.959
biMastec+tamoxifen : 774 pax, 4.29 [1.96,8.94] yrs, HR = 0.97
biMastec : 758 pax, 4.78 [1.87,9.01] yrs, HR = 0.944
contraMastec+tamoxifen : 769 pax, 4.43 [1.83,8.93] yrs, HR = 0.985
contraMastec : 766 pax, 4.13 [1.84,8.23] yrs, HR = 1.036
ipsiMastec+oophor : 746 pax, 4.46 [2.04,8.78] yrs, HR = 0.955
oophor : 754 pax, 3.99 [1.75,8.79] yrs, HR = 1.048

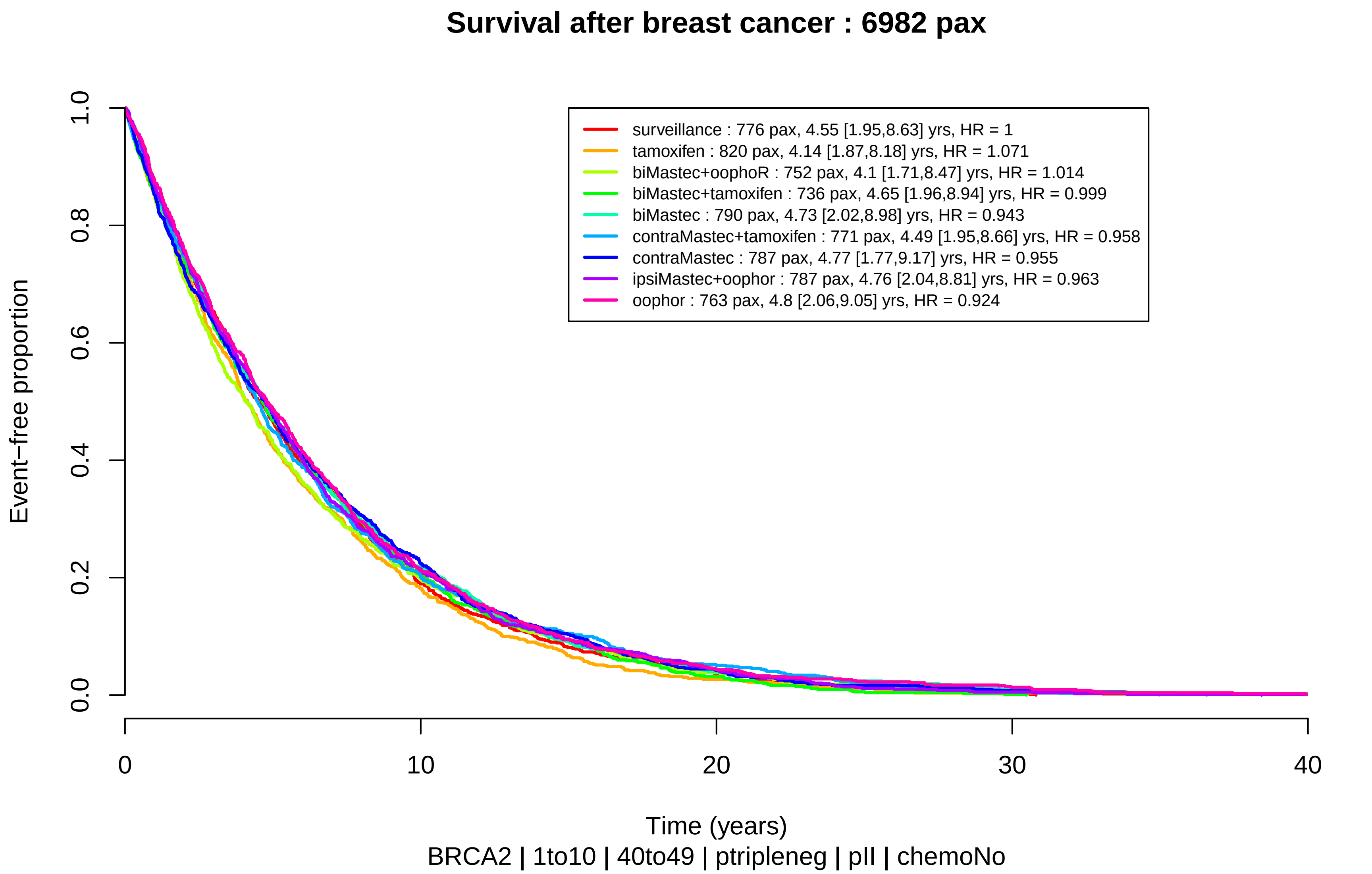

Survival after breast cancer : 6982 pax
Event−free proportion
0.0
0.2
0.4
0.6
0.8
1.0
0
10
20
30
40
surveillance : 776 pax, 4.55 [1.95,8.63] yrs, HR = 1
tamoxifen : 820 pax, 4.14 [1.87,8.18] yrs, HR = 1.071
biMastec+oophoR : 752 pax, 4.1 [1.71,8.47] yrs, HR = 1.014
biMastec+tamoxifen : 736 pax, 4.65 [1.96,8.94] yrs, HR = 0.999
biMastec : 790 pax, 4.73 [2.02,8.98] yrs, HR = 0.943
contraMastec+tamoxifen : 771 pax, 4.49 [1.95,8.66] yrs, HR = 0.958
contraMastec : 787 pax, 4.77 [1.77,9.17] yrs, HR = 0.955
ipsiMastec+oophor : 787 pax, 4.76 [2.04,8.81] yrs, HR = 0.963
oophor : 763 pax, 4.8 [2.06,9.05] yrs, HR = 0.924
Time (years)
BRCA2 | 1to10 | 40to49 | ptripleneg | pII | chemoNo

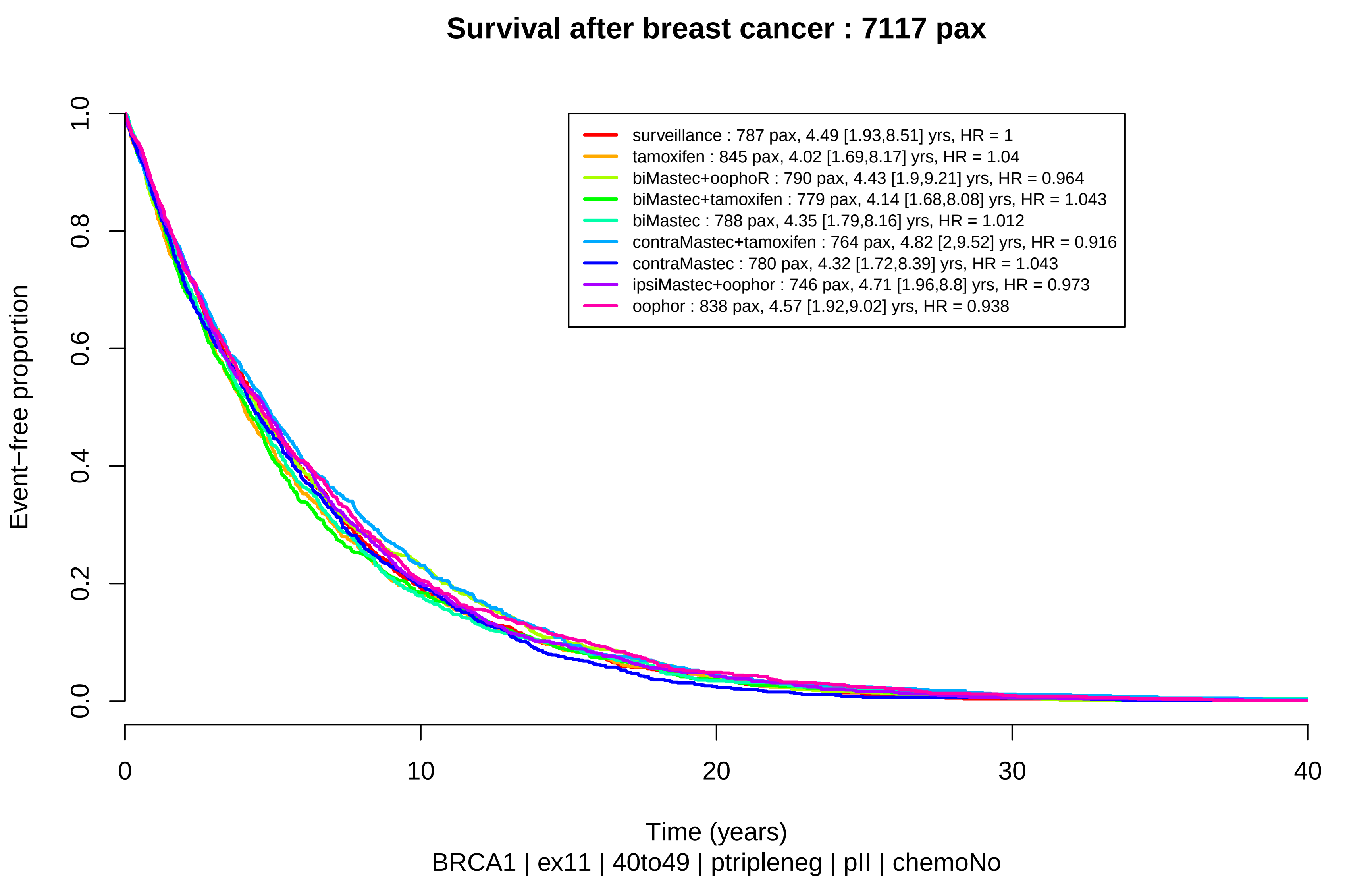

Survival after breast cancer : 7117 pax
surveillance : 787 pax, 4.49 [1.93,8.51] yrs, HR = 1
tamoxifen : 845 pax, 4.02 [1.69,8.17] yrs, HR = 1.04
biMastec+oophoR : 790 pax, 4.43 [1.9,9.21] yrs, HR = 0.964
biMastec+tamoxifen : 779 pax, 4.14 [1.68,8.08] yrs, HR = 1.043
biMastec : 788 pax, 4.35 [1.79,8.16] yrs, HR = 1.012
contraMastec+tamoxifen : 764 pax, 4.82 [2,9.52] yrs, HR = 0.916
contraMastec : 780 pax, 4.32 [1.72,8.39] yrs, HR = 1.043
ipsiMastec+oophor : 746 pax, 4.71 [1.96,8.8] yrs, HR = 0.973
oophor : 838 pax, 4.57 [1.92,9.02] yrs, HR = 0.938
Event−free proportion
0.0
0.2
0.4
0.6
0.8
1.0
0
10
20
30
40
Time (years)
BRCA1 | ex11 | 40to49 | ptripleneg | pII | chemoNo

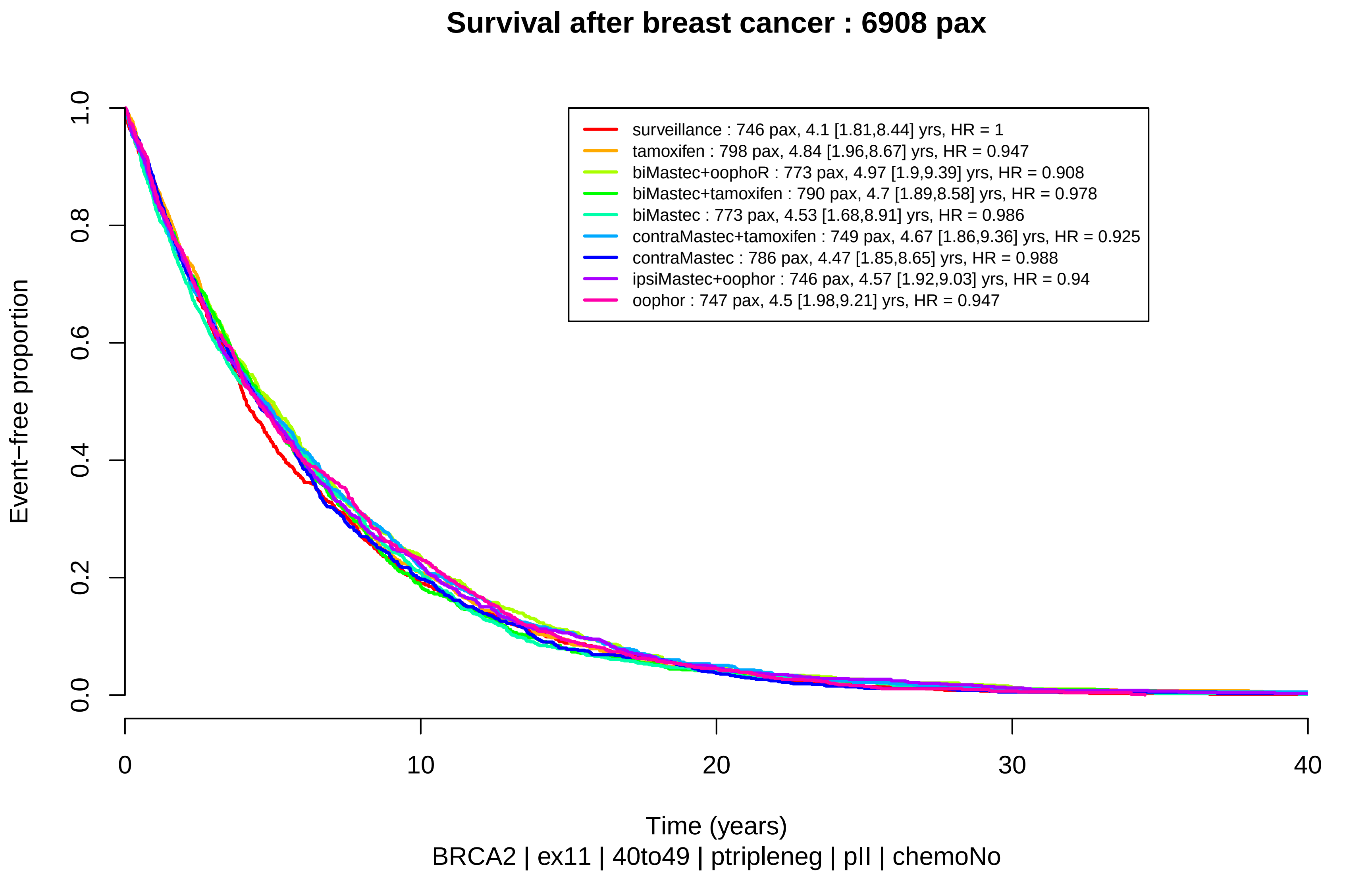

Survival after breast cancer : 6908 pax
Event−free proportion
1.0
0.8
0.6
0.4
0.2
0.0
0
10
20
30
40
Time (years)
BRCA2 | ex11 | 40to49 | ptripleneg | pII | chemoNo
surveillance : 746 pax, 4.1 [1.81,8.44] yrs, HR = 1
tamoxifen : 798 pax, 4.84 [1.96,8.67] yrs, HR = 0.947
biMastec+oophoR : 773 pax, 4.97 [1.9,9.39] yrs, HR = 0.908
biMastec+tamoxifen : 790 pax, 4.7 [1.89,8.58] yrs, HR = 0.978
biMastec : 773 pax, 4.53 [1.68,8.91] yrs, HR = 0.986
contraMastec+tamoxifen : 749 pax, 4.67 [1.86,9.36] yrs, HR = 0.925
contraMastec : 786 pax, 4.47 [1.85,8.65] yrs, HR = 0.988
ipsiMastec+oophor : 746 pax, 4.57 [1.92,9.03] yrs, HR = 0.94
oophor : 747 pax, 4.5 [1.98,9.21] yrs, HR = 0.947

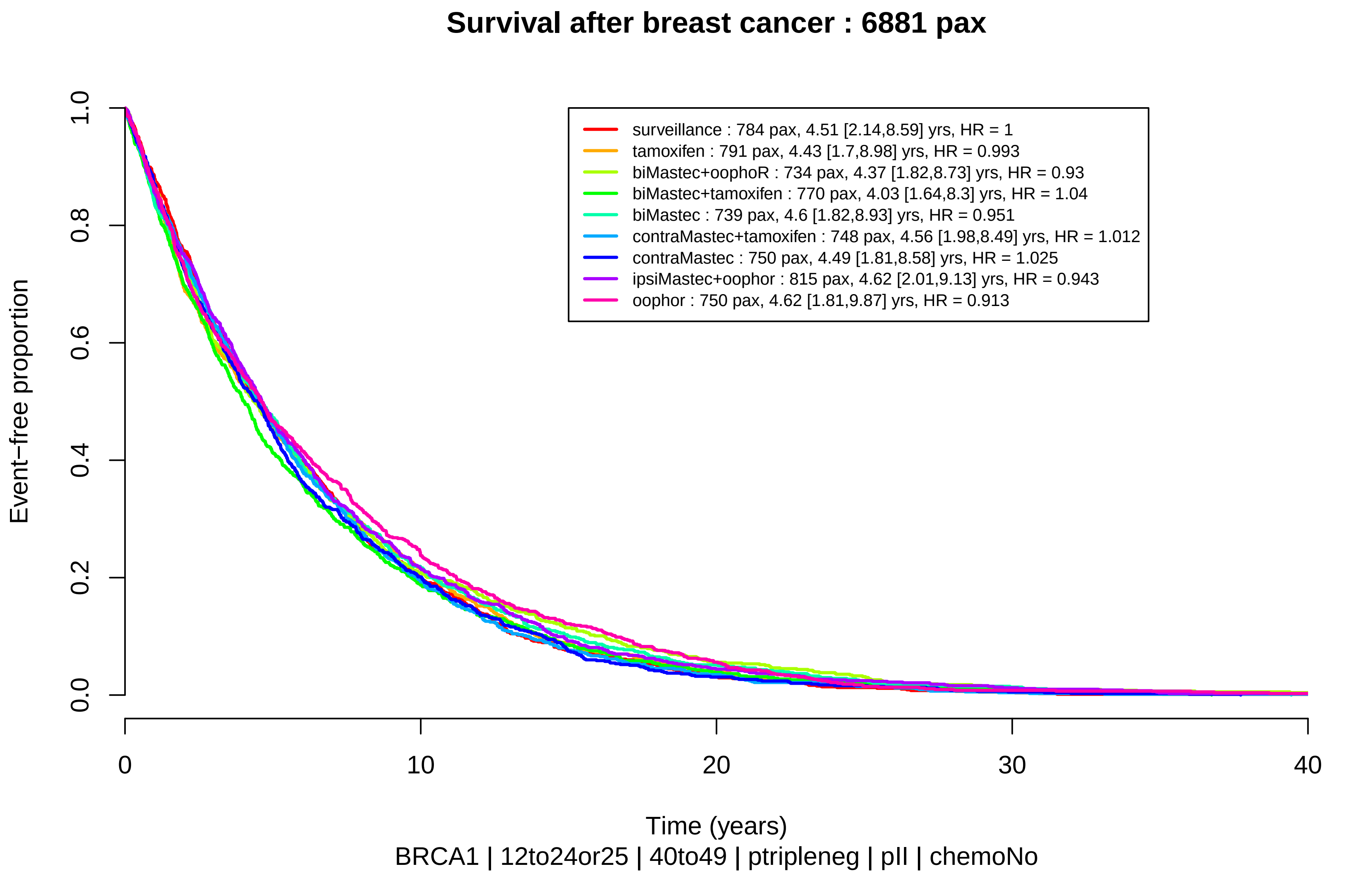

Survival after breast cancer : 6881 pax
surveillance : 784 pax, 4.51 [2.14,8.59] yrs, HR = 1
tamoxifen : 791 pax, 4.43 [1.7,8.98] yrs, HR = 0.993
biMastec+oophoR : 734 pax, 4.37 [1.82,8.73] yrs, HR = 0.93
biMastec+tamoxifen : 770 pax, 4.03 [1.64,8.3] yrs, HR = 1.04
biMastec : 739 pax, 4.6 [1.82,8.93] yrs, HR = 0.951
contraMastec+tamoxifen : 748 pax, 4.56 [1.98,8.49] yrs, HR = 1.012
contraMastec : 750 pax, 4.49 [1.81,8.58] yrs, HR = 1.025
ipsiMastec+oophor : 815 pax, 4.62 [2.01,9.13] yrs, HR = 0.943
oophor : 750 pax, 4.62 [1.81,9.87] yrs, HR = 0.913
Event−free proportion
0.0
0.2
0.4
0.6
0.8
1.0
0
10
20
30
40
Time (years)
BRCA1 | 12to24or25 | 40to49 | ptripleneg | pII | chemoNo

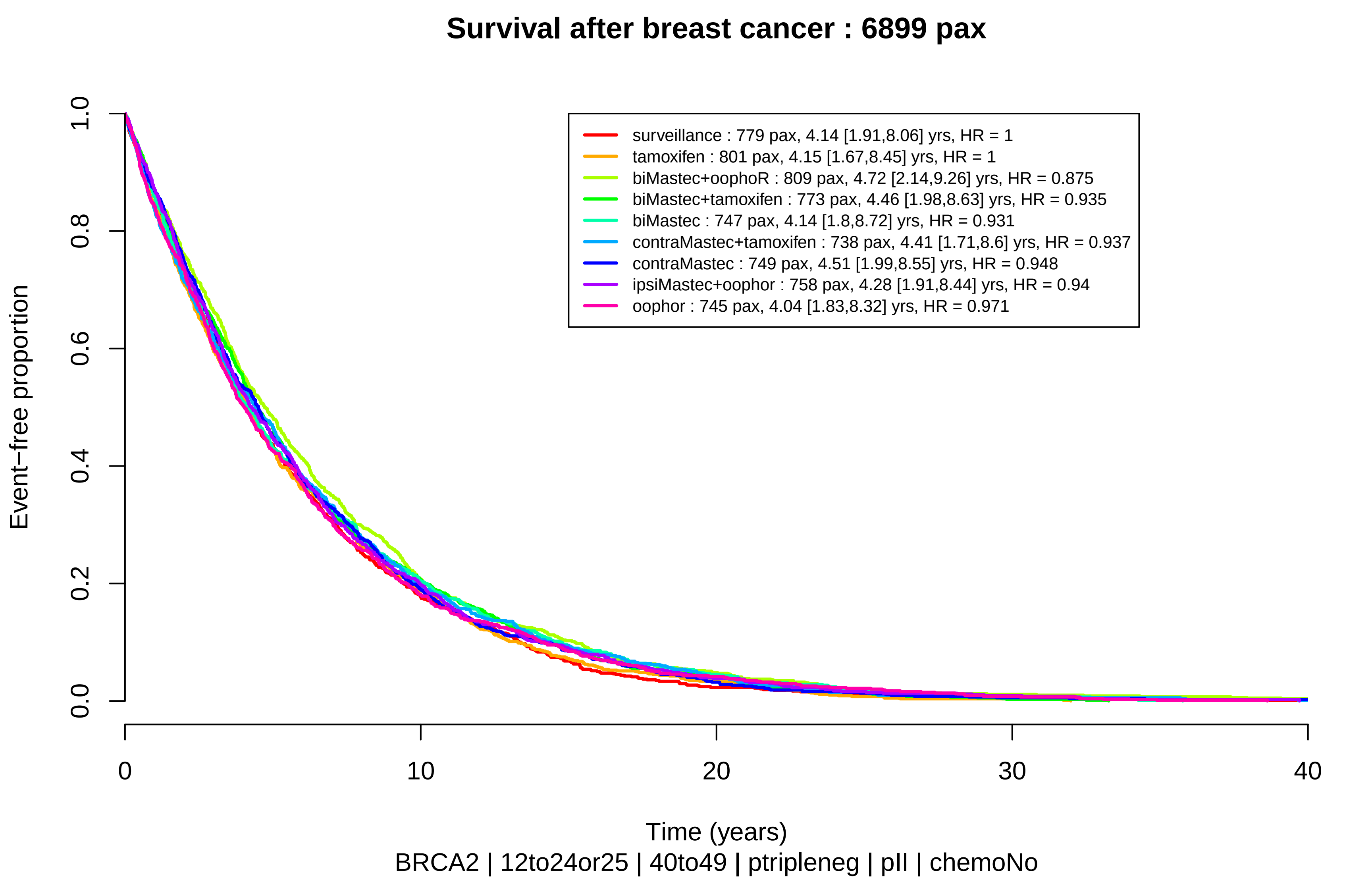

Survival after breast cancer : 6899 pax
Event−free proportion
0.0
0.2
0.4
0.6
0.8
1.0
0
10
20
30
40
Time (years)
BRCA2 | 12to24or25 | 40to49 | ptripleneg | pII | chemoNo
surveillance : 779 pax, 4.14 [1.91,8.06] yrs, HR = 1
tamoxifen : 801 pax, 4.15 [1.67,8.45] yrs, HR = 1
biMastec+oophoR : 809 pax, 4.72 [2.14,9.26] yrs, HR = 0.875
biMastec+tamoxifen : 773 pax, 4.46 [1.98,8.63] yrs, HR = 0.935
biMastec : 747 pax, 4.14 [1.8,8.72] yrs, HR = 0.931
contraMastec+tamoxifen : 738 pax, 4.41 [1.71,8.6] yrs, HR = 0.937
contraMastec : 749 pax, 4.51 [1.99,8.55] yrs, HR = 0.948
ipsiMastec+oophor : 758 pax, 4.28 [1.91,8.44] yrs, HR = 0.94
oophor : 745 pax, 4.04 [1.83,8.32] yrs, HR = 0.971

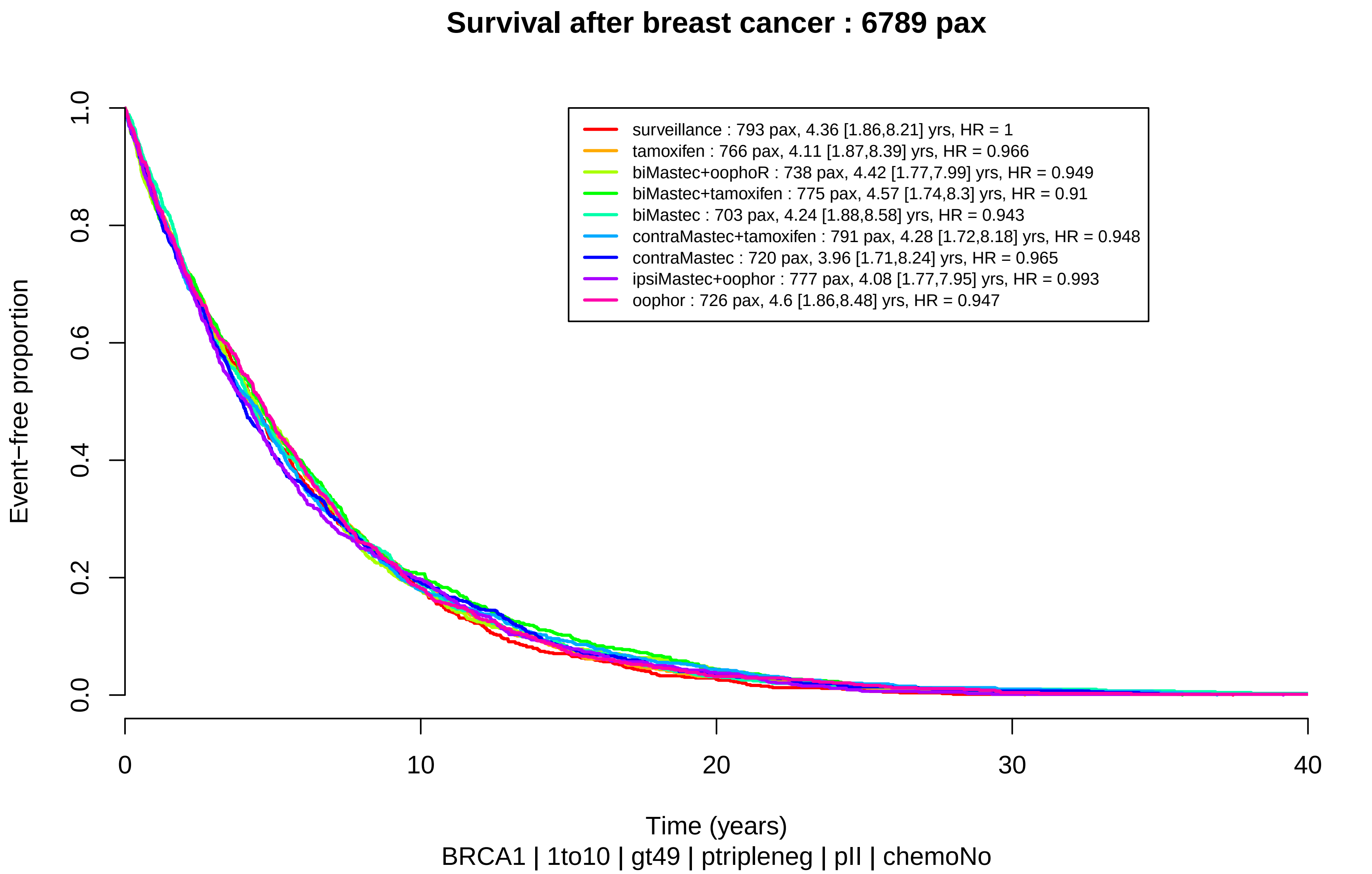
Survival after breast cancer : 6789 pax
surveillance : 793 pax, 4.36 [1.86,8.21] yrs, HR = 1
tamoxifen : 766 pax, 4.11 [1.87,8.39] yrs, HR = 0.966
biMastec+oophoR : 738 pax, 4.42 [1.77,7.99] yrs, HR = 0.949
biMastec+tamoxifen : 775 pax, 4.57 [1.74,8.3] yrs, HR = 0.91
biMastec : 703 pax, 4.24 [1.88,8.58] yrs, HR = 0.943
contraMastec+tamoxifen : 791 pax, 4.28 [1.72,8.18] yrs, HR = 0.948
contraMastec : 720 pax, 3.96 [1.71,8.24] yrs, HR = 0.965
ipsiMastec+oophor : 777 pax, 4.08 [1.77,7.95] yrs, HR = 0.993
oophor : 726 pax, 4.6 [1.86,8.48] yrs, HR = 0.947
Event−free proportion
0.0
0.2
0.4
0.6
0.8
1.0
0
10
20
30
40
Time (years)
BRCA1 | 1to10 | gt49 | ptripleneg | pII | chemoNo

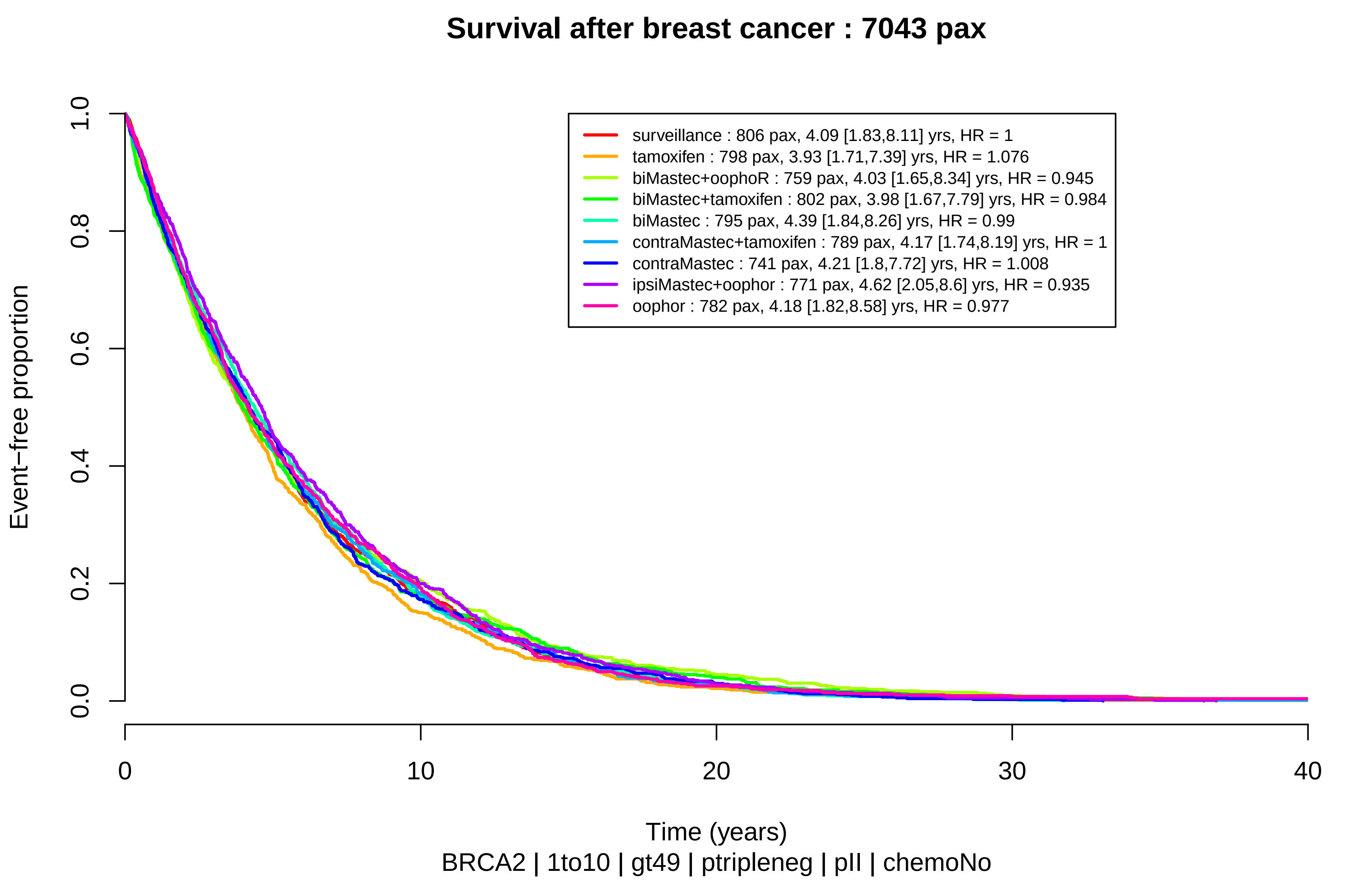
Survival after breast cancer : 7043 pax
Event−free proportion
0.0
0.2
0.4
0.6
0.8
1.0
0
10
20
30
40
Time (years)
BRCA2 | 1to10 | gt49 | ptripleneg | pII | chemoNo
surveillance : 806 pax, 4.09 [1.83,8.11] yrs, HR = 1
tamoxifen : 798 pax, 3.93 [1.71,7.39] yrs, HR = 1.076
biMastec+oophoR : 759 pax, 4.03 [1.65,8.34] yrs, HR = 0.945
biMastec+tamoxifen : 802 pax, 3.98 [1.67,7.79] yrs, HR = 0.984
biMastec : 795 pax, 4.39 [1.84,8.26] yrs, HR = 0.99
contraMastec+tamoxifen : 789 pax, 4.17 [1.74,8.19] yrs, HR = 1
contraMastec : 741 pax, 4.21 [1.8,7.72] yrs, HR = 1.008
ipsiMastec+oophor : 771 pax, 4.62 [2.05,8.6] yrs, HR = 0.935
oophor : 782 pax, 4.18 [1.82,8.58] yrs, HR = 0.977

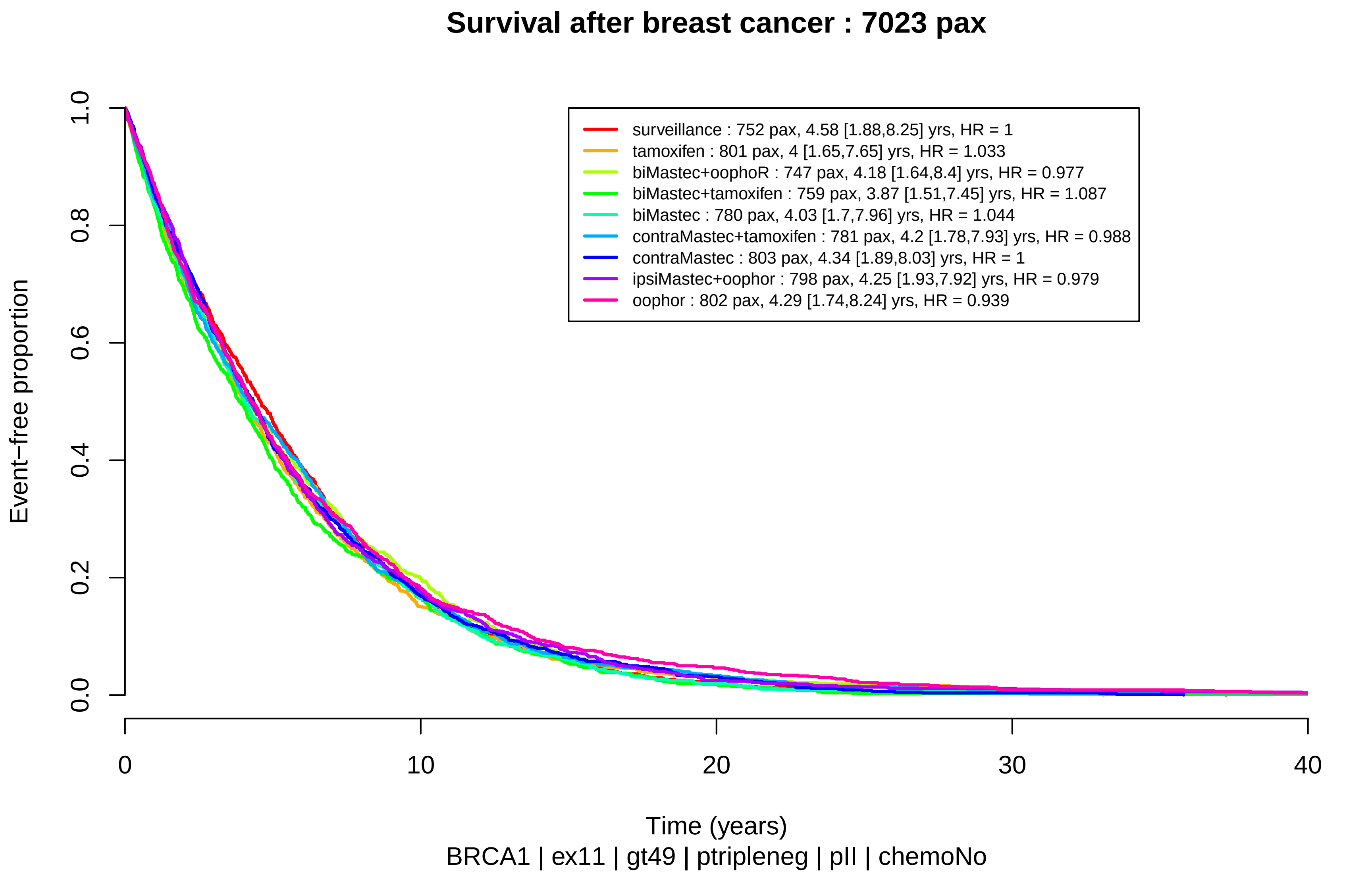

Survival after breast cancer : 7023 pax
surveillance : 752 pax, 4.58 [1.88,8.25] yrs, HR = 1
tamoxifen : 801 pax, 4 [1.65,7.65] yrs, HR = 1.033
biMastec+oophoR : 747 pax, 4.18 [1.64,8.4] yrs, HR = 0.977
biMastec+tamoxifen : 759 pax, 3.87 [1.51,7.45] yrs, HR = 1.087
biMastec : 780 pax, 4.03 [1.7,7.96] yrs, HR = 1.044
contraMastec+tamoxifen : 781 pax, 4.2 [1.78,7.93] yrs, HR = 0.988
contraMastec : 803 pax, 4.34 [1.89,8.03] yrs, HR = 1
ipsiMastec+oophor : 798 pax, 4.25 [1.93,7.92] yrs, HR = 0.979
oophor : 802 pax, 4.29 [1.74,8.24] yrs, HR = 0.939
Event−free proportion
0.0
0.2
0.4
0.6
0.8
1.0
0
10
20
30
40
Time (years)
BRCA1 | ex11 | gt49 | ptripleneg | pII | chemoNo

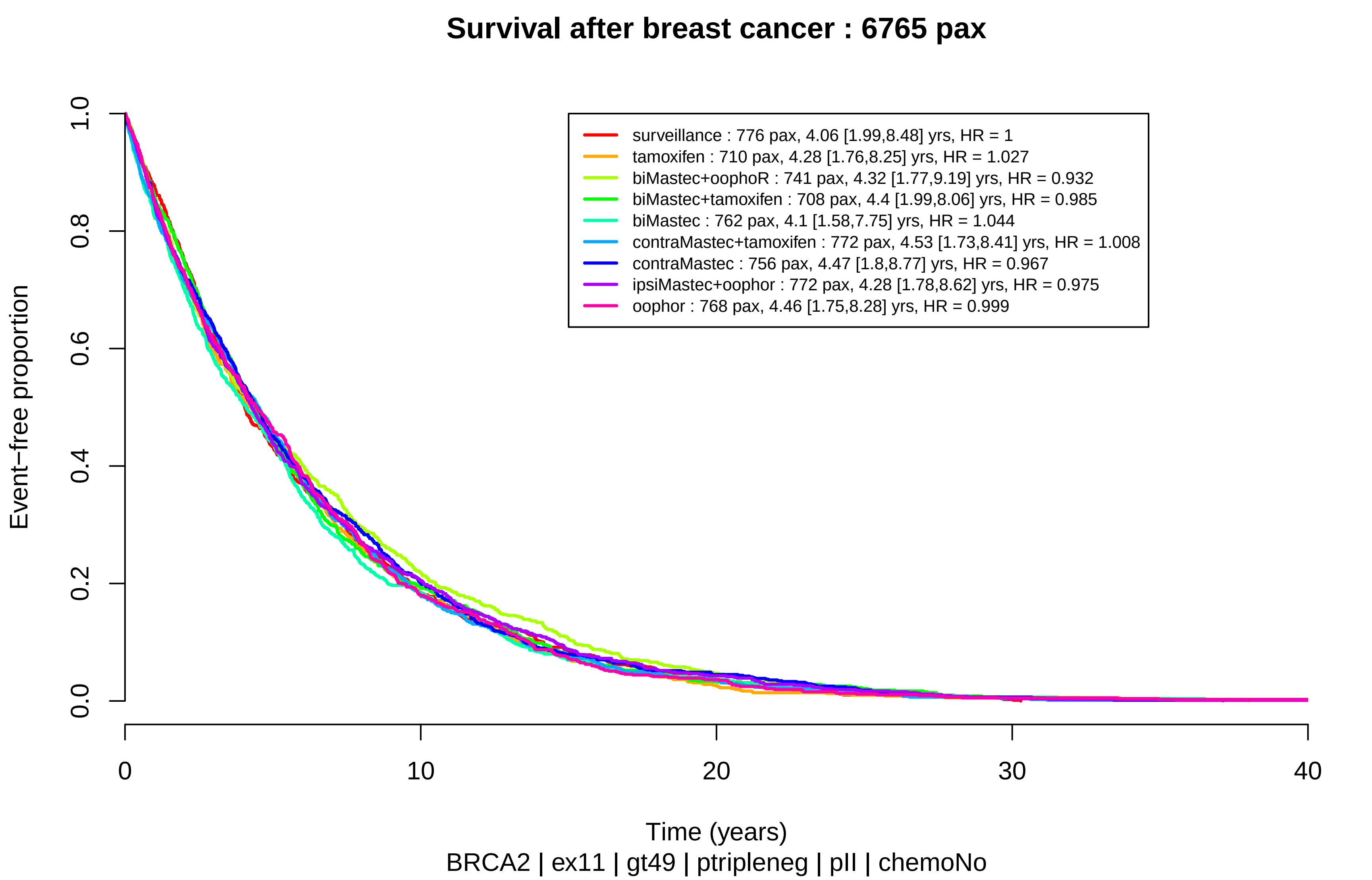

Survival after breast cancer : 6765 pax
Event−free proportion
0.0
0.2
0.4
0.6
0.8
1.0
0
10
20
30
40
Time (years)
BRCA2 | ex11 | gt49 | ptripleneg | pII | chemoNo
surveillance : 776 pax, 4.06 [1.99,8.48] yrs, HR = 1
tamoxifen : 710 pax, 4.28 [1.76,8.25] yrs, HR = 1.027
biMastec+oophoR : 741 pax, 4.32 [1.77,9.19] yrs, HR = 0.932
biMastec+tamoxifen : 708 pax, 4.4 [1.99,8.06] yrs, HR = 0.985
biMastec : 762 pax, 4.1 [1.58,7.75] yrs, HR = 1.044
contraMastec+tamoxifen : 772 pax, 4.53 [1.73,8.41] yrs, HR = 1.008
contraMastec : 756 pax, 4.47 [1.8,8.77] yrs, HR = 0.967
ipsiMastec+oophor : 772 pax, 4.28 [1.78,8.62] yrs, HR = 0.975
oophor : 768 pax, 4.46 [1.75,8.28] yrs, HR = 0.999

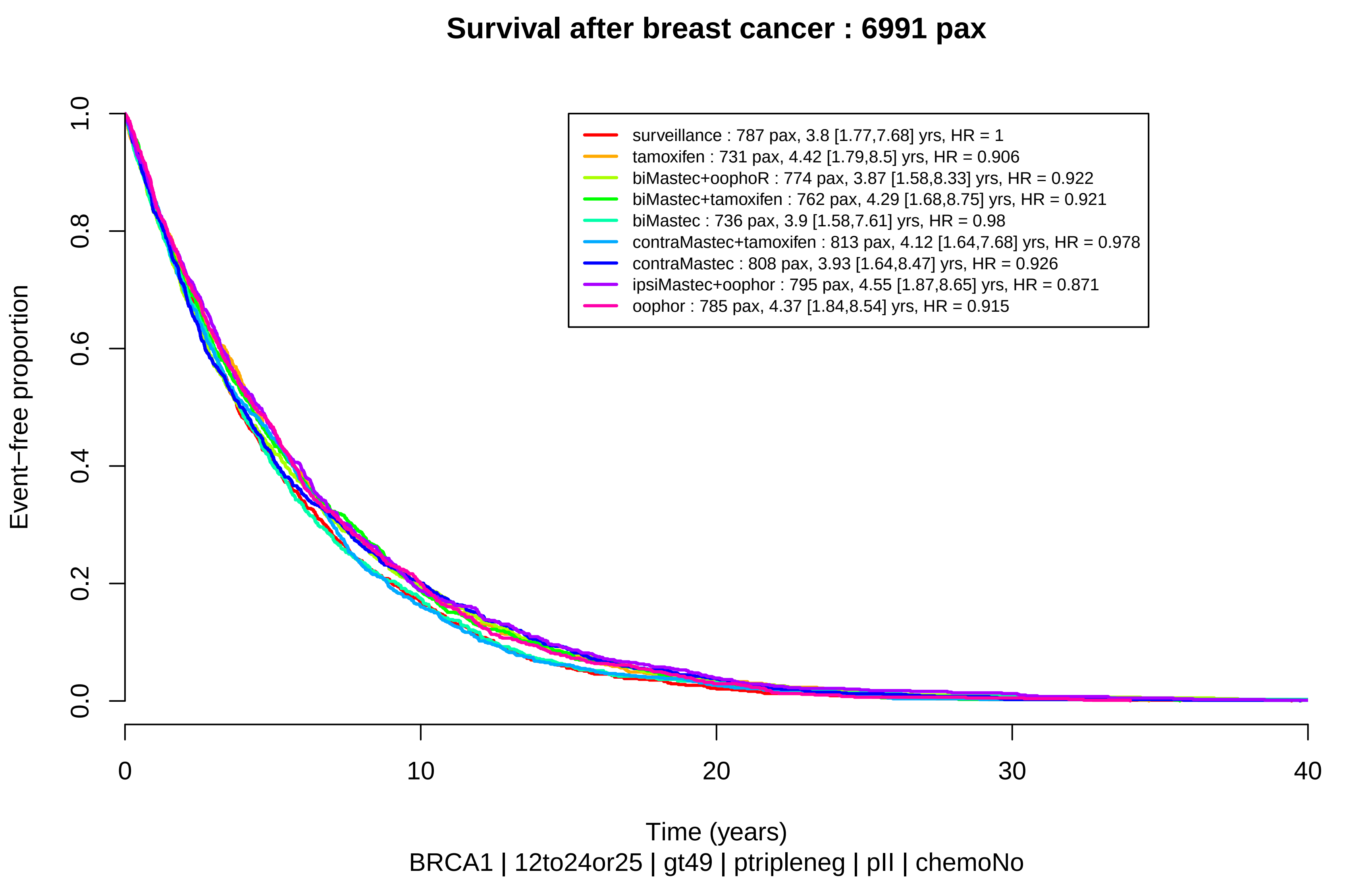
Survival after breast cancer : 6991 pax
surveillance : 787 pax, 3.8 [1.77,7.68] yrs, HR = 1
tamoxifen : 731 pax, 4.42 [1.79,8.5] yrs, HR = 0.906
biMastec+oophoR : 774 pax, 3.87 [1.58,8.33] yrs, HR = 0.922
biMastec+tamoxifen : 762 pax, 4.29 [1.68,8.75] yrs, HR = 0.921
biMastec : 736 pax, 3.9 [1.58,7.61] yrs, HR = 0.98
contraMastec+tamoxifen : 813 pax, 4.12 [1.64,7.68] yrs, HR = 0.978
contraMastec : 808 pax, 3.93 [1.64,8.47] yrs, HR = 0.926
ipsiMastec+oophor : 795 pax, 4.55 [1.87,8.65] yrs, HR = 0.871
oophor : 785 pax, 4.37 [1.84,8.54] yrs, HR = 0.915
Event−free proportion
0.0
0.2
0.4
0.6
0.8
1.0
0
10
20
30
40
Time (years)
BRCA1 | 12to24or25 | gt49 | ptripleneg | pII | chemoNo

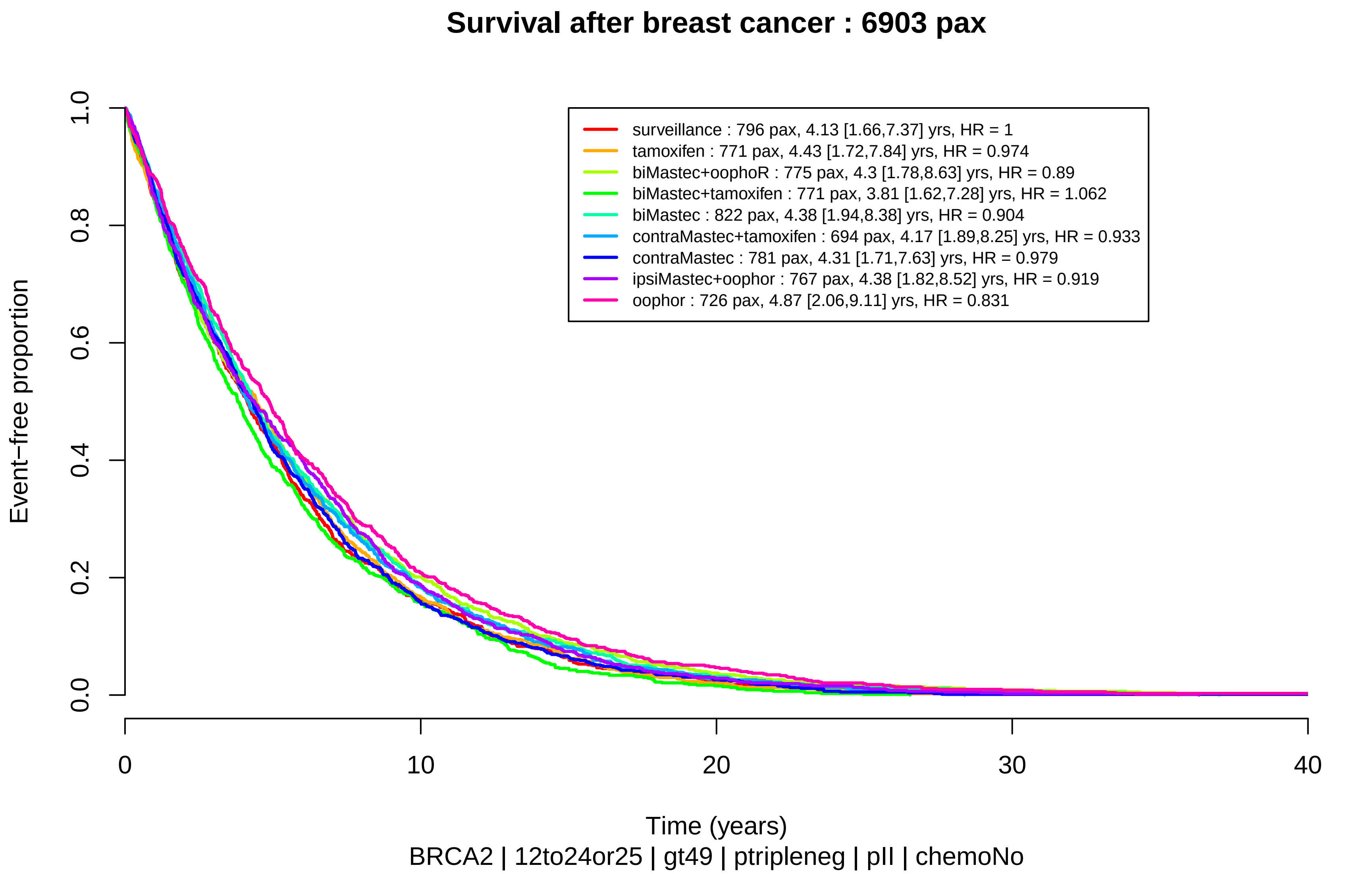

Survival after breast cancer : 6903 pax
surveillance : 796 pax, 4.13 [1.66,7.37] yrs, HR = 1
tamoxifen : 771 pax, 4.43 [1.72,7.84] yrs, HR = 0.974
biMastec+oophoR : 775 pax, 4.3 [1.78,8.63] yrs, HR = 0.89
biMastec+tamoxifen : 771 pax, 3.81 [1.62,7.28] yrs, HR = 1.062
biMastec : 822 pax, 4.38 [1.94,8.38] yrs, HR = 0.904
contraMastec+tamoxifen : 694 pax, 4.17 [1.89,8.25] yrs, HR = 0.933
contraMastec : 781 pax, 4.31 [1.71,7.63] yrs, HR = 0.979
ipsiMastec+oophor : 767 pax, 4.38 [1.82,8.52] yrs, HR = 0.919
oophor : 726 pax, 4.87 [2.06,9.11] yrs, HR = 0.831
Event−free proportion
0.0
0.2
0.4
0.6
0.8
1.0
0
10
20
30
40
Time (years)
BRCA2 | 12to24or25 | gt49 | ptripleneg | pII | chemoNo

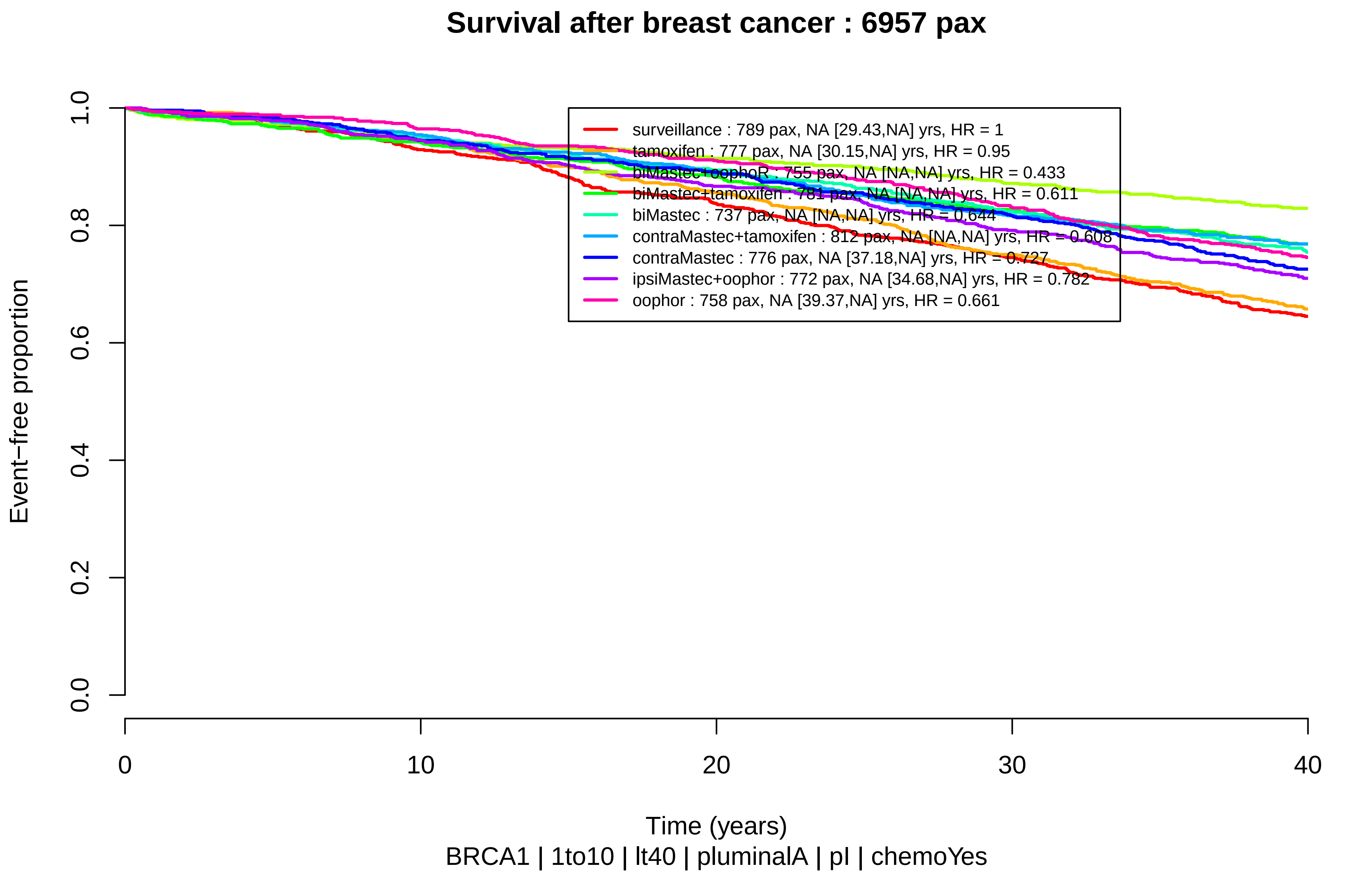

Survival after breast cancer : 6957 pax
surveillance : 789 pax, NA [29.43,NA] yrs, HR = 1
tamoxifen : 777 pax, NA [30.15,NA] yrs, HR = 0.95
biMastec+oophoR : 755 pax, NA [NA,NA] yrs, HR = 0.433
biMastec+tamoxifen : 781 pax, NA [NA,NA] yrs, HR = 0.611
biMastec : 737 pax, NA [NA,NA] yrs, HR = 0.644
contraMastec+tamoxifen : 812 pax, NA [NA,NA] yrs, HR = 0.608
contraMastec : 776 pax, NA [37.18,NA] yrs, HR = 0.727
ipsiMastec+oophor : 772 pax, NA [34.68,NA] yrs, HR = 0.782
oophor : 758 pax, NA [39.37,NA] yrs, HR = 0.661
Event−free proportion
0.0
0.2
0.4
0.6
0.8
1.0
0
10
20
30
40
Time (years)
BRCA1 | 1to10 | lt40 | pluminalA | pl | chemoYes

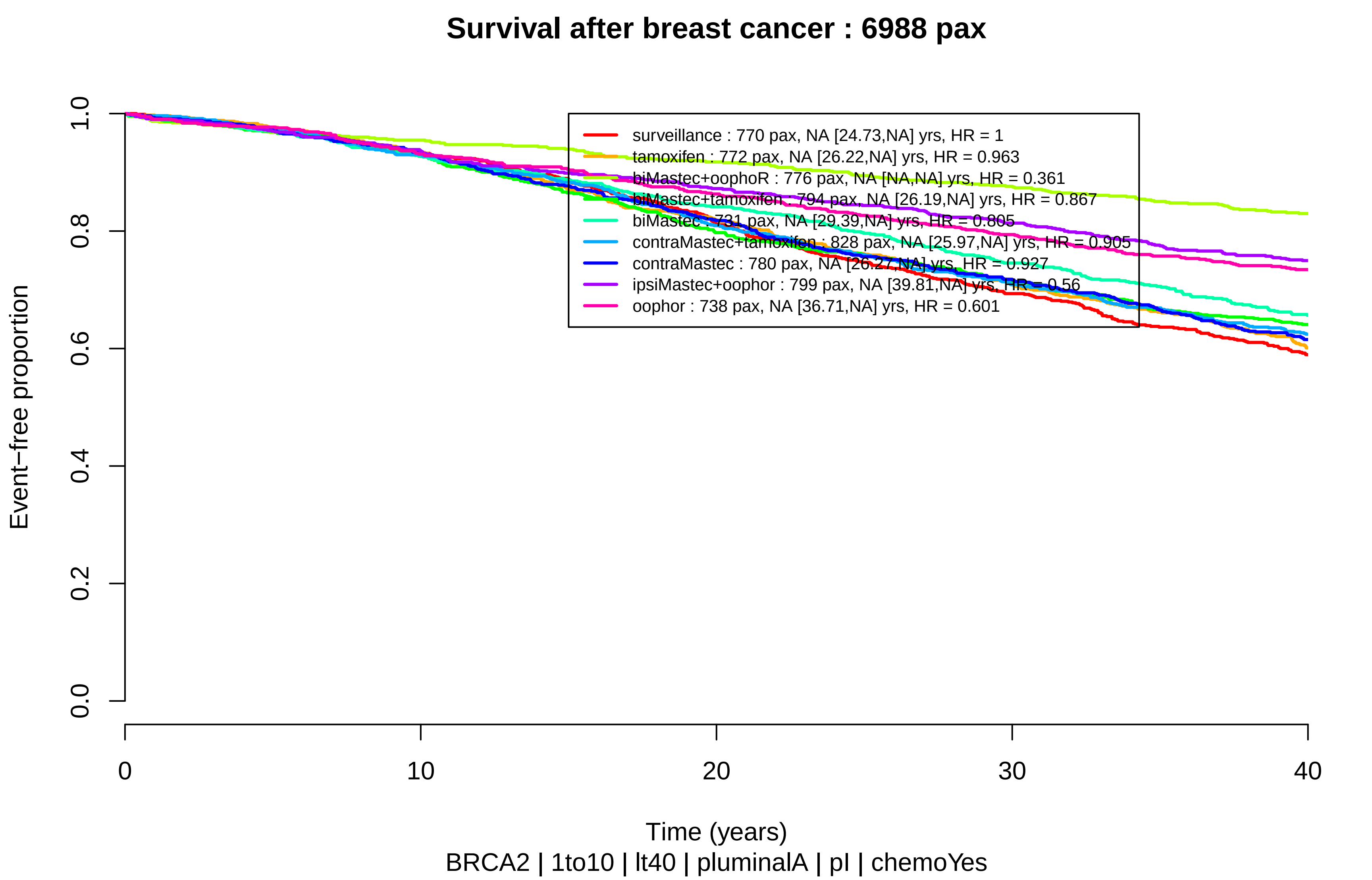

Survival after breast cancer : 6988 pax
Event−free proportion
0.0
0.2
0.4
0.6
0.8
1.0
0
10
20
30
40
Time (years)
BRCA2 | 1to10 | lt40 | pluminalA | pI | chemoYes
surveillance : 770 pax, NA [24.73,NA] yrs, HR = 1
tamoxifen : 772 pax, NA [26.22,NA] yrs, HR = 0.963
biMastec+oophoR : 776 pax, NA [NA,NA] yrs, HR = 0.361
biMastec+tamoxifen : 794 pax, NA [26.19,NA] yrs, HR = 0.867
biMastec : 731 pax, NA [29.39,NA] yrs, HR = 0.805
contraMastec+tamoxifen : 828 pax, NA [25.97,NA] yrs, HR = 0.905
contraMastec : 780 pax, NA [26.27,NA] yrs, HR = 0.927
ipsiMastec+oophor : 799 pax, NA [39.81,NA] yrs, HR = 0.56
oophor : 738 pax, NA [36.71,NA] yrs, HR = 0.601

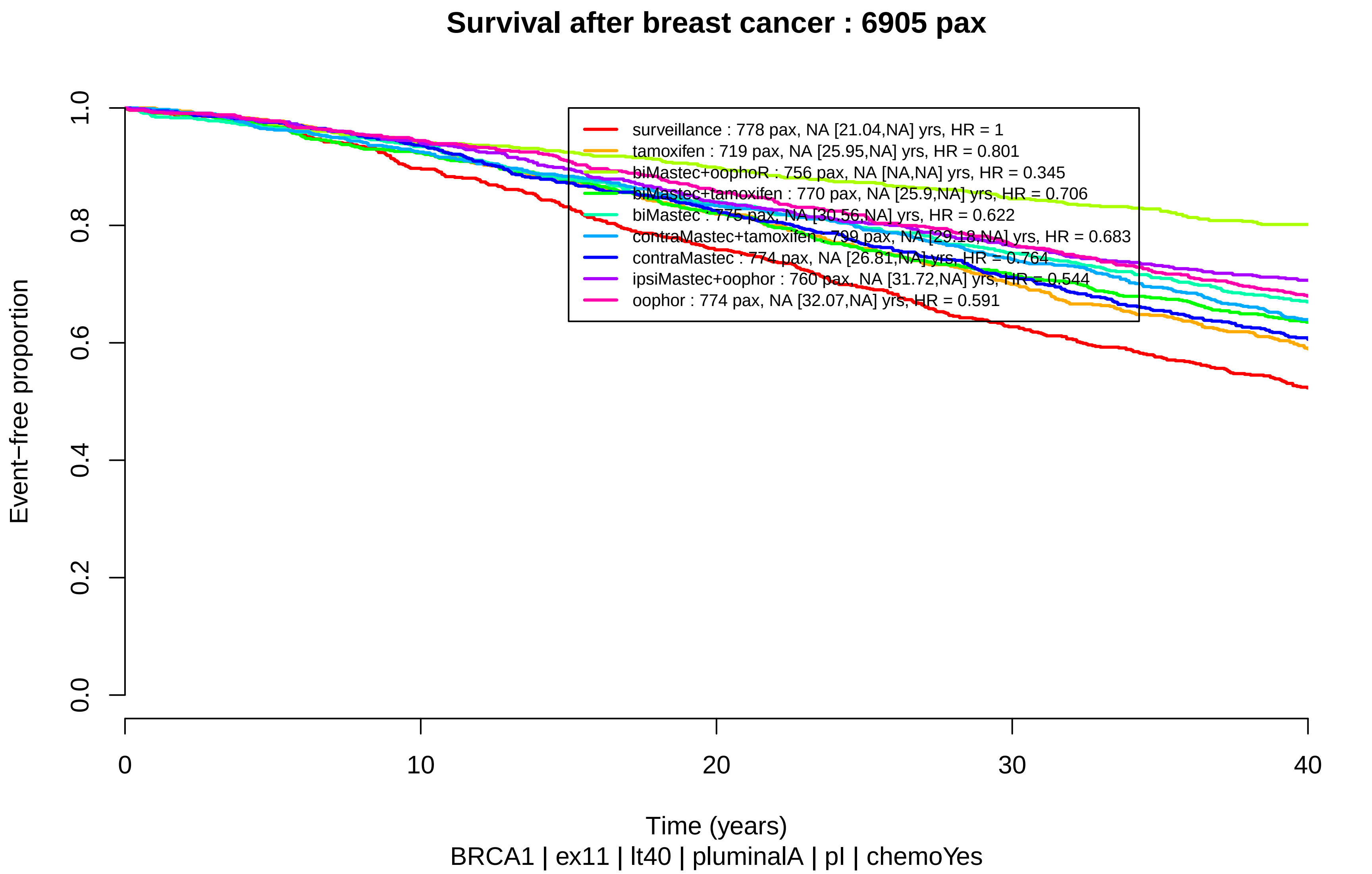

Survival after breast cancer : 6905 pax
surveillance : 778 pax, NA [21.04,NA] yrs, HR = 1
tamoxifen : 719 pax, NA [25.95,NA] yrs, HR = 0.801
biMastec+oophoR : 756 pax, NA [NA,NA] yrs, HR = 0.345
biMastec+tamoxifen : 770 pax, NA [25.9,NA] yrs, HR = 0.706
biMastec : 775 pax, NA [30.56,NA] yrs, HR = 0.622
contraMastec+tamoxifen : 799 pax, NA [29.18,NA] yrs, HR = 0.683
contraMastec : 774 pax, NA [26.81,NA] yrs, HR = 0.764
ipsiMastec+oophor : 760 pax, NA [31.72,NA] yrs, HR = 0.544
oophor : 774 pax, NA [32.07,NA] yrs, HR = 0.591
Event−free proportion
0.0
0.2
0.4
0.6
0.8
1.0
0
10
20
30
40
Time (years)
BRCA1 | ex11 | lt40 | pluminalA | pl | chemoYes

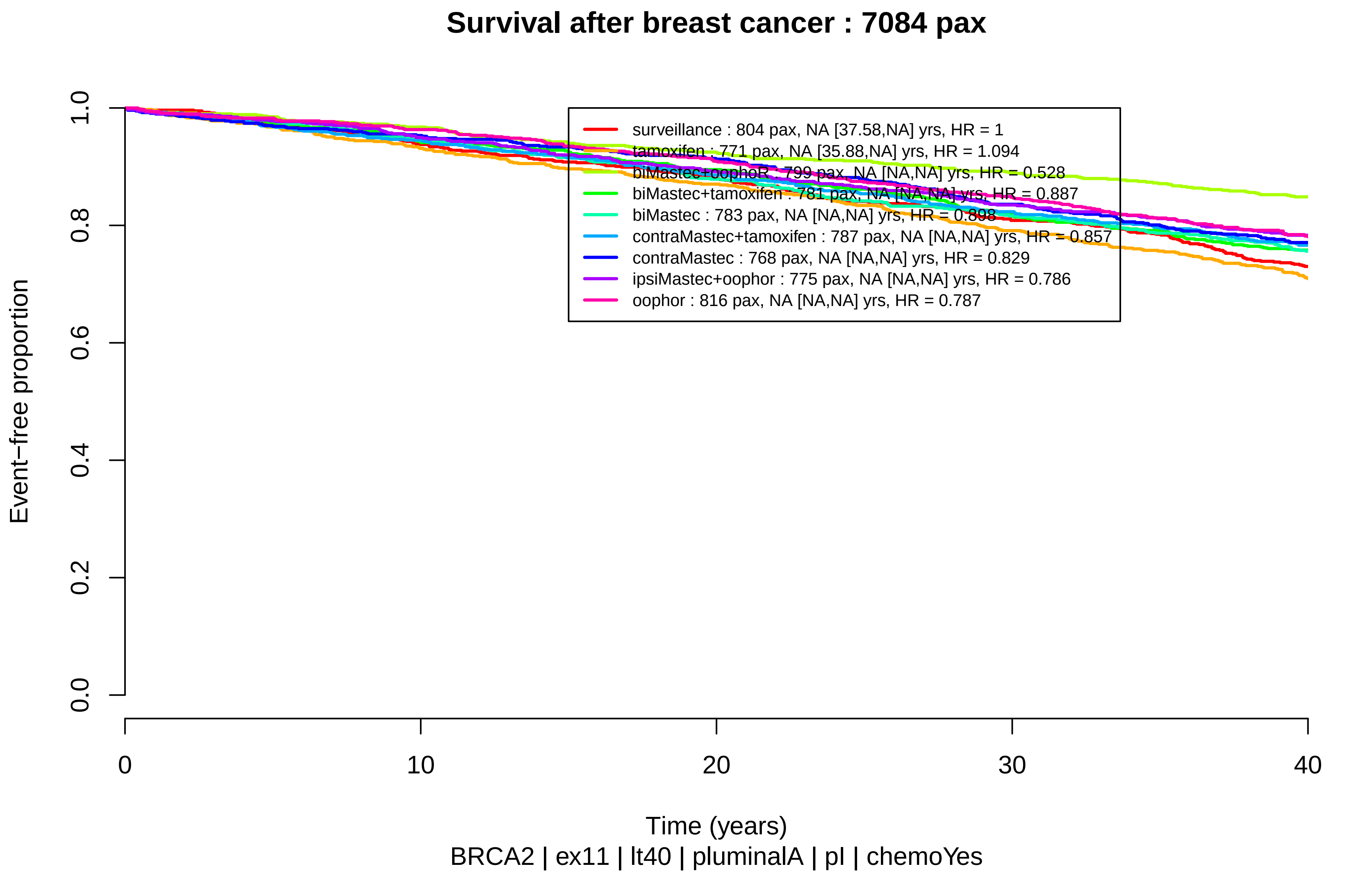

Survival after breast cancer : 7084 pax
Event−free proportion
0.0
0.2
0.4
0.6
0.8
1.0
0
10
20
30
40
Time (years)
BRCA2 | ex11 | lt40 | pluminalA | pl | chemoYes
surveillance : 804 pax, NA [37.58,NA] yrs, HR = 1
tamoxifen : 771 pax, NA [35.88,NA] yrs, HR = 1.094
biMastec+oophoR : 799 pax, NA [NA,NA] yrs, HR = 0.528
biMastec+tamoxifen : 781 pax, NA [NA,NA] yrs, HR = 0.887
biMastec : 783 pax, NA [NA,NA] yrs, HR = 0.898
contraMastec+tamoxifen : 787 pax, NA [NA,NA] yrs, HR = 0.857
contraMastec : 768 pax, NA [NA,NA] yrs, HR = 0.829
ipsiMastec+oophor : 775 pax, NA [NA,NA] yrs, HR = 0.786
oophor : 816 pax, NA [NA,NA] yrs, HR = 0.787

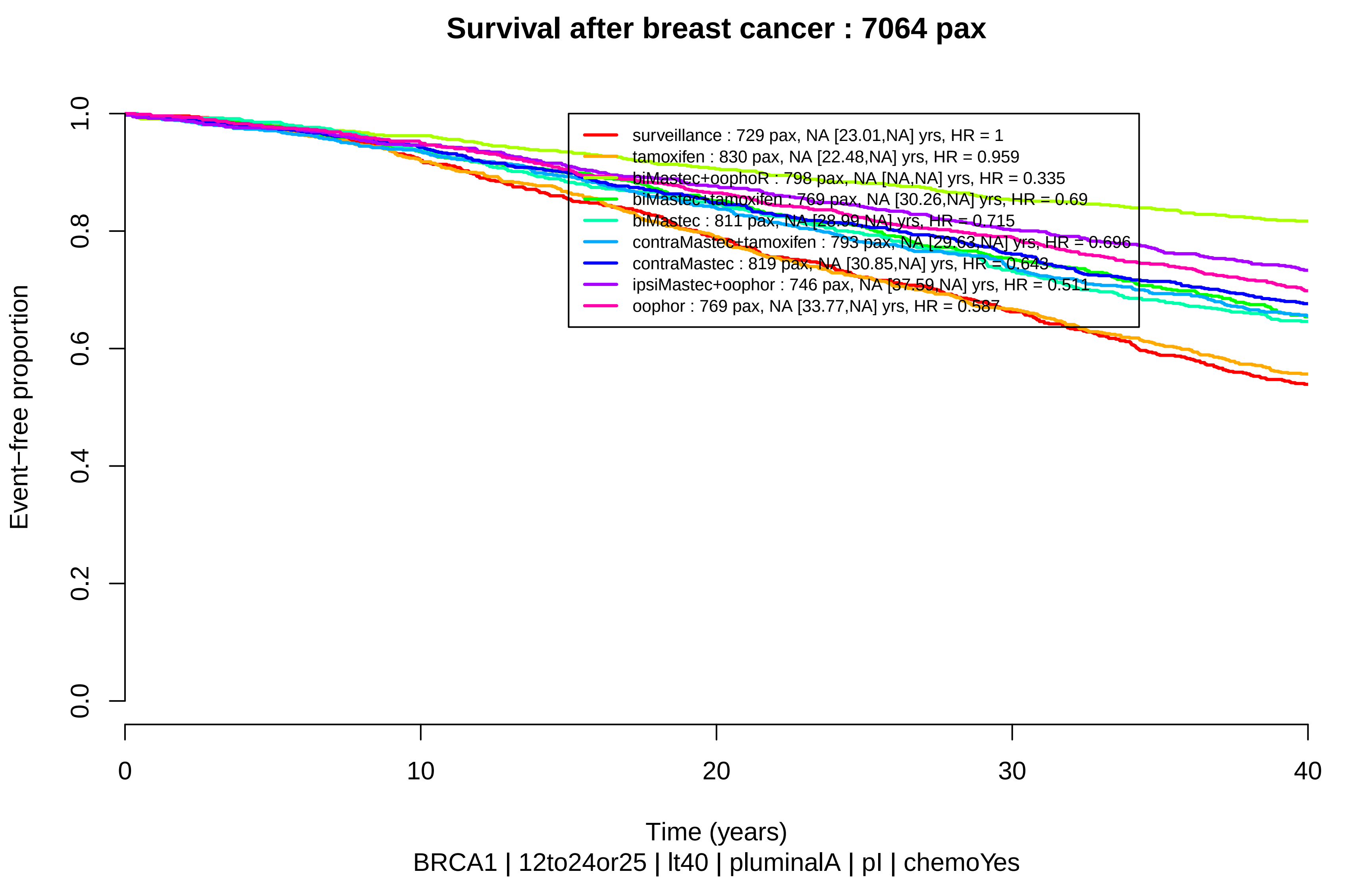
Survival after breast cancer : 7064 pax
surveillance : 729 pax, NA [23.01,NA] yrs, HR = 1
tamoxifen : 830 pax, NA [22.48,NA] yrs, HR = 0.959
biMastec+oophoR : 798 pax, NA [NA,NA] yrs, HR = 0.335
biMastec+tamoxifen : 769 pax, NA [30.26,NA] yrs, HR = 0.69
biMastec : 811 pax, NA [28.99,NA] yrs, HR = 0.715
contraMastec+tamoxifen : 793 pax, NA [29.63,NA] yrs, HR = 0.696
contraMastec : 819 pax, NA [30.85,NA] yrs, HR = 0.643
ipsiMastec+oophor : 746 pax, NA [37.59,NA] yrs, HR = 0.511
oophor : 769 pax, NA [33.77,NA] yrs, HR = 0.587
Event−free proportion
0.0
0.2
0.4
0.6
0.8
1.0
0
10
20
30
40
Time (years)
BRCA1 | 12to24or25 | lt40 | pluminalA | pl | chemoYes

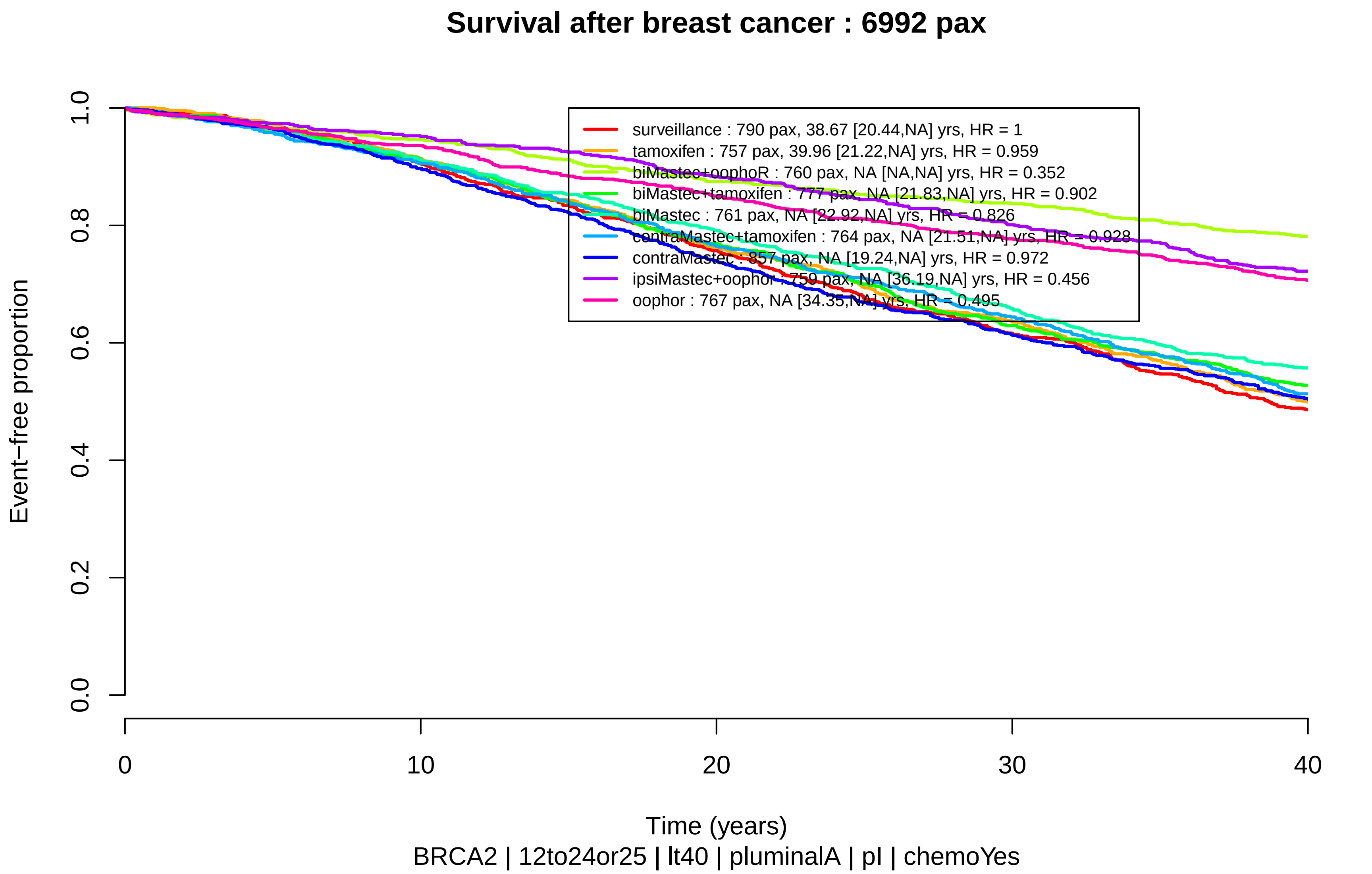
Survival after breast cancer : 6992 pax
Event−free proportion
0.0
0.2
0.4
0.6
0.8
1.0
0
10
20
30
40
Time (years)
BRCA2 | 12to24or25 | lt40 | pluminalA | pI | chemoYes
surveillance : 790 pax, 38.67 [20.44,NA] yrs, HR = 1
tamoxifen : 757 pax, 39.96 [21.22,NA] yrs, HR = 0.959
biMastec+oophoR : 760 pax, NA [NA,NA] yrs, HR = 0.352
biMastec+tamoxifen : 777 pax, NA [21.83,NA] yrs, HR = 0.902
biMastec : 761 pax, NA [22.92,NA] yrs, HR = 0.826
contraMastec+tamoxifen : 764 pax, NA [21.51,NA] yrs, HR = 0.928
contraMastec : 857 pax, NA [19.24,NA] yrs, HR = 0.972
ipsiMastec+oophor : 759 pax, NA [36.19,NA] yrs, HR = 0.456
oophor : 767 pax, NA [34.35,NA] yrs, HR = 0.495

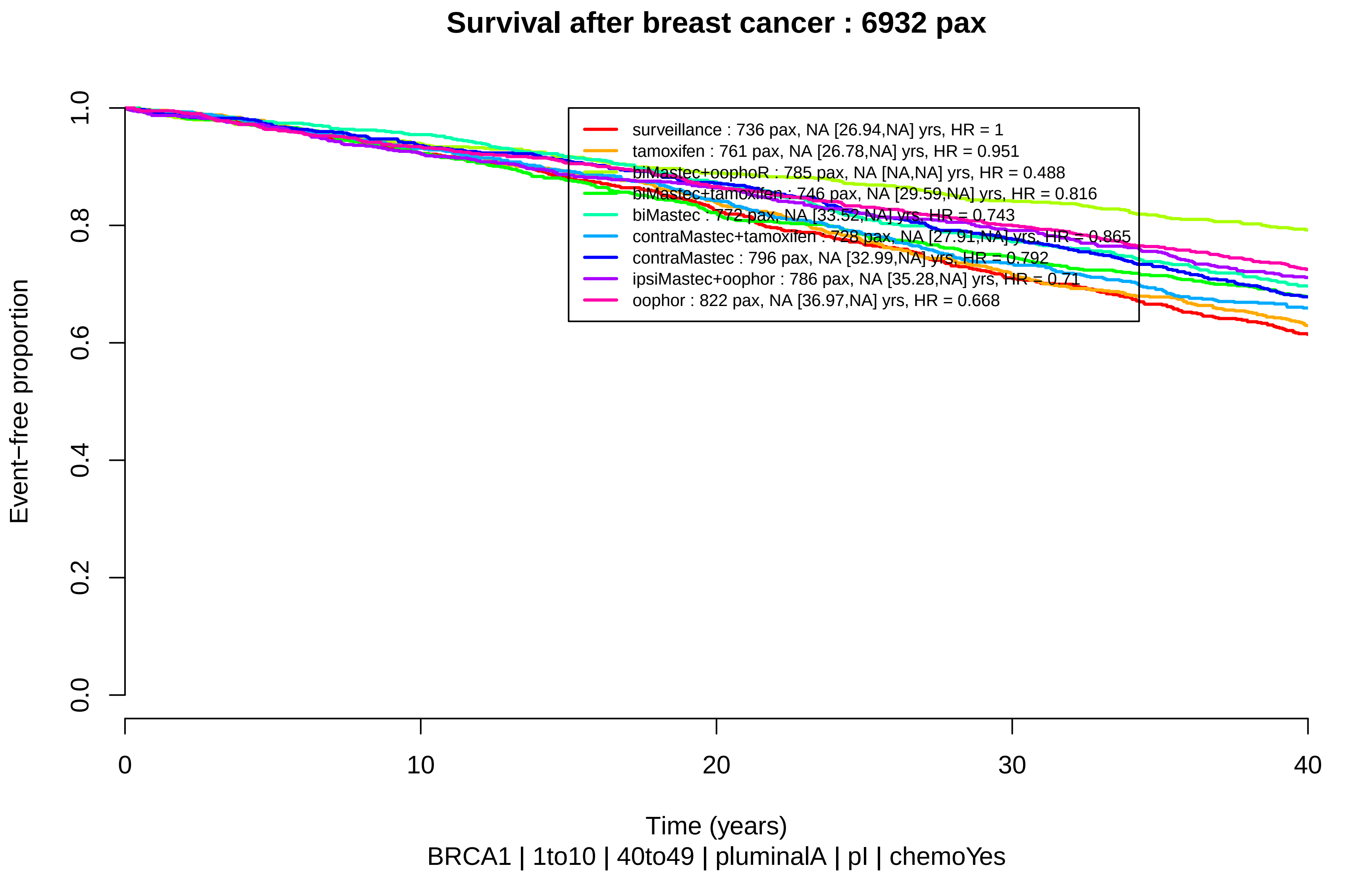

Survival after breast cancer : 6932 pax
surveillance : 736 pax, NA [26.94,NA] yrs, HR = 1
tamoxifen : 761 pax, NA [26.78,NA] yrs, HR = 0.951
biMastec+oophoR : 785 pax, NA [NA,NA] yrs, HR = 0.488
biMastec+tamoxifen : 746 pax, NA [29.59,NA] yrs, HR = 0.816
biMastec : 772 pax, NA [33.52,NA] yrs, HR = 0.743
contraMastec+tamoxifen : 728 pax, NA [27.91,NA] yrs, HR = 0.865
contraMastec : 796 pax, NA [32.99,NA] yrs, HR = 0.792
ipsiMastec+oophor : 786 pax, NA [35.28,NA] yrs, HR = 0.71
oophor : 822 pax, NA [36.97,NA] yrs, HR = 0.668
Event−free proportion
0.0
0.2
0.4
0.6
0.8
1.0
0
10
20
30
40
Time (years)
BRCA1 | 1to10 | 40to49 | pluminalA | pI | chemoYes

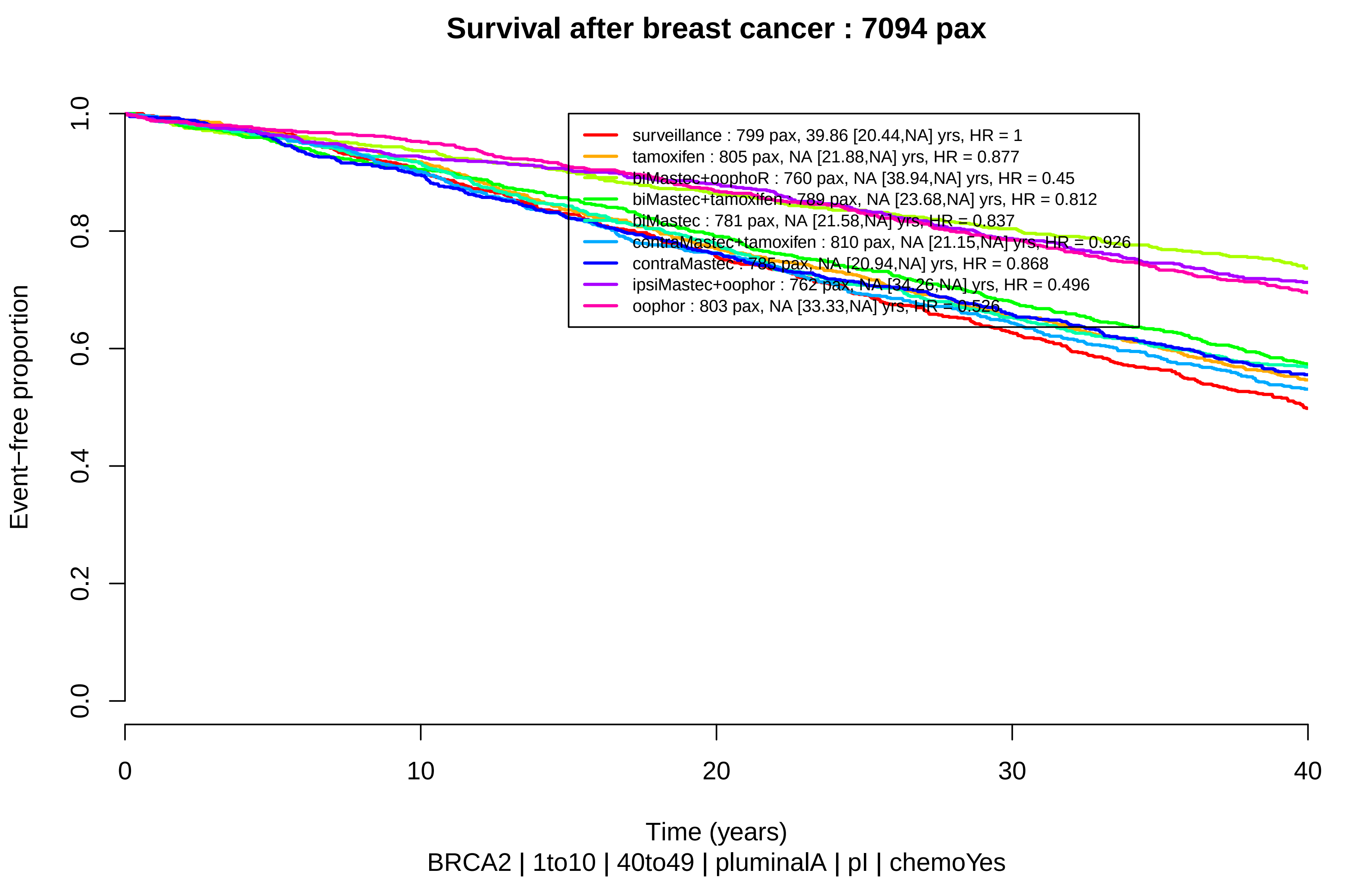

Survival after breast cancer : 7094 pax
Event−free proportion
0.0
0.2
0.4
0.6
0.8
1.0
0
10
20
30
40
Time (years)
BRCA2 | 1to10 | 40to49 | pluminalA | pI | chemoYes
surveillance : 799 pax, 39.86 [20.44,NA] yrs, HR = 1
tamoxifen : 805 pax, NA [21.88,NA] yrs, HR = 0.877
biMastec+oophoR : 760 pax, NA [38.94,NA] yrs, HR = 0.45
biMastec+tamoxifen : 789 pax, NA [23.68,NA] yrs, HR = 0.812
biMastec : 781 pax, NA [21.58,NA] yrs, HR = 0.837
contraMastec+tamoxifen : 810 pax, NA [21.15,NA] yrs, HR = 0.926
contraMastec : 785 pax, NA [20.94,NA] yrs, HR = 0.868
ipsiMastec+oophor : 762 pax, NA [34.26,NA] yrs, HR = 0.496
oophor : 803 pax, NA [33.33,NA] yrs, HR = 0.526

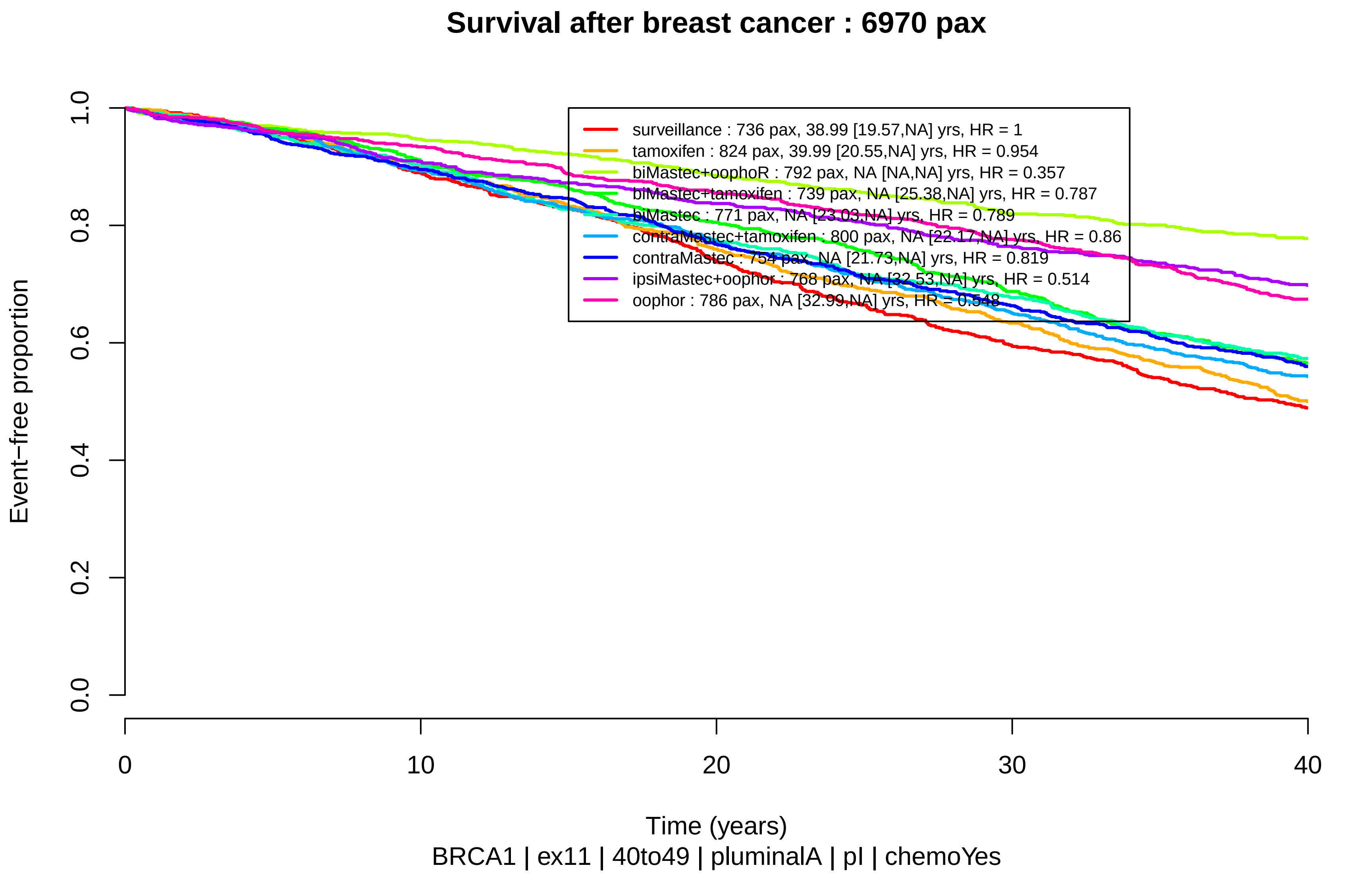
Survival after breast cancer : 6970 pax
surveillance : 736 pax, 38.99 [19.57,NA] yrs, HR = 1
tamoxifen : 824 pax, 39.99 [20.55,NA] yrs, HR = 0.954
biMastec+oophoR : 792 pax, NA [NA,NA] yrs, HR = 0.357
biMastec+tamoxifen : 739 pax, NA [25.38,NA] yrs, HR = 0.787
biMastec : 771 pax, NA [23.03,NA] yrs, HR = 0.789
contraMastec+tamoxifen : 800 pax, NA [22.17,NA] yrs, HR = 0.86
contraMastec : 754 pax, NA [21.73,NA] yrs, HR = 0.819
ipsiMastec+oophor : 768 pax, NA [32.53,NA] yrs, HR = 0.514
oophor : 786 pax, NA [32.99,NA] yrs, HR = 0.548
Event−free proportion
0.0
0.2
0.4
0.6
0.8
1.0
0
10
20
30
40
Time (years)
BRCA1 | ex11 | 40to49 | pluminalA | pI | chemoYes

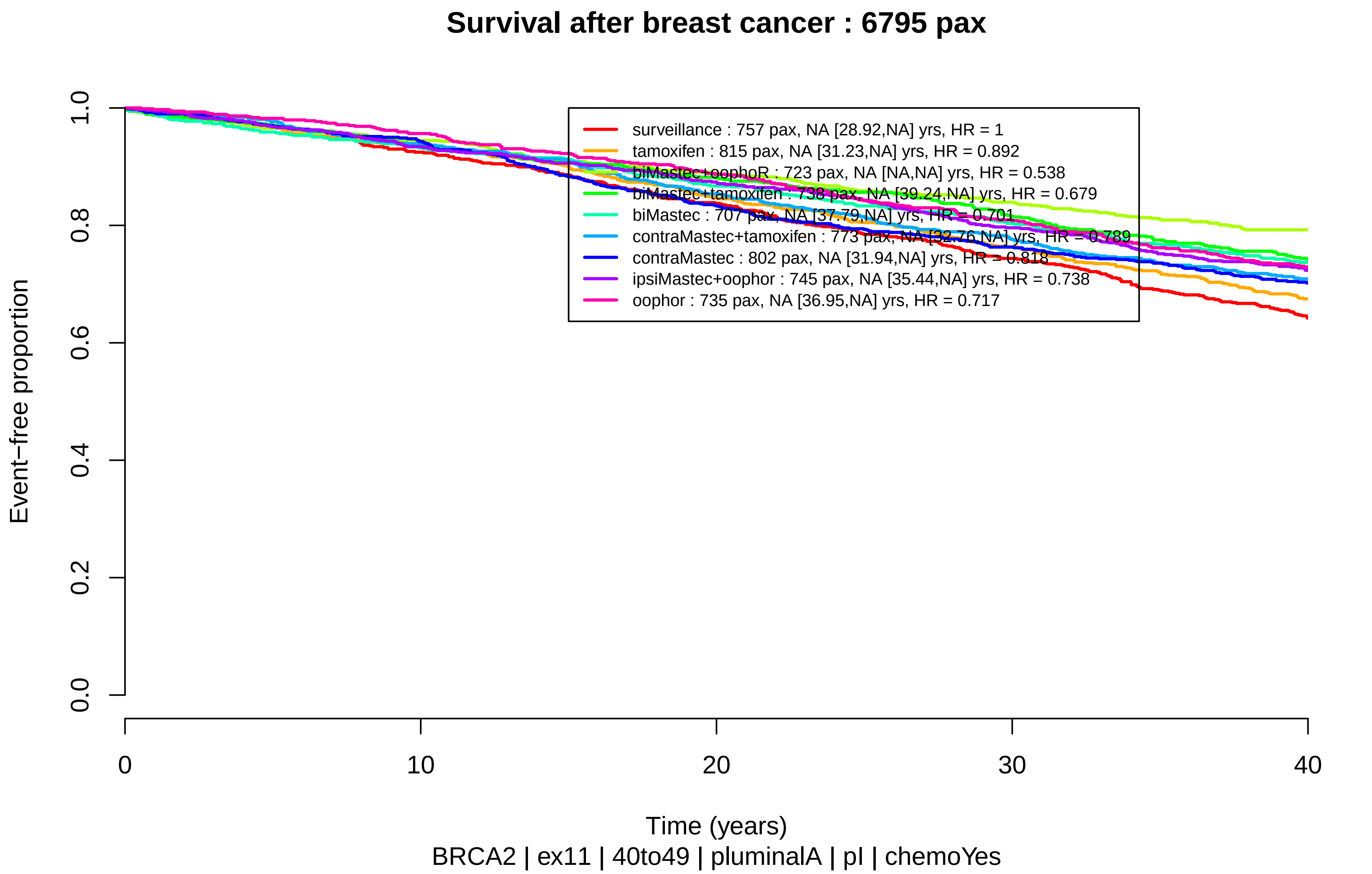
Survival after breast cancer : 6795 pax
Event−free proportion
0.0
0.2
0.4
0.6
0.8
1.0
0
10
20
30
40
Time (years)
BRCA2 | ex11 | 40to49 | pluminalA | pI | chemoYes
surveillance : 757 pax, NA [28.92,NA] yrs, HR = 1
tamoxifen : 815 pax, NA [31.23,NA] yrs, HR = 0.892
biMastec+oophoR : 723 pax, NA [NA,NA] yrs, HR = 0.538
biMastec+tamoxifen : 738 pax, NA [39.24,NA] yrs, HR = 0.679
biMastec : 707 pax, NA [37.79,NA] yrs, HR = 0.701
contraMastec+tamoxifen : 773 pax, NA [32.76,NA] yrs, HR = 0.789
contraMastec : 802 pax, NA [31.94,NA] yrs, HR = 0.818
ipsiMastec+oophor : 745 pax, NA [35.44,NA] yrs, HR = 0.738
oophor : 735 pax, NA [36.95,NA] yrs, HR = 0.717

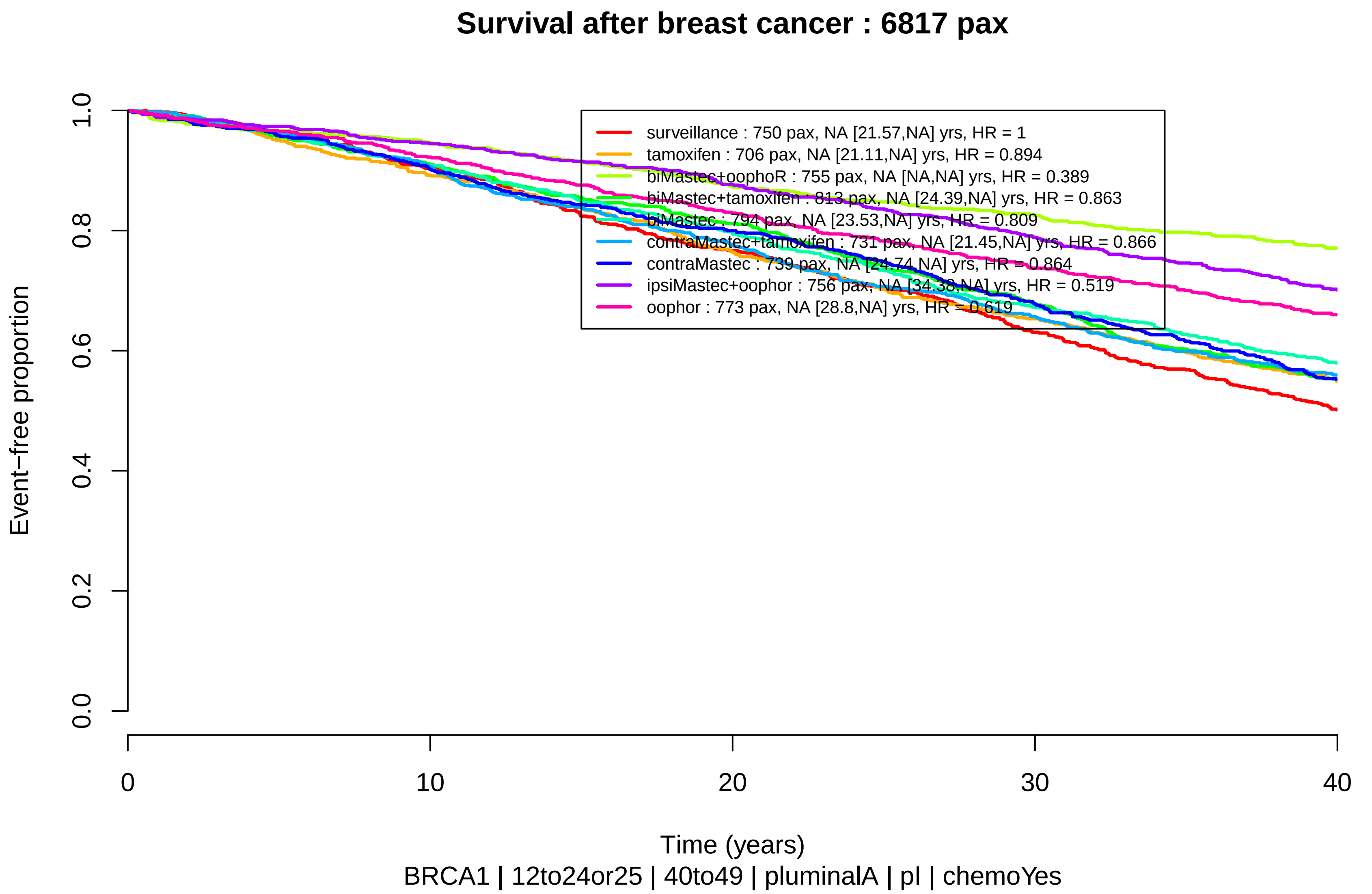

Survival after breast cancer : 6817 pax
surveillance : 750 pax, NA [21.57,NA] yrs, HR = 1
tamoxifen : 706 pax, NA [21.11,NA] yrs, HR = 0.894
biMastec+oophoR : 755 pax, NA [NA,NA] yrs, HR = 0.389
biMastec+tamoxifen : 813 pax, NA [24.39,NA] yrs, HR = 0.863
biMastec : 794 pax, NA [23.53,NA] yrs, HR = 0.809
contraMastec+tamoxifen : 731 pax, NA [21.45,NA] yrs, HR = 0.866
contraMastec : 739 pax, NA [24.74,NA] yrs, HR = 0.864
ipsiMastec+oophor : 756 pax, NA [34.38,NA] yrs, HR = 0.519
oophor : 773 pax, NA [28.8,NA] yrs, HR = 0.619
Event−free proportion
0.0
0.2
0.4
0.6
0.8
1.0
0
10
20
30
40
Time (years)
BRCA1 | 12to24or25 | 40to49 | pluminalA | pI | chemoYes

Survival after breast cancer : 7033 pax

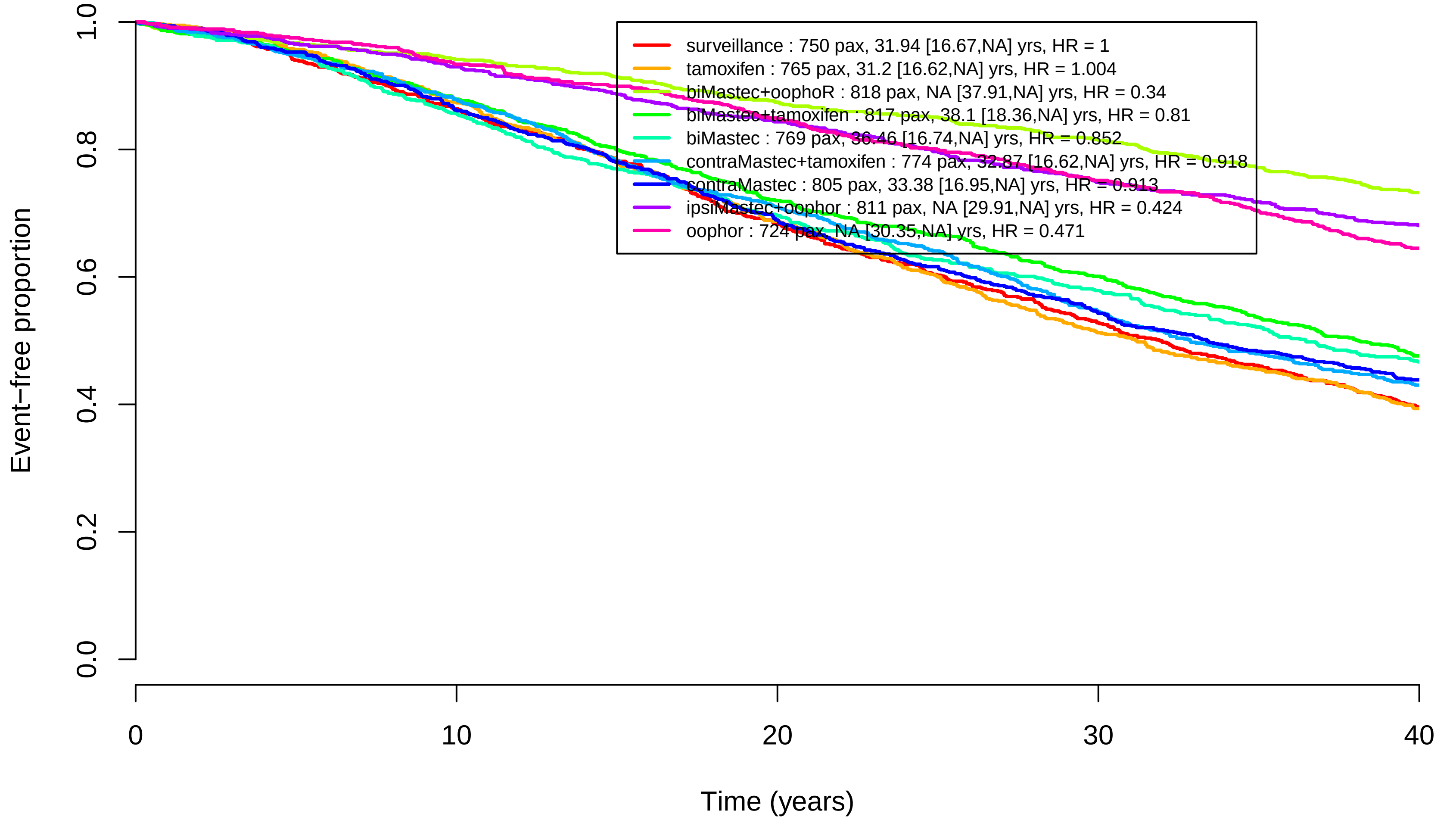

BRCA2 | 12to24or25 | 40to49 | pluminalA | pI | chemoYes

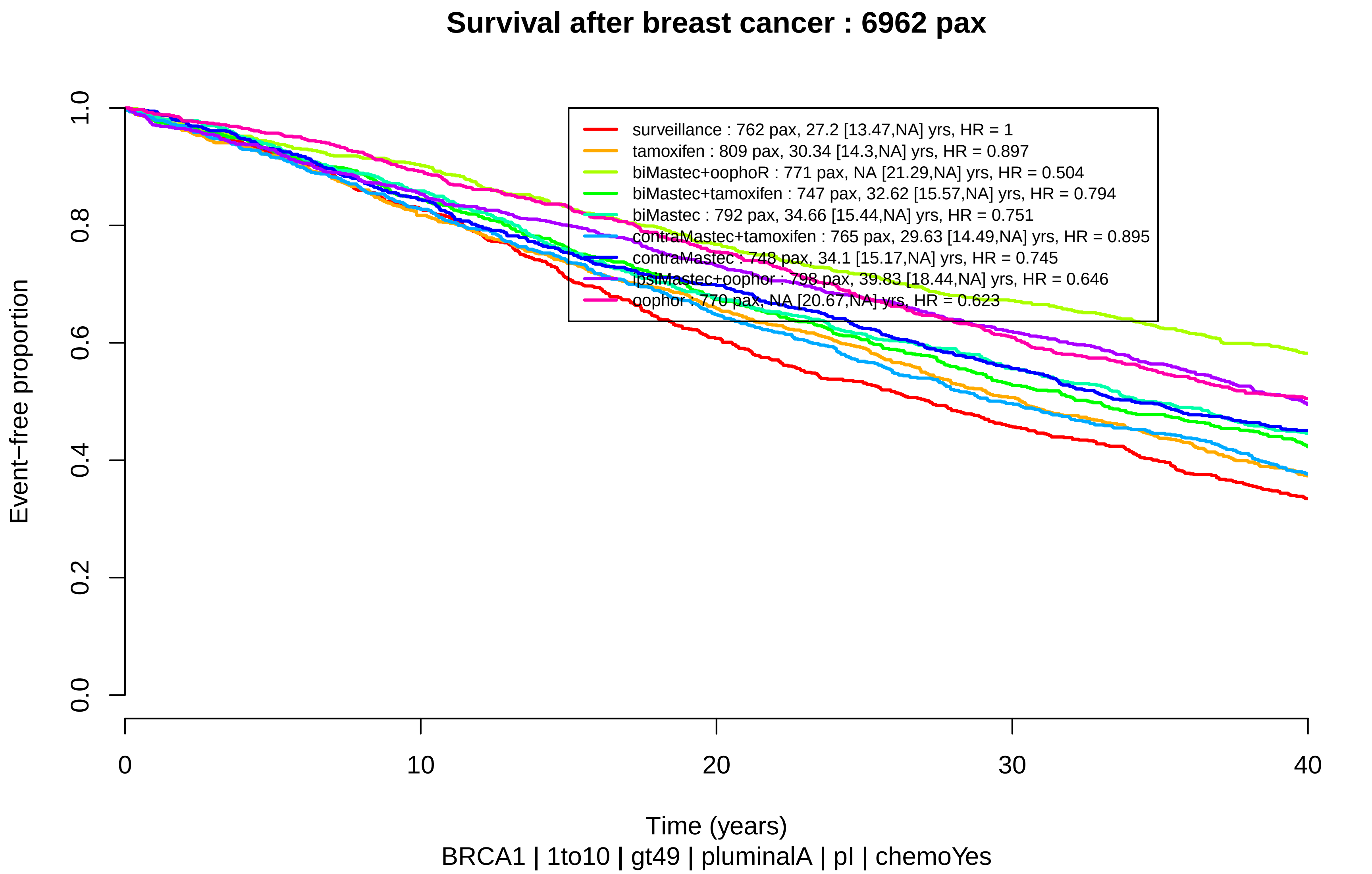
Survival after breast cancer : 6962 pax
surveillance : 762 pax, 27.2 [13.47,NA] yrs, HR = 1
tamoxifen : 809 pax, 30.34 [14.3,NA] yrs, HR = 0.897
biMastec+oophoR : 771 pax, NA [21.29,NA] yrs, HR = 0.504
biMastec+tamoxifen : 747 pax, 32.62 [15.57,NA] yrs, HR = 0.794
biMastec : 792 pax, 34.66 [15.44,NA] yrs, HR = 0.751
contraMastec+tamoxifen : 765 pax, 29.63 [14.49,NA] yrs, HR = 0.895
contraMastec : 748 pax, 34.1 [15.17,NA] yrs, HR = 0.745
ipsiMastec+oophor : 798 pax, 39.83 [18.44,NA] yrs, HR = 0.646
oophor : 770 pax, NA [20.67,NA] yrs, HR = 0.623
Event−free proportion
0.0
0.2
0.4
0.6
0.8
1.0
0
10
20
30
40
Time (years)
BRCA1 | 1to10 | gt49 | pluminalA | pI | chemoYes

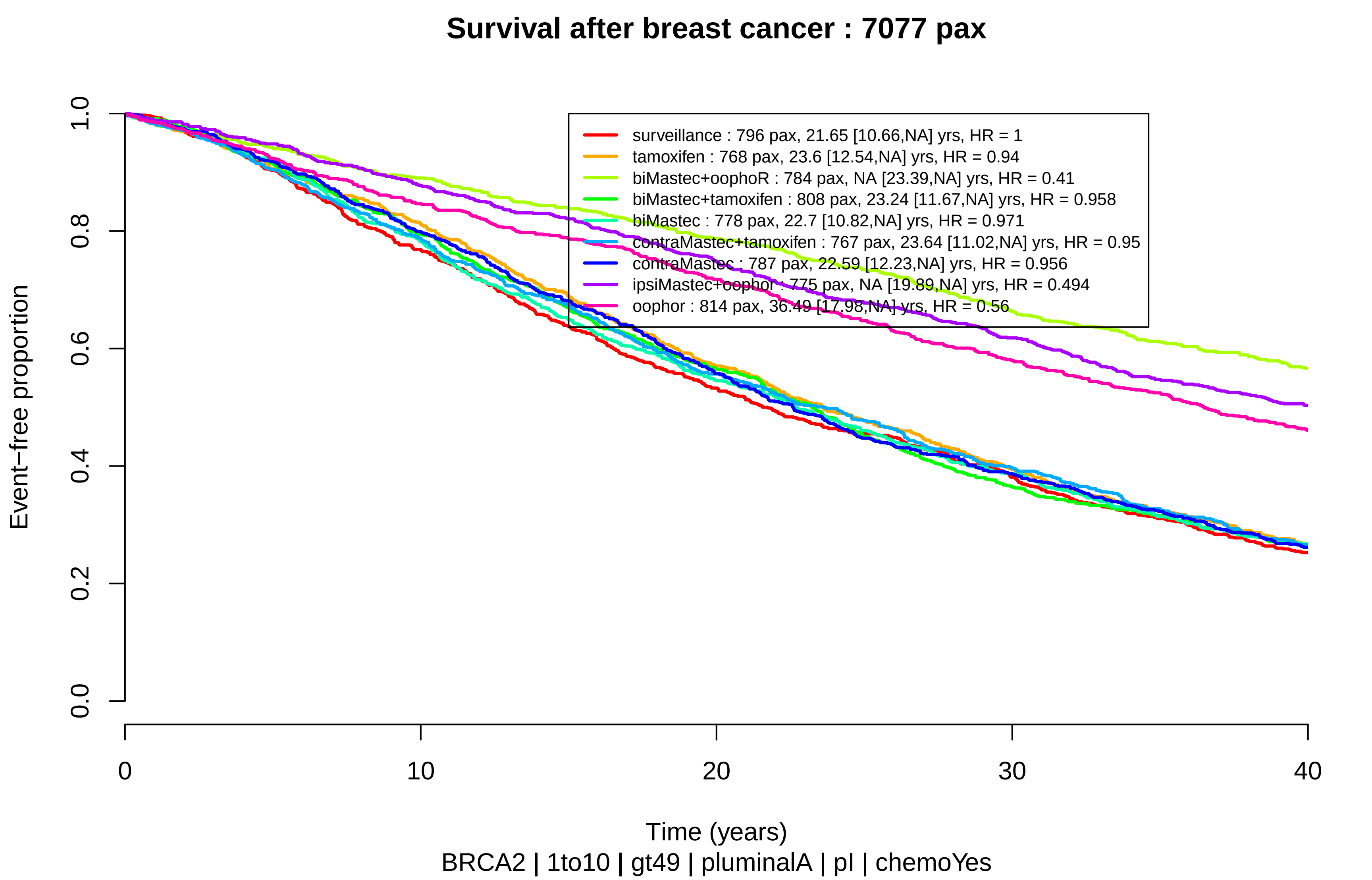

Survival after breast cancer : 7077 pax
surveillance : 796 pax, 21.65 [10.66,NA] yrs, HR = 1
tamoxifen : 768 pax, 23.6 [12.54,NA] yrs, HR = 0.94
biMastec+oophoR : 784 pax, NA [23.39,NA] yrs, HR = 0.41
biMastec+tamoxifen : 808 pax, 23.24 [11.67,NA] yrs, HR = 0.958
biMastec : 778 pax, 22.7 [10.82,NA] yrs, HR = 0.971
contraMastec+tamoxifen : 767 pax, 23.64 [11.02,NA] yrs, HR = 0.95
contraMastec : 787 pax, 22.59 [12.23,NA] yrs, HR = 0.956
ipsiMastec+oophor : 775 pax, NA [19.89,NA] yrs, HR = 0.494
oophor : 814 pax, 36.49 [17.98,NA] yrs, HR = 0.56
Event−free proportion
0.0
0.2
0.4
0.6
0.8
1.0
0
10
20
30
40
Time (years)
BRCA2 | 1to10 | gt49 | pluminalA | pI | chemoYes

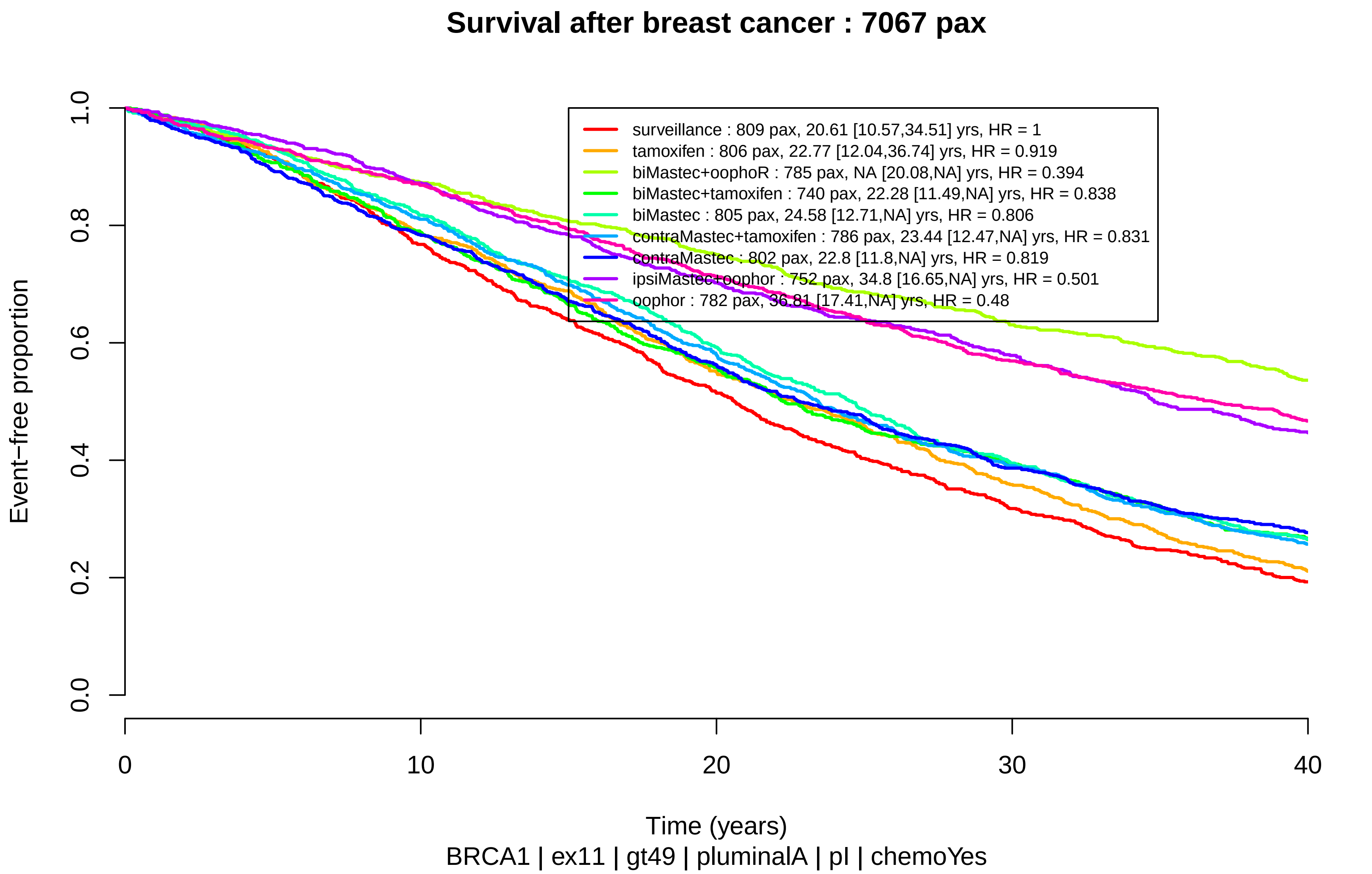
Survival after breast cancer : 7067 pax
Event−free proportion
0.0
0.2
0.4
0.6
0.8
1.0
0
10
20
30
40
Time (years)
BRCA1 | ex11 | gt49 | pluminalA | pI | chemoYes
surveillance : 809 pax, 20.61 [10.57,34.51] yrs, HR = 1
tamoxifen : 806 pax, 22.77 [12.04,36.74] yrs, HR = 0.919
biMastec+oophoR : 785 pax, NA [20.08,NA] yrs, HR = 0.394
biMastec+tamoxifen : 740 pax, 22.28 [11.49,NA] yrs, HR = 0.838
biMastec : 805 pax, 24.58 [12.71,NA] yrs, HR = 0.806
contraMastec+tamoxifen : 786 pax, 23.44 [12.47,NA] yrs, HR = 0.831
contraMastec : 802 pax, 22.8 [11.8,NA] yrs, HR = 0.819
ipsiMastec+oophor : 752 pax, 34.8 [16.65,NA] yrs, HR = 0.501
oophor : 782 pax, 36.81 [17.41,NA] yrs, HR = 0.48

Survival after breast cancer : 7033 pax

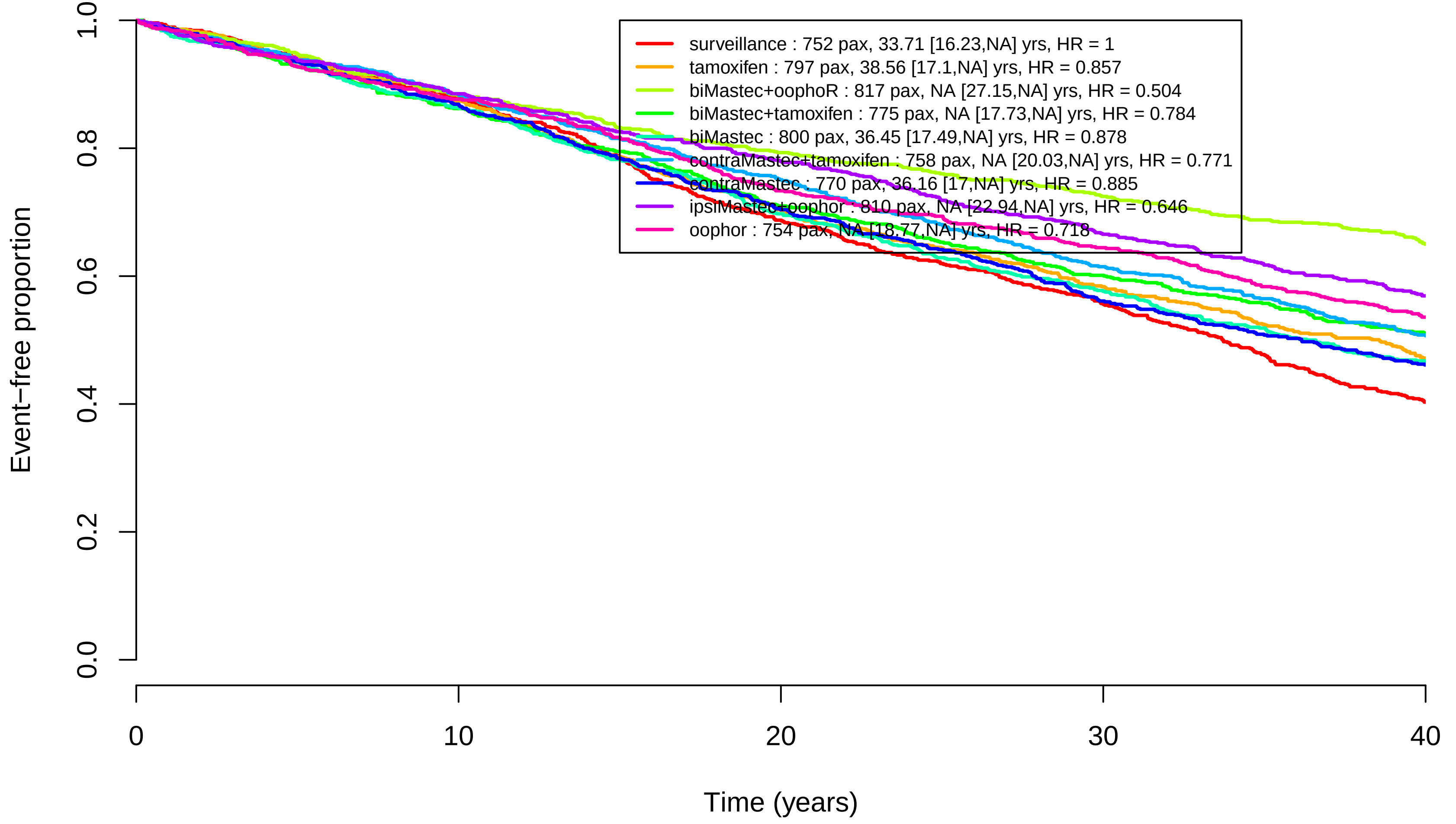

BRCA2 | ex11 | gt49 | pluminalA | pI | chemoYes

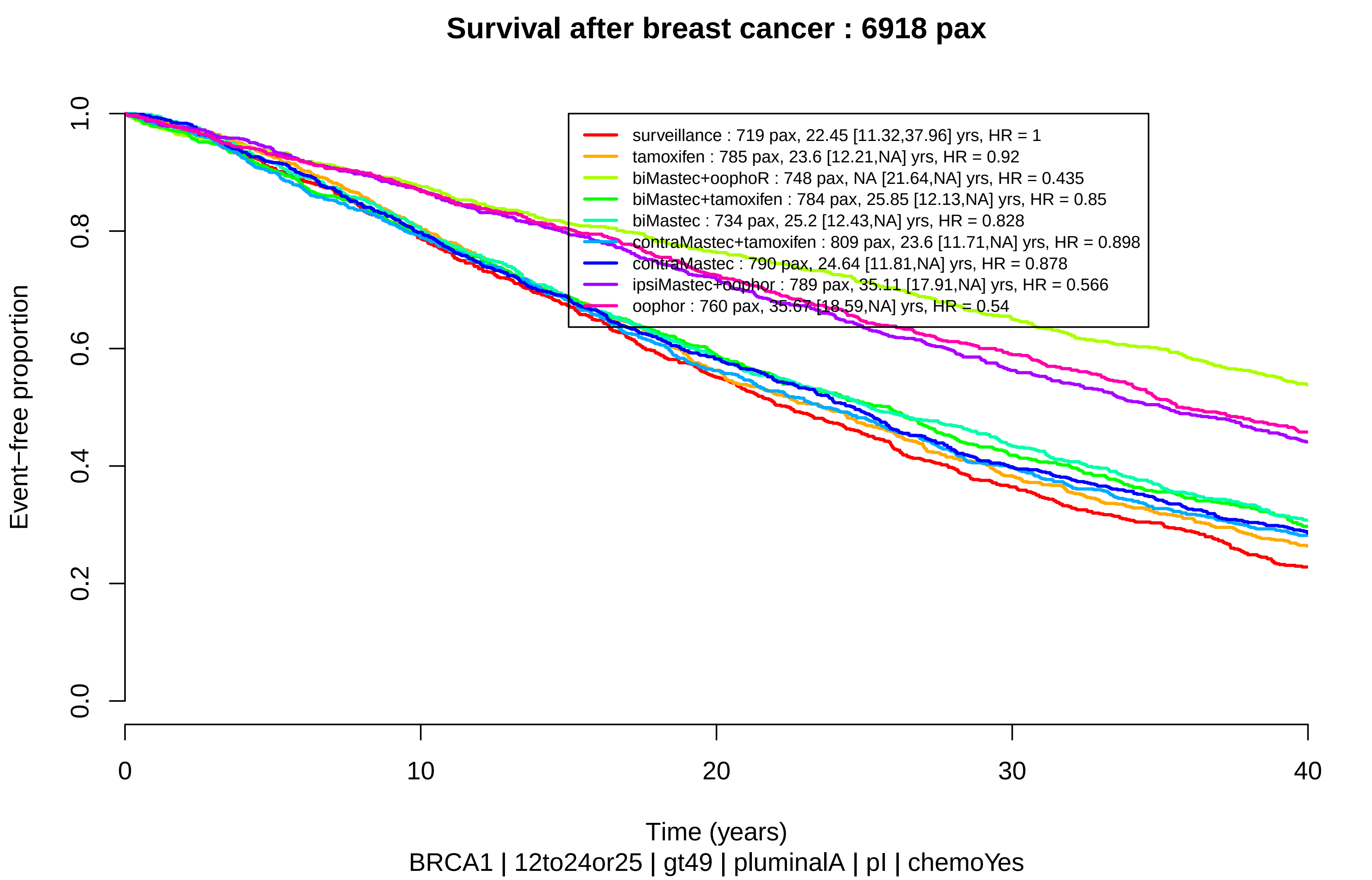
Survival after breast cancer : 6918 pax
surveillance : 719 pax, 22.45 [11.32,37.96] yrs, HR = 1
tamoxifen : 785 pax, 23.6 [12.21,NA] yrs, HR = 0.92
biMastec+oophoR : 748 pax, NA [21.64,NA] yrs, HR = 0.435
biMastec+tamoxifen : 784 pax, 25.85 [12.13,NA] yrs, HR = 0.85
biMastec : 734 pax, 25.2 [12.43,NA] yrs, HR = 0.828
contraMastec+tamoxifen : 809 pax, 23.6 [11.71,NA] yrs, HR = 0.898
contraMastec : 790 pax, 24.64 [11.81,NA] yrs, HR = 0.878
ipsiMastec+oophor : 789 pax, 35.11 [17.91,NA] yrs, HR = 0.566
oophor : 760 pax, 35.67 [18.59,NA] yrs, HR = 0.54
Event−free proportion
0.0
0.2
0.4
0.6
0.8
1.0
0
10
20
30
40
Time (years)
BRCA1 | 12to24or25 | gt49 | pluminalA | pI | chemoYes

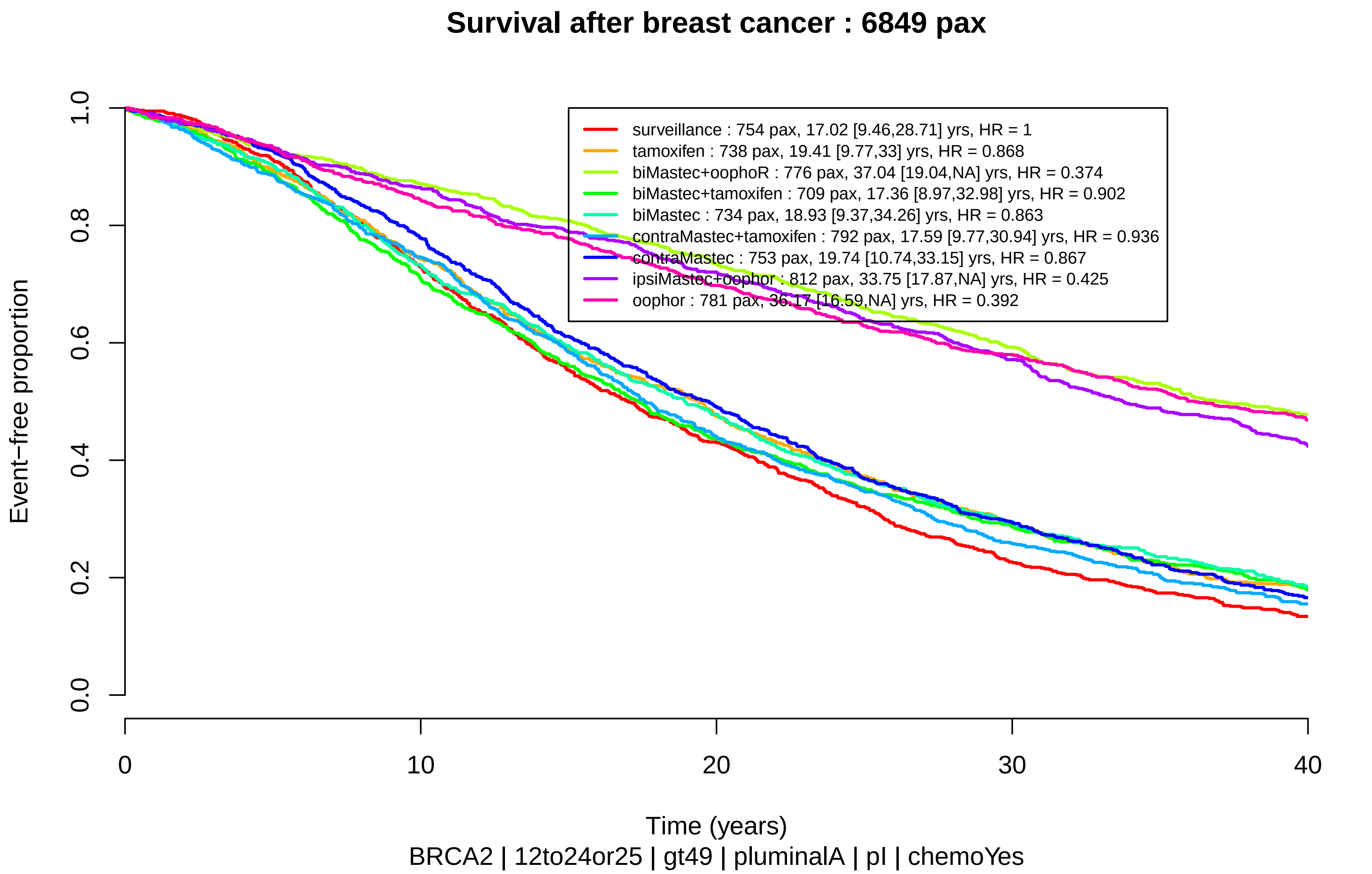

Survival after breast cancer : 6849 pax
surveillance : 754 pax, 17.02 [9.46,28.71] yrs, HR = 1
tamoxifen : 738 pax, 19.41 [9.77,33] yrs, HR = 0.868
biMastec+oophoR : 776 pax, 37.04 [19.04,NA] yrs, HR = 0.374
biMastec+tamoxifen : 709 pax, 17.36 [8.97,32.98] yrs, HR = 0.902
biMastec : 734 pax, 18.93 [9.37,34.26] yrs, HR = 0.863
contraMastec+tamoxifen : 792 pax, 17.59 [9.77,30.94] yrs, HR = 0.936
contraMastec : 753 pax, 19.74 [10.74,33.15] yrs, HR = 0.867
ipsiMastec+oophor : 812 pax, 33.75 [17.87,NA] yrs, HR = 0.425
oophor : 781 pax, 36.17 [16.59,NA] yrs, HR = 0.392
Event−free proportion
0.0
0.2
0.4
0.6
0.8
1.0
0
10
20
30
40
Time (years)
BRCA2 | 12to24or25 | gt49 | pluminalA | pI | chemoYes

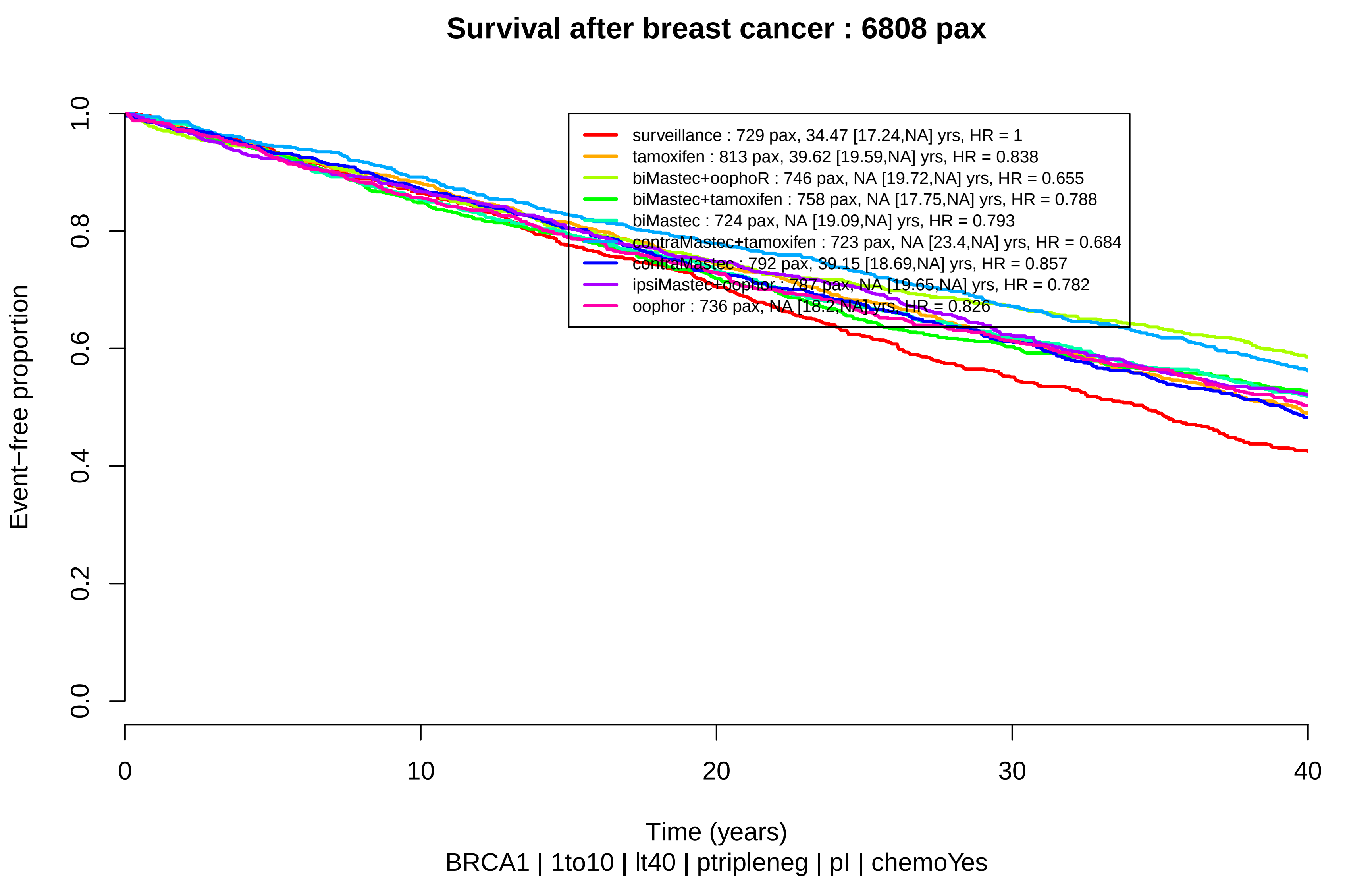
Survival after breast cancer : 6808 pax
surveillance : 729 pax, 34.47 [17.24,NA] yrs, HR = 1
tamoxifen : 813 pax, 39.62 [19.59,NA] yrs, HR = 0.838
biMastec+oophoR : 746 pax, NA [19.72,NA] yrs, HR = 0.655
biMastec+tamoxifen : 758 pax, NA [17.75,NA] yrs, HR = 0.788
biMastec : 724 pax, NA [19.09,NA] yrs, HR = 0.793
contraMastec+tamoxifen : 723 pax, NA [23.4,NA] yrs, HR = 0.684
contraMastec : 792 pax, 39.15 [18.69,NA] yrs, HR = 0.857
ipsiMastec+oophor : 787 pax, NA [19.65,NA] yrs, HR = 0.782
oophor : 736 pax, NA [18.2,NA] yrs, HR = 0.826
Event−free proportion
0.0
0.2
0.4
0.6
0.8
1.0
0
10
20
30
40
Time (years)
BRCA1 | 1to10 | lt40 | ptripleneg | pl | chemoYes

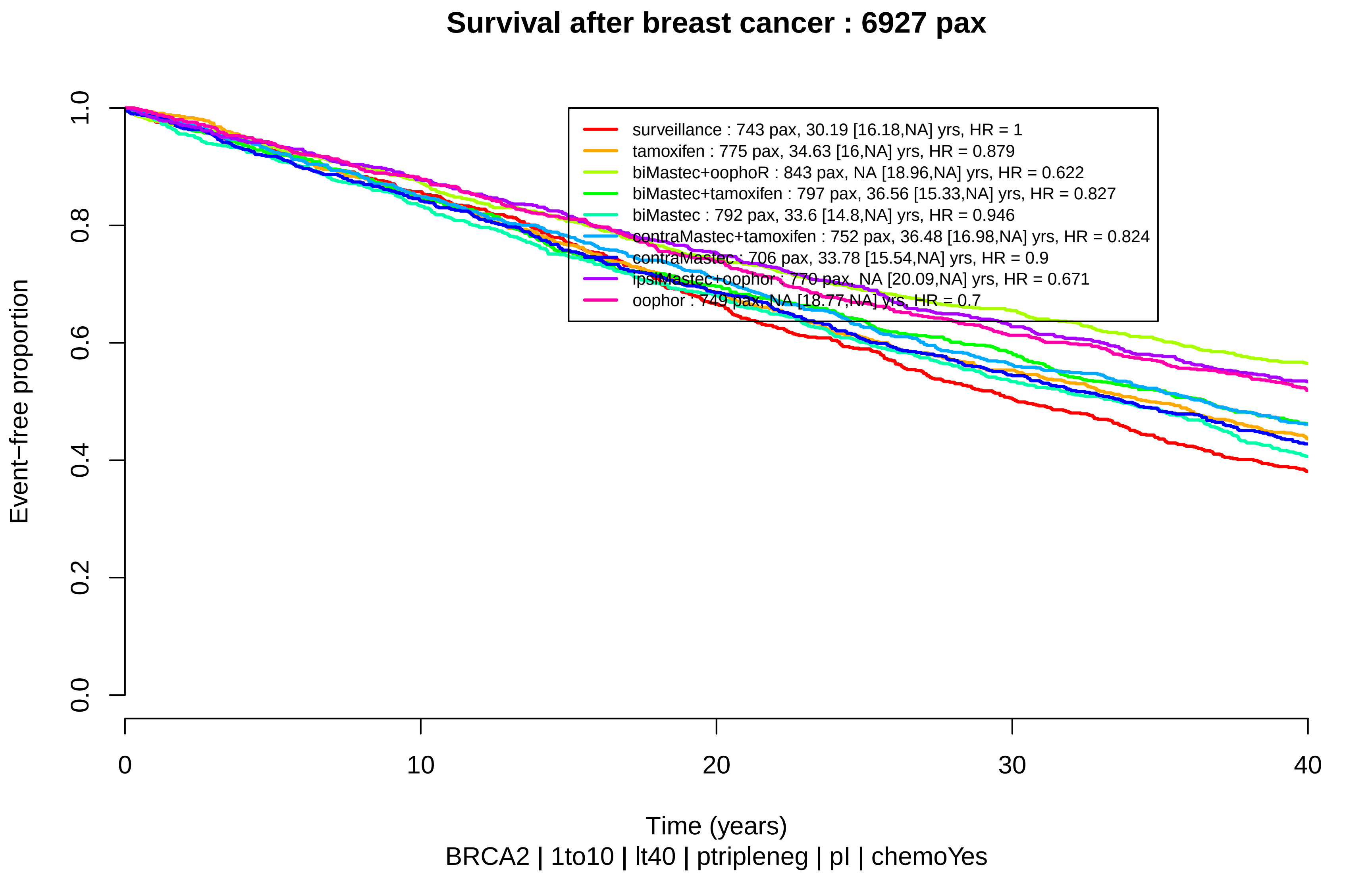
Survival after breast cancer : 6927 pax
Event−free proportion
0.0
0.2
0.4
0.6
0.8
1.0
0
10
20
30
40
Time (years)
BRCA2 | 1to10 | lt40 | ptripleneg | pl | chemoYes
surveillance : 743 pax, 30.19 [16.18,NA] yrs, HR = 1
tamoxifen : 775 pax, 34.63 [16,NA] yrs, HR = 0.879
biMastec+oophoR : 843 pax, NA [18.96,NA] yrs, HR = 0.622
biMastec+tamoxifen : 797 pax, 36.56 [15.33,NA] yrs, HR = 0.827
biMastec : 792 pax, 33.6 [14.8,NA] yrs, HR = 0.946
contraMastec+tamoxifen : 752 pax, 36.48 [16.98,NA] yrs, HR = 0.824
contraMastec : 706 pax, 33.78 [15.54,NA] yrs, HR = 0.9
ipsiMastec+oophor : 770 pax, NA [20.09,NA] yrs, HR = 0.671
oophor : 749 pax, NA [18.77,NA] yrs, HR = 0.7

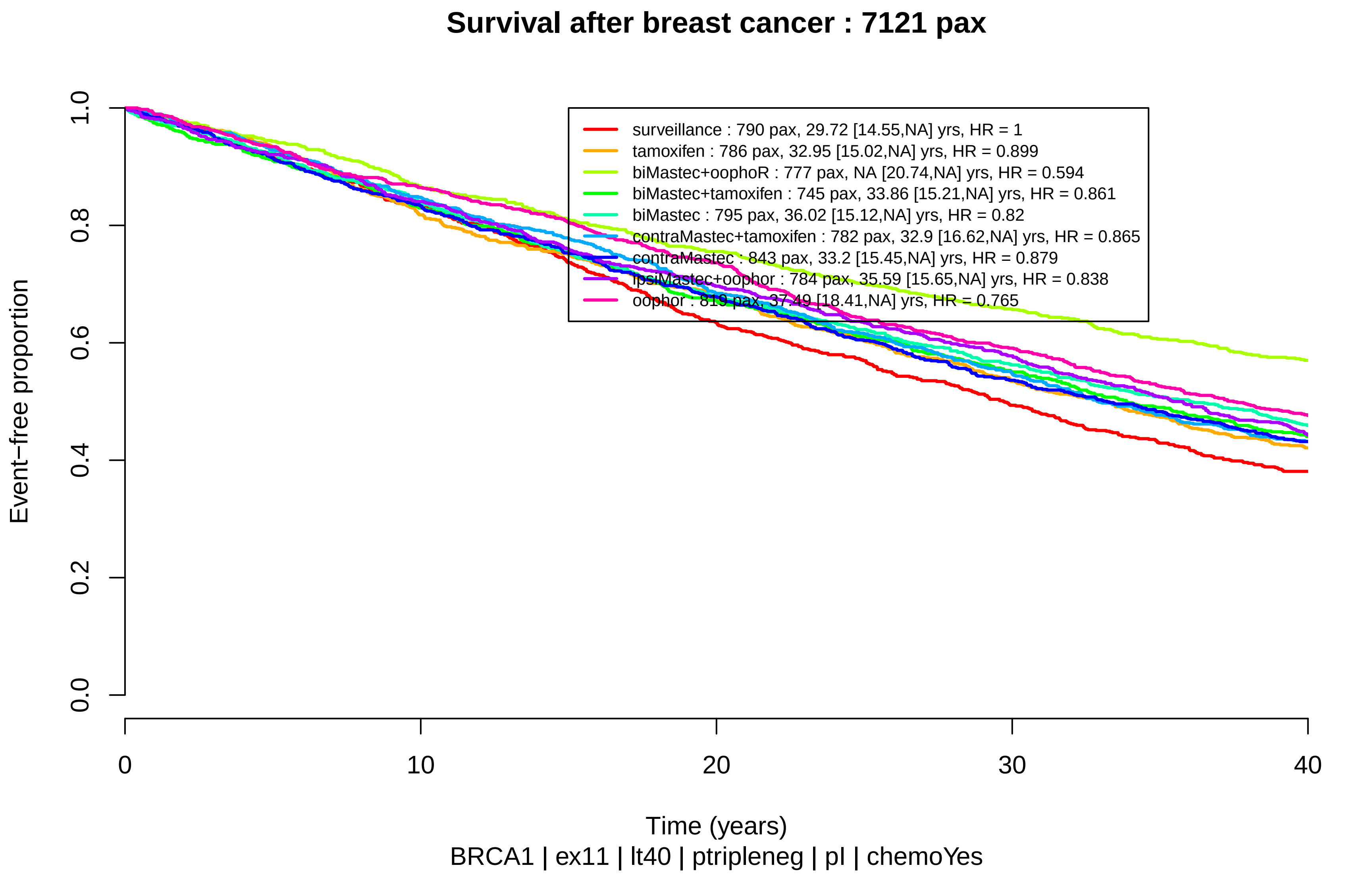

Survival after breast cancer : 7121 pax
Event−free proportion
0.0
0.2
0.4
0.6
0.8
1.0
0
10
20
30
40
Time (years)
BRCA1 | ex11 | lt40 | ptripleneg | pl | chemoYes
surveillance : 790 pax, 29.72 [14.55,NA] yrs, HR = 1
tamoxifen : 786 pax, 32.95 [15.02,NA] yrs, HR = 0.899
biMastec+oophoR : 777 pax, NA [20.74,NA] yrs, HR = 0.594
biMastec+tamoxifen : 745 pax, 33.86 [15.21,NA] yrs, HR = 0.861
biMastec : 795 pax, 36.02 [15.12,NA] yrs, HR = 0.82
contraMastec+tamoxifen : 782 pax, 32.9 [16.62,NA] yrs, HR = 0.865
contraMastec : 843 pax, 33.2 [15.45,NA] yrs, HR = 0.879
ipsiMastec+oophor : 784 pax, 35.59 [15.65,NA] yrs, HR = 0.838
oophor : 819 pax, 37.49 [18.41,NA] yrs, HR = 0.765

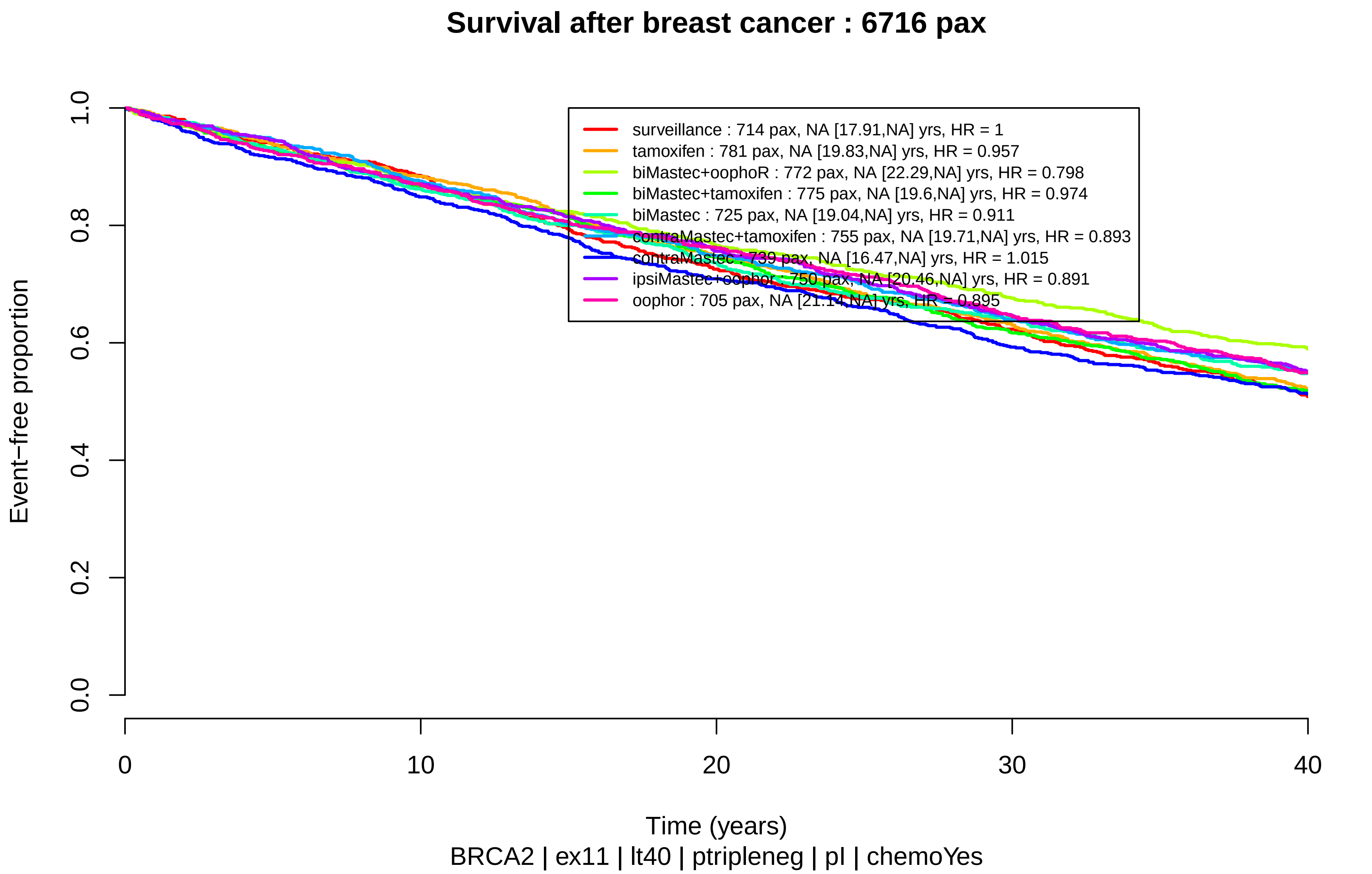

Survival after breast cancer : 6716 pax
surveillance : 714 pax, NA [17.91,NA] yrs, HR = 1
tamoxifen : 781 pax, NA [19.83,NA] yrs, HR = 0.957
biMastec+oophoR : 772 pax, NA [22.29,NA] yrs, HR = 0.798
biMastec+tamoxifen : 775 pax, NA [19.6,NA] yrs, HR = 0.974
biMastec : 725 pax, NA [19.04,NA] yrs, HR = 0.911
contraMastec+tamoxifen : 755 pax, NA [19.71,NA] yrs, HR = 0.893
contraMastec : 739 pax, NA [16.47,NA] yrs, HR = 1.015
ipsiMastec+oophor : 750 pax, NA [20.46,NA] yrs, HR = 0.891
oophor : 705 pax, NA [21.14,NA] yrs, HR = 0.895
Event−free proportion
0.0
0.2
0.4
0.6
0.8
1.0
0
10
20
30
40
Time (years)
BRCA2 | ex11 | lt40 | ptripleneg | pl | chemoYes

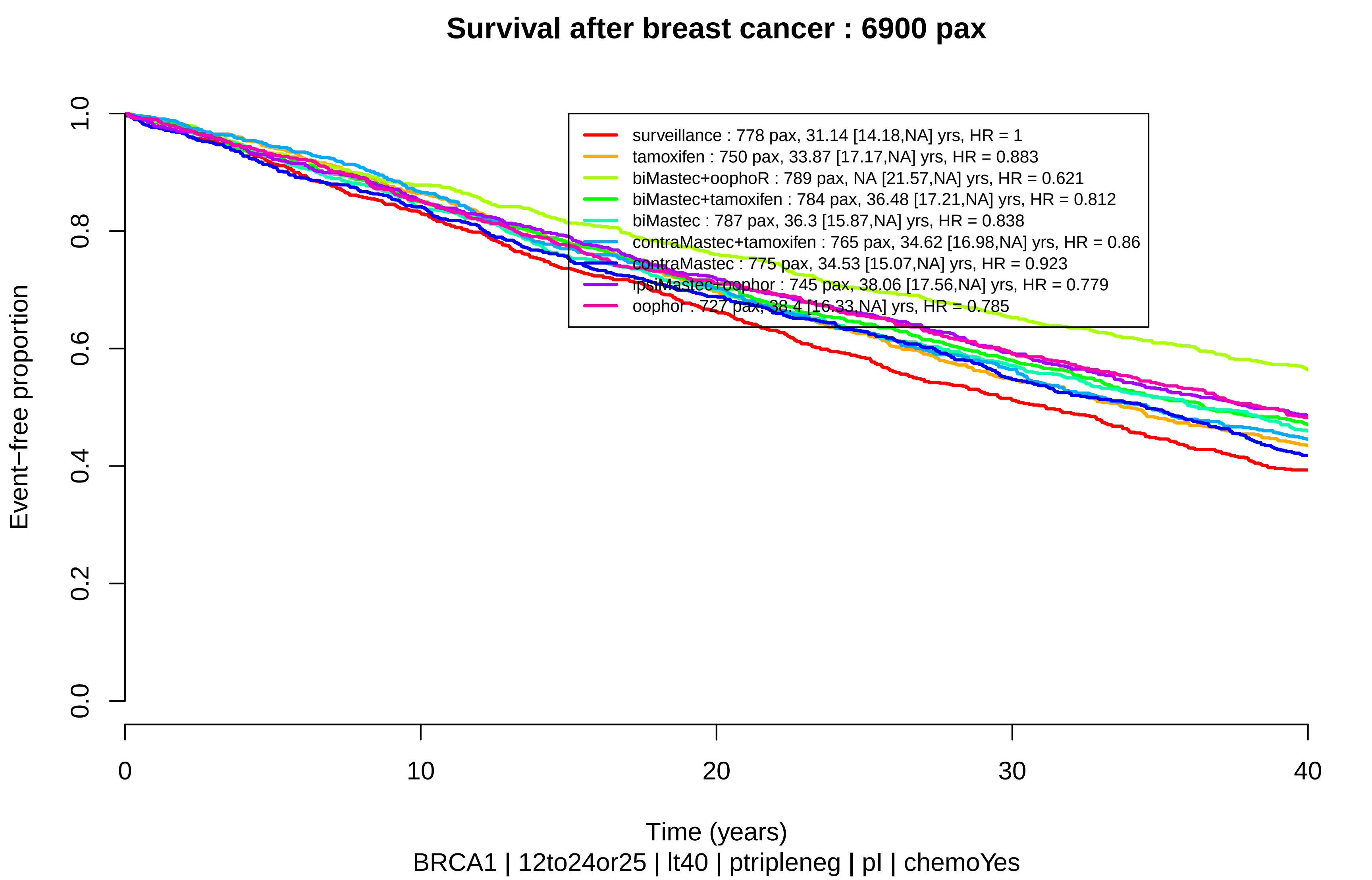

Survival after breast cancer : 6900 pax
Event−free proportion
0.0
0.2
0.4
0.6
0.8
1.0
0
10
20
30
40
Time (years)
BRCA1 | 12to24or25 | lt40 | ptripleneg | pI | chemoYes
surveillance : 778 pax, 31.14 [14.18,NA] yrs, HR = 1
tamoxifen : 750 pax, 33.87 [17.17,NA] yrs, HR = 0.883
biMastec+oophoR : 789 pax, NA [21.57,NA] yrs, HR = 0.621
biMastec+tamoxifen : 784 pax, 36.48 [17.21,NA] yrs, HR = 0.812
biMastec : 787 pax, 36.3 [15.87,NA] yrs, HR = 0.838
contraMastec+tamoxifen : 765 pax, 34.62 [16.98,NA] yrs, HR = 0.86
contraMastec : 775 pax, 34.53 [15.07,NA] yrs, HR = 0.923
ipsiMastec+oophor : 745 pax, 38.06 [17.56,NA] yrs, HR = 0.779
oophor : 727 pax, 38.4 [16.33,NA] yrs, HR = 0.785

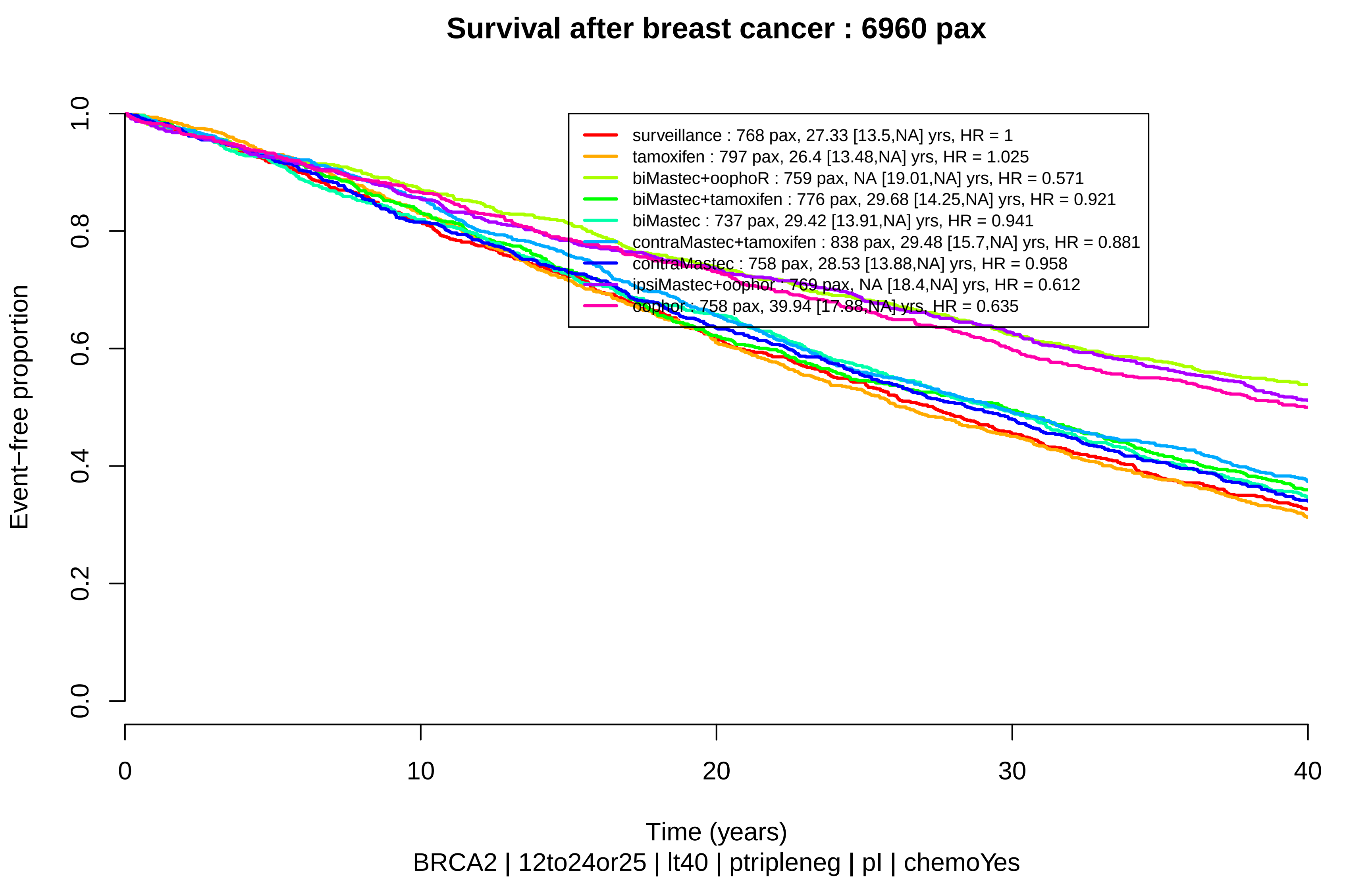

Survival after breast cancer : 6960 pax
surveillance : 768 pax, 27.33 [13.5,NA] yrs, HR = 1
tamoxifen : 797 pax, 26.4 [13.48,NA] yrs, HR = 1.025
biMastec+oophoR : 759 pax, NA [19.01,NA] yrs, HR = 0.571
biMastec+tamoxifen : 776 pax, 29.68 [14.25,NA] yrs, HR = 0.921
biMastec : 737 pax, 29.42 [13.91,NA] yrs, HR = 0.941
contraMastec+tamoxifen : 838 pax, 29.48 [15.7,NA] yrs, HR = 0.881
contraMastec : 758 pax, 28.53 [13.88,NA] yrs, HR = 0.958
ipsiMastec+oophor : 769 pax, NA [18.4,NA] yrs, HR = 0.612
oophor : 758 pax, 39.94 [17.88,NA] yrs, HR = 0.635
Event−free proportion
0.0
0.2
0.4
0.6
0.8
1.0
0
10
20
30
40
Time (years)
BRCA2 | 12to24or25 | lt40 | ptripleneg | pl | chemoYes

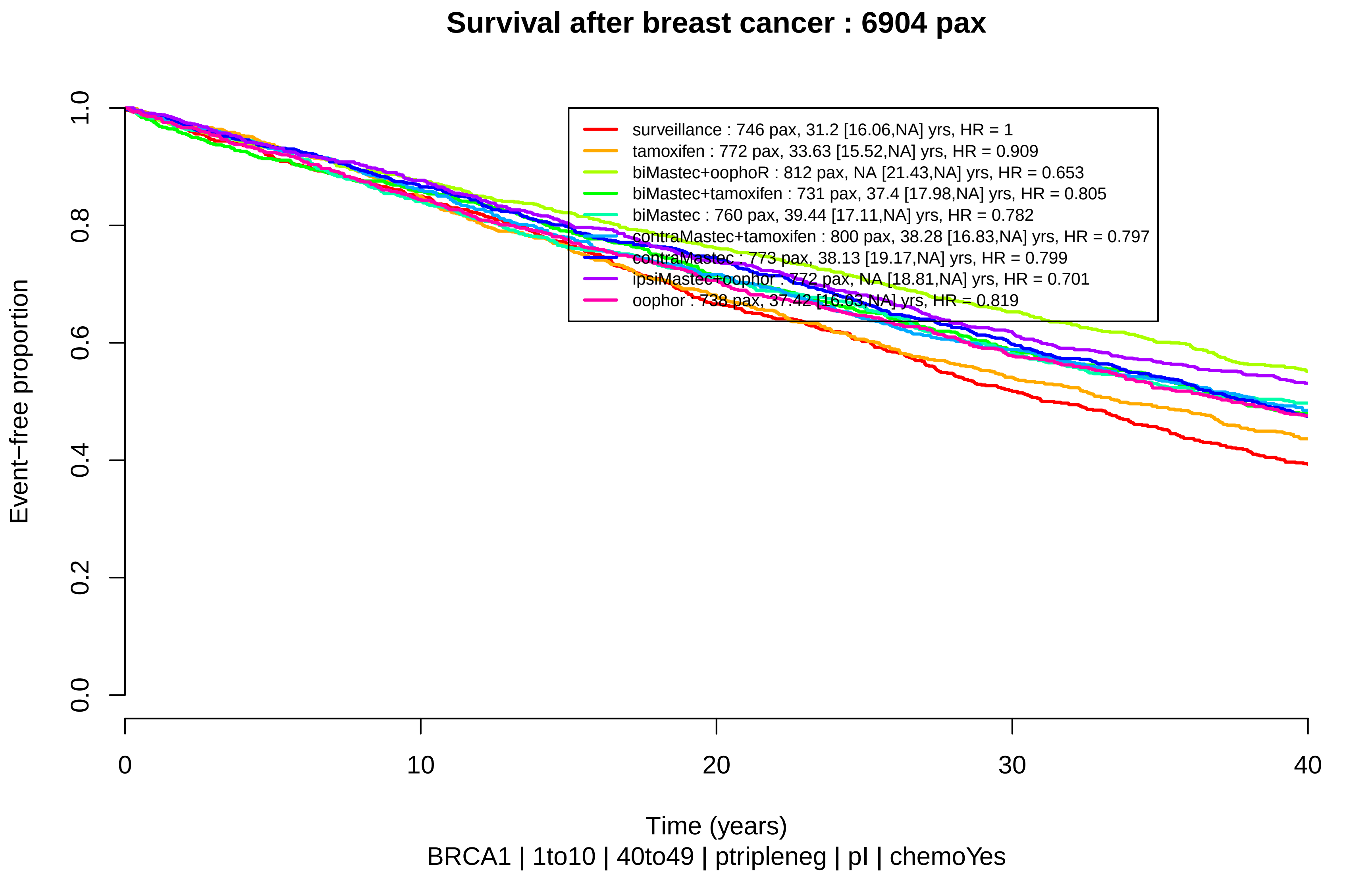
Survival after breast cancer : 6904 pax
surveillance : 746 pax, 31.2 [16.06,NA] yrs, HR = 1
tamoxifen : 772 pax, 33.63 [15.52,NA] yrs, HR = 0.909
biMastec+oophoR : 812 pax, NA [21.43,NA] yrs, HR = 0.653
biMastec+tamoxifen : 731 pax, 37.4 [17.98,NA] yrs, HR = 0.805
biMastec : 760 pax, 39.44 [17.11,NA] yrs, HR = 0.782
contraMastec+tamoxifen : 800 pax, 38.28 [16.83,NA] yrs, HR = 0.797
contraMastec : 773 pax, 38.13 [19.17,NA] yrs, HR = 0.799
ipsiMastec+oophor : 772 pax, NA [18.81,NA] yrs, HR = 0.701
oophor : 738 pax, 37.42 [16.63,NA] yrs, HR = 0.819
Event−free proportion
0.0
0.2
0.4
0.6
0.8
1.0
0
10
20
30
40
Time (years)
BRCA1 | 1to10 | 40to49 | ptripleneg | pI | chemoYes

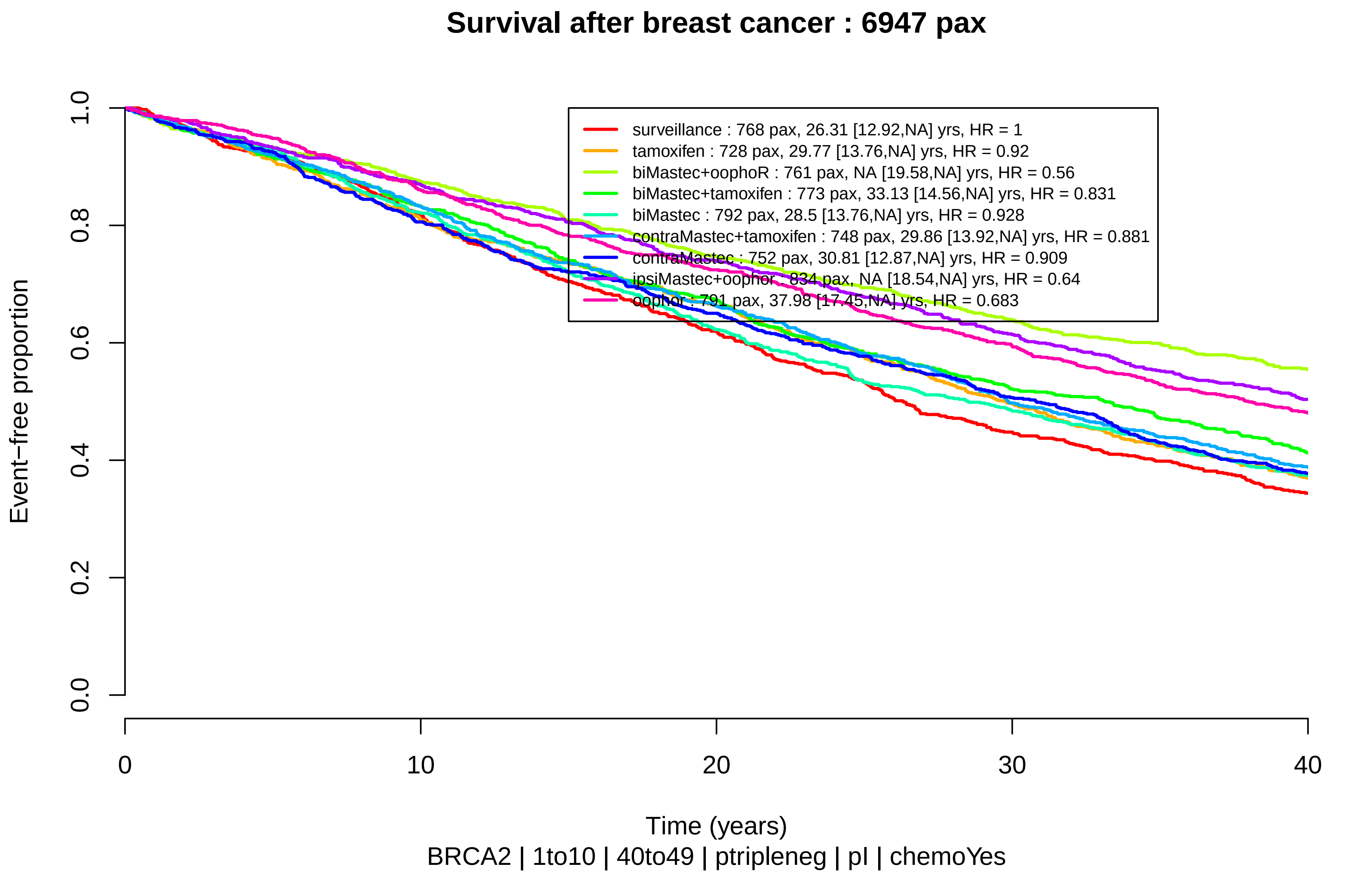

Survival after breast cancer : 6947 pax
surveillance : 768 pax, 26.31 [12.92,NA] yrs, HR = 1
tamoxifen : 728 pax, 29.77 [13.76,NA] yrs, HR = 0.92
biMastec+oophoR : 761 pax, NA [19.58,NA] yrs, HR = 0.56
biMastec+tamoxifen : 773 pax, 33.13 [14.56,NA] yrs, HR = 0.831
biMastec : 792 pax, 28.5 [13.76,NA] yrs, HR = 0.928
contraMastec+tamoxifen : 748 pax, 29.86 [13.92,NA] yrs, HR = 0.881
contraMastec : 752 pax, 30.81 [12.87,NA] yrs, HR = 0.909
ipsiMastec+oophor : 834 pax, NA [18.54,NA] yrs, HR = 0.64
oophor : 791 pax, 37.98 [17.45,NA] yrs, HR = 0.683
Event−free proportion
0.0
0.2
0.4
0.6
0.8
1.0
0
10
20
30
40
Time (years)
BRCA2 | 1to10 | 40to49 | ptripleneg | pl | chemoYes

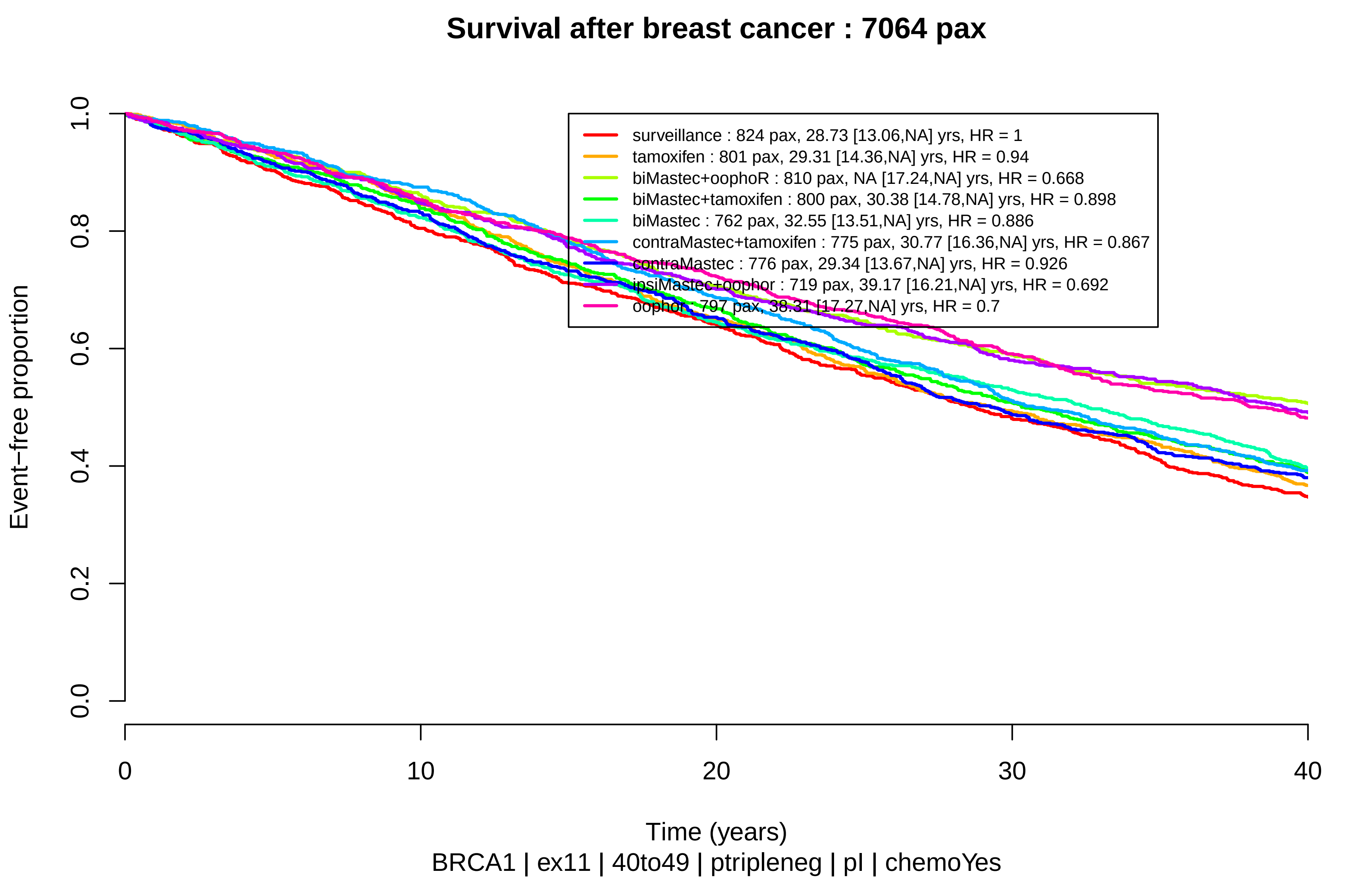

Survival after breast cancer : 7064 pax
surveillance : 824 pax, 28.73 [13.06,NA] yrs, HR = 1
tamoxifen : 801 pax, 29.31 [14.36,NA] yrs, HR = 0.94
biMastec+oophoR : 810 pax, NA [17.24,NA] yrs, HR = 0.668
biMastec+tamoxifen : 800 pax, 30.38 [14.78,NA] yrs, HR = 0.898
biMastec : 762 pax, 32.55 [13.51,NA] yrs, HR = 0.886
contraMastec+tamoxifen : 775 pax, 30.77 [16.36,NA] yrs, HR = 0.867
contraMastec : 776 pax, 29.34 [13.67,NA] yrs, HR = 0.926
ipsiMastec+oophor : 719 pax, 39.17 [16.21,NA] yrs, HR = 0.692
oophor : 797 pax, 38.31 [17.27,NA] yrs, HR = 0.7
Event−free proportion
0.0
0.2
0.4
0.6
0.8
1.0
0
10
20
30
40
Time (years)
BRCA1 | ex11 | 40to49 | ptripleneg | pl | chemoYes

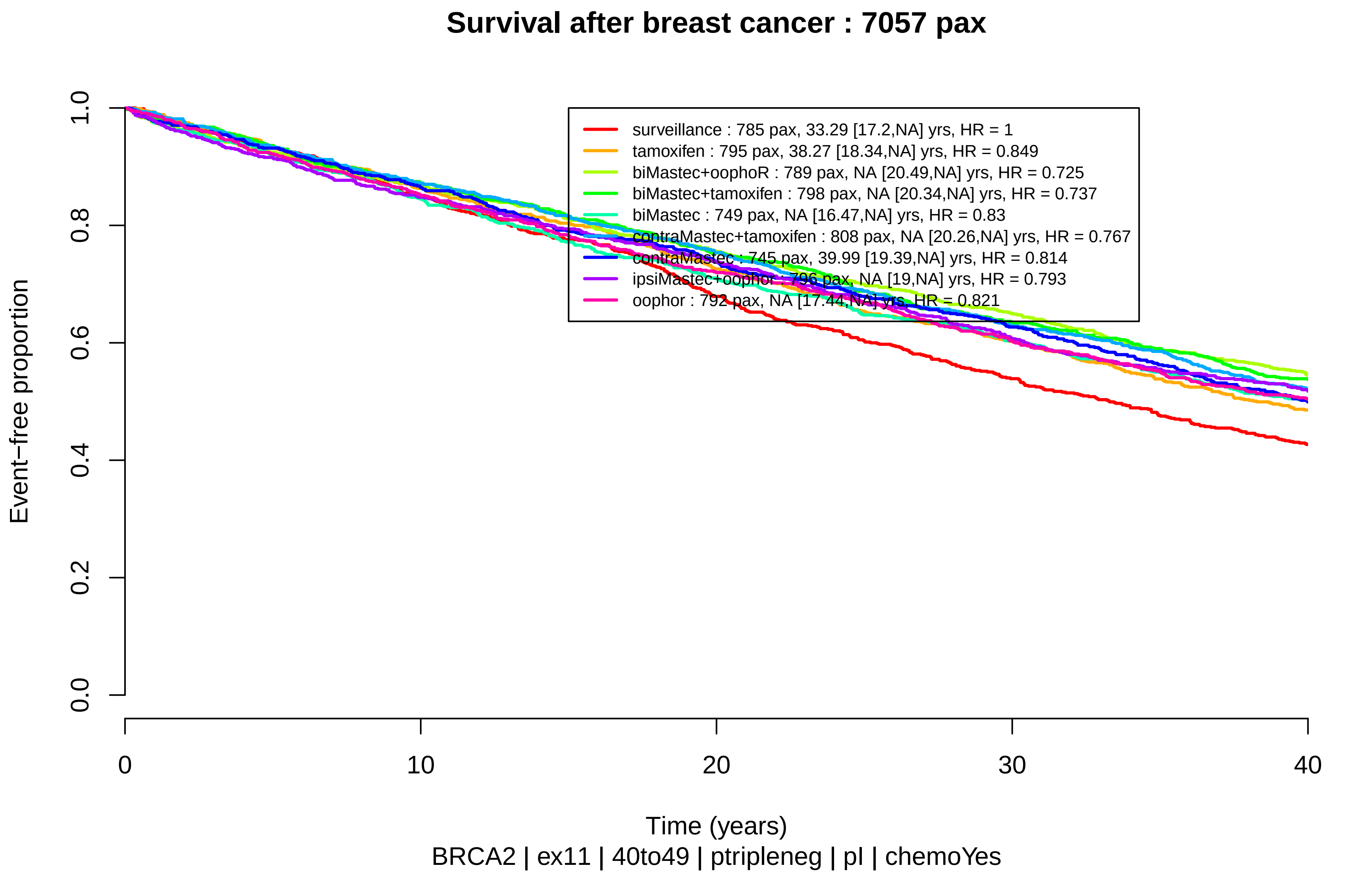

Survival after breast cancer : 7057 pax
surveillance : 785 pax, 33.29 [17.2,NA] yrs, HR = 1
tamoxifen : 795 pax, 38.27 [18.34,NA] yrs, HR = 0.849
biMastec+oophoR : 789 pax, NA [20.49,NA] yrs, HR = 0.725
biMastec+tamoxifen : 798 pax, NA [20.34,NA] yrs, HR = 0.737
biMastec : 749 pax, NA [16.47,NA] yrs, HR = 0.83
contraMastec+tamoxifen : 808 pax, NA [20.26,NA] yrs, HR = 0.767
contraMastec : 745 pax, 39.99 [19.39,NA] yrs, HR = 0.814
ipsiMastec+oophor : 796 pax, NA [19,NA] yrs, HR = 0.793
oophor : 792 pax, NA [17.44,NA] yrs, HR = 0.821
Event−free proportion
0.0
0.2
0.4
0.6
0.8
1.0
0
10
20
30
40
Time (years)
BRCA2 | ex11 | 40to49 | ptripleneg | pl | chemoYes

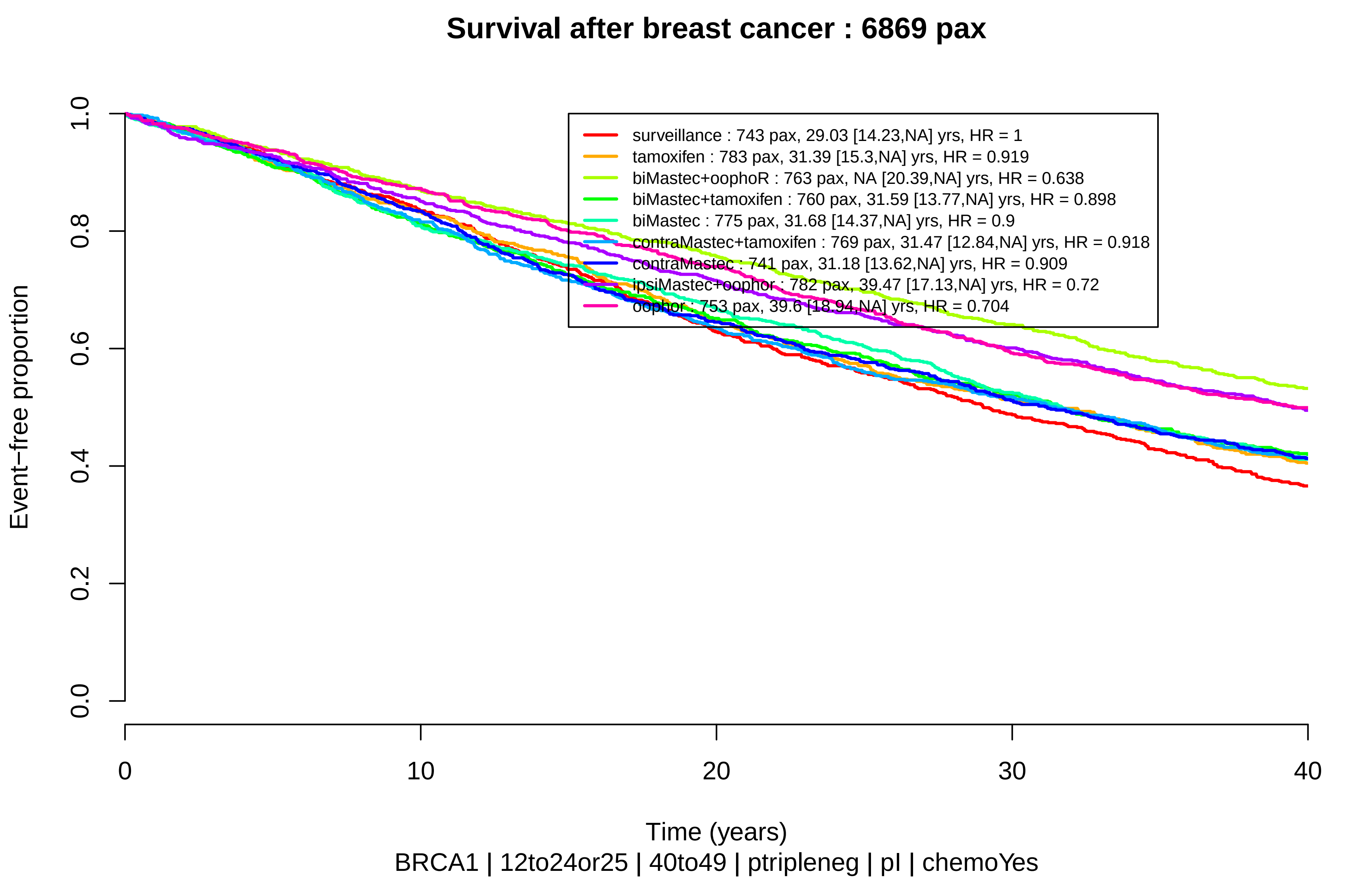

Survival after breast cancer : 6869 pax
surveillance : 743 pax, 29.03 [14.23,NA] yrs, HR = 1
tamoxifen : 783 pax, 31.39 [15.3,NA] yrs, HR = 0.919
biMastec+oophoR : 763 pax, NA [20.39,NA] yrs, HR = 0.638
biMastec+tamoxifen : 760 pax, 31.59 [13.77,NA] yrs, HR = 0.898
biMastec : 775 pax, 31.68 [14.37,NA] yrs, HR = 0.9
contraMastec+tamoxifen : 769 pax, 31.47 [12.84,NA] yrs, HR = 0.918
contraMastec : 741 pax, 31.18 [13.62,NA] yrs, HR = 0.909
ipsiMastec+oophor : 782 pax, 39.47 [17.13,NA] yrs, HR = 0.72
oophor : 753 pax, 39.6 [18.94,NA] yrs, HR = 0.704
Event−free proportion
0.0
0.2
0.4
0.6
0.8
1.0
0
10
20
30
40
Time (years)
BRCA1 | 12to24or25 | 40to49 | ptripleneg | pI | chemoYes

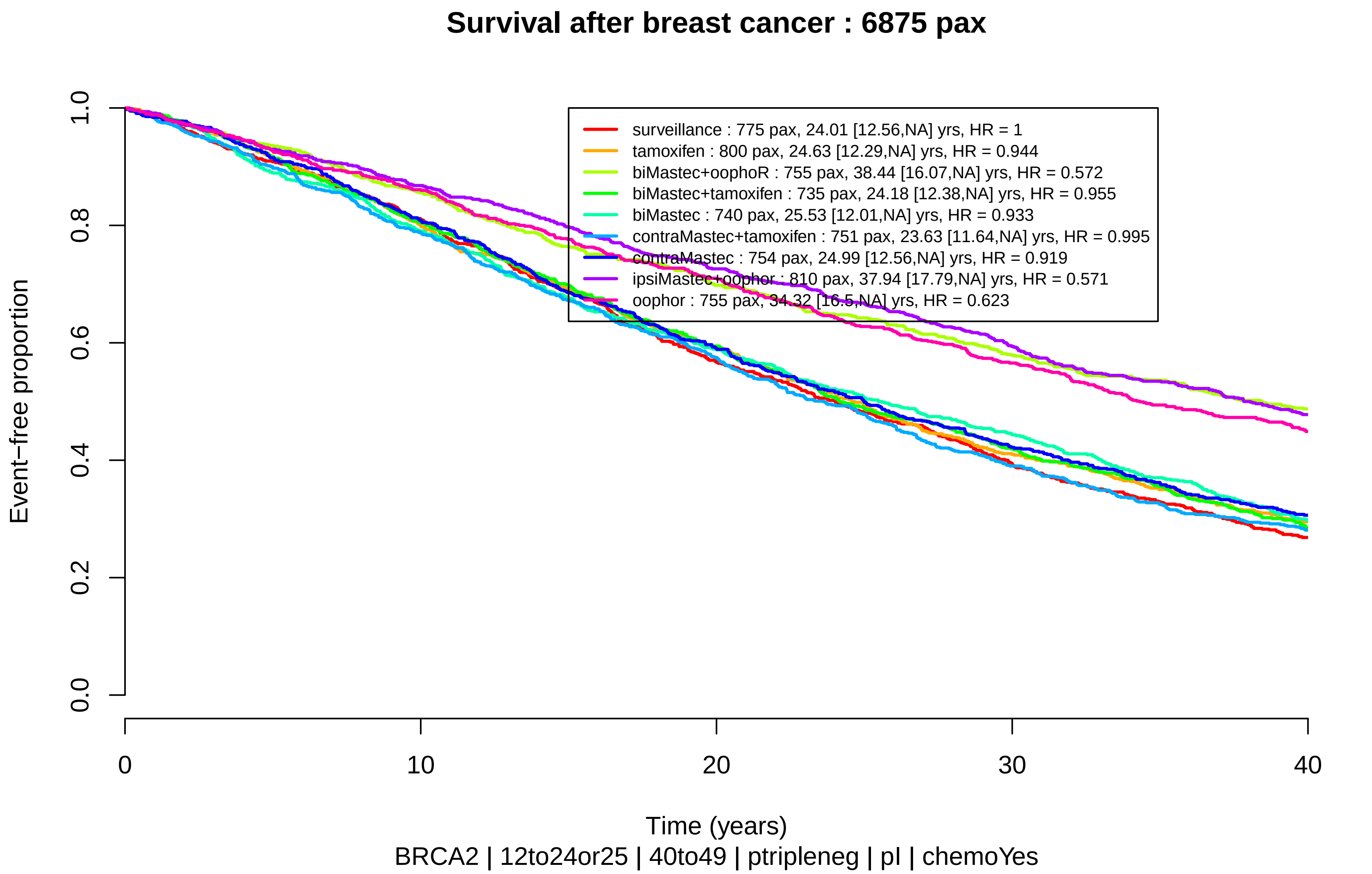
Survival after breast cancer : 6875 pax
surveillance : 775 pax, 24.01 [12.56,NA] yrs, HR = 1
tamoxifen : 800 pax, 24.63 [12.29,NA] yrs, HR = 0.944
biMastec+oophoR : 755 pax, 38.44 [16.07,NA] yrs, HR = 0.572
biMastec+tamoxifen : 735 pax, 24.18 [12.38,NA] yrs, HR = 0.955
biMastec : 740 pax, 25.53 [12.01,NA] yrs, HR = 0.933
contraMastec+tamoxifen : 751 pax, 23.63 [11.64,NA] yrs, HR = 0.995
contraMastec : 754 pax, 24.99 [12.56,NA] yrs, HR = 0.919
ipsiMastec+oophor : 810 pax, 37.94 [17.79,NA] yrs, HR = 0.571
oophor : 755 pax, 34.32 [16.5,NA] yrs, HR = 0.623
Event−free proportion
0.0
0.2
0.4
0.6
0.8
1.0
0
10
20
30
40
Time (years)
BRCA2 | 12to24or25 | 40to49 | ptripleneg | pI | chemoYes

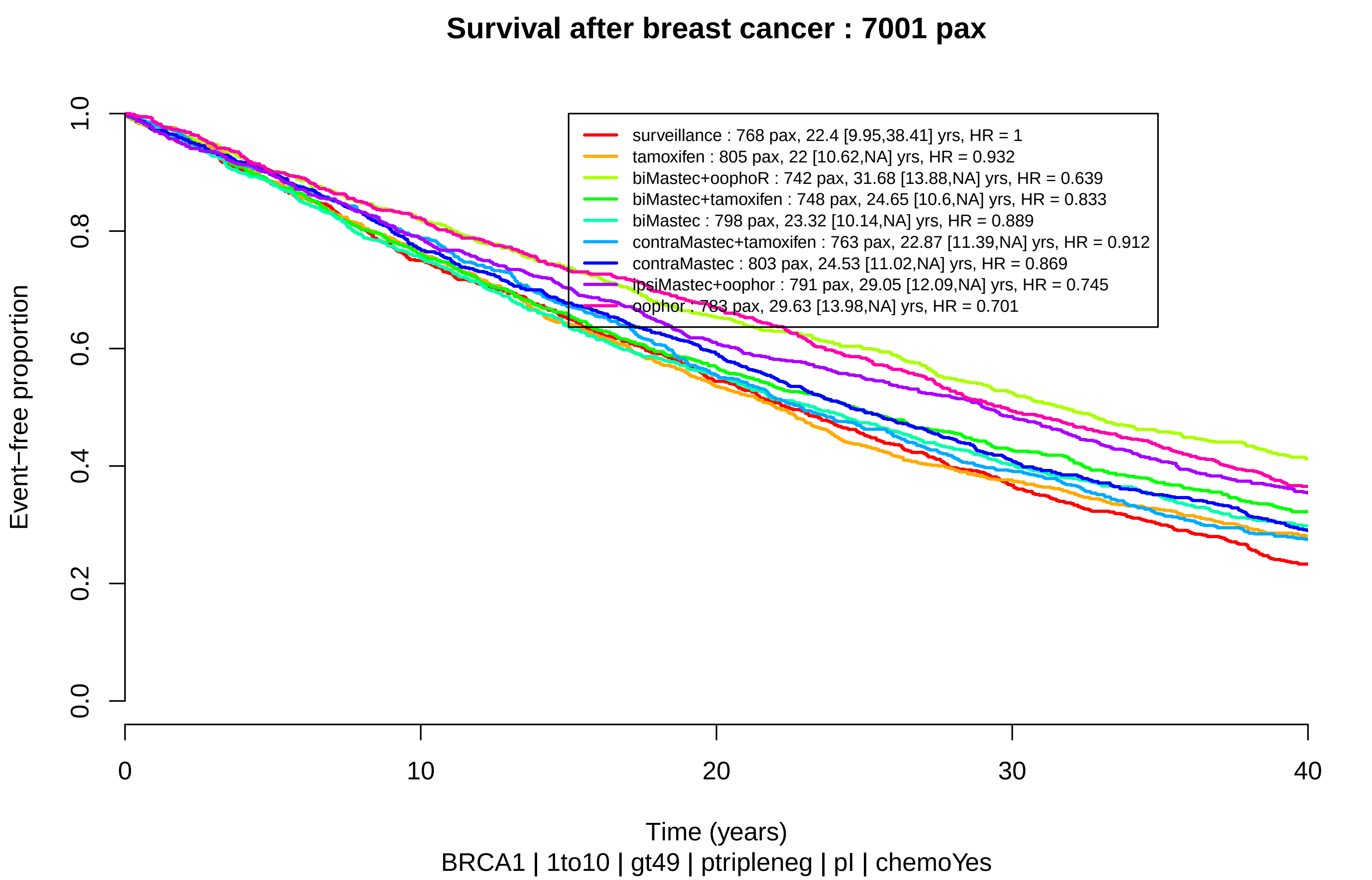

Survival after breast cancer : 7001 pax
Event−free proportion
0.0
0.2
0.4
0.6
0.8
1.0
0
10
20
30
40
Time (years)
BRCA1 | 1to10 | gt49 | ptripleneg | pl | chemoYes
surveillance : 768 pax, 22.4 [9.95,38.41] yrs, HR = 1
tamoxifen : 805 pax, 22 [10.62,NA] yrs, HR = 0.932
biMastec+oophoR : 742 pax, 31.68 [13.88,NA] yrs, HR = 0.639
biMastec+tamoxifen : 748 pax, 24.65 [10.6,NA] yrs, HR = 0.833
biMastec : 798 pax, 23.32 [10.14,NA] yrs, HR = 0.889
contraMastec+tamoxifen : 763 pax, 22.87 [11.39,NA] yrs, HR = 0.912
contraMastec : 803 pax, 24.53 [11.02,NA] yrs, HR = 0.869
ipsiMastec+oophor : 791 pax, 29.05 [12.09,NA] yrs, HR = 0.745
oophor : 783 pax, 29.63 [13.98,NA] yrs, HR = 0.701

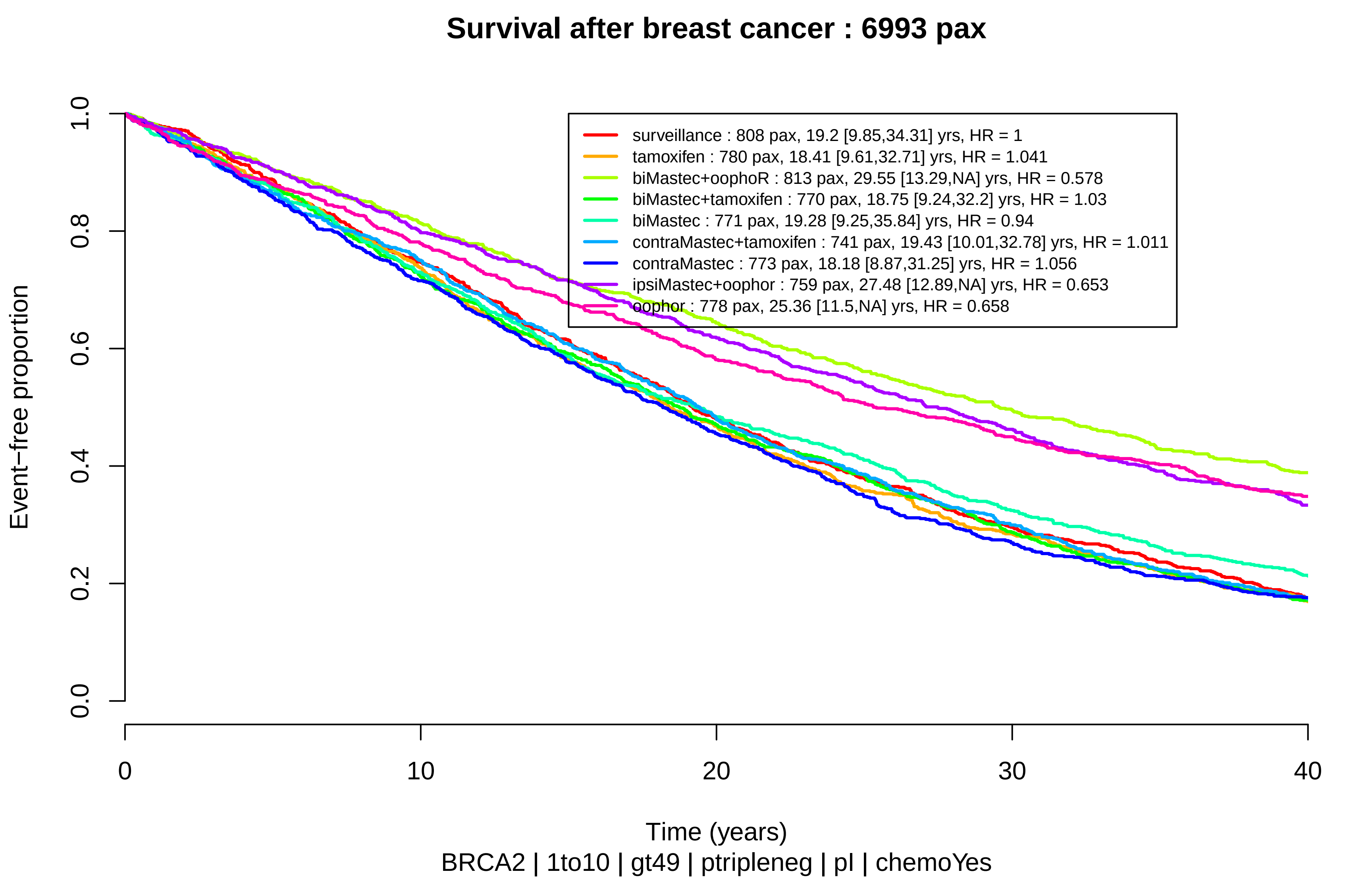

Survival after breast cancer : 6993 pax
surveillance : 808 pax, 19.2 [9.85,34.31] yrs, HR = 1
tamoxifen : 780 pax, 18.41 [9.61,32.71] yrs, HR = 1.041
biMastec+oophoR : 813 pax, 29.55 [13.29,NA] yrs, HR = 0.578
biMastec+tamoxifen : 770 pax, 18.75 [9.24,32.2] yrs, HR = 1.03
biMastec : 771 pax, 19.28 [9.25,35.84] yrs, HR = 0.94
contraMastec+tamoxifen : 741 pax, 19.43 [10.01,32.78] yrs, HR = 1.011
contraMastec : 773 pax, 18.18 [8.87,31.25] yrs, HR = 1.056
ipsiMastec+oophor : 759 pax, 27.48 [12.89,NA] yrs, HR = 0.653
oophor : 778 pax, 25.36 [11.5,NA] yrs, HR = 0.658
Event−free proportion
0.0
0.2
0.4
0.6
0.8
1.0
0
10
20
30
40
Time (years)
BRCA2 | 1to10 | gt49 | ptripleneg | pl | chemoYes

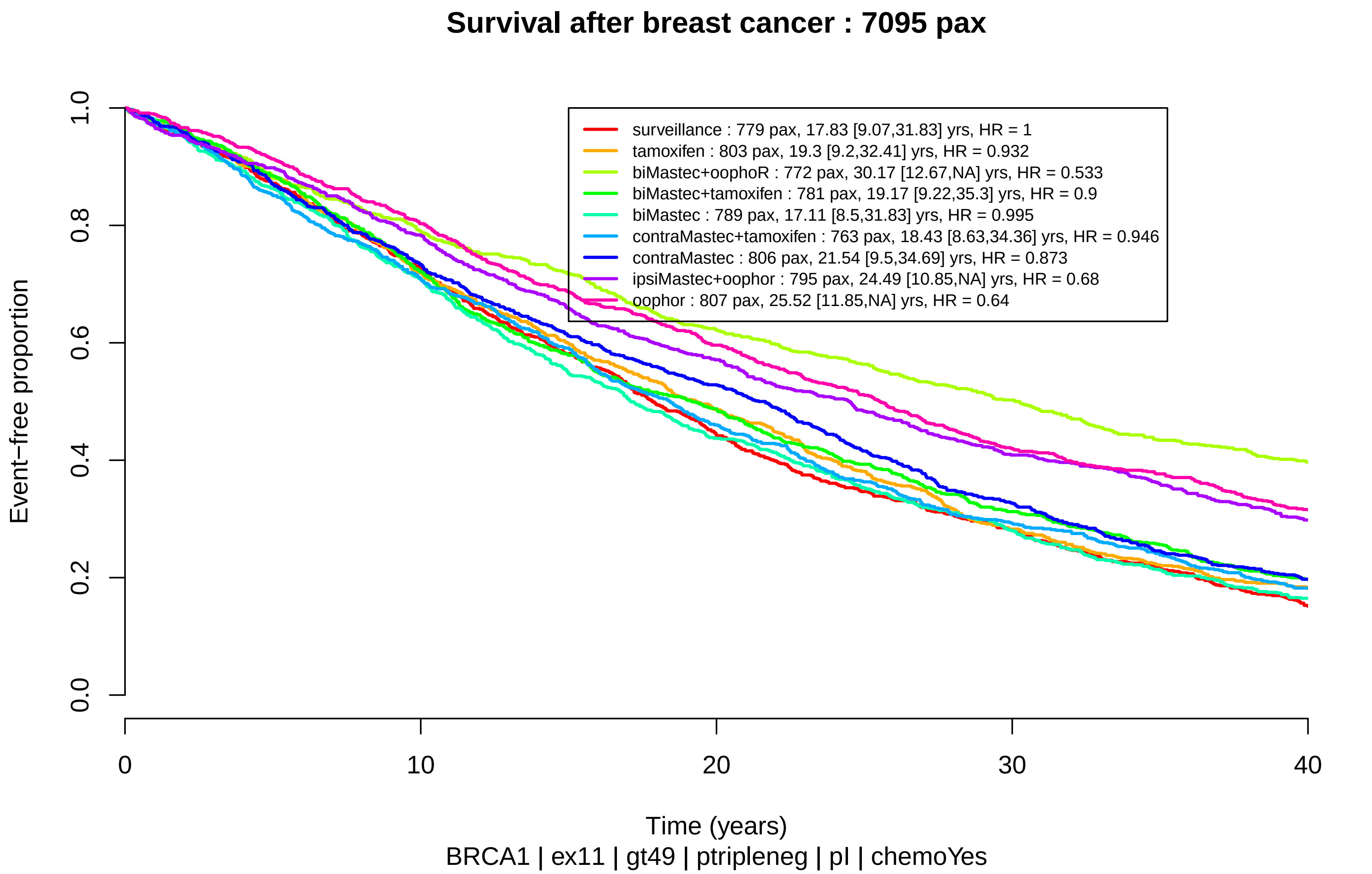

Survival after breast cancer : 7095 pax
surveillance : 779 pax, 17.83 [9.07,31.83] yrs, HR = 1
tamoxifen : 803 pax, 19.3 [9.2,32.41] yrs, HR = 0.932
biMastec+oophoR : 772 pax, 30.17 [12.67,NA] yrs, HR = 0.533
biMastec+tamoxifen : 781 pax, 19.17 [9.22,35.3] yrs, HR = 0.9
biMastec : 789 pax, 17.11 [8.5,31.83] yrs, HR = 0.995
contraMastec+tamoxifen : 763 pax, 18.43 [8.63,34.36] yrs, HR = 0.946
contraMastec : 806 pax, 21.54 [9.5,34.69] yrs, HR = 0.873
ipsiMastec+oophor : 795 pax, 24.49 [10.85,NA] yrs, HR = 0.68
oophor : 807 pax, 25.52 [11.85,NA] yrs, HR = 0.64
Event−free proportion
0.0
0.2
0.4
0.6
0.8
1.0
0
10
20
30
40
Time (years)
BRCA1 | ex11 | gt49 | ptripleneg | pl | chemoYes

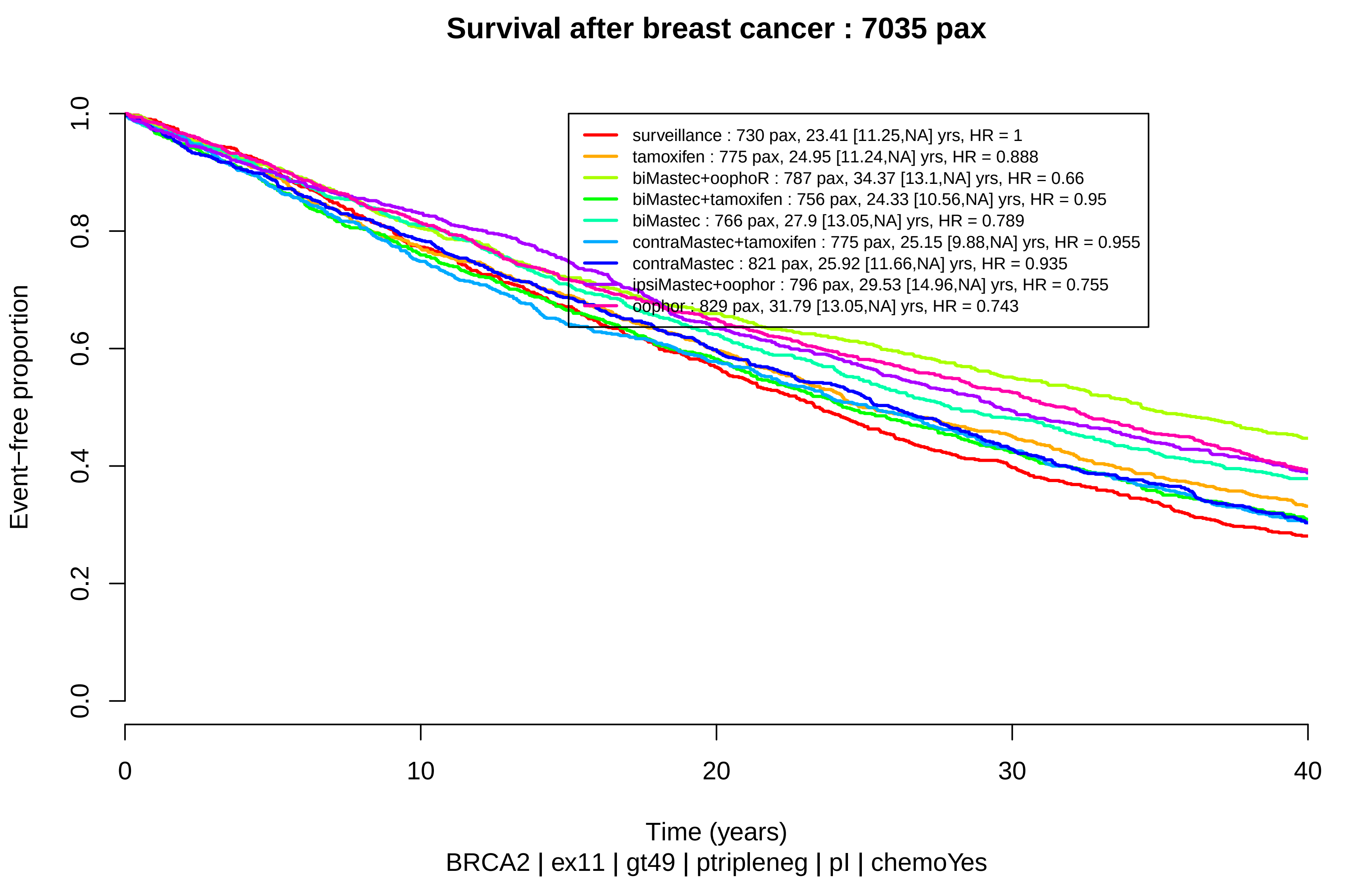

Survival after breast cancer : 7035 pax
surveillance : 730 pax, 23.41 [11.25,NA] yrs, HR = 1
tamoxifen : 775 pax, 24.95 [11.24,NA] yrs, HR = 0.888
biMastec+oophoR : 787 pax, 34.37 [13.1,NA] yrs, HR = 0.66
biMastec+tamoxifen : 756 pax, 24.33 [10.56,NA] yrs, HR = 0.95
biMastec : 766 pax, 27.9 [13.05,NA] yrs, HR = 0.789
contraMastec+tamoxifen : 775 pax, 25.15 [9.88,NA] yrs, HR = 0.955
contraMastec : 821 pax, 25.92 [11.66,NA] yrs, HR = 0.935
ipsiMastec+oophor : 796 pax, 29.53 [14.96,NA] yrs, HR = 0.755
oophor : 829 pax, 31.79 [13.05,NA] yrs, HR = 0.743
Event−free proportion
0.0
0.2
0.4
0.6
0.8
1.0
0
10
20
30
40
Time (years)
BRCA2 | ex11 | gt49 | ptripleneg | pI | chemoYes

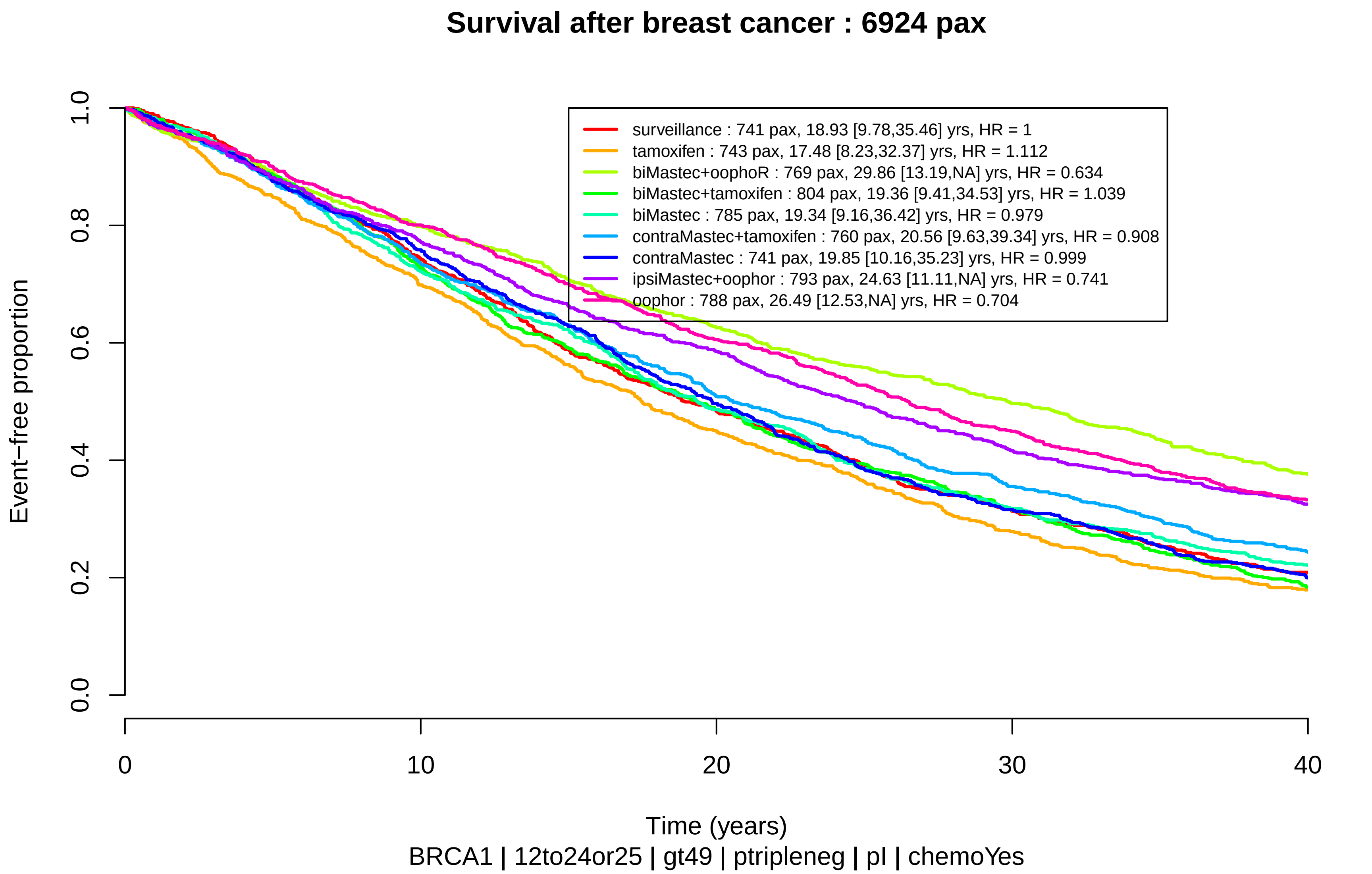
Survival after breast cancer : 6924 pax
surveillance : 741 pax, 18.93 [9.78,35.46] yrs, HR = 1
tamoxifen : 743 pax, 17.48 [8.23,32.37] yrs, HR = 1.112
biMastec+oophoR : 769 pax, 29.86 [13.19,NA] yrs, HR = 0.634
biMastec+tamoxifen : 804 pax, 19.36 [9.41,34.53] yrs, HR = 1.039
biMastec : 785 pax, 19.34 [9.16,36.42] yrs, HR = 0.979
contraMastec+tamoxifen : 760 pax, 20.56 [9.63,39.34] yrs, HR = 0.908
contraMastec : 741 pax, 19.85 [10.16,35.23] yrs, HR = 0.999
ipsiMastec+oophor : 793 pax, 24.63 [11.11,NA] yrs, HR = 0.741
oophor : 788 pax, 26.49 [12.53,NA] yrs, HR = 0.704
Event−free proportion
0.0
0.2
0.4
0.6
0.8
1.0
0
10
20
30
40
Time (years)
BRCA1 | 12to24or25 | gt49 | ptripleneg | pl | chemoYes

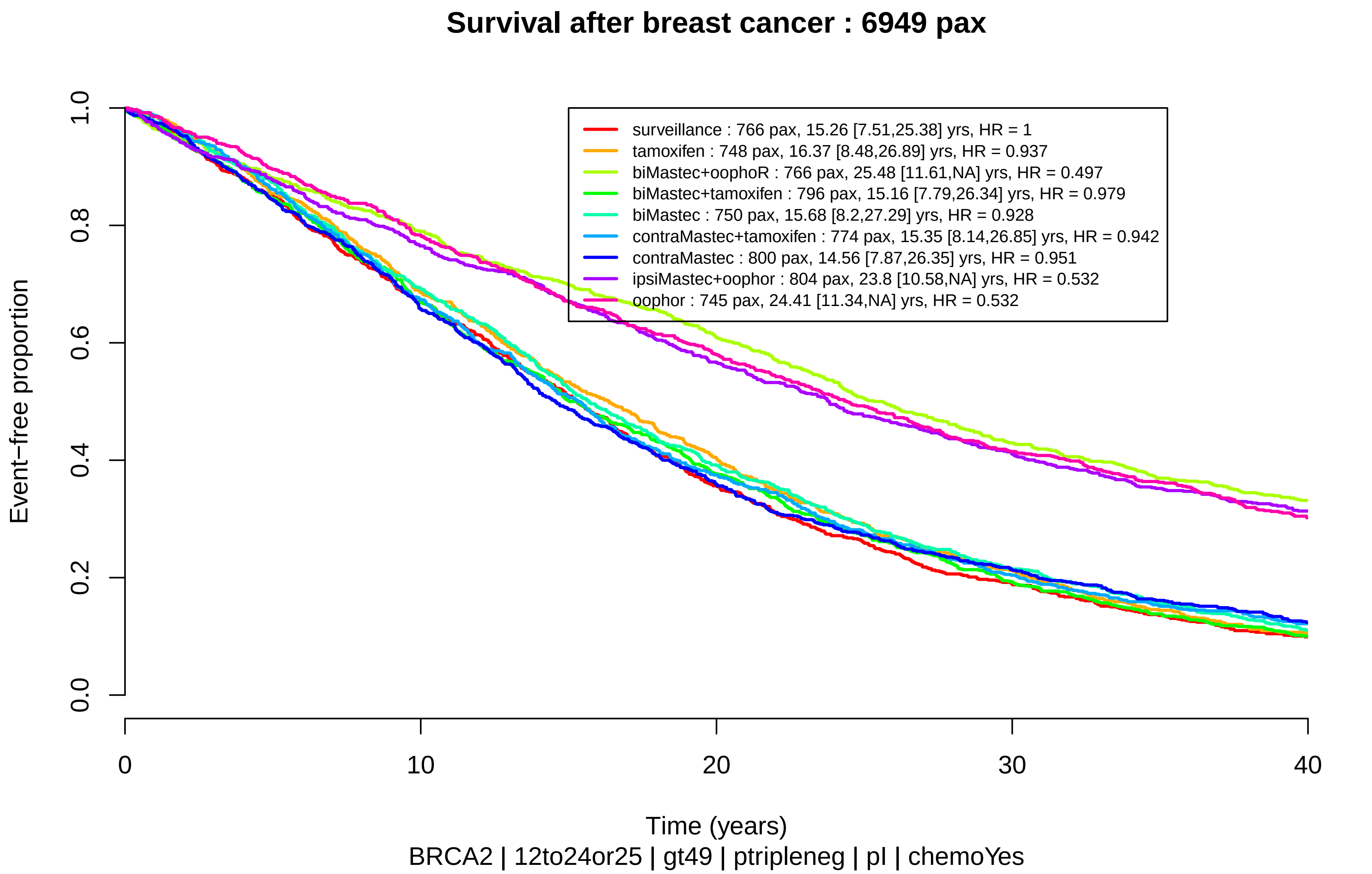

Survival after breast cancer : 6949 pax
surveillance : 766 pax, 15.26 [7.51,25.38] yrs, HR = 1
tamoxifen : 748 pax, 16.37 [8.48,26.89] yrs, HR = 0.937
biMastec+oophoR : 766 pax, 25.48 [11.61,NA] yrs, HR = 0.497
biMastec+tamoxifen : 796 pax, 15.16 [7.79,26.34] yrs, HR = 0.979
biMastec : 750 pax, 15.68 [8.2,27.29] yrs, HR = 0.928
contraMastec+tamoxifen : 774 pax, 15.35 [8.14,26.85] yrs, HR = 0.942
contraMastec : 800 pax, 14.56 [7.87,26.35] yrs, HR = 0.951
ipsiMastec+oophor : 804 pax, 23.8 [10.58,NA] yrs, HR = 0.532
oophor : 745 pax, 24.41 [11.34,NA] yrs, HR = 0.532
Event−free proportion
0.0
0.2
0.4
0.6
0.8
1.0
0
10
20
30
40
Time (years)
BRCA2 | 12to24or25 | gt49 | ptripleneg | pl | chemoYes

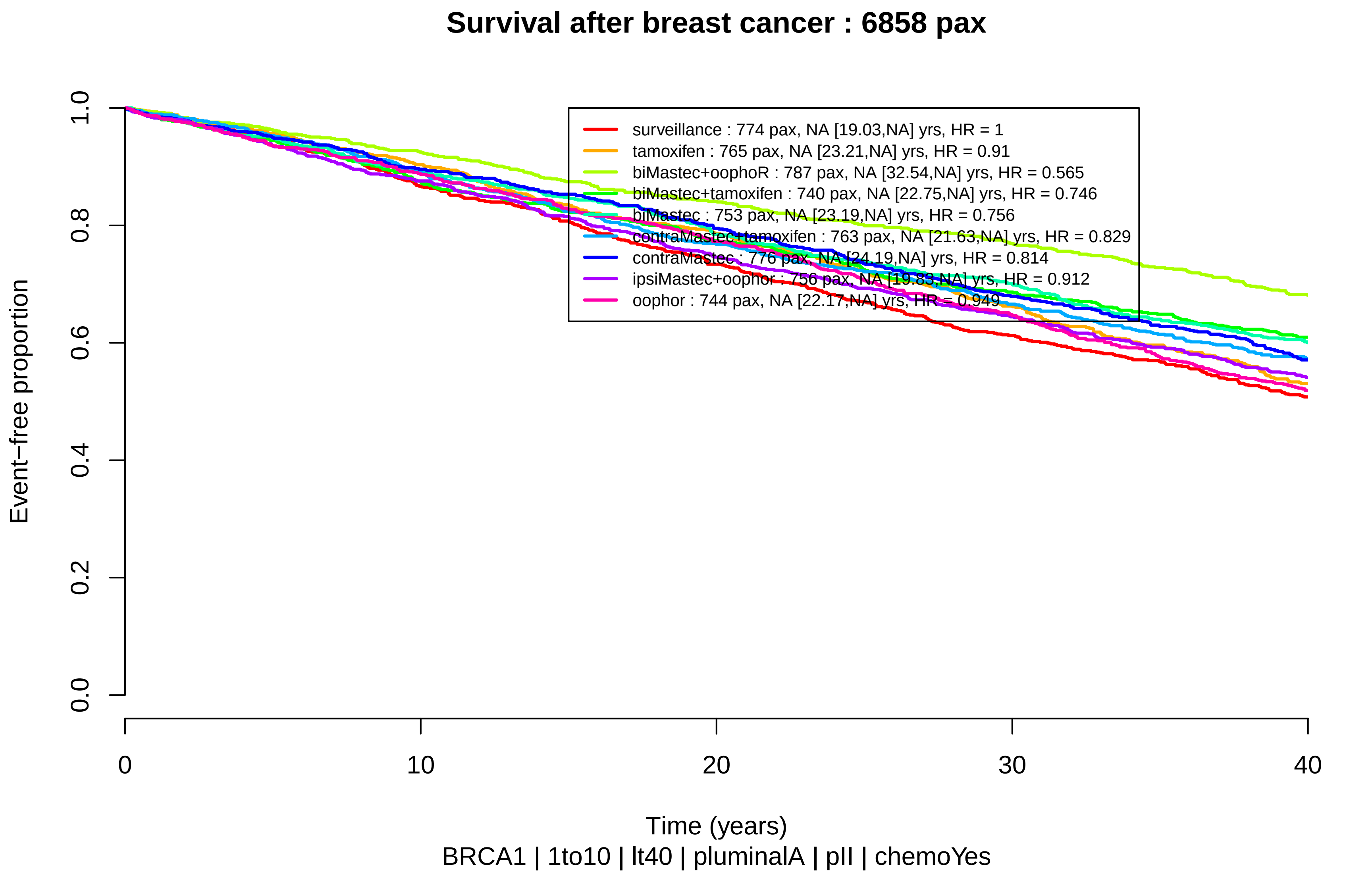
Survival after breast cancer : 6858 pax
surveillance : 774 pax, NA [19.03,NA] yrs, HR = 1
tamoxifen : 765 pax, NA [23.21,NA] yrs, HR = 0.91
biMastec+oophoR : 787 pax, NA [32.54,NA] yrs, HR = 0.565
biMastec+tamoxifen : 740 pax, NA [22.75,NA] yrs, HR = 0.746
biMastec : 753 pax, NA [23.19,NA] yrs, HR = 0.756
contraMastec+tamoxifen : 763 pax, NA [21.63,NA] yrs, HR = 0.829
contraMastec : 776 pax, NA [24.19,NA] yrs, HR = 0.814
ipsiMastec+oophor : 756 pax, NA [19.83,NA] yrs, HR = 0.912
oophor : 744 pax, NA [22.17,NA] yrs, HR = 0.949
Event−free proportion
0.0
0.2
0.4
0.6
0.8
1.0
0
10
20
30
40
Time (years)
BRCA1 | 1to10 | lt40 | pluminalA | pII | chemoYes

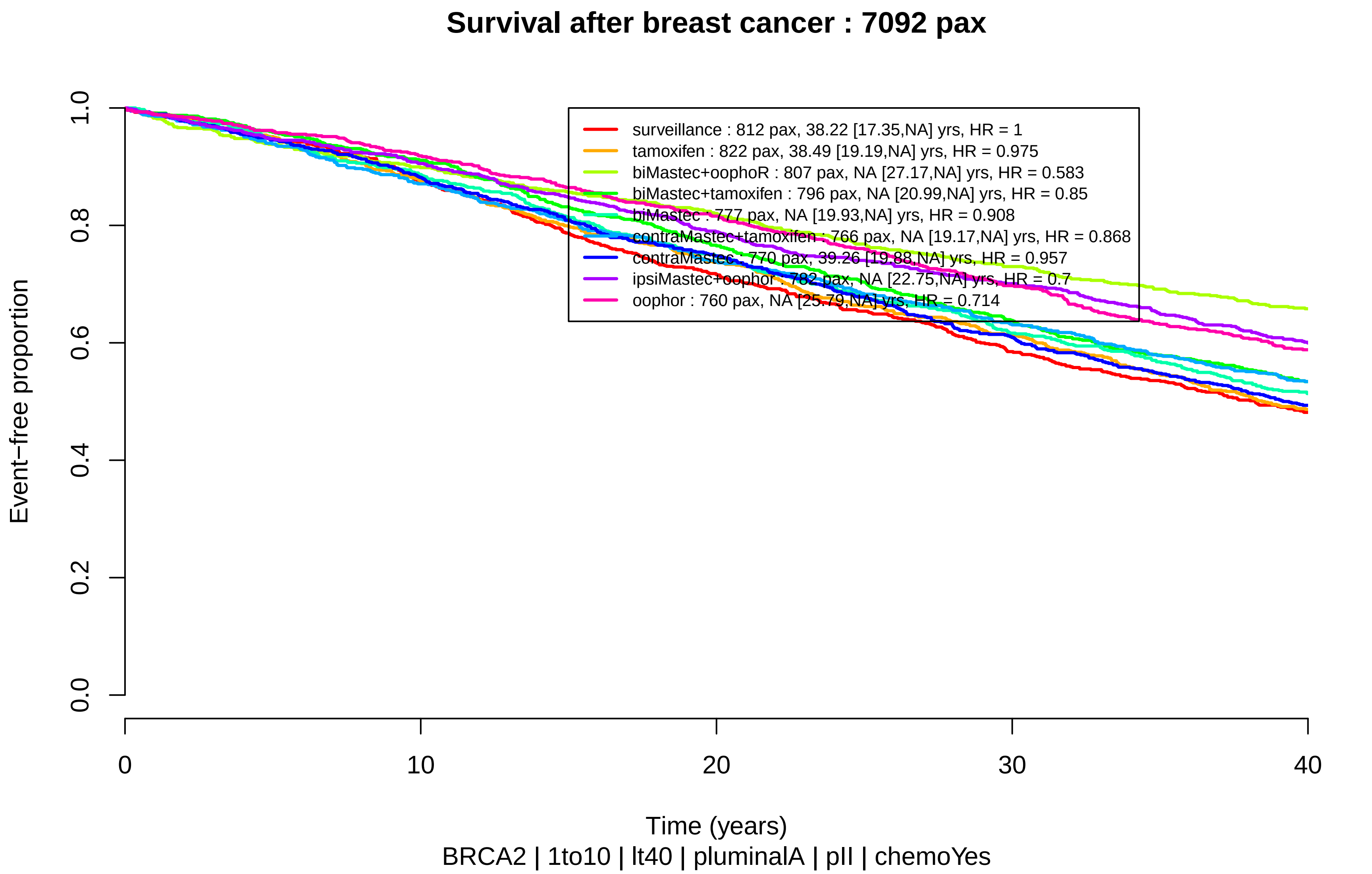

Survival after breast cancer : 7092 pax
surveillance : 812 pax, 38.22 [17.35,NA] yrs, HR = 1
tamoxifen : 822 pax, 38.49 [19.19,NA] yrs, HR = 0.975
biMastec+oophoR : 807 pax, NA [27.17,NA] yrs, HR = 0.583
biMastec+tamoxifen : 796 pax, NA [20.99,NA] yrs, HR = 0.85
biMastec : 777 pax, NA [19.93,NA] yrs, HR = 0.908
contraMastec+tamoxifen : 766 pax, NA [19.17,NA] yrs, HR = 0.868
contraMastec : 770 pax, 39.26 [19.88,NA] yrs, HR = 0.957
ipsiMastec+oophor : 782 pax, NA [22.75,NA] yrs, HR = 0.7
oophor : 760 pax, NA [25.79,NA] yrs, HR = 0.714
Event−free proportion
0.0
0.2
0.4
0.6
0.8
1.0
0
10
20
30
40
Time (years)
BRCA2 | 1to10 | lt40 | pluminalA | pII | chemoYes

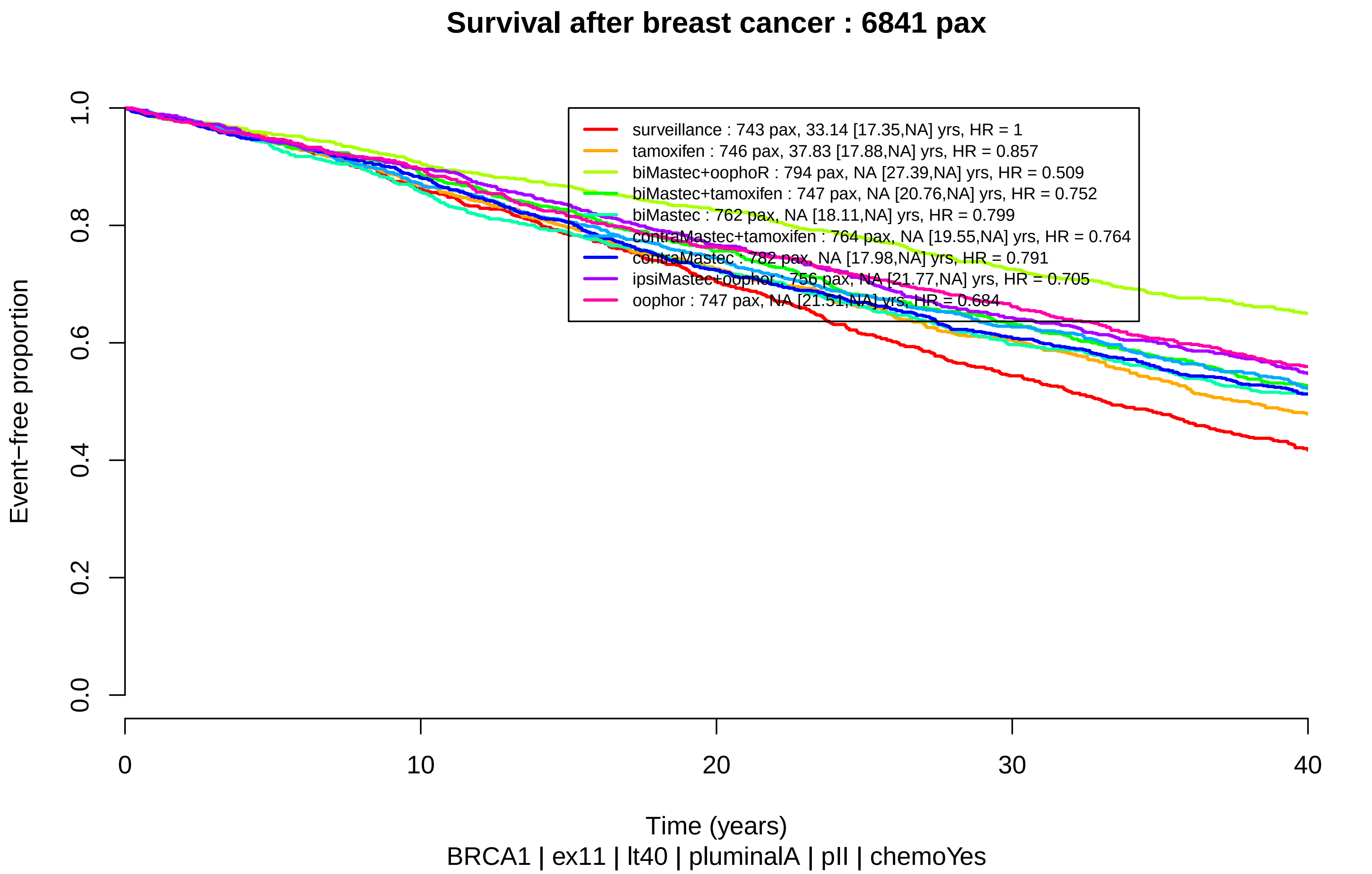
Survival after breast cancer : 6841 pax
surveillance : 743 pax, 33.14 [17.35,NA] yrs, HR = 1
tamoxifen : 746 pax, 37.83 [17.88,NA] yrs, HR = 0.857
biMastec+oophoR : 794 pax, NA [27.39,NA] yrs, HR = 0.509
biMastec+tamoxifen : 747 pax, NA [20.76,NA] yrs, HR = 0.752
biMastec : 762 pax, NA [18.11,NA] yrs, HR = 0.799
contraMastec+tamoxifen : 764 pax, NA [19.55,NA] yrs, HR = 0.764
contraMastec : 782 pax, NA [17.98,NA] yrs, HR = 0.791
ipsiMastec+oophor : 756 pax, NA [21.77,NA] yrs, HR = 0.705
oophor : 747 pax, NA [21.51,NA] yrs, HR = 0.684
Event−free proportion
0.0
0.2
0.4
0.6
0.8
1.0
0
10
20
30
40
Time (years)
BRCA1 | ex11 | lt40 | pluminalA | pII | chemoYes

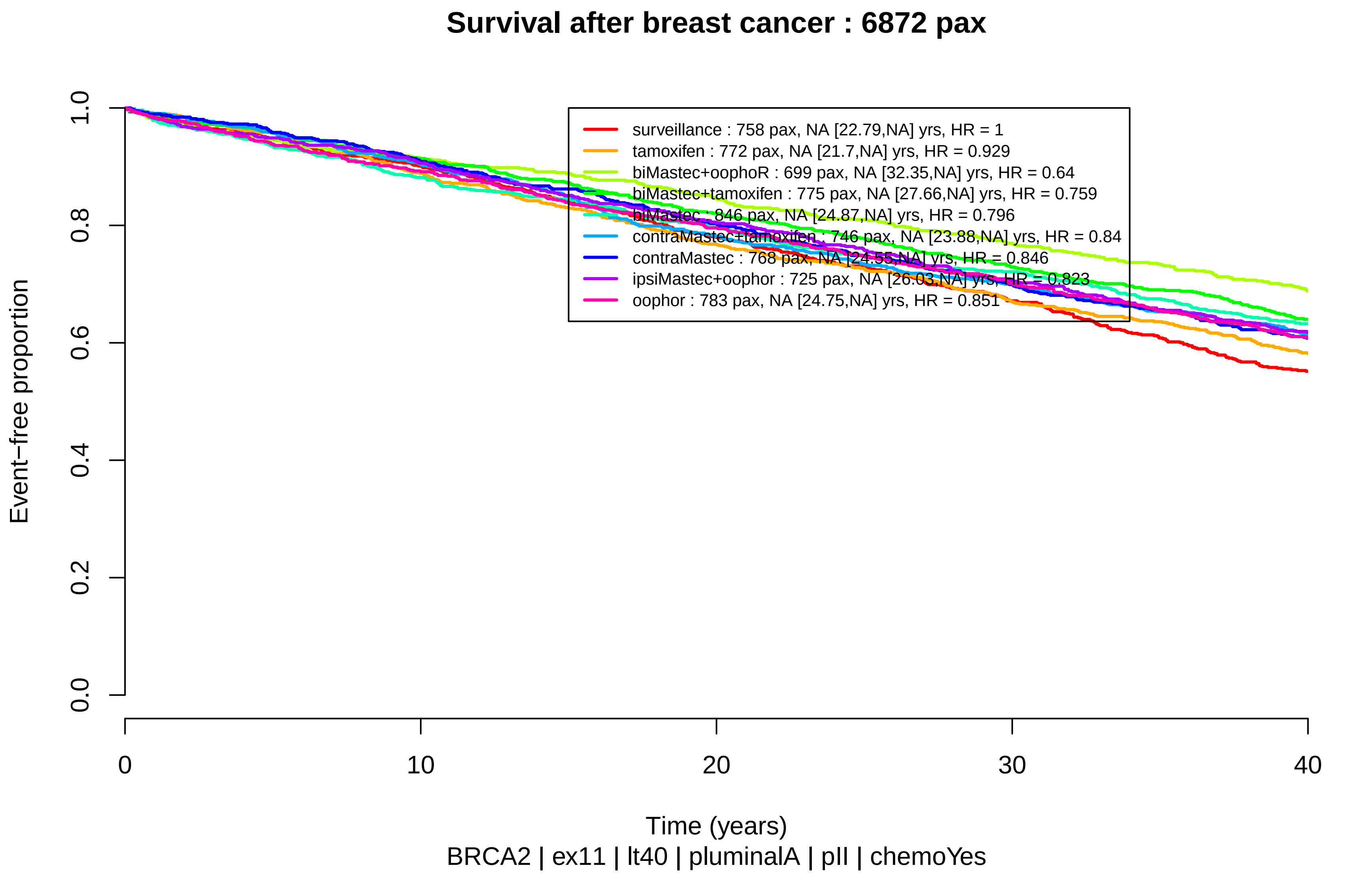

Survival after breast cancer : 6872 pax
surveillance : 758 pax, NA [22.79,NA] yrs, HR = 1
tamoxifen : 772 pax, NA [21.7,NA] yrs, HR = 0.929
biMastec+oophoR : 699 pax, NA [32.35,NA] yrs, HR = 0.64
biMastec+tamoxifen : 775 pax, NA [27.66,NA] yrs, HR = 0.759
biMastec : 846 pax, NA [24.87,NA] yrs, HR = 0.796
contraMastec+tamoxifen : 746 pax, NA [23.88,NA] yrs, HR = 0.84
contraMastec : 768 pax, NA [24.55,NA] yrs, HR = 0.846
ipsiMastec+oophor : 725 pax, NA [26.03,NA] yrs, HR = 0.823
oophor : 783 pax, NA [24.75,NA] yrs, HR = 0.851
Event−free proportion
0.0
0.2
0.4
0.6
0.8
1.0
0
10
20
30
40
Time (years)
BRCA2 | ex11 | lt40 | pluminalA | pII | chemoYes

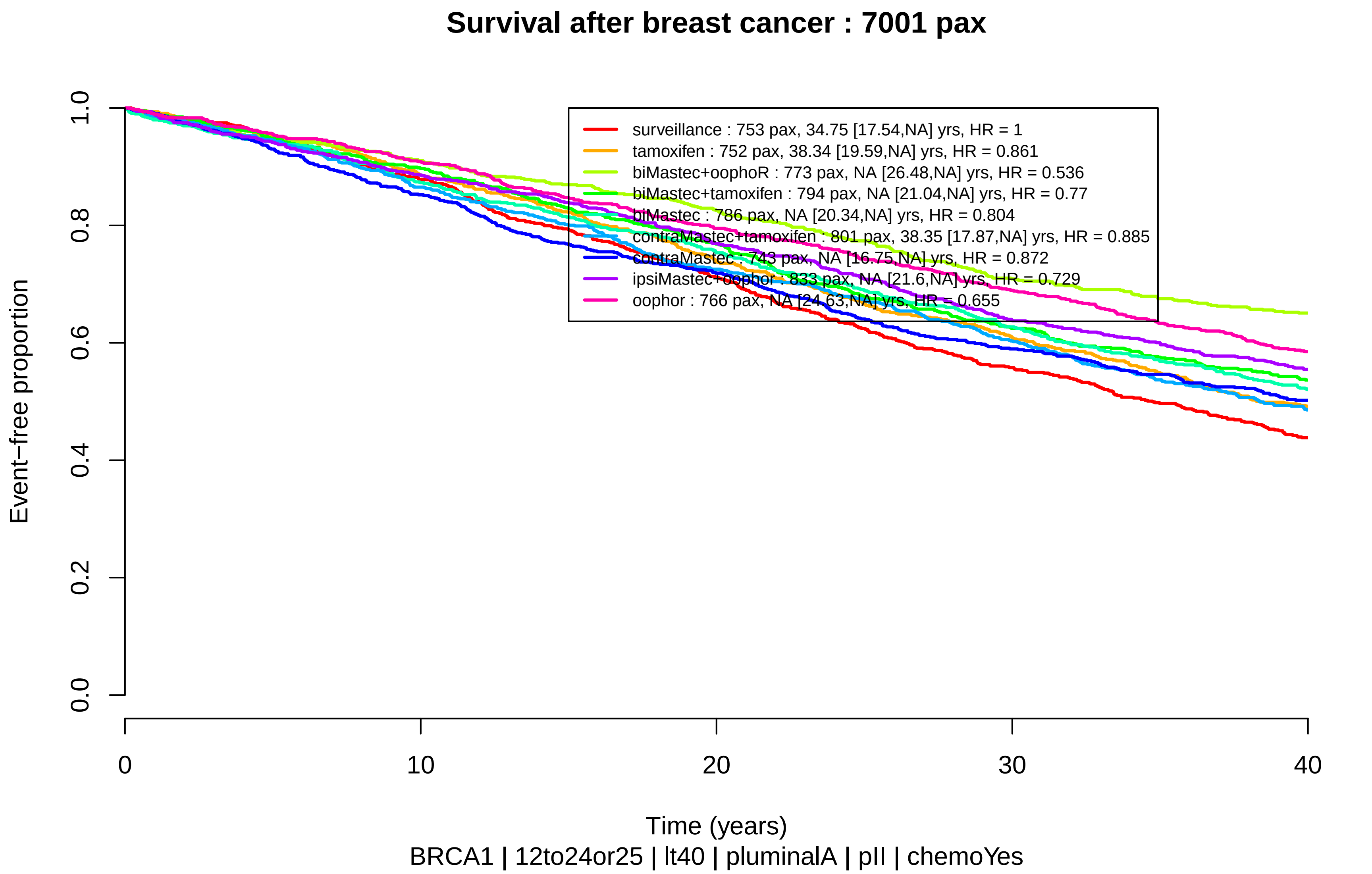

Survival after breast cancer : 7001 pax
surveillance : 753 pax, 34.75 [17.54,NA] yrs, HR = 1
tamoxifen : 752 pax, 38.34 [19.59,NA] yrs, HR = 0.861
biMastec+oophoR : 773 pax, NA [26.48,NA] yrs, HR = 0.536
biMastec+tamoxifen : 794 pax, NA [21.04,NA] yrs, HR = 0.77
biMastec : 786 pax, NA [20.34,NA] yrs, HR = 0.804
contraMastec+tamoxifen : 801 pax, 38.35 [17.87,NA] yrs, HR = 0.885
contraMastec : 743 pax, NA [16.75,NA] yrs, HR = 0.872
ipsiMastec+oophor : 833 pax, NA [21.6,NA] yrs, HR = 0.729
oophor : 766 pax, NA [24.63,NA] yrs, HR = 0.655
Event−free proportion
0.0
0.2
0.4
0.6
0.8
1.0
0
10
20
30
40
Time (years)
BRCA1 | 12to24or25 | lt40 | pluminalA | pII | chemoYes

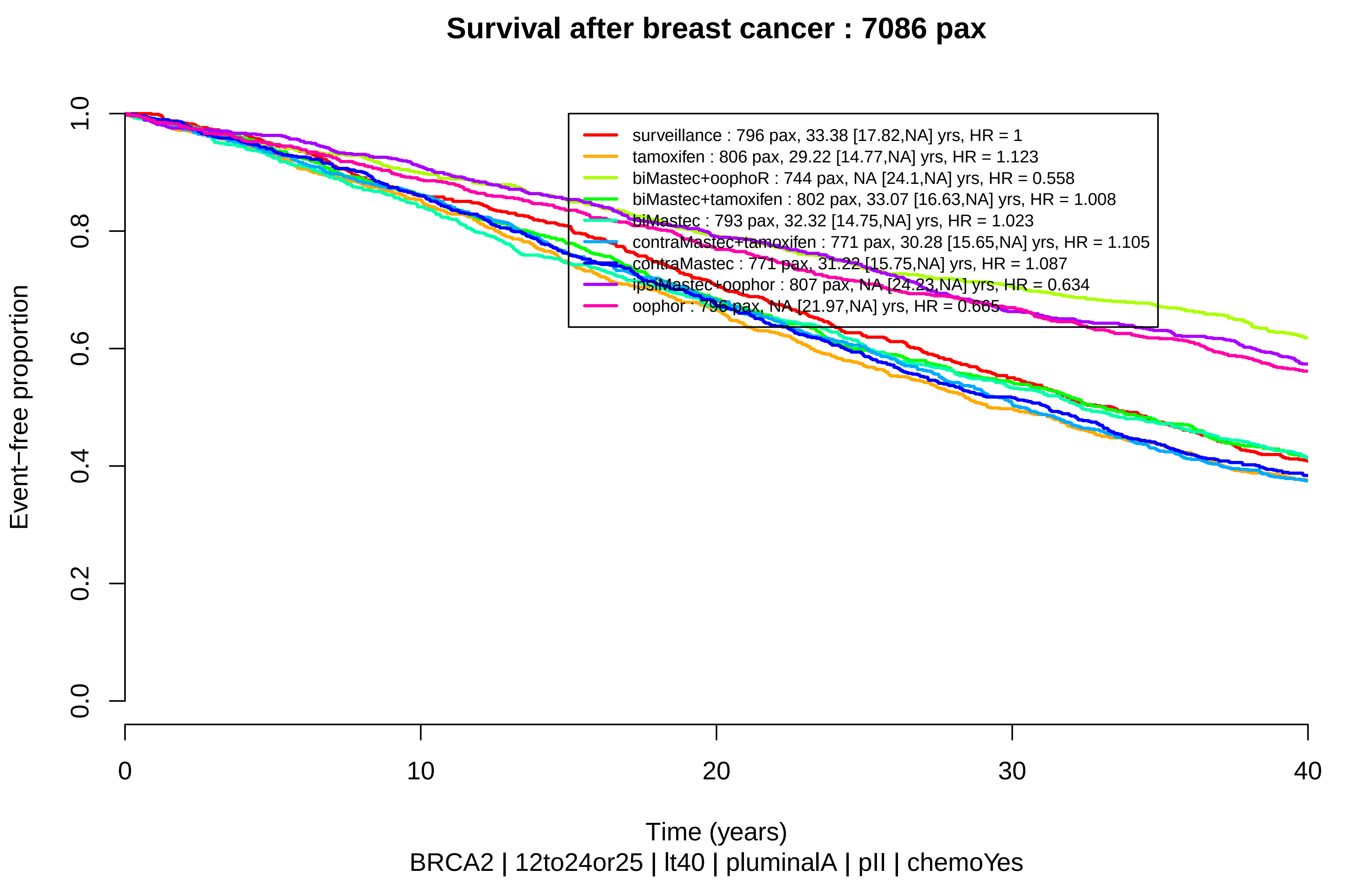

Survival after breast cancer : 7086 pax
surveillance : 796 pax, 33.38 [17.82,NA] yrs, HR = 1
tamoxifen : 806 pax, 29.22 [14.77,NA] yrs, HR = 1.123
biMastec+oophoR : 744 pax, NA [24.1,NA] yrs, HR = 0.558
biMastec+tamoxifen : 802 pax, 33.07 [16.63,NA] yrs, HR = 1.008
biMastec : 793 pax, 32.32 [14.75,NA] yrs, HR = 1.023
contraMastec+tamoxifen : 771 pax, 30.28 [15.65,NA] yrs, HR = 1.105
contraMastec : 771 pax, 31.22 [15.75,NA] yrs, HR = 1.087
ipsiMastec+oophor : 807 pax, NA [24.23,NA] yrs, HR = 0.634
oophor : 796 pax, NA [21.97,NA] yrs, HR = 0.665
Event−free proportion
0.0
0.2
0.4
0.6
0.8
1.0
0
10
20
30
40
Time (years)
BRCA2 | 12to24or25 | lt40 | pluminalA | pII | chemoYes

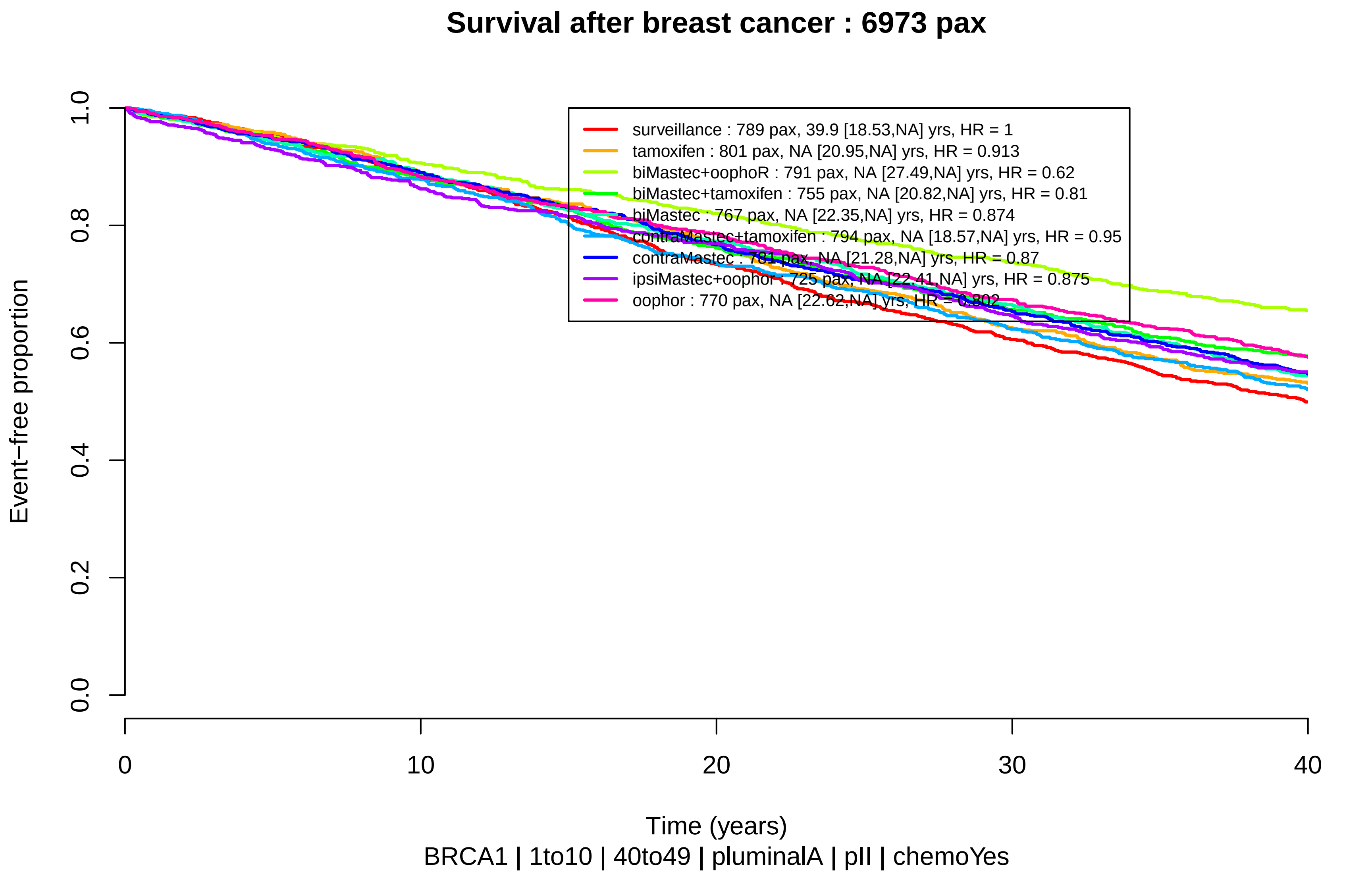
Survival after breast cancer : 6973 pax
surveillance : 789 pax, 39.9 [18.53,NA] yrs, HR = 1
tamoxifen : 801 pax, NA [20.95,NA] yrs, HR = 0.913
biMastec+oophoR : 791 pax, NA [27.49,NA] yrs, HR = 0.62
biMastec+tamoxifen : 755 pax, NA [20.82,NA] yrs, HR = 0.81
biMastec : 767 pax, NA [22.35,NA] yrs, HR = 0.874
contraMastec+tamoxifen : 794 pax, NA [18.57,NA] yrs, HR = 0.95
contraMastec : 781 pax, NA [21.28,NA] yrs, HR = 0.87
ipsiMastec+oophor : 725 pax, NA [22.41,NA] yrs, HR = 0.875
oophor : 770 pax, NA [22.62,NA] yrs, HR = 0.802
Event−free proportion
0.0
0.2
0.4
0.6
0.8
1.0
0
10
20
30
40
Time (years)
BRCA1 | 1to10 | 40to49 | pluminalA | pII | chemoYes

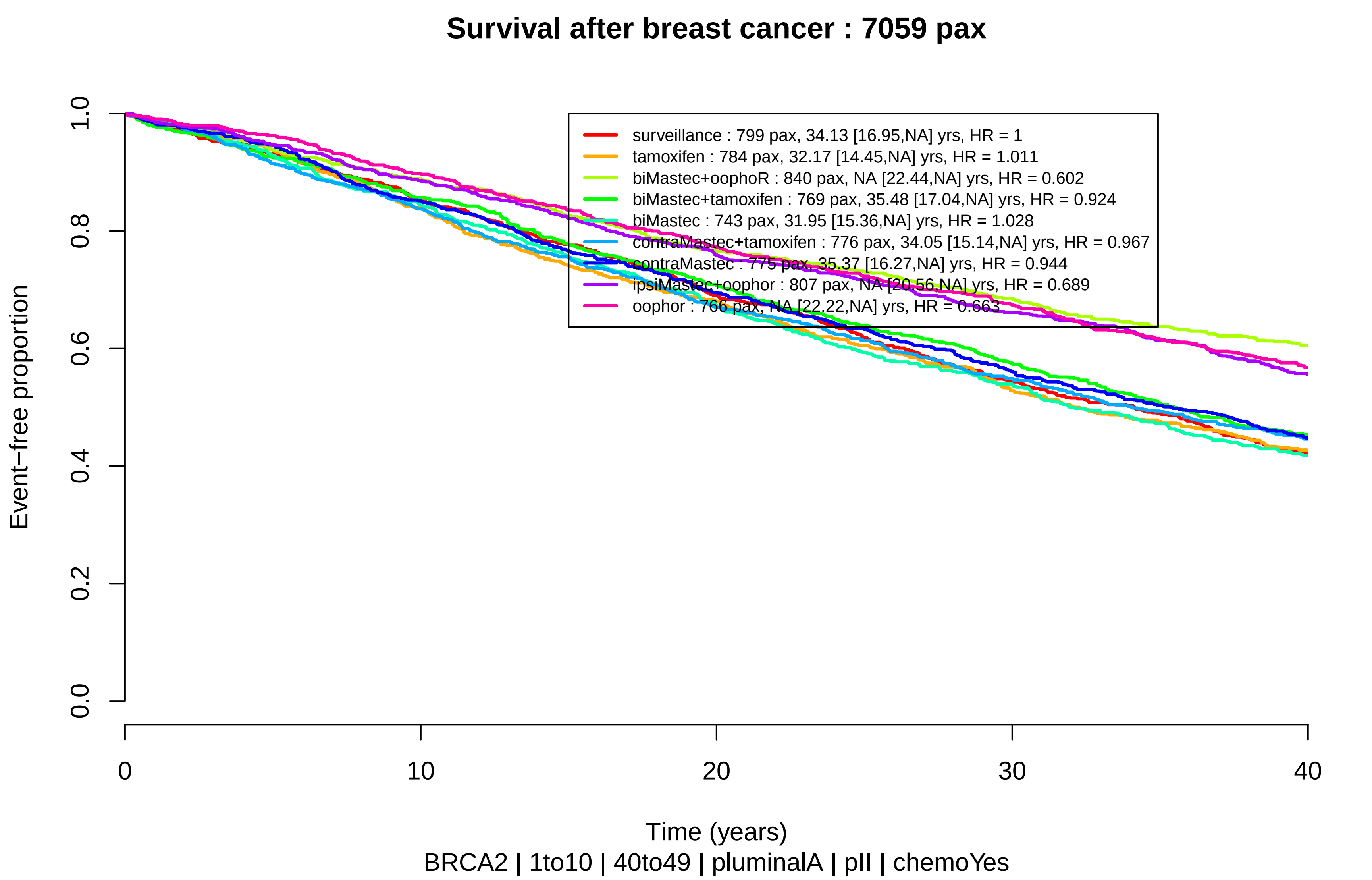

Survival after breast cancer : 7059 pax
surveillance : 799 pax, 34.13 [16.95,NA] yrs, HR = 1
tamoxifen : 784 pax, 32.17 [14.45,NA] yrs, HR = 1.011
biMastec+oophoR : 840 pax, NA [22.44,NA] yrs, HR = 0.602
biMastec+tamoxifen : 769 pax, 35.48 [17.04,NA] yrs, HR = 0.924
biMastec : 743 pax, 31.95 [15.36,NA] yrs, HR = 1.028
contraMastec+tamoxifen : 776 pax, 34.05 [15.14,NA] yrs, HR = 0.967
contraMastec : 775 pax, 35.37 [16.27,NA] yrs, HR = 0.944
ipsiMastec+oophor : 807 pax, NA [20.56,NA] yrs, HR = 0.689
oophor : 766 pax, NA [22.22,NA] yrs, HR = 0.663
Event−free proportion
0.0
0.2
0.4
0.6
0.8
1.0
0
10
20
30
40
Time (years)
BRCA2 | 1to10 | 40to49 | pluminalA | pII | chemoYes

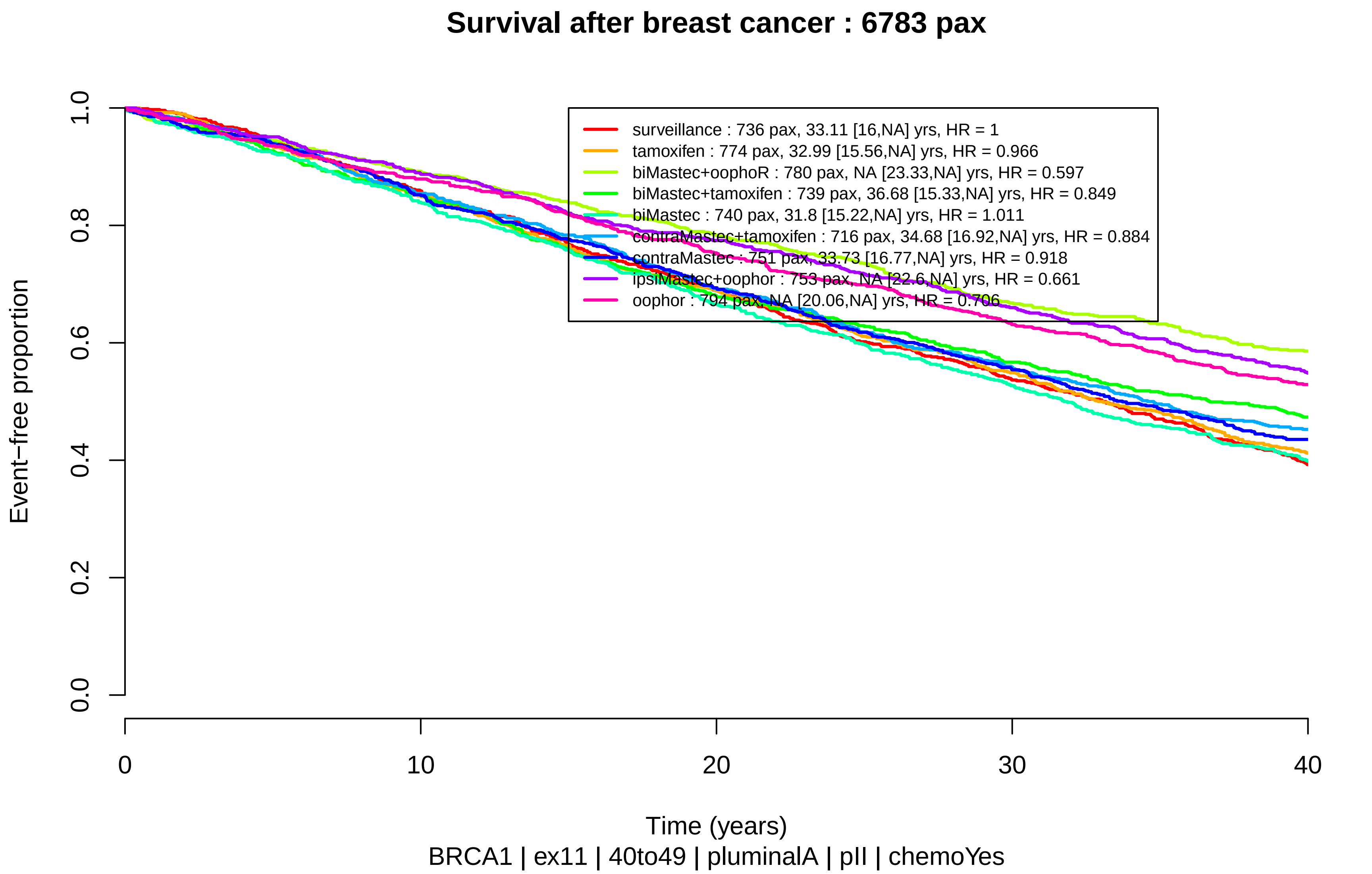

Survival after breast cancer : 6783 pax
surveillance : 736 pax, 33.11 [16,NA] yrs, HR = 1
tamoxifen : 774 pax, 32.99 [15.56,NA] yrs, HR = 0.966
biMastec+oophoR : 780 pax, NA [23.33,NA] yrs, HR = 0.597
biMastec+tamoxifen : 739 pax, 36.68 [15.33,NA] yrs, HR = 0.849
biMastec : 740 pax, 31.8 [15.22,NA] yrs, HR = 1.011
contraMastec+tamoxifen : 716 pax, 34.68 [16.92,NA] yrs, HR = 0.884
contraMastec : 751 pax, 33.73 [16.77,NA] yrs, HR = 0.918
ipsiMastec+oophor : 753 pax, NA [22.6,NA] yrs, HR = 0.661
oophor : 794 pax, NA [20.06,NA] yrs, HR = 0.706
Event−free proportion
0.0
0.2
0.4
0.6
0.8
1.0
0
10
20
30
40
Time (years)
BRCA1 | ex11 | 40to49 | pluminalA | pII | chemoYes

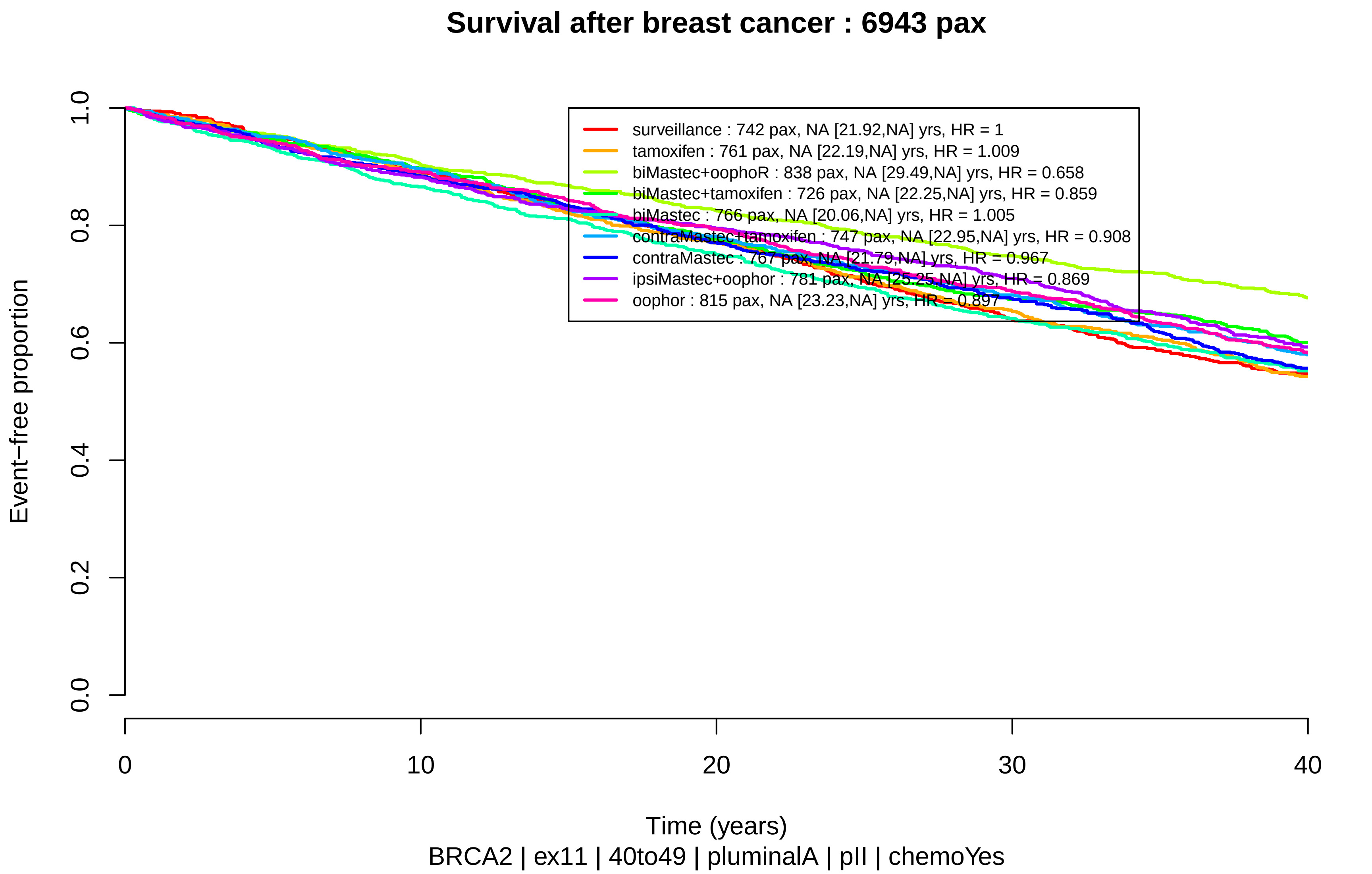
Survival after breast cancer : 6943 pax
Event−free proportion
0.0
0.2
0.4
0.6
0.8
1.0
0
10
20
30
40
Time (years)
BRCA2 | ex11 | 40to49 | pluminalA | pII | chemoYes
surveillance : 742 pax, NA [21.92,NA] yrs, HR = 1
tamoxifen : 761 pax, NA [22.19,NA] yrs, HR = 1.009
biMastec+oophoR : 838 pax, NA [29.49,NA] yrs, HR = 0.658
biMastec+tamoxifen : 726 pax, NA [22.25,NA] yrs, HR = 0.859
biMastec : 766 pax, NA [20.06,NA] yrs, HR = 1.005
contraMastec+tamoxifen : 747 pax, NA [22.95,NA] yrs, HR = 0.908
contraMastec : 767 pax, NA [21.79,NA] yrs, HR = 0.967
ipsiMastec+oophor : 781 pax, NA [25.25,NA] yrs, HR = 0.869
oophor : 815 pax, NA [23.23,NA] yrs, HR = 0.897

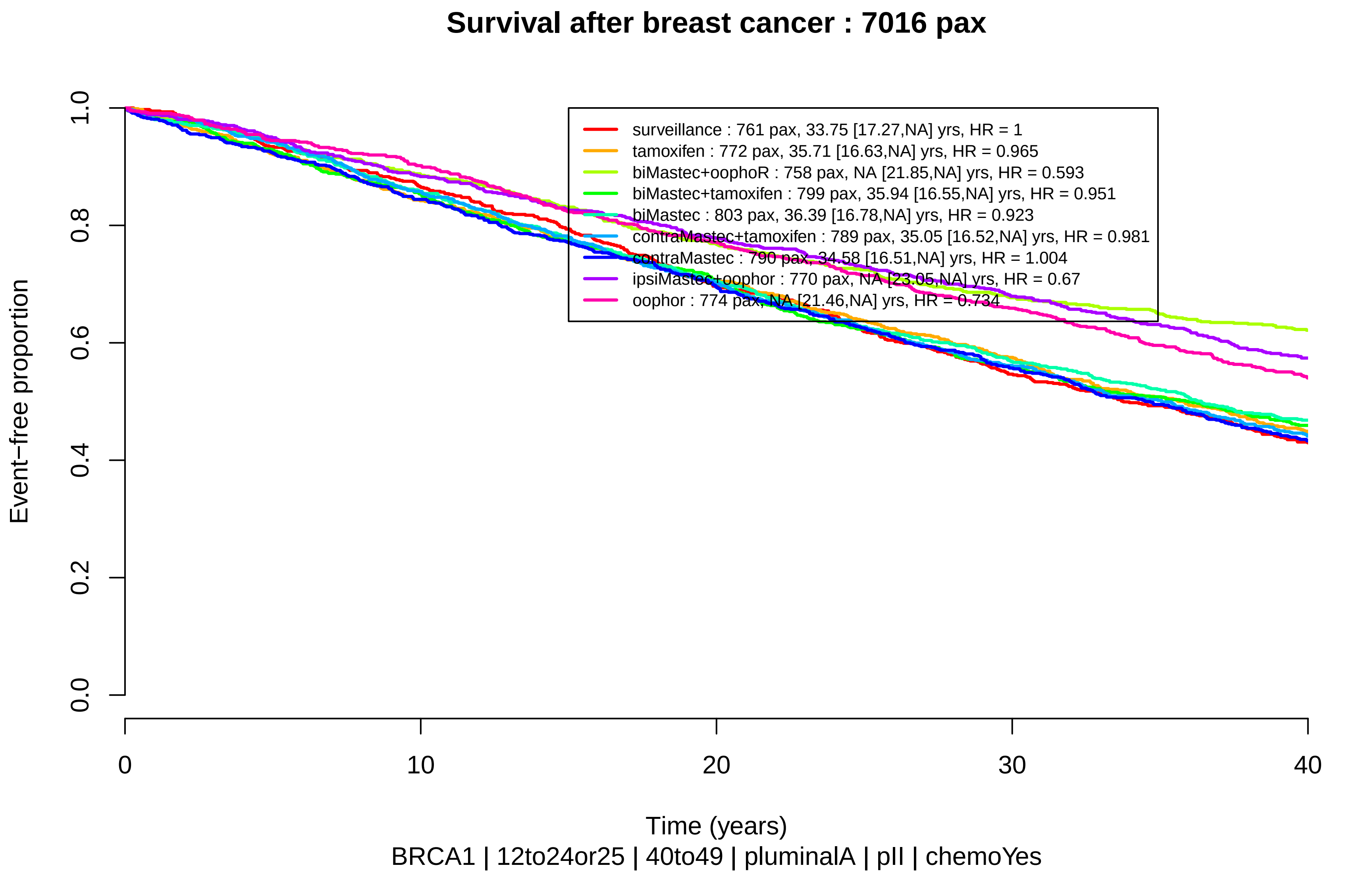

Survival after breast cancer : 7016 pax
Event−free proportion
0.0
0.2
0.4
0.6
0.8
1.0
0
10
20
30
40
Time (years)
BRCA1 | 12to24or25 | 40to49 | pluminalA | pII | chemoYes
surveillance : 761 pax, 33.75 [17.27,NA] yrs, HR = 1
tamoxifen : 772 pax, 35.71 [16.63,NA] yrs, HR = 0.965
biMastec+oophoR : 758 pax, NA [21.85,NA] yrs, HR = 0.593
biMastec+tamoxifen : 799 pax, 35.94 [16.55,NA] yrs, HR = 0.951
biMastec : 803 pax, 36.39 [16.78,NA] yrs, HR = 0.923
contraMastec+tamoxifen : 789 pax, 35.05 [16.52,NA] yrs, HR = 0.981
contraMastec : 790 pax, 34.58 [16.51,NA] yrs, HR = 1.004
ipsiMastec+oophor : 770 pax, NA [23.05,NA] yrs, HR = 0.67
oophor : 774 pax, NA [21.46,NA] yrs, HR = 0.734

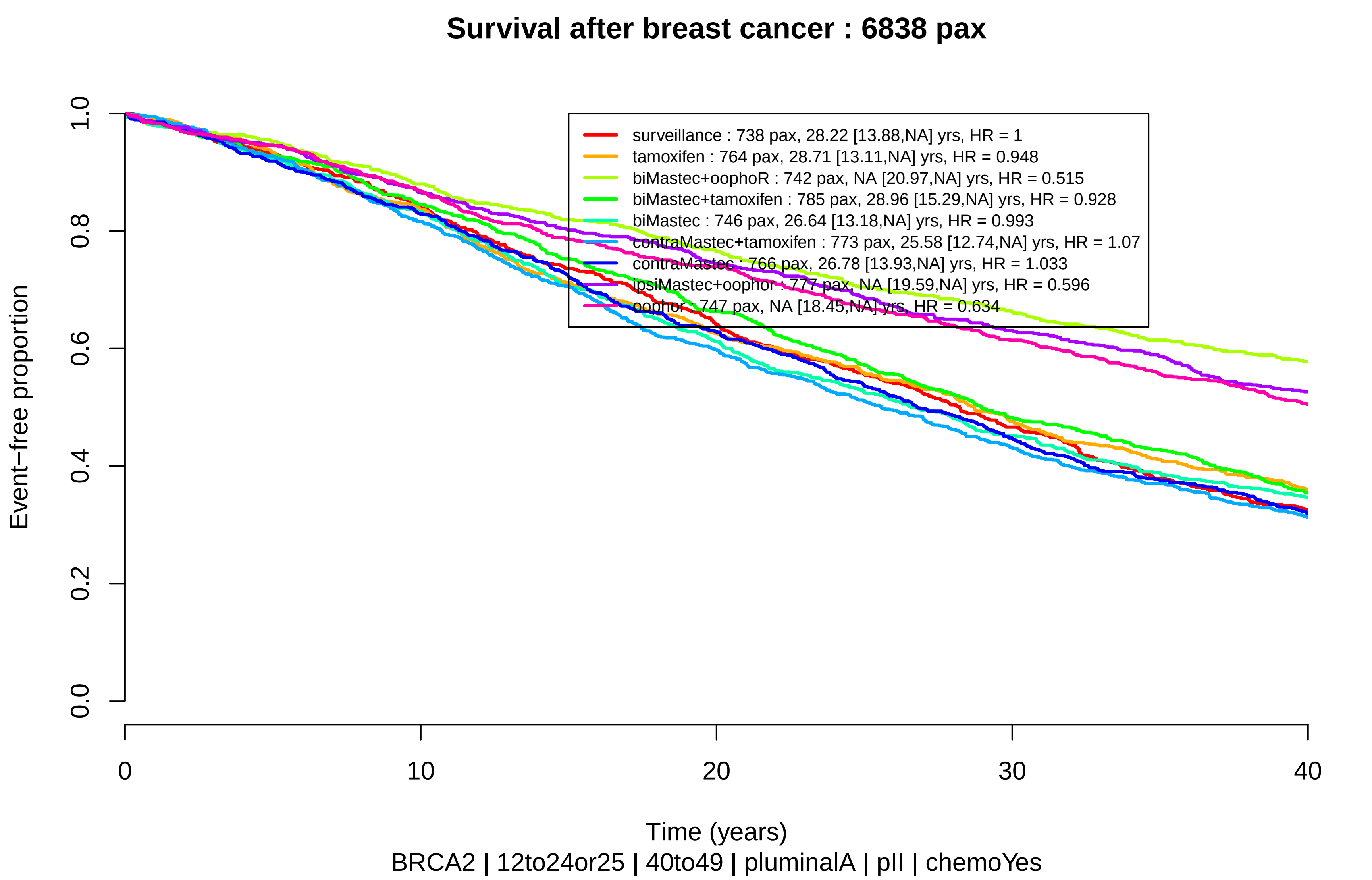

Survival after breast cancer : 6838 pax
surveillance : 738 pax, 28.22 [13.88,NA] yrs, HR = 1
tamoxifen : 764 pax, 28.71 [13.11,NA] yrs, HR = 0.948
biMastec+oophoR : 742 pax, NA [20.97,NA] yrs, HR = 0.515
biMastec+tamoxifen : 785 pax, 28.96 [15.29,NA] yrs, HR = 0.928
biMastec : 746 pax, 26.64 [13.18,NA] yrs, HR = 0.993
contraMastec+tamoxifen : 773 pax, 25.58 [12.74,NA] yrs, HR = 1.07
contraMastec : 766 pax, 26.78 [13.93,NA] yrs, HR = 1.033
ipsiMastec+oophor : 777 pax, NA [19.59,NA] yrs, HR = 0.596
oophor : 747 pax, NA [18.45,NA] yrs, HR = 0.634
Event−free proportion
0.0
0.2
0.4
0.6
0.8
1.0
0
10
20
30
40
Time (years)
BRCA2 | 12to24or25 | 40to49 | pluminalA | pII | chemoYes

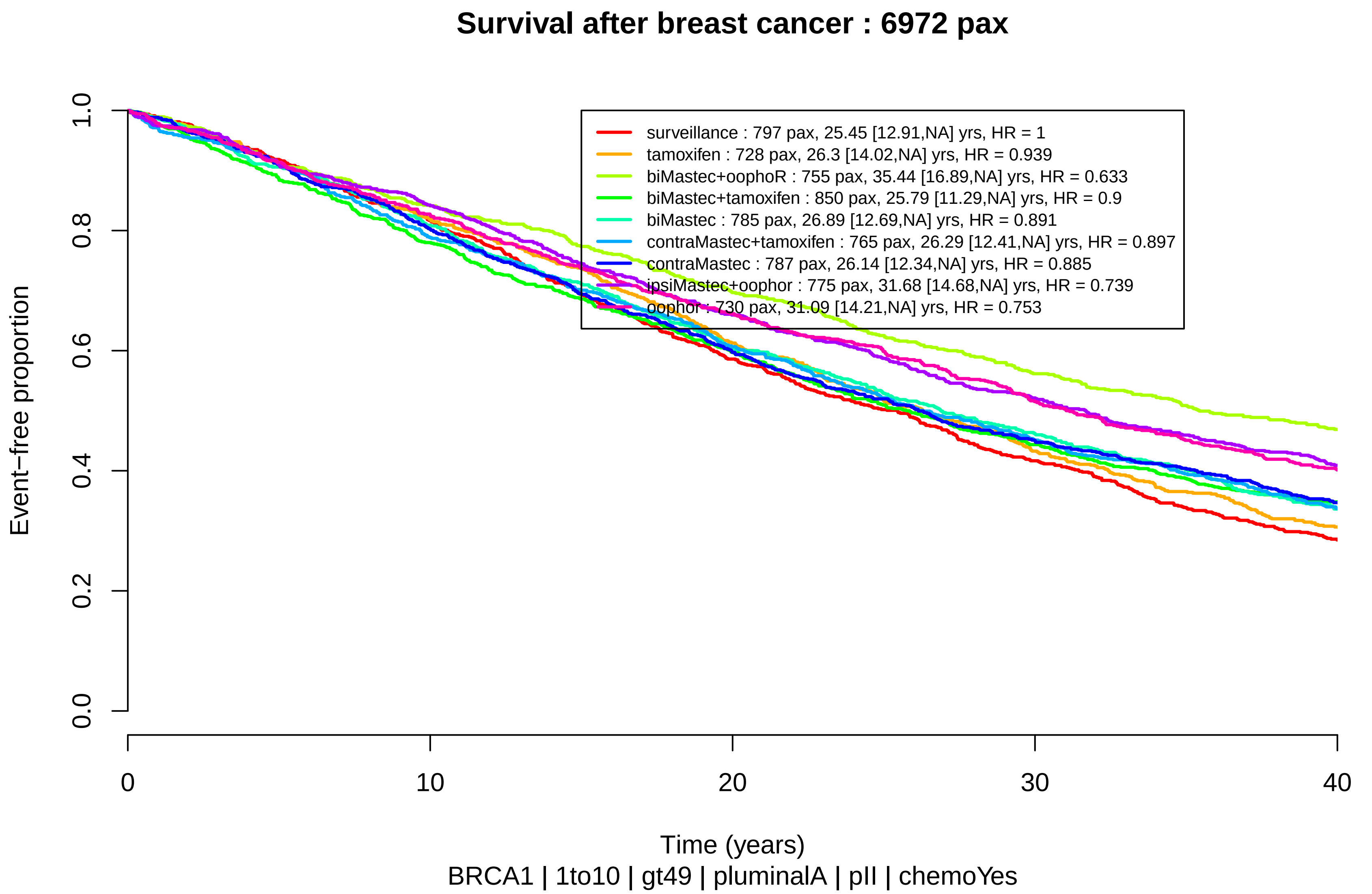

Survival after breast cancer : 6972 pax
surveillance : 797 pax, 25.45 [12.91,NA] yrs, HR = 1
tamoxifen : 728 pax, 26.3 [14.02,NA] yrs, HR = 0.939
biMastec+oophoR : 755 pax, 35.44 [16.89,NA] yrs, HR = 0.633
biMastec+tamoxifen : 850 pax, 25.79 [11.29,NA] yrs, HR = 0.9
biMastec : 785 pax, 26.89 [12.69,NA] yrs, HR = 0.891
contraMastec+tamoxifen : 765 pax, 26.29 [12.41,NA] yrs, HR = 0.897
contraMastec : 787 pax, 26.14 [12.34,NA] yrs, HR = 0.885
ipsiMastec+oophor : 775 pax, 31.68 [14.68,NA] yrs, HR = 0.739
oophor : 730 pax, 31.09 [14.21,NA] yrs, HR = 0.753
Event−free proportion
0.0
0.2
0.4
0.6
0.8
1.0
0
10
20
30
40
Time (years)
BRCA1 | 1to10 | gt49 | pluminalA | pII | chemoYes

Survival after breast cancer : 6914 pax

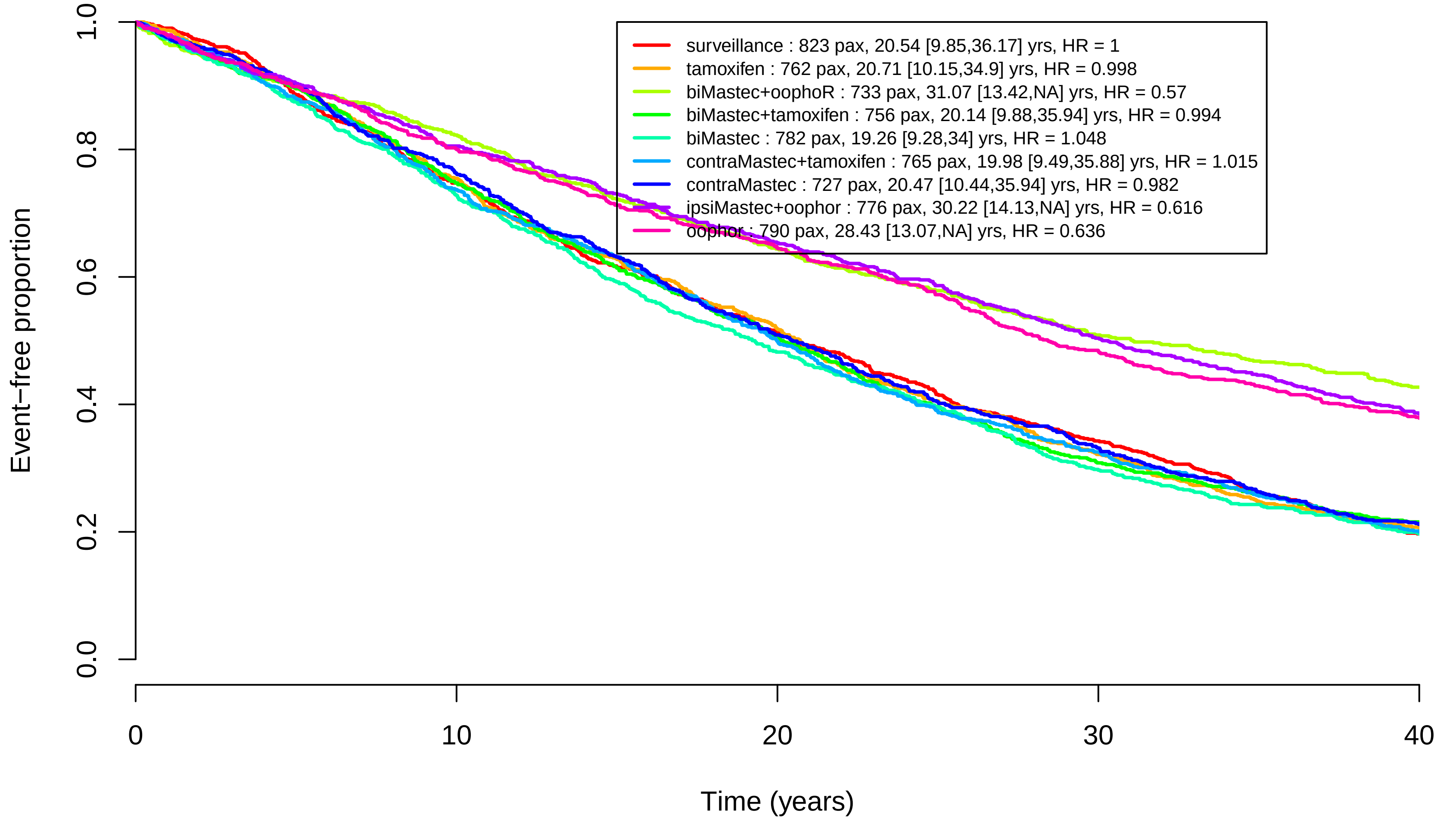

BRCA2 | 1to10 | gt49 | pluminalA | pII | chemoYes

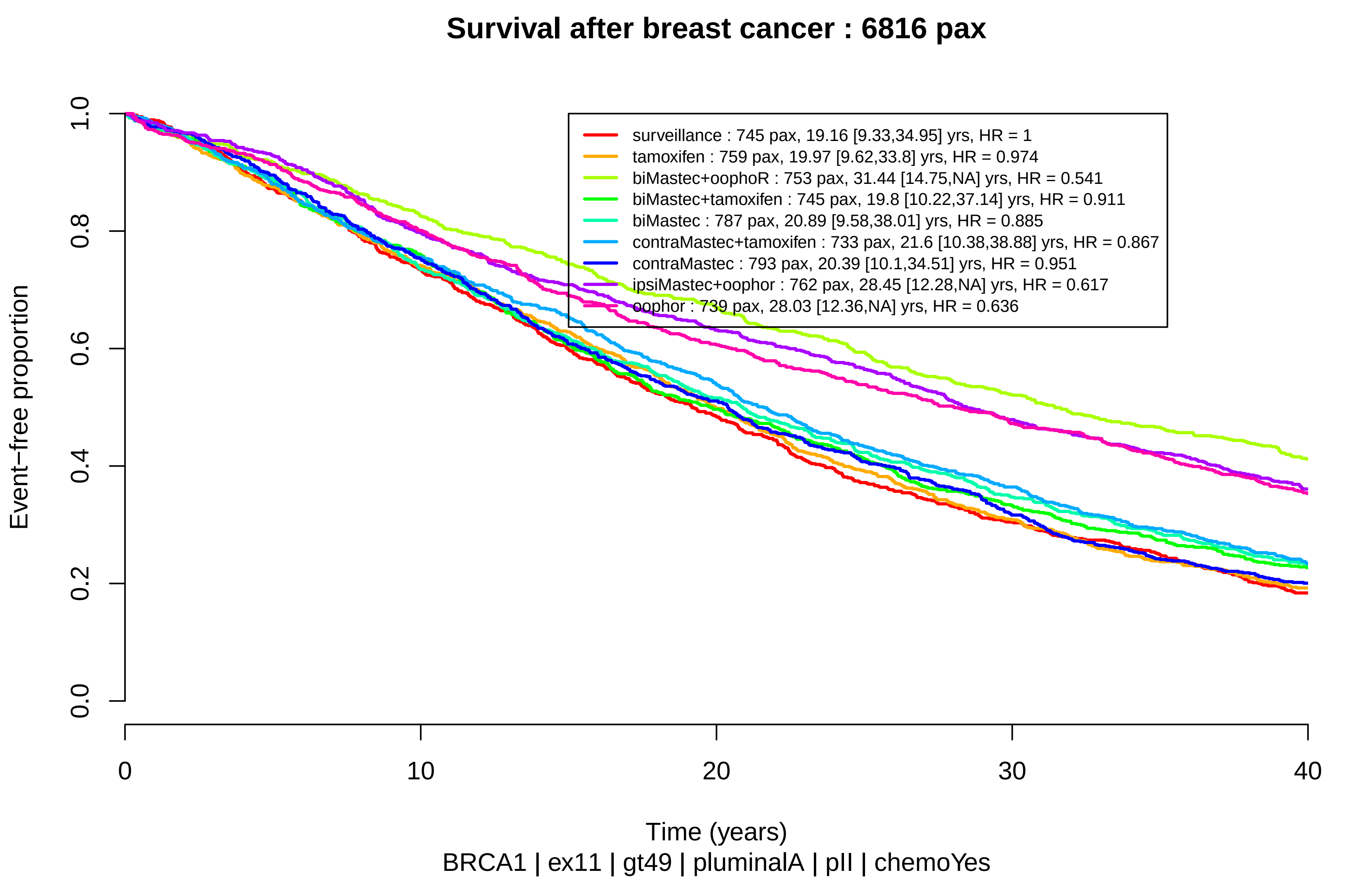
Survival after breast cancer : 6816 pax
surveillance : 745 pax, 19.16 [9.33,34.95] yrs, HR = 1
tamoxifen : 759 pax, 19.97 [9.62,33.8] yrs, HR = 0.974
biMastec+oophoR : 753 pax, 31.44 [14.75,NA] yrs, HR = 0.541
biMastec+tamoxifen : 745 pax, 19.8 [10.22,37.14] yrs, HR = 0.911
biMastec : 787 pax, 20.89 [9.58,38.01] yrs, HR = 0.885
contraMastec+tamoxifen : 733 pax, 21.6 [10.38,38.88] yrs, HR = 0.867
contraMastec : 793 pax, 20.39 [10.1,34.51] yrs, HR = 0.951
ipsiMastec+oophor : 762 pax, 28.45 [12.28,NA] yrs, HR = 0.617
oophor : 739 pax, 28.03 [12.36,NA] yrs, HR = 0.636
Event−free proportion
0.0
0.2
0.4
0.6
0.8
1.0
0
10
20
30
40
Time (years)
BRCA1 | ex11 | gt49 | pluminalA | pII | chemoYes

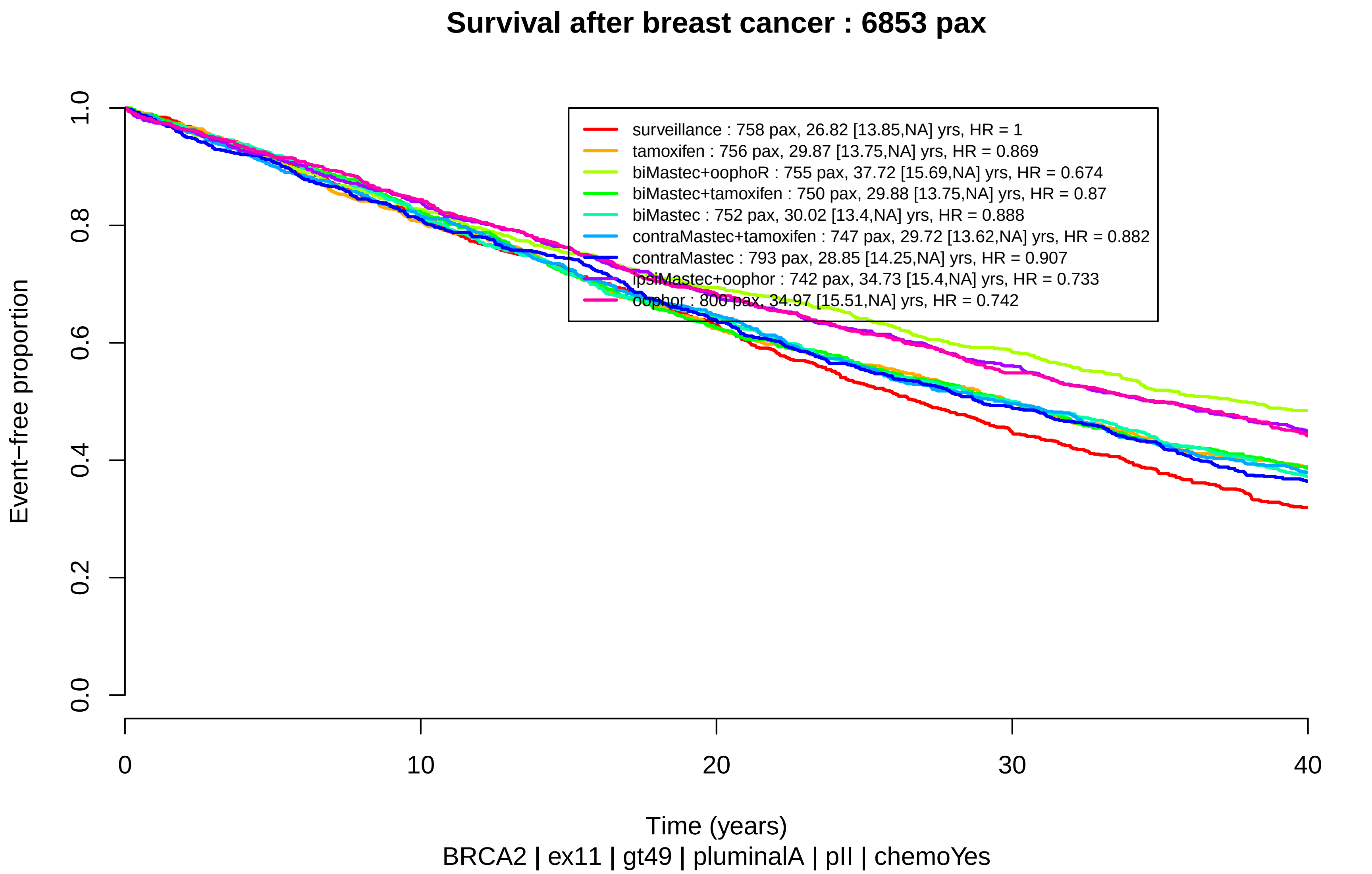
Survival after breast cancer : 6853 pax
Event−free proportion
0.0
0.2
0.4
0.6
0.8
1.0
0
10
20
30
40
Time (years)
BRCA2 | ex11 | gt49 | pluminalA | pII | chemoYes
surveillance : 758 pax, 26.82 [13.85,NA] yrs, HR = 1
tamoxifen : 756 pax, 29.87 [13.75,NA] yrs, HR = 0.869
biMastec+oophoR : 755 pax, 37.72 [15.69,NA] yrs, HR = 0.674
biMastec+tamoxifen : 750 pax, 29.88 [13.75,NA] yrs, HR = 0.87
biMastec : 752 pax, 30.02 [13.4,NA] yrs, HR = 0.888
contraMastec+tamoxifen : 747 pax, 29.72 [13.62,NA] yrs, HR = 0.882
contraMastec : 793 pax, 28.85 [14.25,NA] yrs, HR = 0.907
ipsiMastec+oophor : 742 pax, 34.73 [15.4,NA] yrs, HR = 0.733
oophor : 800 pax, 34.97 [15.51,NA] yrs, HR = 0.742

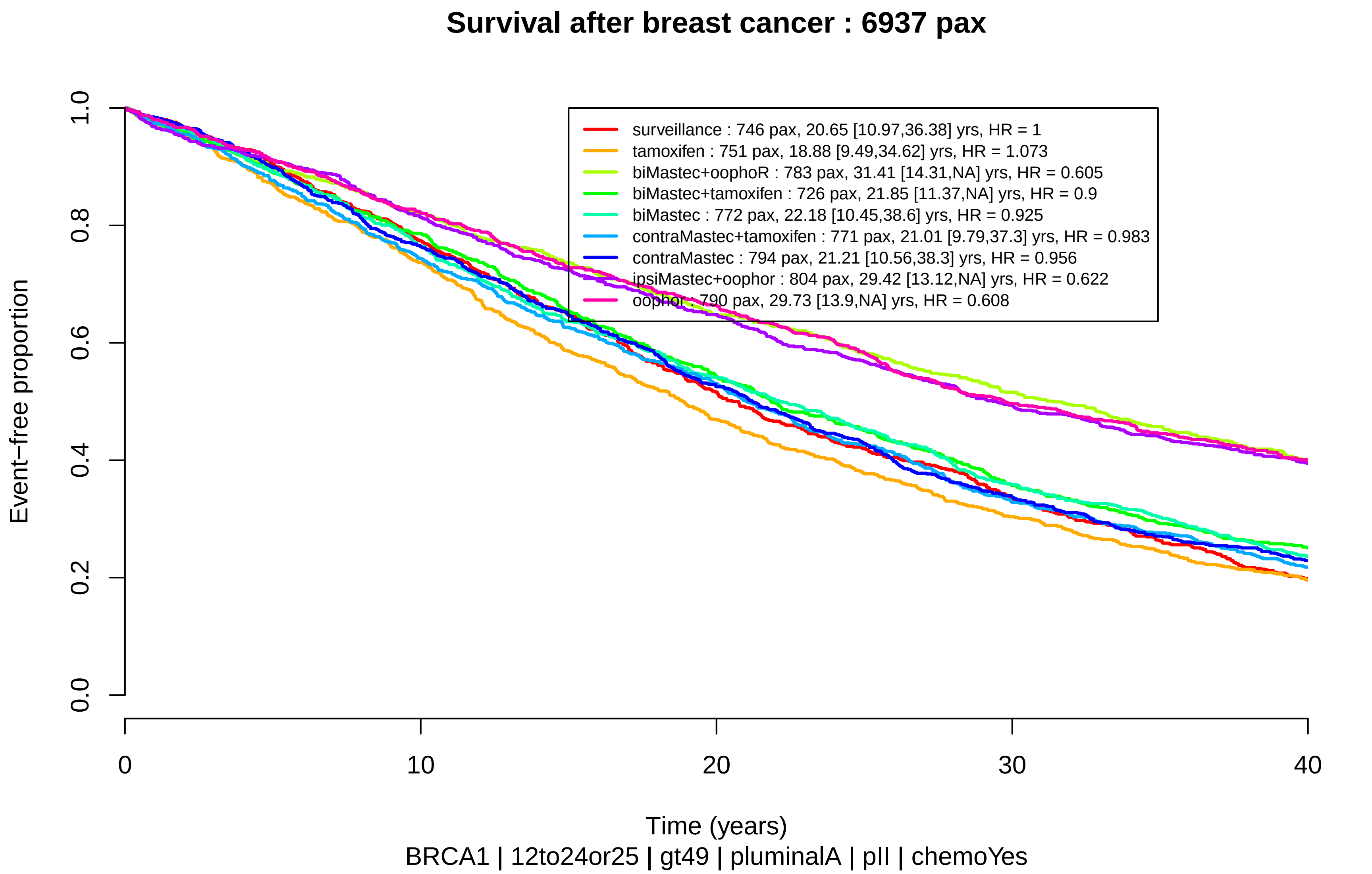

Survival after breast cancer : 6937 pax
surveillance : 746 pax, 20.65 [10.97,36.38] yrs, HR = 1
tamoxifen : 751 pax, 18.88 [9.49,34.62] yrs, HR = 1.073
biMastec+oophoR : 783 pax, 31.41 [14.31,NA] yrs, HR = 0.605
biMastec+tamoxifen : 726 pax, 21.85 [11.37,NA] yrs, HR = 0.9
biMastec : 772 pax, 22.18 [10.45,38.6] yrs, HR = 0.925
contraMastec+tamoxifen : 771 pax, 21.01 [9.79,37.3] yrs, HR = 0.983
contraMastec : 794 pax, 21.21 [10.56,38.3] yrs, HR = 0.956
ipsiMastec+oophor : 804 pax, 29.42 [13.12,NA] yrs, HR = 0.622
oophor : 790 pax, 29.73 [13.9,NA] yrs, HR = 0.608
Event−free proportion
0.0
0.2
0.4
0.6
0.8
1.0
0
10
20
30
40
Time (years)
BRCA1 | 12to24or25 | gt49 | pluminalA | pII | chemoYes

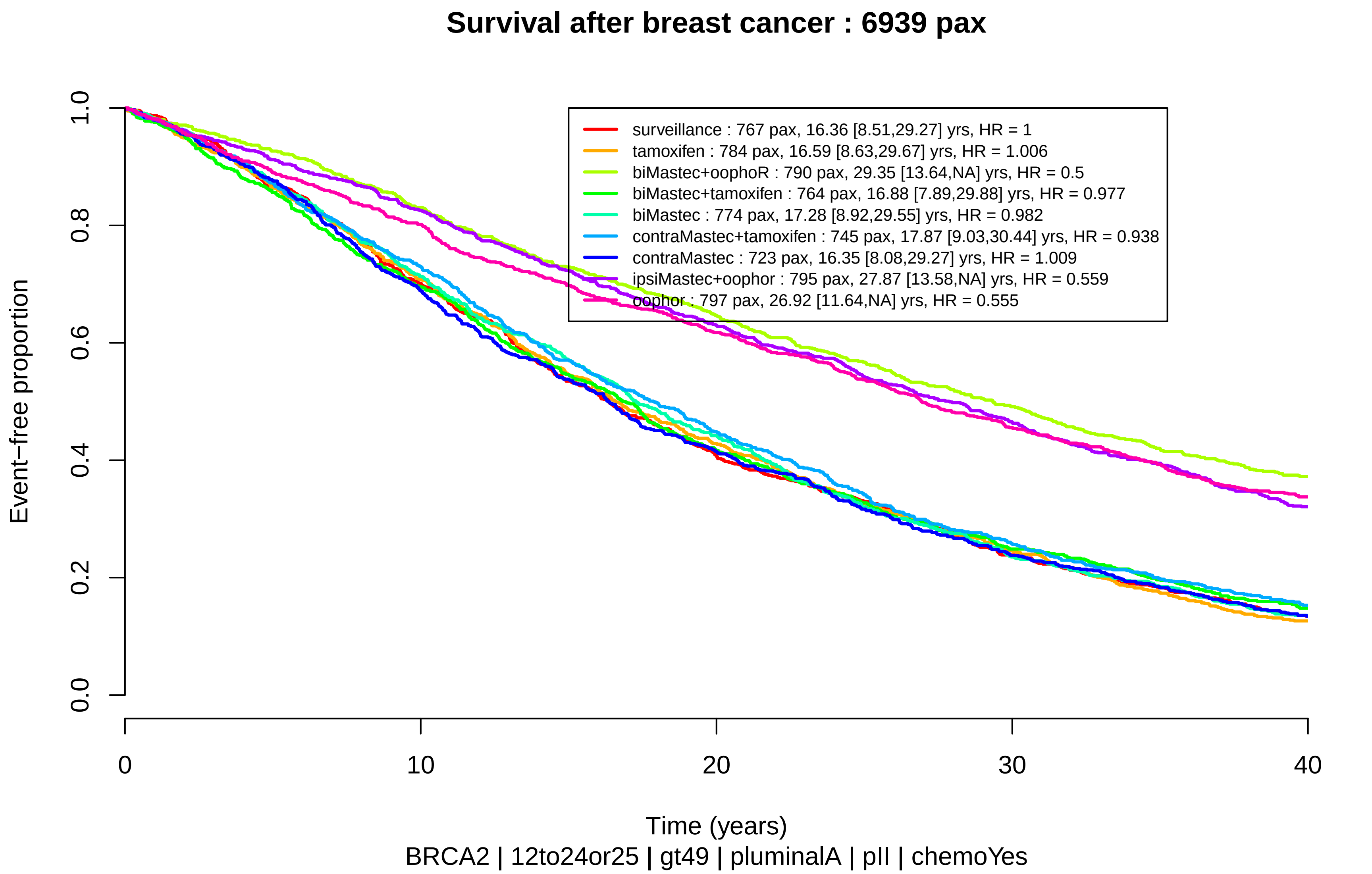

Survival after breast cancer : 6939 pax
surveillance : 767 pax, 16.36 [8.51,29.27] yrs, HR = 1
tamoxifen : 784 pax, 16.59 [8.63,29.67] yrs, HR = 1.006
biMastec+oophoR : 790 pax, 29.35 [13.64,NA] yrs, HR = 0.5
biMastec+tamoxifen : 764 pax, 16.88 [7.89,29.88] yrs, HR = 0.977
biMastec : 774 pax, 17.28 [8.92,29.55] yrs, HR = 0.982
contraMastec+tamoxifen : 745 pax, 17.87 [9.03,30.44] yrs, HR = 0.938
contraMastec : 723 pax, 16.35 [8.08,29.27] yrs, HR = 1.009
ipsiMastec+oophor : 795 pax, 27.87 [13.58,NA] yrs, HR = 0.559
oophor : 797 pax, 26.92 [11.64,NA] yrs, HR = 0.555
Event−free proportion
0.0
0.2
0.4
0.6
0.8
1.0
0
10
20
30
40
Time (years)
BRCA2 | 12to24or25 | gt49 | pluminalA | pII | chemoYes

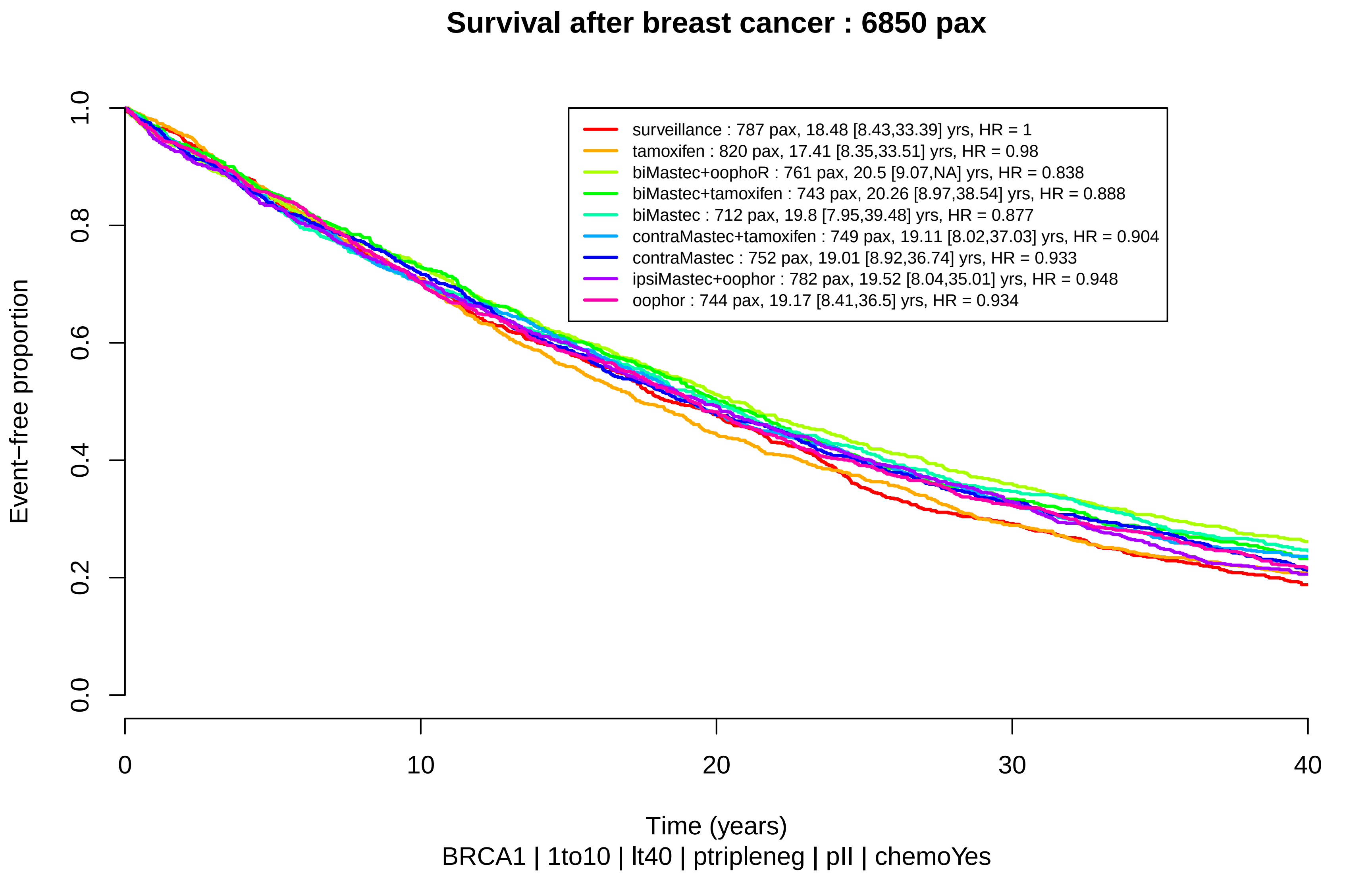

Survival after breast cancer : 6850 pax
surveillance : 787 pax, 18.48 [8.43,33.39] yrs, HR = 1
tamoxifen : 820 pax, 17.41 [8.35,33.51] yrs, HR = 0.98
biMastec+oophoR : 761 pax, 20.5 [9.07,NA] yrs, HR = 0.838
biMastec+tamoxifen : 743 pax, 20.26 [8.97,38.54] yrs, HR = 0.888
biMastec : 712 pax, 19.8 [7.95,39.48] yrs, HR = 0.877
contraMastec+tamoxifen : 749 pax, 19.11 [8.02,37.03] yrs, HR = 0.904
contraMastec : 752 pax, 19.01 [8.92,36.74] yrs, HR = 0.933
ipsiMastec+oophor : 782 pax, 19.52 [8.04,35.01] yrs, HR = 0.948
oophor : 744 pax, 19.17 [8.41,36.5] yrs, HR = 0.934
Event−free proportion
0.0
0.2
0.4
0.6
0.8
1.0
0
10
20
30
40
Time (years)
BRCA1 | 1to10 | lt40 | ptripleneg | pII | chemoYes

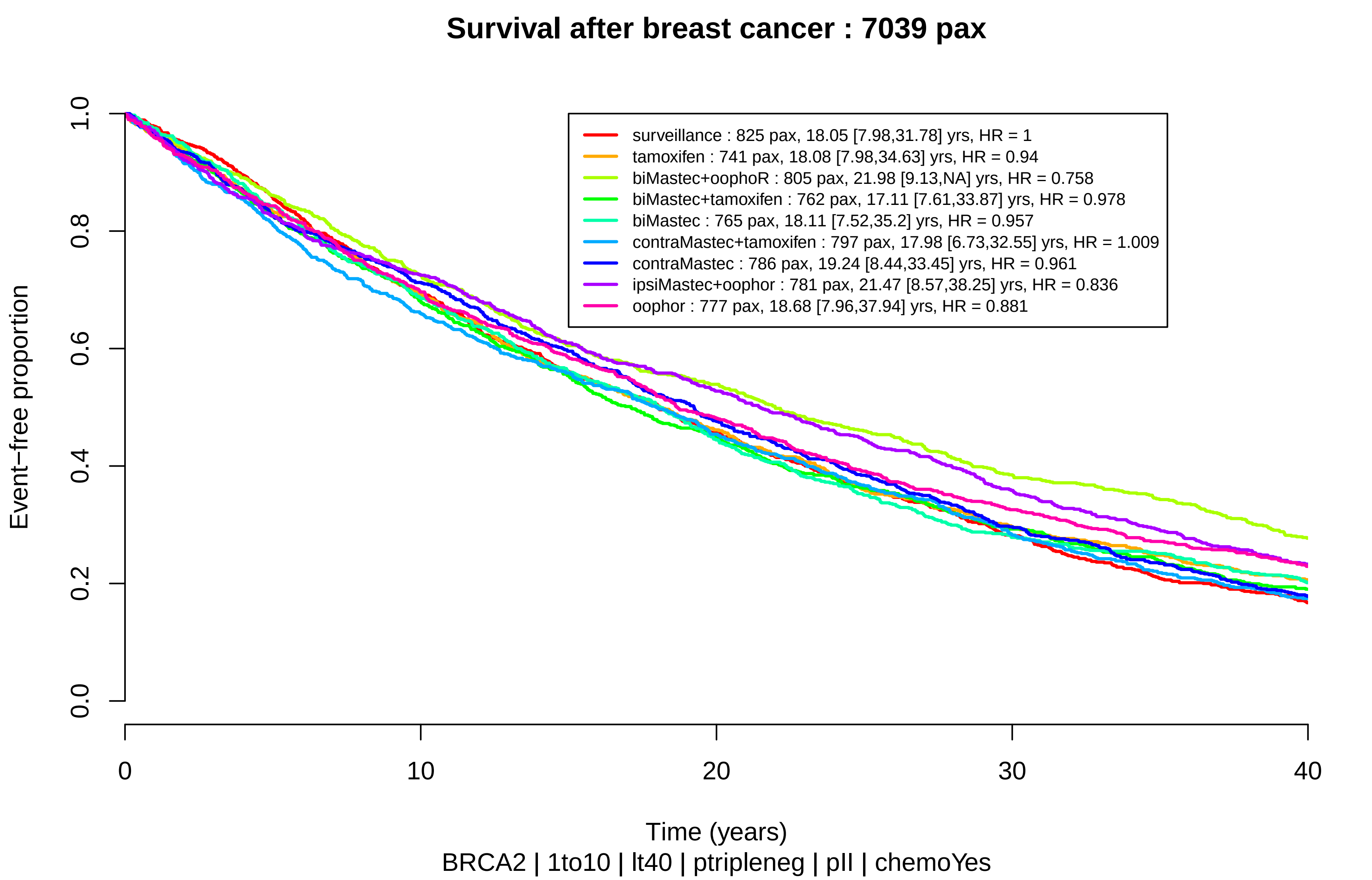

Survival after breast cancer : 7039 pax
surveillance : 825 pax, 18.05 [7.98,31.78] yrs, HR = 1
tamoxifen : 741 pax, 18.08 [7.98,34.63] yrs, HR = 0.94
biMastec+oophoR : 805 pax, 21.98 [9.13,NA] yrs, HR = 0.758
biMastec+tamoxifen : 762 pax, 17.11 [7.61,33.87] yrs, HR = 0.978
biMastec : 765 pax, 18.11 [7.52,35.2] yrs, HR = 0.957
contraMastec+tamoxifen : 797 pax, 17.98 [6.73,32.55] yrs, HR = 1.009
contraMastec : 786 pax, 19.24 [8.44,33.45] yrs, HR = 0.961
ipsiMastec+oophor : 781 pax, 21.47 [8.57,38.25] yrs, HR = 0.836
oophor : 777 pax, 18.68 [7.96,37.94] yrs, HR = 0.881
Event−free proportion
0.0
0.2
0.4
0.6
0.8
1.0
0
10
20
30
40
Time (years)
BRCA2 | 1to10 | lt40 | ptripleneg | pII | chemoYes

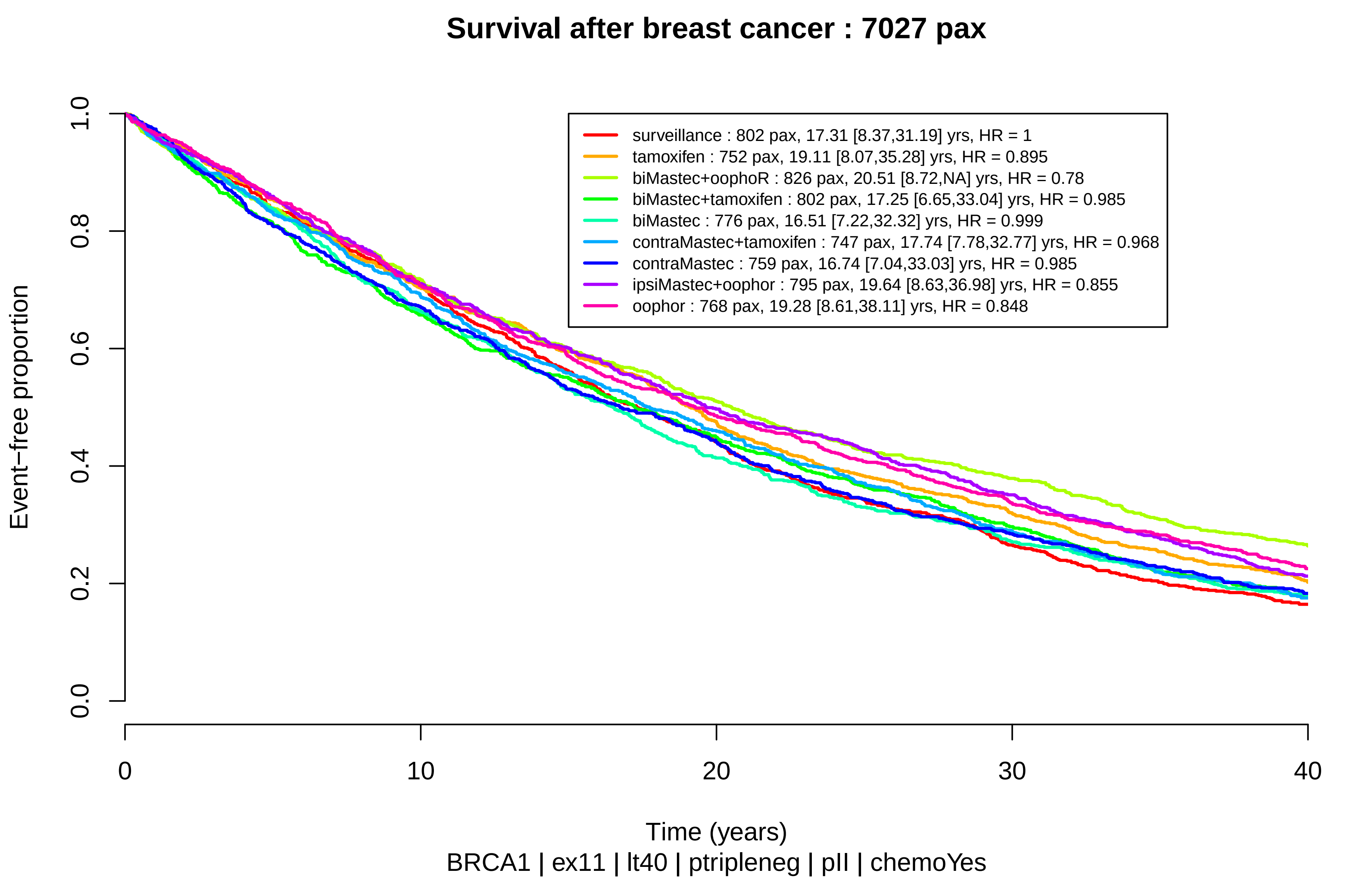
Survival after breast cancer : 7027 pax
Event−free proportion
0.0
0.2
0.4
0.6
0.8
1.0
0
10
20
30
40
Time (years)
BRCA1 | ex11 | lt40 | ptripleneg | pII | chemoYes
surveillance : 802 pax, 17.31 [8.37,31.19] yrs, HR = 1
tamoxifen : 752 pax, 19.11 [8.07,35.28] yrs, HR = 0.895
biMastec+oophoR : 826 pax, 20.51 [8.72,NA] yrs, HR = 0.78
biMastec+tamoxifen : 802 pax, 17.25 [6.65,33.04] yrs, HR = 0.985
biMastec : 776 pax, 16.51 [7.22,32.32] yrs, HR = 0.999
contraMastec+tamoxifen : 747 pax, 17.74 [7.78,32.77] yrs, HR = 0.968
contraMastec : 759 pax, 16.74 [7.04,33.03] yrs, HR = 0.985
ipsiMastec+oophor : 795 pax, 19.64 [8.63,36.98] yrs, HR = 0.855
oophor : 768 pax, 19.28 [8.61,38.11] yrs, HR = 0.848

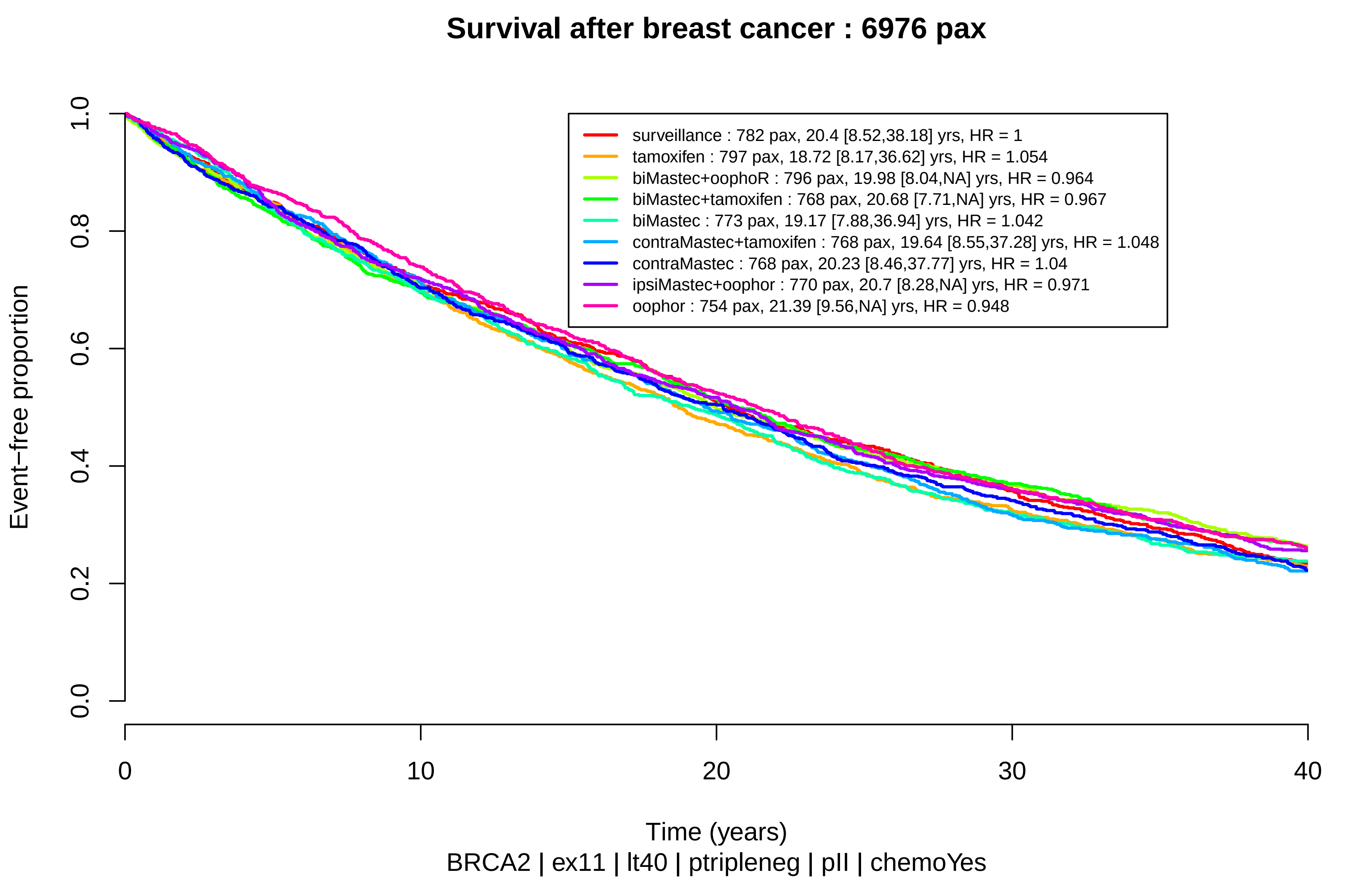

Survival after breast cancer : 6976 pax
surveillance : 782 pax, 20.4 [8.52,38.18] yrs, HR = 1
tamoxifen : 797 pax, 18.72 [8.17,36.62] yrs, HR = 1.054
biMastec+oophoR : 796 pax, 19.98 [8.04,NA] yrs, HR = 0.964
biMastec+tamoxifen : 768 pax, 20.68 [7.71,NA] yrs, HR = 0.967
biMastec : 773 pax, 19.17 [7.88,36.94] yrs, HR = 1.042
contraMastec+tamoxifen : 768 pax, 19.64 [8.55,37.28] yrs, HR = 1.048
contraMastec : 768 pax, 20.23 [8.46,37.77] yrs, HR = 1.04
ipsiMastec+oophor : 770 pax, 20.7 [8.28,NA] yrs, HR = 0.971
oophor : 754 pax, 21.39 [9.56,NA] yrs, HR = 0.948
Event−free proportion
0.0
0.2
0.4
0.6
0.8
1.0
0
10
20
30
40
Time (years)
BRCA2 | ex11 | lt40 | ptripleneg | pII | chemoYes

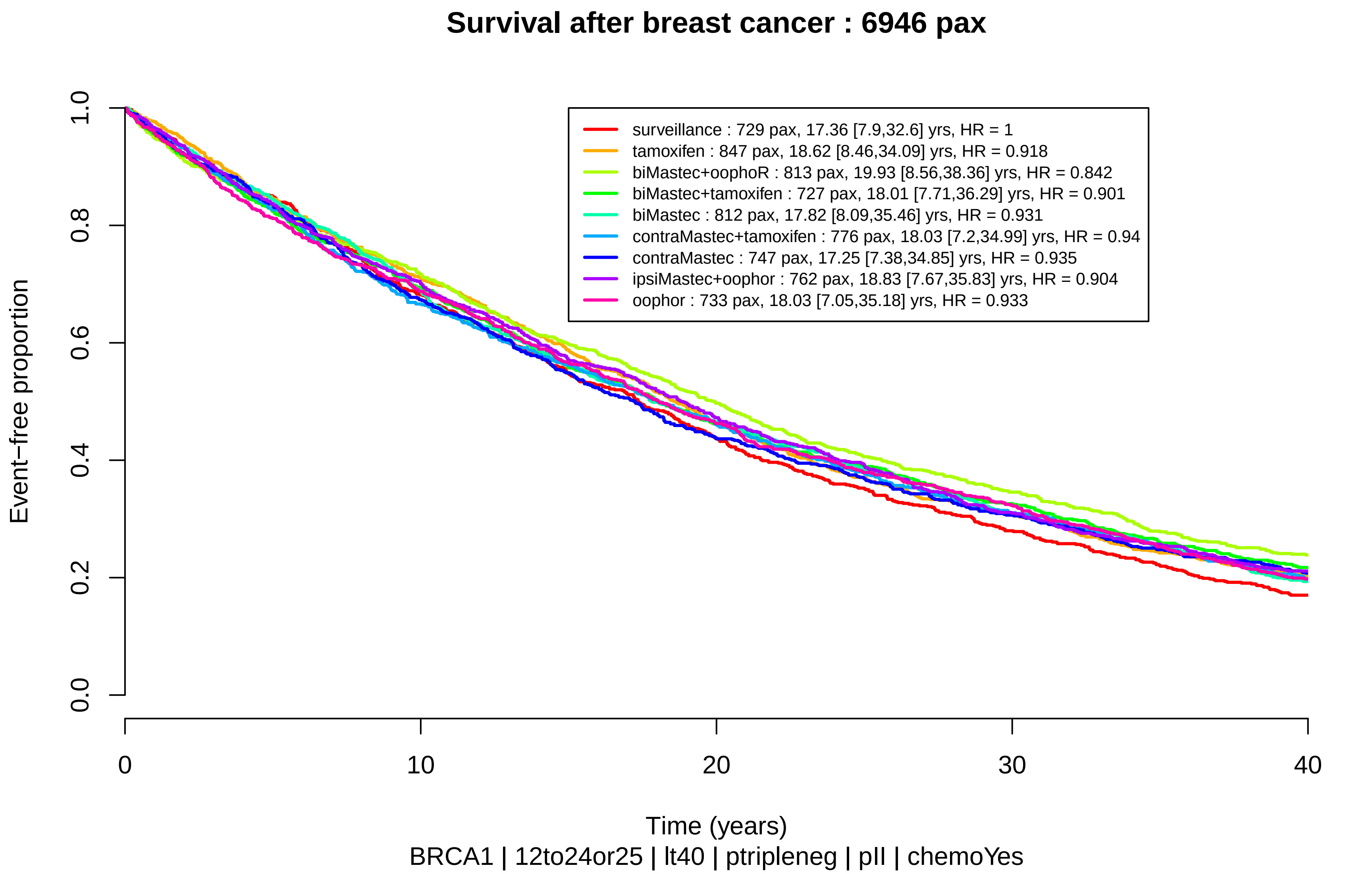
Survival after breast cancer : 6946 pax
surveillance : 729 pax, 17.36 [7.9,32.6] yrs, HR = 1
tamoxifen : 847 pax, 18.62 [8.46,34.09] yrs, HR = 0.918
biMastec+oophoR : 813 pax, 19.93 [8.56,38.36] yrs, HR = 0.842
biMastec+tamoxifen : 727 pax, 18.01 [7.71,36.29] yrs, HR = 0.901
biMastec : 812 pax, 17.82 [8.09,35.46] yrs, HR = 0.931
contraMastec+tamoxifen : 776 pax, 18.03 [7.2,34.99] yrs, HR = 0.94
contraMastec : 747 pax, 17.25 [7.38,34.85] yrs, HR = 0.935
ipsiMastec+oophor : 762 pax, 18.83 [7.67,35.83] yrs, HR = 0.904
oophor : 733 pax, 18.03 [7.05,35.18] yrs, HR = 0.933
Event−free proportion
0.0
0.2
0.4
0.6
0.8
1.0
0
10
20
30
40
Time (years)
BRCA1 | 12to24or25 | lt40 | ptripleneg | pII | chemoYes

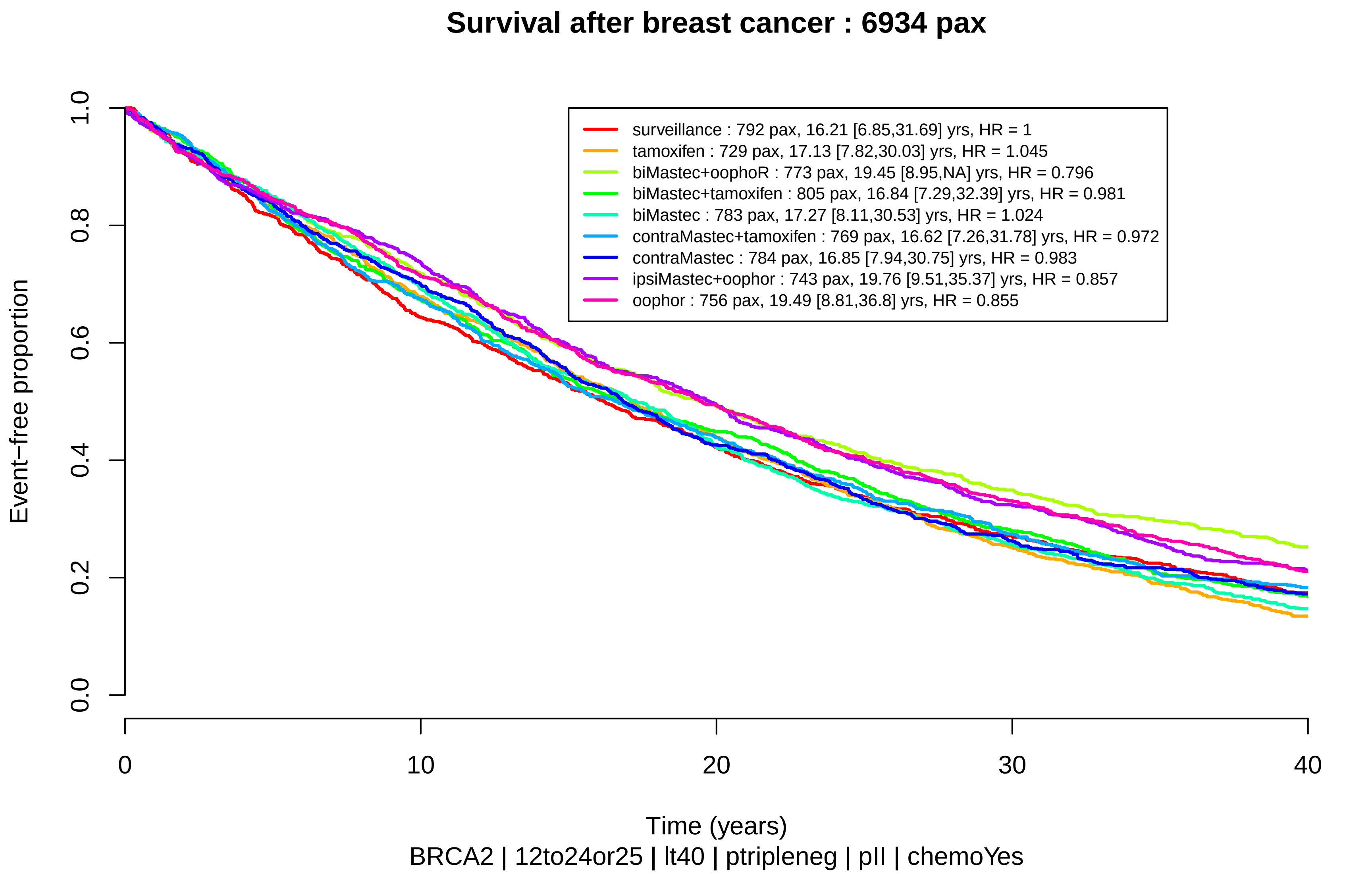
Survival after breast cancer : 6934 pax
surveillance : 792 pax, 16.21 [6.85,31.69] yrs, HR = 1
tamoxifen : 729 pax, 17.13 [7.82,30.03] yrs, HR = 1.045
biMastec+oophoR : 773 pax, 19.45 [8.95,NA] yrs, HR = 0.796
biMastec+tamoxifen : 805 pax, 16.84 [7.29,32.39] yrs, HR = 0.981
biMastec : 783 pax, 17.27 [8.11,30.53] yrs, HR = 1.024
contraMastec+tamoxifen : 769 pax, 16.62 [7.26,31.78] yrs, HR = 0.972
contraMastec : 784 pax, 16.85 [7.94,30.75] yrs, HR = 0.983
ipsiMastec+oophor : 743 pax, 19.76 [9.51,35.37] yrs, HR = 0.857
oophor : 756 pax, 19.49 [8.81,36.8] yrs, HR = 0.855
Event−free proportion
0.0
0.2
0.4
0.6
0.8
1.0
0
10
20
30
40
Time (years)
BRCA2 | 12to24or25 | lt40 | ptripleneg | pII | chemoYes

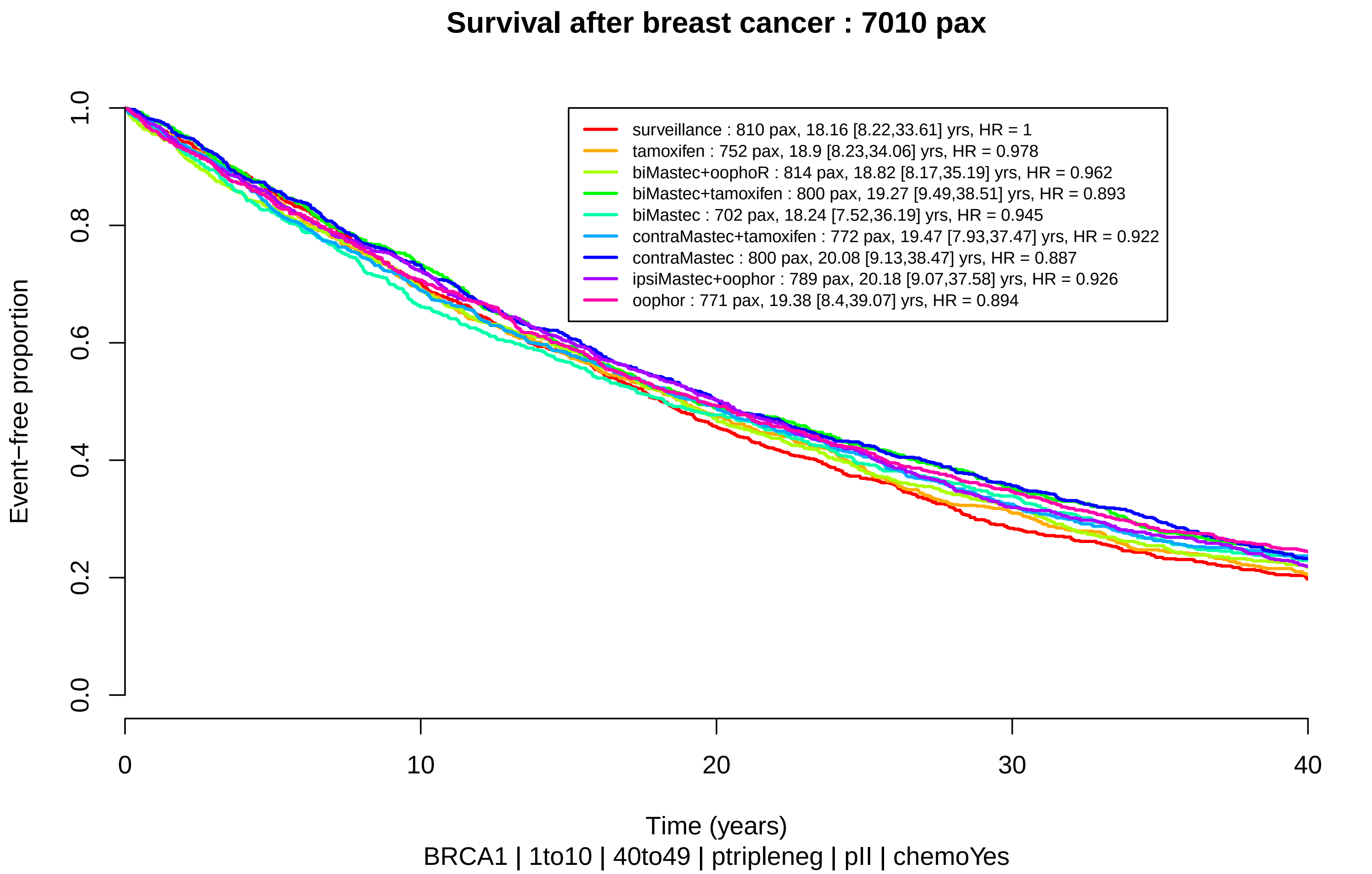
Survival after breast cancer : 7010 pax
surveillance : 810 pax, 18.16 [8.22,33.61] yrs, HR = 1
tamoxifen : 752 pax, 18.9 [8.23,34.06] yrs, HR = 0.978
biMastec+oophoR : 814 pax, 18.82 [8.17,35.19] yrs, HR = 0.962
biMastec+tamoxifen : 800 pax, 19.27 [9.49,38.51] yrs, HR = 0.893
biMastec : 702 pax, 18.24 [7.52,36.19] yrs, HR = 0.945
contraMastec+tamoxifen : 772 pax, 19.47 [7.93,37.47] yrs, HR = 0.922
contraMastec : 800 pax, 20.08 [9.13,38.47] yrs, HR = 0.887
ipsiMastec+oophor : 789 pax, 20.18 [9.07,37.58] yrs, HR = 0.926
oophor : 771 pax, 19.38 [8.4,39.07] yrs, HR = 0.894
Event−free proportion
0.0
0.2
0.4
0.6
0.8
1.0
0
10
20
30
40
Time (years)
BRCA1 | 1to10 | 40to49 | ptripleneg | pII | chemoYes

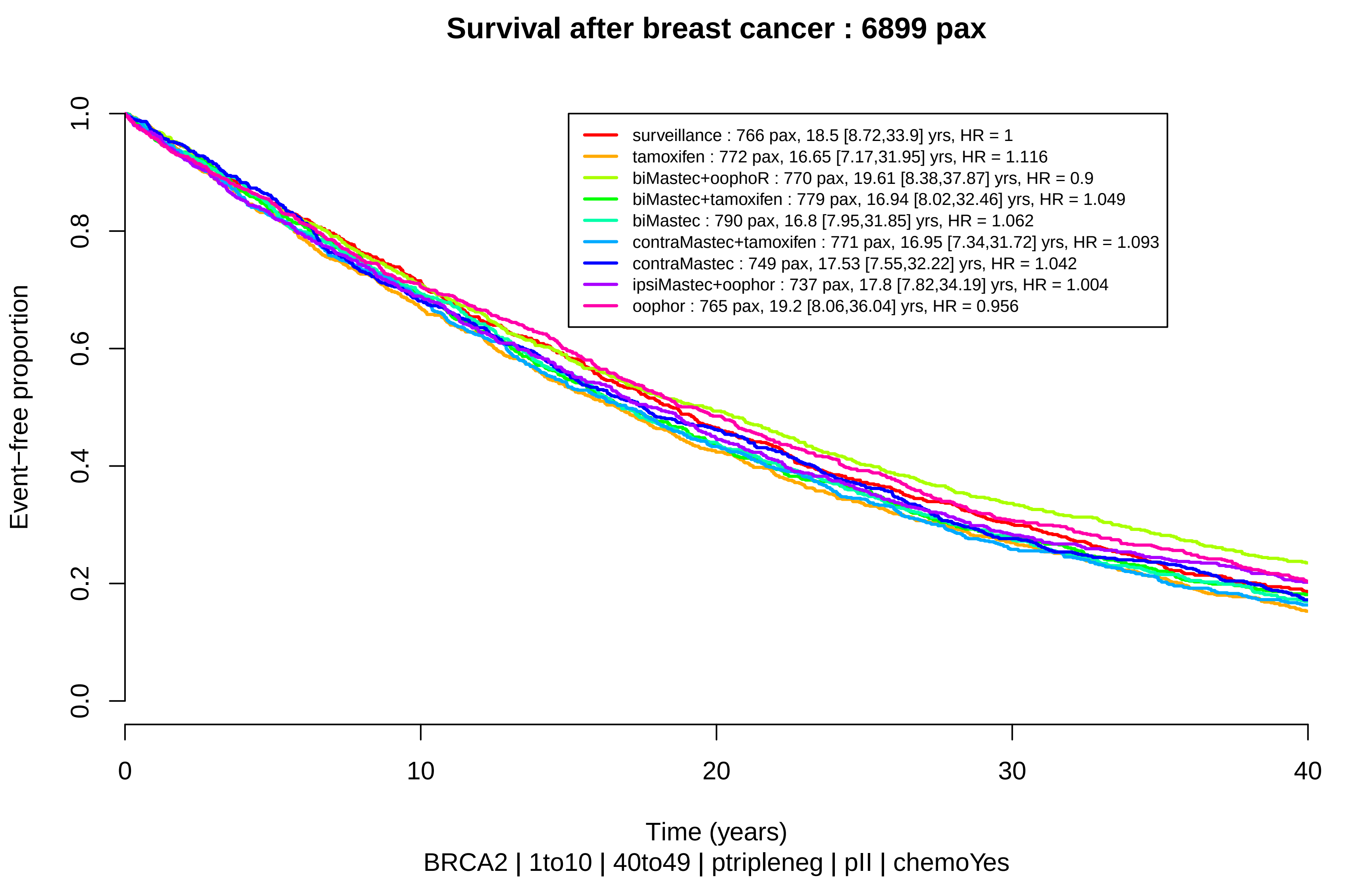

Survival after breast cancer : 6899 pax
surveillance : 766 pax, 18.5 [8.72,33.9] yrs, HR = 1
tamoxifen : 772 pax, 16.65 [7.17,31.95] yrs, HR = 1.116
biMastec+oophoR : 770 pax, 19.61 [8.38,37.87] yrs, HR = 0.9
biMastec+tamoxifen : 779 pax, 16.94 [8.02,32.46] yrs, HR = 1.049
biMastec : 790 pax, 16.8 [7.95,31.85] yrs, HR = 1.062
contraMastec+tamoxifen : 771 pax, 16.95 [7.34,31.72] yrs, HR = 1.093
contraMastec : 749 pax, 17.53 [7.55,32.22] yrs, HR = 1.042
ipsiMastec+oophor : 737 pax, 17.8 [7.82,34.19] yrs, HR = 1.004
oophor : 765 pax, 19.2 [8.06,36.04] yrs, HR = 0.956
Event−free proportion
0.0
0.2
0.4
0.6
0.8
1.0
0
10
20
30
40
Time (years)
BRCA2 | 1to10 | 40to49 | ptripleneg | pII | chemoYes

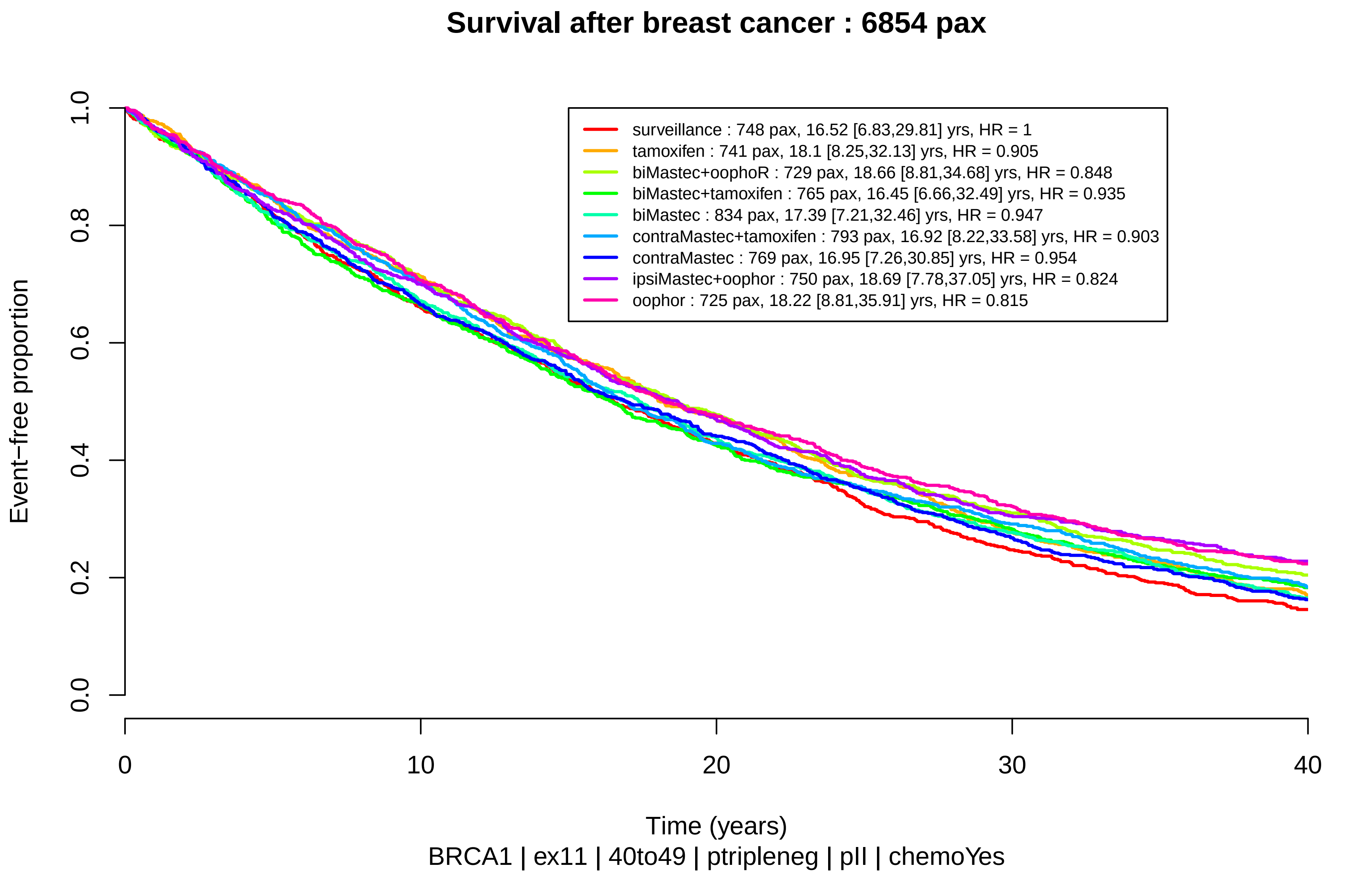
Survival after breast cancer : 6854 pax
Event−free proportion
0.0
0.2
0.4
0.6
0.8
1.0
0
10
20
30
40
Time (years)
BRCA1 | ex11 | 40to49 | ptripleneg | pII | chemoYes
surveillance : 748 pax, 16.52 [6.83,29.81] yrs, HR = 1
tamoxifen : 741 pax, 18.1 [8.25,32.13] yrs, HR = 0.905
biMastec+oophoR : 729 pax, 18.66 [8.81,34.68] yrs, HR = 0.848
biMastec+tamoxifen : 765 pax, 16.45 [6.66,32.49] yrs, HR = 0.935
biMastec : 834 pax, 17.39 [7.21,32.46] yrs, HR = 0.947
contraMastec+tamoxifen : 793 pax, 16.92 [8.22,33.58] yrs, HR = 0.903
contraMastec : 769 pax, 16.95 [7.26,30.85] yrs, HR = 0.954
ipsiMastec+oophor : 750 pax, 18.69 [7.78,37.05] yrs, HR = 0.824
oophor : 725 pax, 18.22 [8.81,35.91] yrs, HR = 0.815

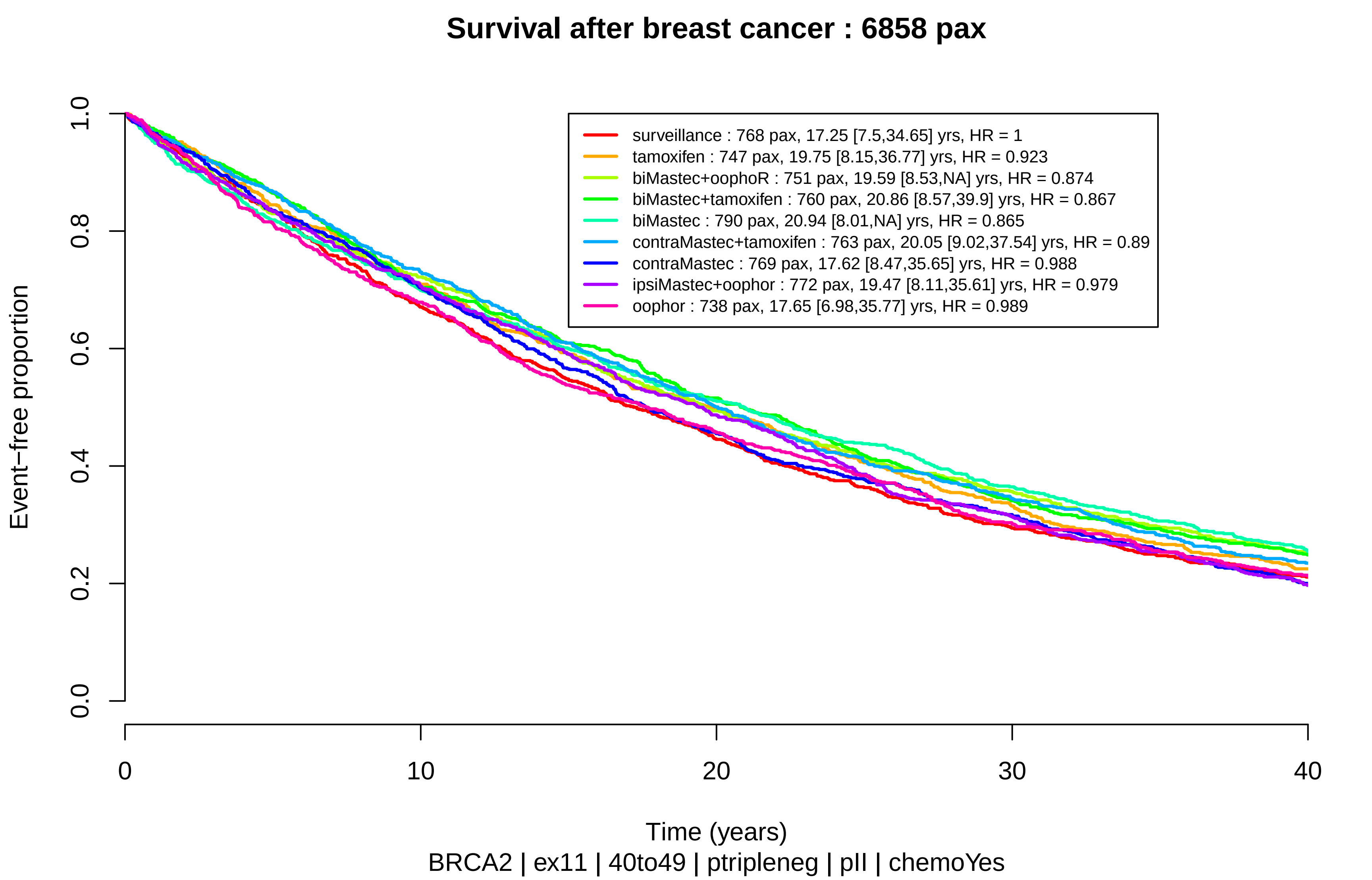
Survival after breast cancer : 6858 pax
surveillance : 768 pax, 17.25 [7.5,34.65] yrs, HR = 1
tamoxifen : 747 pax, 19.75 [8.15,36.77] yrs, HR = 0.923
biMastec+oophoR : 751 pax, 19.59 [8.53,NA] yrs, HR = 0.874
biMastec+tamoxifen : 760 pax, 20.86 [8.57,39.9] yrs, HR = 0.867
biMastec : 790 pax, 20.94 [8.01,NA] yrs, HR = 0.865
contraMastec+tamoxifen : 763 pax, 20.05 [9.02,37.54] yrs, HR = 0.89
contraMastec : 769 pax, 17.62 [8.47,35.65] yrs, HR = 0.988
ipsiMastec+oophor : 772 pax, 19.47 [8.11,35.61] yrs, HR = 0.979
oophor : 738 pax, 17.65 [6.98,35.77] yrs, HR = 0.989
Event−free proportion
0.0
0.2
0.4
0.6
0.8
1.0
0
10
20
30
40
Time (years)
BRCA2 | ex11 | 40to49 | ptripleneg | pII | chemoYes

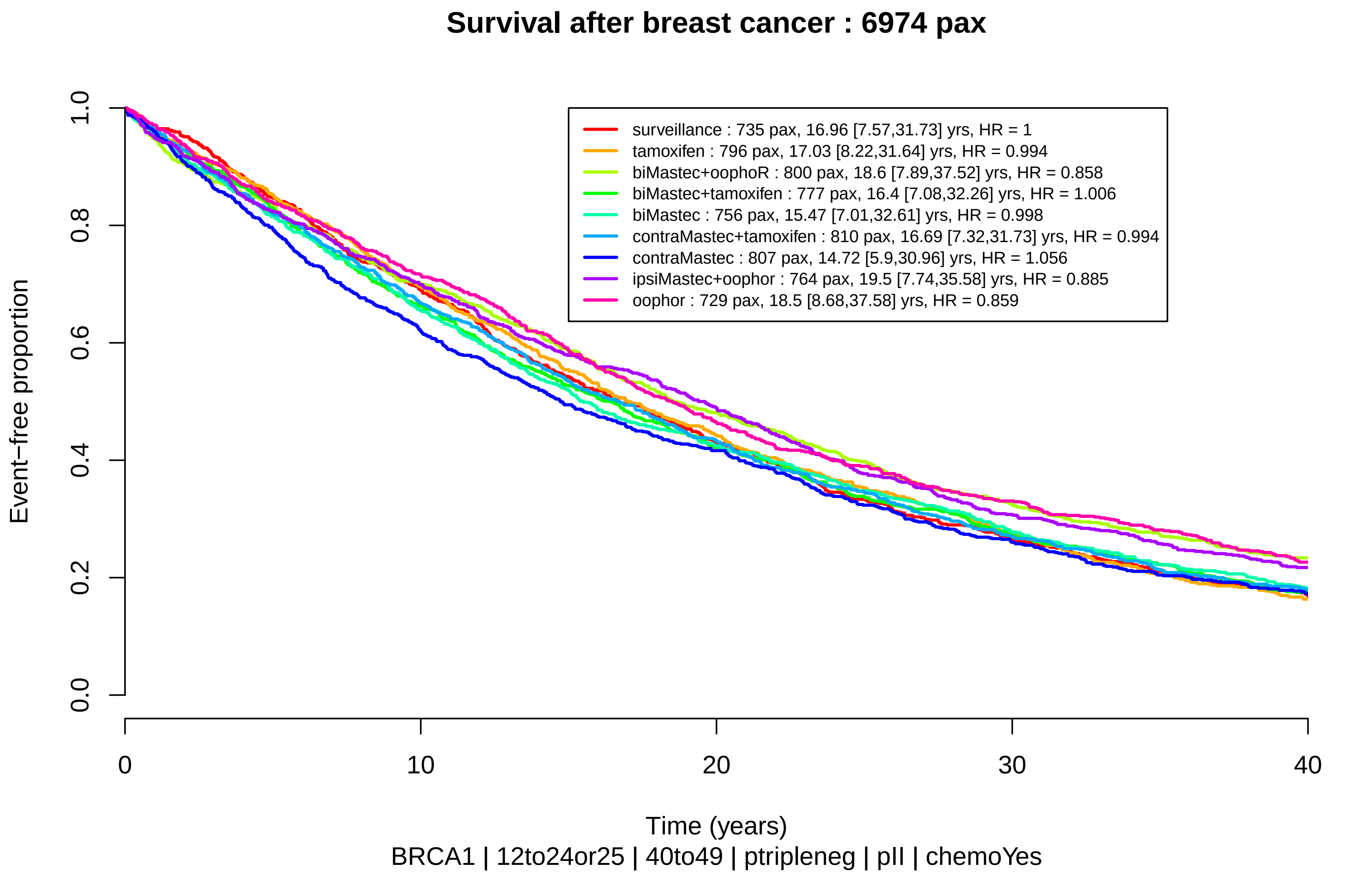

Survival after breast cancer : 6974 pax
surveillance : 735 pax, 16.96 [7.57,31.73] yrs, HR = 1
tamoxifen : 796 pax, 17.03 [8.22,31.64] yrs, HR = 0.994
biMastec+oophoR : 800 pax, 18.6 [7.89,37.52] yrs, HR = 0.858
biMastec+tamoxifen : 777 pax, 16.4 [7.08,32.26] yrs, HR = 1.006
biMastec : 756 pax, 15.47 [7.01,32.61] yrs, HR = 0.998
contraMastec+tamoxifen : 810 pax, 16.69 [7.32,31.73] yrs, HR = 0.994
contraMastec : 807 pax, 14.72 [5.9,30.96] yrs, HR = 1.056
ipsiMastec+oophor : 764 pax, 19.5 [7.74,35.58] yrs, HR = 0.885
oophor : 729 pax, 18.5 [8.68,37.58] yrs, HR = 0.859
Event−free proportion
0.0
0.2
0.4
0.6
0.8
1.0
0
10
20
30
40
Time (years)
BRCA1 | 12to24or25 | 40to49 | ptripleneg | pII | chemoYes

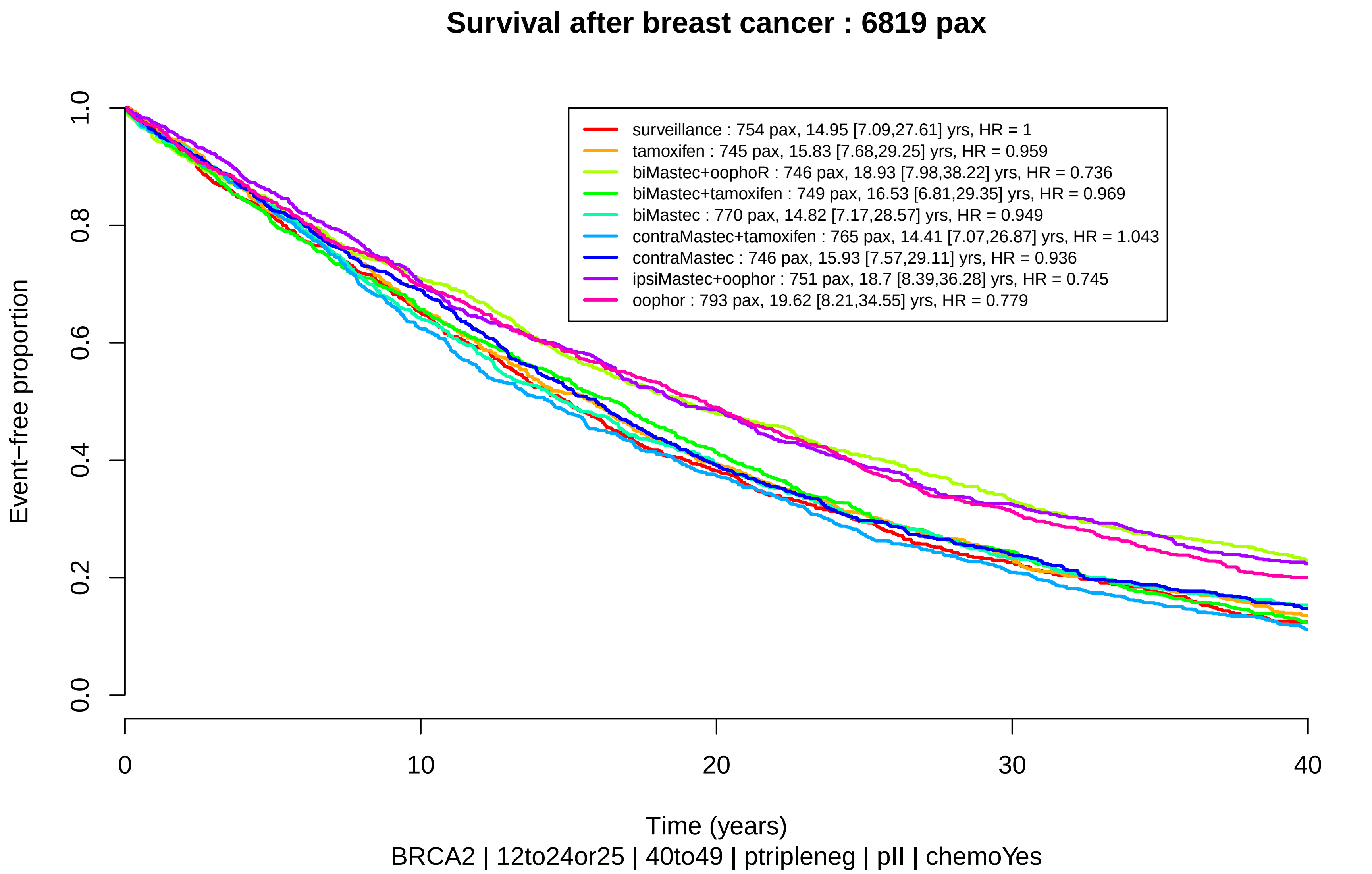

Survival after breast cancer : 6819 pax
surveillance : 754 pax, 14.95 [7.09,27.61] yrs, HR = 1
tamoxifen : 745 pax, 15.83 [7.68,29.25] yrs, HR = 0.959
biMastec+oophoR : 746 pax, 18.93 [7.98,38.22] yrs, HR = 0.736
biMastec+tamoxifen : 749 pax, 16.53 [6.81,29.35] yrs, HR = 0.969
biMastec : 770 pax, 14.82 [7.17,28.57] yrs, HR = 0.949
contraMastec+tamoxifen : 765 pax, 14.41 [7.07,26.87] yrs, HR = 1.043
contraMastec : 746 pax, 15.93 [7.57,29.11] yrs, HR = 0.936
ipsiMastec+oophor : 751 pax, 18.7 [8.39,36.28] yrs, HR = 0.745
oophor : 793 pax, 19.62 [8.21,34.55] yrs, HR = 0.779
Event−free proportion
0.0
0.2
0.4
0.6
0.8
1.0
0
10
20
30
40
Time (years)
BRCA2 | 12to24or25 | 40to49 | ptripleneg | pII | chemoYes

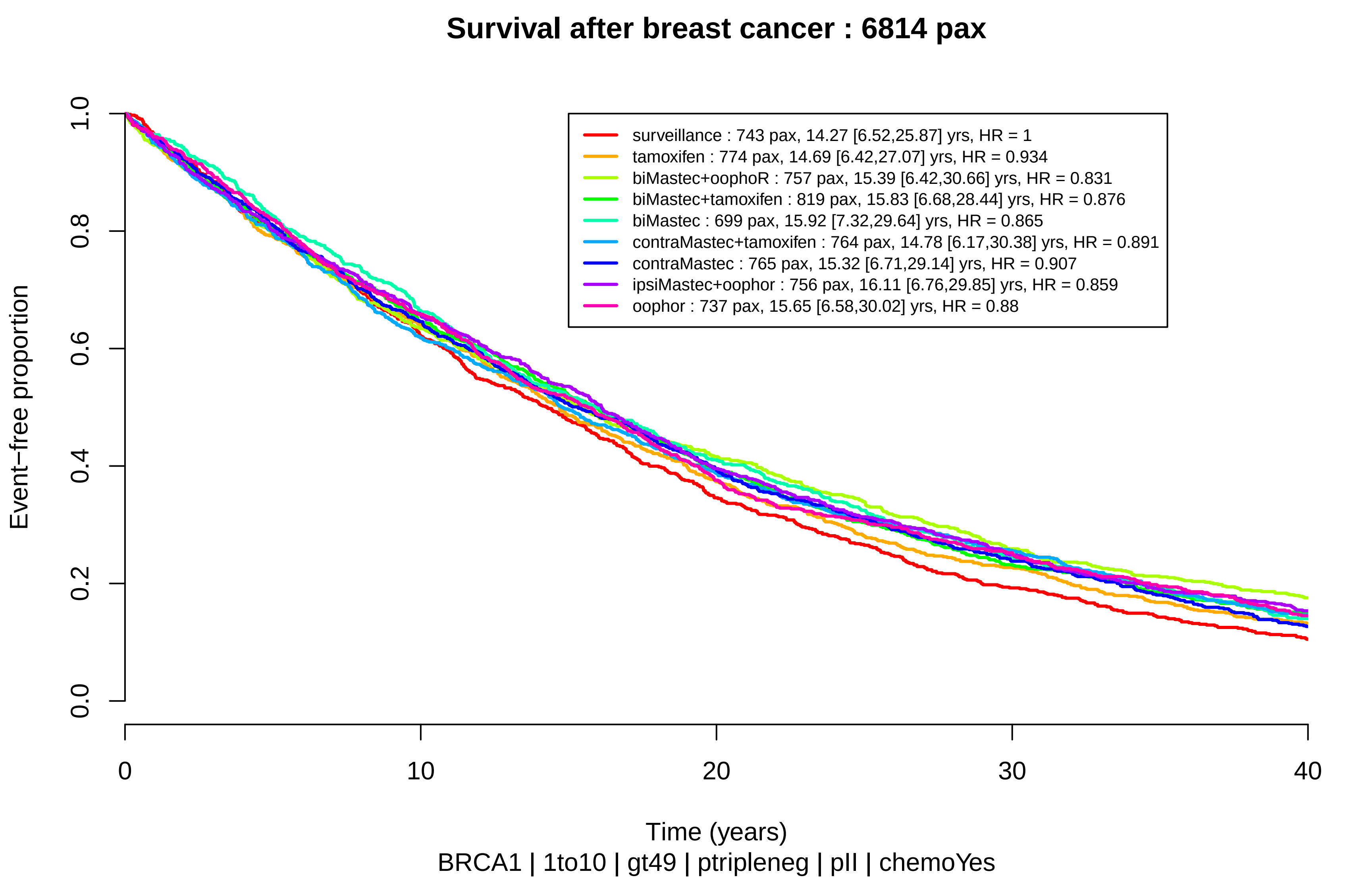

Survival after breast cancer : 6814 pax
surveillance : 743 pax, 14.27 [6.52,25.87] yrs, HR = 1
tamoxifen : 774 pax, 14.69 [6.42,27.07] yrs, HR = 0.934
biMastec+oophoR : 757 pax, 15.39 [6.42,30.66] yrs, HR = 0.831
biMastec+tamoxifen : 819 pax, 15.83 [6.68,28.44] yrs, HR = 0.876
biMastec : 699 pax, 15.92 [7.32,29.64] yrs, HR = 0.865
contraMastec+tamoxifen : 764 pax, 14.78 [6.17,30.38] yrs, HR = 0.891
contraMastec : 765 pax, 15.32 [6.71,29.14] yrs, HR = 0.907
ipsiMastec+oophor : 756 pax, 16.11 [6.76,29.85] yrs, HR = 0.859
oophor : 737 pax, 15.65 [6.58,30.02] yrs, HR = 0.88
Event−free proportion
0.0
0.2
0.4
0.6
0.8
1.0
0
10
20
30
40
Time (years)
BRCA1 | 1to10 | gt49 | ptripleneg | pII | chemoYes

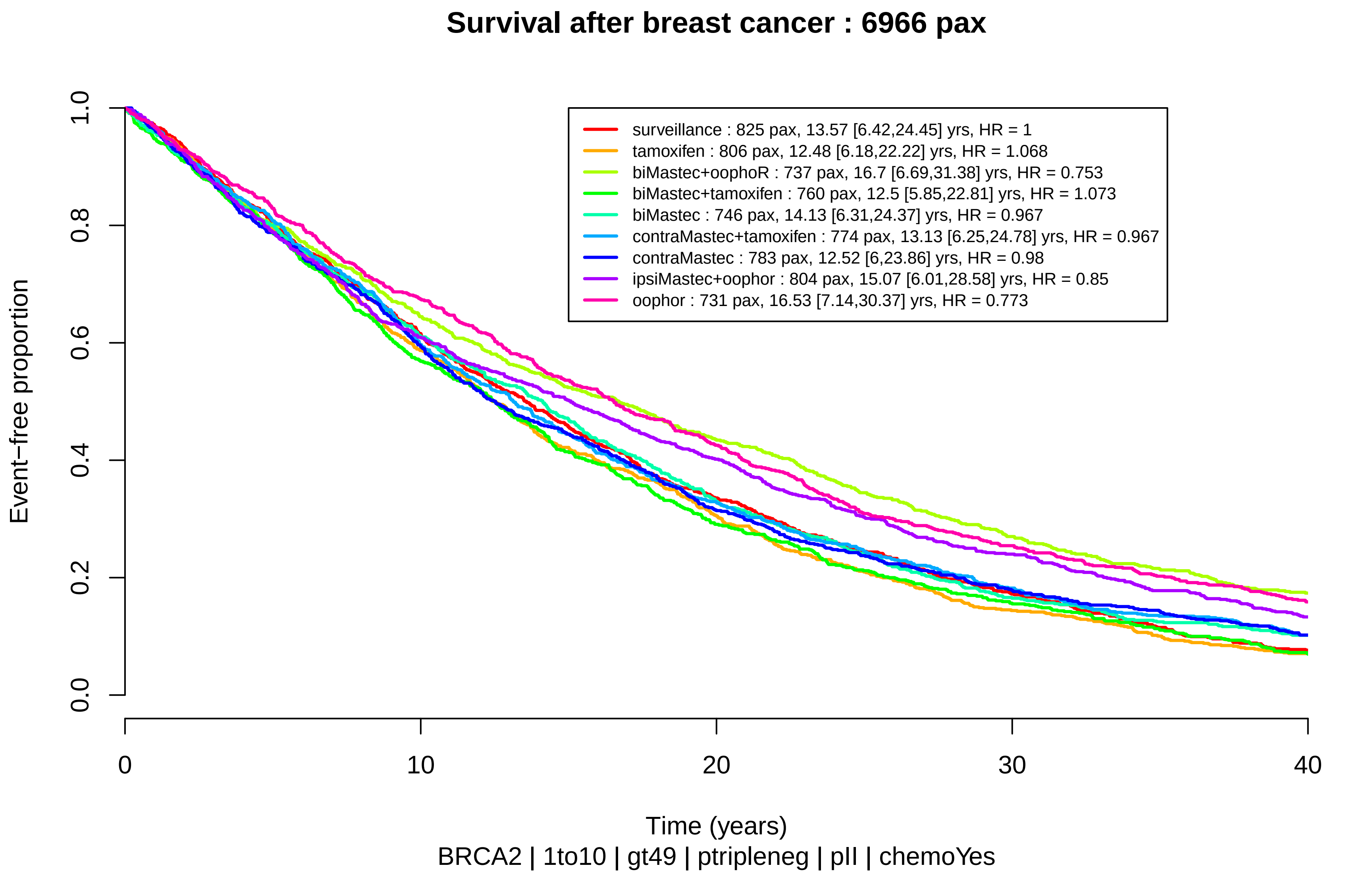
Survival after breast cancer : 6966 pax
surveillance : 825 pax, 13.57 [6.42,24.45] yrs, HR = 1
tamoxifen : 806 pax, 12.48 [6.18,22.22] yrs, HR = 1.068
biMastec+oophoR : 737 pax, 16.7 [6.69,31.38] yrs, HR = 0.753
biMastec+tamoxifen : 760 pax, 12.5 [5.85,22.81] yrs, HR = 1.073
biMastec : 746 pax, 14.13 [6.31,24.37] yrs, HR = 0.967
contraMastec+tamoxifen : 774 pax, 13.13 [6.25,24.78] yrs, HR = 0.967
contraMastec : 783 pax, 12.52 [6,23.86] yrs, HR = 0.98
ipsiMastec+oophor : 804 pax, 15.07 [6.01,28.58] yrs, HR = 0.85
oophor : 731 pax, 16.53 [7.14,30.37] yrs, HR = 0.773
Event−free proportion
0.0
0.2
0.4
0.6
0.8
1.0
0
10
20
30
40
Time (years)
BRCA2 | 1to10 | gt49 | ptripleneg | pII | chemoYes

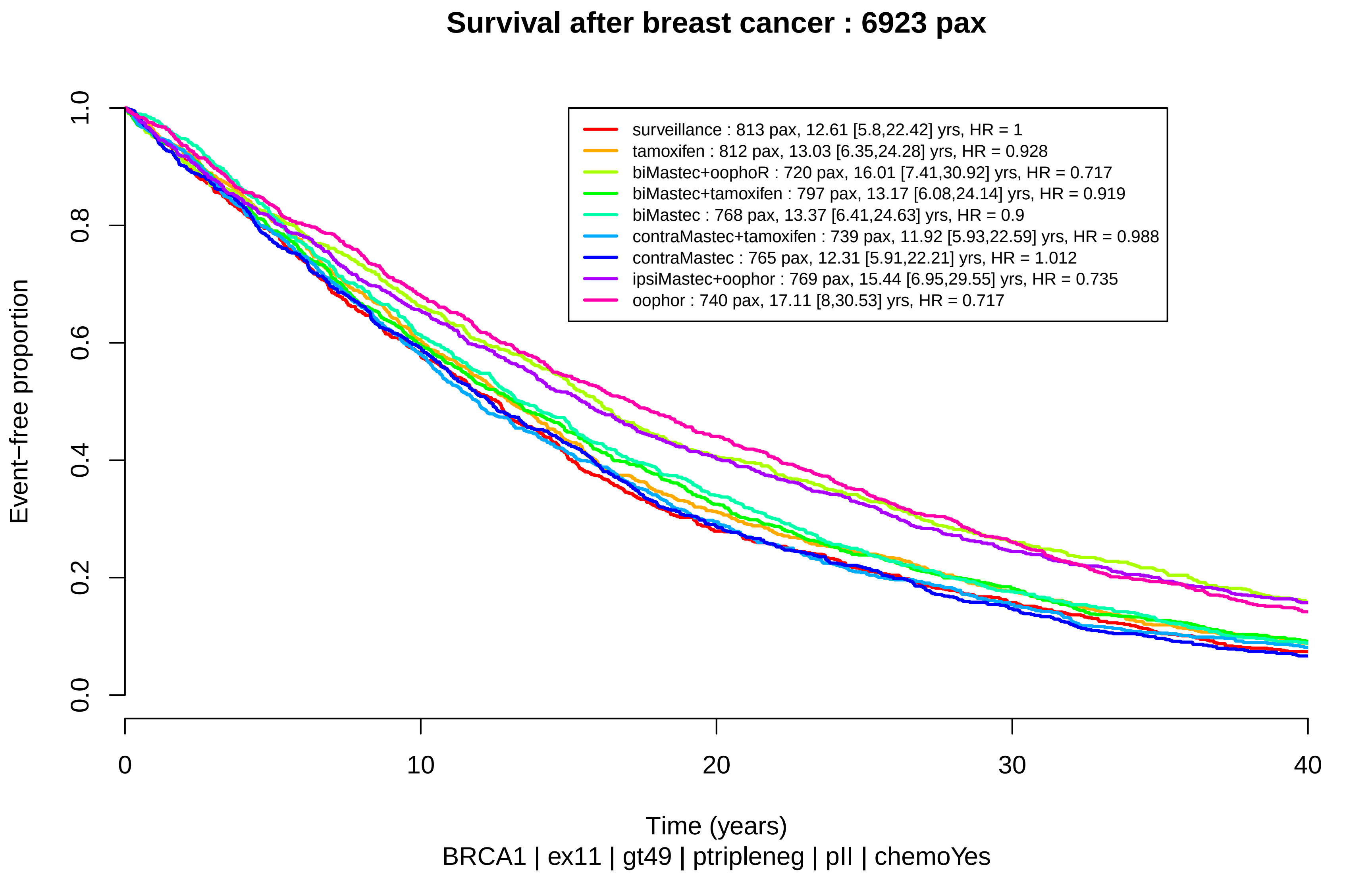

Survival after breast cancer : 6923 pax
surveillance : 813 pax, 12.61 [5.8,22.42] yrs, HR = 1
tamoxifen : 812 pax, 13.03 [6.35,24.28] yrs, HR = 0.928
biMastec+oophoR : 720 pax, 16.01 [7.41,30.92] yrs, HR = 0.717
biMastec+tamoxifen : 797 pax, 13.17 [6.08,24.14] yrs, HR = 0.919
biMastec : 768 pax, 13.37 [6.41,24.63] yrs, HR = 0.9
contraMastec+tamoxifen : 739 pax, 11.92 [5.93,22.59] yrs, HR = 0.988
contraMastec : 765 pax, 12.31 [5.91,22.21] yrs, HR = 1.012
ipsiMastec+oophor : 769 pax, 15.44 [6.95,29.55] yrs, HR = 0.735
oophor : 740 pax, 17.11 [8,30.53] yrs, HR = 0.717
Event−free proportion
0.0
0.2
0.4
0.6
0.8
1.0
0
10
20
30
40
Time (years)
BRCA1 | ex11 | gt49 | ptripleneg | pII | chemoYes

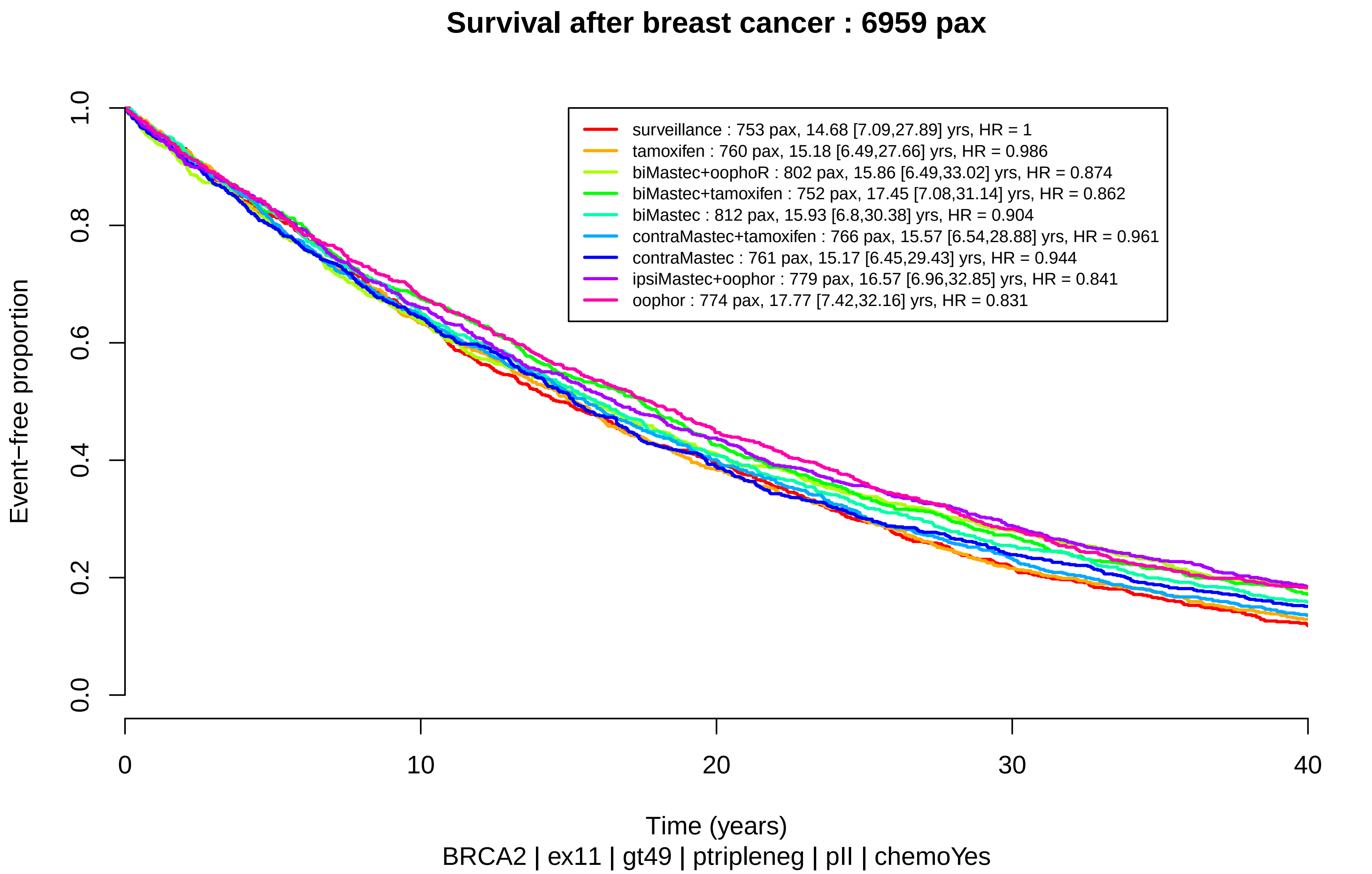
Survival after breast cancer : 6959 pax
Event−free proportion
0.0
0.2
0.4
0.6
0.8
1.0
0
10
20
30
40
Time (years)
BRCA2 | ex11 | gt49 | ptripleneg | pII | chemoYes
surveillance : 753 pax, 14.68 [7.09,27.89] yrs, HR = 1
tamoxifen : 760 pax, 15.18 [6.49,27.66] yrs, HR = 0.986
biMastec+oophoR : 802 pax, 15.86 [6.49,33.02] yrs, HR = 0.874
biMastec+tamoxifen : 752 pax, 17.45 [7.08,31.14] yrs, HR = 0.862
biMastec : 812 pax, 15.93 [6.8,30.38] yrs, HR = 0.904
contraMastec+tamoxifen : 766 pax, 15.57 [6.54,28.88] yrs, HR = 0.961
contraMastec : 761 pax, 15.17 [6.45,29.43] yrs, HR = 0.944
ipsiMastec+oophor : 779 pax, 16.57 [6.96,32.85] yrs, HR = 0.841
oophor : 774 pax, 17.77 [7.42,32.16] yrs, HR = 0.831

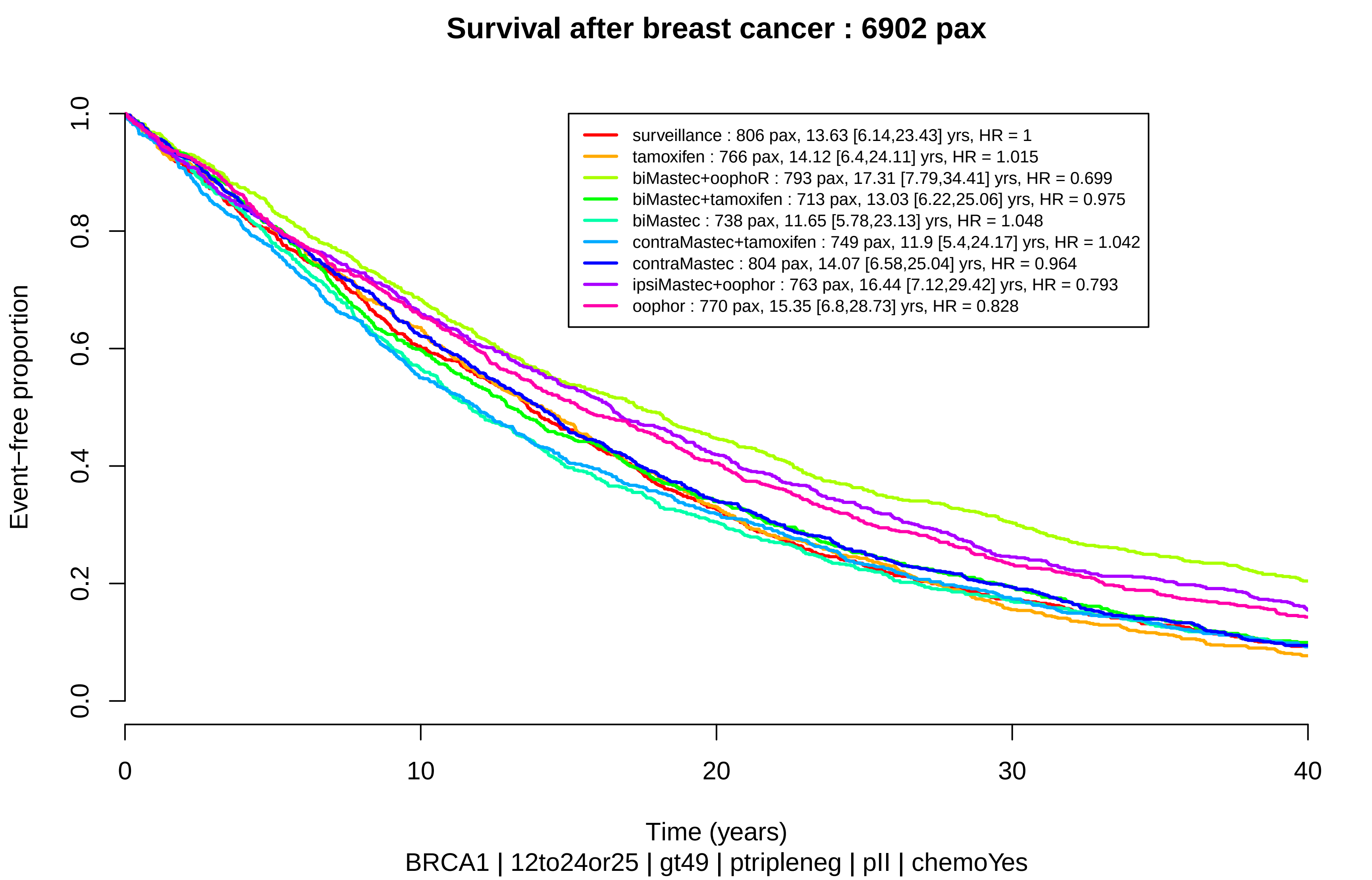

Survival after breast cancer : 6902 pax
surveillance : 806 pax, 13.63 [6.14,23.43] yrs, HR = 1
tamoxifen : 766 pax, 14.12 [6.4,24.11] yrs, HR = 1.015
biMastec+oophoR : 793 pax, 17.31 [7.79,34.41] yrs, HR = 0.699
biMastec+tamoxifen : 713 pax, 13.03 [6.22,25.06] yrs, HR = 0.975
biMastec : 738 pax, 11.65 [5.78,23.13] yrs, HR = 1.048
contraMastec+tamoxifen : 749 pax, 11.9 [5.4,24.17] yrs, HR = 1.042
contraMastec : 804 pax, 14.07 [6.58,25.04] yrs, HR = 0.964
ipsiMastec+oophor : 763 pax, 16.44 [7.12,29.42] yrs, HR = 0.793
oophor : 770 pax, 15.35 [6.8,28.73] yrs, HR = 0.828
Event−free proportion
0.0
0.2
0.4
0.6
0.8
1.0
0
10
20
30
40
Time (years)
BRCA1 | 12to24or25 | gt49 | ptripleneg | pII | chemoYes

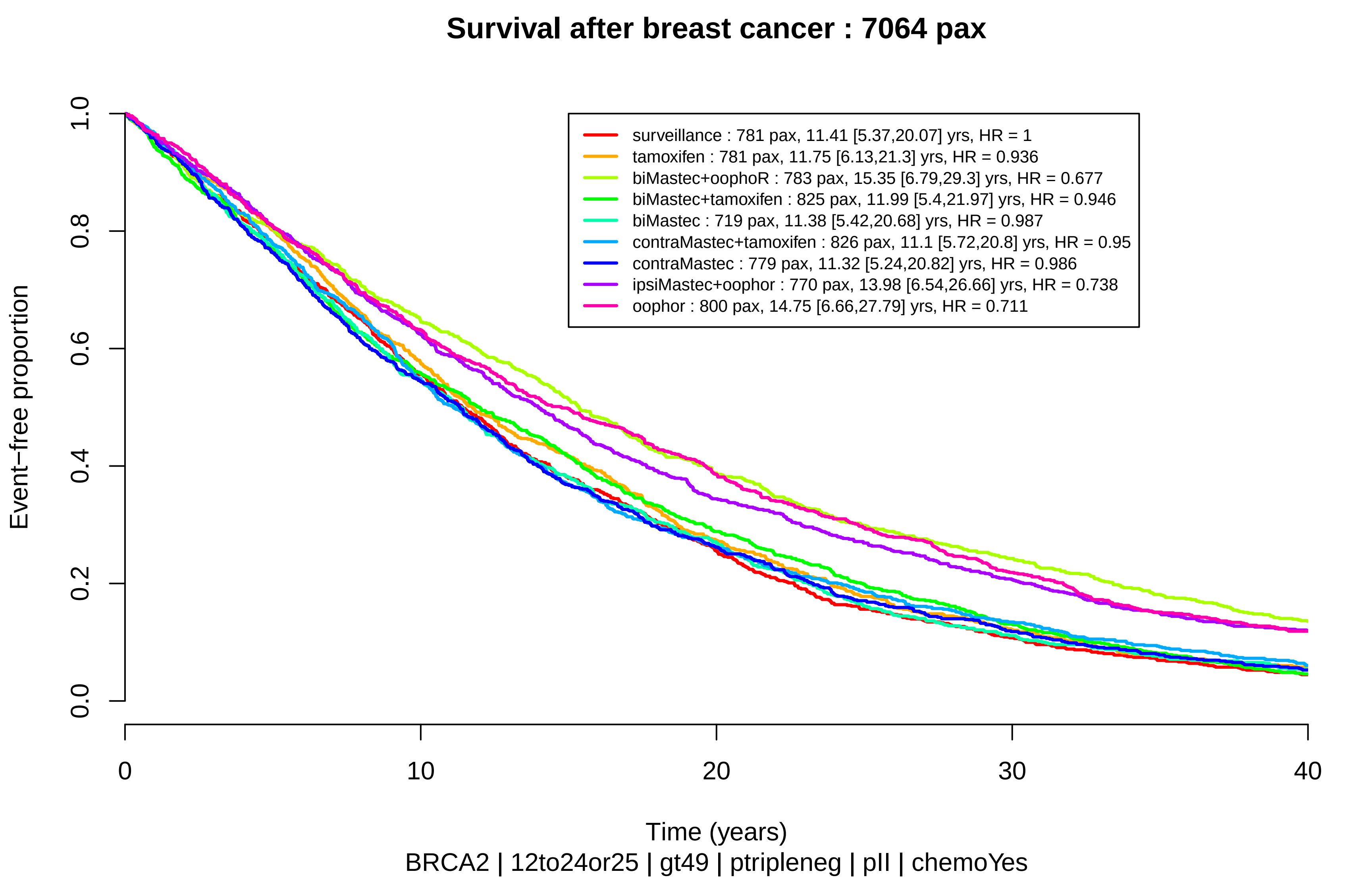
Survival after breast cancer : 7064 pax
surveillance : 781 pax, 11.41 [5.37,20.07] yrs, HR = 1
tamoxifen : 781 pax, 11.75 [6.13,21.3] yrs, HR = 0.936
biMastec+oophoR : 783 pax, 15.35 [6.79,29.3] yrs, HR = 0.677
biMastec+tamoxifen : 825 pax, 11.99 [5.4,21.97] yrs, HR = 0.946
biMastec : 719 pax, 11.38 [5.42,20.68] yrs, HR = 0.987
contraMastec+tamoxifen : 826 pax, 11.1 [5.72,20.8] yrs, HR = 0.95
contraMastec : 779 pax, 11.32 [5.24,20.82] yrs, HR = 0.986
ipsiMastec+oophor : 770 pax, 13.98 [6.54,26.66] yrs, HR = 0.738
oophor : 800 pax, 14.75 [6.66,27.79] yrs, HR = 0.711
Event−free proportion
0.0
0.2
0.4
0.6
0.8
1.0
0
10
20
30
40
Time (years)
BRCA2 | 12to24or25 | gt49 | ptripleneg | pII | chemoYes
