# Supplementary material for: Effectiveness of Secondary Risk–Reducing Strategies in Patients With Unilateral Breast Cancer With Pathogenic Variants of BRCA1 and BRCA2 Subjected to Breast-Conserving Surgery: Evidence-Based Simulation Study
Source: JMIR Form Res. 2022 Dec 29;6(12):e37144. doi: 10.2196/37144 (PMC9837710; doi:10.2196/37144)
Supplement: Multimedia Appendix 2 [file formative_v6i12e37144_app2.docx]

| Variable | | | | | | | | | | | % | Hazard ratios and 95% CI derived from Cox regression | Data source |
| --- | --- | --- | --- | --- | --- | --- | --- | --- | --- | --- | --- | --- | --- |
| **Primary breast cancer** | | | | | | | | | | |  |  |  |
|  | 5-year breast cancer–specific mortality rate | | | | | | | | | |  |  | 50 |
|  | Luminal-like | | | | | | | | | |  |  |  |
|  | Stage I | | | | | | | | | | 1.2 | 1.00 |  |
|  | Stage II | | | | | | | | | | 3.9 | 1.00 |  |
|  | Triple negative | | | | | | | | | |  |  |  |
|  | Stage I | | | | | | | | | | 7.1 | 4.68 (4.06-5.38) |  |
|  | Stage II | | | | | | | | | | — | 3.93 (3.63-4.26) |  |
| **Contralateral breast cancer** | | | | | | | | | | |  |  |  |
|  | | ***BRCA1* carriers** | | | | | | | | |  |  | 23 |
|  | |  | **<40 years at first breast cancer** | | | | | | | |  |  |  |
|  | |  |  | | 5 | | | | | | — | 14.1 (10.1-18.0) |  |
|  | |  |  | | 10 | | | | | | — | 30.1 (24.0-36.2) |  |
|  | |  |  | | 15 | | | | | | — | 40.8 (33.2-48.3) |  |
|  | |  |  | | 25 | | | | | | — | 55.1 (45.4-65.9) |  |
|  | |  | **40-49 years at first breast cancer** | | | | | | | |  |  |  |
|  | |  |  | | 5 | | | | | | — | 9.2 (5.8-12.5) |  |
|  | |  |  | | 10 | | | | | | — | 16.7 (11.7-21.7) |  |
|  | |  |  | | 15 | | | | | | — | 23.2 (16.9-29.6) |  |
|  | |  |  | | 25 | | | | | | — | 44.5 (33.2-55.7) |  |
|  | |  | **>50 years at first breast cancer** | | | | | | | |  |  |  |
|  | |  |  | | 5 | | | | | | — | 7.1 (3.8-10.5) |  |
|  | |  |  | | 10 | | | | | | — | 11.4 (6.5-16.3) |  |
|  | |  |  | | 15 | | | | | | — | 18.7 (11.0-26.3) |  |
|  | |  |  | | 25 | | | | | | — | 21.6 (12.3-30.8) |  |
|  | | ***BRCA2* carriers** | | | | | | | | |  |  |  |
|  | |  | **<40 years at first breast cancer** | | | | | | | |  |  |  |
|  | |  |  | | 5 | | | | | | — | 2.9 (0.0-6.3) |  |
|  | |  |  | | 10 | | | | | | — | 18.2 (7.9-28.5) |  |
|  | |  |  | | 15 | | | | | | — | 20.9 (9.7-32.1) |  |
|  | |  |  | | 25 | | | | | | — | 38.5 (18.5-58.2) |  |
|  | |  | **40-49 years at first breast cancer** | | | | | | | |  |  |  |
|  | |  |  | | 5 | | | | | | — | 6.9 (2.7-11.1) |  |
|  | |  |  | | 10 | | | | | | — | 13.4 (7.0-19.8) |  |
|  | |  |  | | 15 | | | | | | — | 22.0 (12.1-31.9) |  |
|  | |  |  | | 25 | | | | | | — | 40.5 (22.2-58.6) |  |
|  | |  | **>50 years at first breast cancer** | | | | | | | |  |  |  |
|  | |  |  | | 5 | | | | | | — | 3.5 (0.9-6.1) |  |
|  | |  |  | | 10 | | | | | | — | 10.4 (4.9-16.0) |  |
|  | |  |  | | 15 | | | | | | — | 15.5 (7.8-23.3) |  |
|  | |  |  | | 25 | | | | | | — | 15.5 (7.8-23.3) |  |
|  | | **Disease stage at diagnosis** | | | | | | | | |  |  | 61 |
|  | |  | **Ductal carcinoma in situ** | | | | | | | | 25 | — |  |
|  | |  |  | | I stage | | | | | | 42 | — |  |
|  | |  |  | | II stage | | | | | | 19 | — |  |
|  | |  |  | | III stage | | | | | | 7 | — |  |
|  | |  |  | | IV stage | | | | | | 7 | — |  |
|  | | **20-year contralateral breast cancer–specific mortality rate** | | | | | | | | |  |  |  |
|  | |  | Ductal carcinoma in situ | | | | | | | | 3.3 | — | 58 |
|  | | **5-year contralateral breast cancer–specific mortality rate** | | | | | | | | |  |  | 52 |
|  | |  | **Luminal-like** | | | | | | | |  |  |  |
|  | |  |  | | Stage I | | | | | | 1.2 | — |  |
|  | |  |  | | Stage II | | | | | | 3.9 | — |  |
|  | |  |  | | Stage III | | | | | | 15 | — |  |
|  | |  |  | | **Stage IV** | | | | | |  |  | 59 |
|  | |  |  | |  | | | | *BRCA1* | | 74.1 | — |  |
|  | |  |  | |  | | | | *BRCA2* | | 57.1 | — |  |
|  | |  | **Triple negative** | | | | | | | |  |  |  |
|  | |  |  | | Stage I | | | | | | 7.1 | 4.68 (4.06-5.38) |  |
|  | |  |  | | Stage II | | | | | | — | 3.93 (3.63-4.26) |  |
|  | |  |  | | Stage III | | | | | | 51.1 | 4.45 (4.02-4.93) |  |
|  | |  |  | | **Stage IV** | | | | | |  |  | 59 |
|  | |  |  | |  | | | | *BRCA1* | | 74.1 | — |  |
|  | |  |  | |  | | | | *BRCA2* | | 57.1 |  |  |
| **Ipsilateral breast event** | | | | | | | | | | |  |  |  |
|  | | **Cumulative incidence** | | | | | | | | |  |  | 5 |
|  | |  | 5 years | | | | | | | | 4.1 | — |  |
|  | |  | 10 years | | | | | | | | 10.5 | — |  |
|  | |  | 15 years | | | | | | | | 23.5 | — |  |
|  | |  | 20 years | | | | | | | | 30.2 | — |  |
|  | | **Protective risk factors** | | | | | | | | |  |  | 33 |
|  | |  | Bilateral prophylactic oophorectomy | | | | | | | | — | 0.42 (0.22-0.81) |  |
|  | |  | Adjuvant chemotherapy | | | | | | | | — | 0.51 (0.31-0.84) |  |
|  | |  | Disease stage at diagnosis | | | | | | | |  |  | 61 |
|  | | **Ductal carcinoma in situ** | | | | | | | | | 25 | — |  |
|  | |  | I stage | | | | | | | | 42 | — |  |
|  | |  | II stage | | | | | | | | 19 | — |  |
|  | |  | III stage | | | | | | | | 7 | — |  |
|  | |  | IV stage | | | | | | | | 7 | — |  |
|  | | **20-year ipsilateral breast cancer–specific mortality rate** | | | | | | | | |  |  |  |
|  | |  | Ductal carcinoma in situ | | | | | | | | 3.3 | — | 61 |
|  | | **5-year ipsilateral breast cancer–specific mortality rate** | | | | | | | | |  |  | 50 |
|  | |  | **Luminal-like** | | | | | | | |  |  |  |
|  | |  |  | | Stage I | | | | | | 1.2 | — |  |
|  | |  |  | | Stage II | | | | | | 3.9 | — |  |
|  | |  |  | | Stage III | | | | | | 15 | — |  |
|  | |  |  | | **Stage IV** | | | | | |  |  | 59 |
|  | |  |  | |  | | | *BRCA1* | | | 74.1 | — |  |
|  | |  |  | |  | | | *BRCA2* | | | 57.1 | — |  |
| **Triple negative** | | | | | | | | | | |  |  |  |
|  | | Stage I | | | | | | | | | 7.1 | 4.68 (4.06-5.38) |  |
|  | | Stage II | | | | | | | | |  | 3.93 (3.63-4.26) |  |
|  | | Stage III | | | | | | | | | 51.1 | 4.45 (4.02-4.93) |  |
|  | | **Stage IV** | | | | | | | | |  |  | 59 |
|  | |  | | *BRCA1* | | | | | | | 74.1 | — |  |
|  | |  | | *BRCA2* | | | | | | | 57.1 | — |  |
| **Ovarian cancer** | | | | | | | | | | |  |  |  |
|  | | **Lifetime incidence** | | | | | | | | |  |  | 52 |
|  | |  | | ***BRCA1*** | | | | | | |  |  |  |
|  | |  | |  | | | Exons 1-10 | | | | 18.2 | — |  |
|  | |  | |  | | | Exon 11 | | | | 36.4 | — |  |
|  | |  | |  | | | Exons 12-24 | | | | 36.4 | — |  |
|  | |  | | ***BRCA2*** | | | | | | |  |  |  |
|  | |  | |  | | | Exons 1-10 | | | | 13.4 | — |  |
|  | |  | |  | | | Exons 11 | | | | 33.3 | — |  |
|  | |  | |  | | | Exons 12-25 | | | | 53.3 |  |  |
|  | | **Disease stage at diagnosis** | | | | | | | | |  |  | 53 |
|  | |  | | Stage I | | | | | | | 20 | — |  |
|  | |  | | Stage II | | | | | | | 5 | — |  |
|  | |  | | Stage III | | | | | | | 58 | — |  |
|  | |  | | Stage IV | | | | | | | 17 |  |  |
|  | | **10-year ovarian cancer mortality** | | | | | | | | |  |  |  |
|  | |  | | Stage I | | | | | | | 27 | — |  |
|  | |  | | Stage II | | | | | | | 55 | — |  |
|  | |  | | Stage III | | | | | | | 79 | — |  |
|  | |  | | Stage IV | | | | | | | 95 | — |  |
|  | | **Prevention strategies** | | | | | | | | |  |  |  |
|  | |  | | **Prophylactic contralateral mastectomy** | | | | | | |  |  |  |
|  | |  | |  | | Breast cancer risk reduction | | | | | 91 | — | 67 |
|  | |  | |  | | Surgical mortality | | | | | 0.24 | — | 71 |
|  | |  | | **Prophylactic ipsilateral mastectomy** | | | | | | |  |  |  |
|  | |  | |  | | **Cumulative incidence** | | | | |  |  | 5 |
|  | |  | |  | |  | | | | 5 years | 1.4 | —^a^ |  |
|  | |  | |  | |  | | | | 10 years | 3.5 | — |  |
|  | |  | |  | |  | | | | 15 years | 5.5 | — |  |
|  | |  | |  | |  | | | | 20 years | 5.5 | — |  |
|  | | **Protective risk factors** | | | | | | | | |  |  | 36 |
|  | |  | | Bilateral prophylactic oophorectomy | | | | | | | — | 0.42 (0.22-0.81) |  |
|  | |  | | Adjuvant chemotherapy | | | | | | | — | 0.51 (0.31-0.84) |  |
|  | |  | | Surgical mortality | | | | | | | 0.24 | — | 71 |
|  | | **Prophylactic bilateral salpingo-oophorectomy** | | | | | | | | |  |  |  |
|  | |  | | **Ovarian cancer risk reduction** | | | | | | |  |  |  |
|  | |  | |  | | *BRCA1* carriers | | | | | 80 | 0.20 (0.12-0.32) | 19 |
|  | |  | |  | | *BRCA2* carriers | | | | | 79 | 0.21 (0.02-0.191) | 19 |
|  | |  | | **Breast cancer risk reduction** | | | | | | | 56 | 0.46 (0.27-0.79) |  |
|  | |  | |  | | Excess mortality from noncancer (vascular, neurological or mental, other) causes for patients with first breast cancer onset before the age of 40 years | | | | | — | 1.71 (0.95–3.09) | 66 |
|  | | **5 years of daily tamoxifen** | | | | | | | | |  |  |  |
|  | |  | | Risk of second primary contralateral breast cancer | | | | | | |  |  |  |
|  | | ***BRCA1* pathogenic variant** | | | | | | | | |  |  | 32 |
|  | |  | | Adjusted for estrogen receptor status, age at diagnosis, year of diagnosis, bilateral prophylactic oophorectomy | | | | | | | — | 0.44 (0.25-0.85) |  |
|  | | ***BRCA2* pathogenic variant** | | | | | | | | |  |  | 32 |
|  | |  | | Adjusted for estrogen receptor status, age at diagnosis, year of diagnosis, bilateral prophylactic oophorectomy | | | | | | | — | 0.33 (0.17-0.64) |  |

^a^Not available
